# Metabolic Responses of *Amaranthus caudatus* Roots and Leaves to Zinc Stress

**DOI:** 10.3390/plants14142119

**Published:** 2025-07-09

**Authors:** Natalia Osmolovskaya, Tatiana Bilova, Anastasia Gurina, Anastasia Orlova, Viet D. Vu, Stanislav Sukhikh, Tatiana Zhilkina, Nadezhda Frolova, Elena Tarakhovskaya, Anastasia Kamionskaya, Andrej Frolov

**Affiliations:** 1 Department of Plant Physiology and Biochemistry, St. Petersburg State University, 199034 St. Petersburg, Russia; bilova.tatiana@gmail.com (T.B.); anastasia.gurina@list.ru (A.G.); vuvietdung01031982@gmail.com (V.D.V.); elena.tarakhovskaya@gmail.com (E.T.); 2 Laboratory of Analytical Biochemistry and Biotechnology, K.A. Timiryazev Institute of Plant Physiology of the Russian Academy of Science, 127276 Moscow, Russia; orlova@ifr.moscow (A.O.); frolov@ifr.moscow (A.F.); 3 Coast Branch—Vietnam Russian Tropical Center, Nha Trang City 650000, Vietnam; 4 Laboratory of Microbiology and Biotechnology, Immanuel Kant Baltic Federal University, 236041 Kaliningrad, Russia; ssukhikh@kantiana.ru; 5 Federal Research Centre Fundamentals of Biotechnology of the Russian Academy of Science, 119071 Moscow, Russia; timofeeva.bio@gmail.com (T.Z.); akamio@fbras.ru (A.K.); 6 Vavilov Institute of General Genetics, St. Petersburg Branch, Russian Academy of Sciences, 199034 St. Petersburg, Russia

**Keywords:** *Amaranthus caudatus*, complexation of Zn^2+^ ions, GC-MS-based metabolomics, gluconate, heavy metals, metabolic adjustment, osmotic regulation, ROS scavenging, salicylate, Zn stress

## Abstract

In recent decades, heavy metal pollution has become a significant environmental stress factor. Plants are characterized by high biochemical plasticity and can adjust their metabolism to ensure survival under a changing environment. Here we report, to our knowledge, the first gas chromatography-mass spectrometry (GC-MS)-based metabolomics study of Zn-induced stress responses in *Amaranthus caudatus* plants. The study was performed with root and leaf aqueous methanolic extracts after their lyophilization and sequential derivatization with methoxylamine hydrochloride and *N*-methyl-*N*-(trimethylsilyl)trifluoroacetamide. In total, 419 derivatives were detected in the samples, and 144 of them could be putatively annotated. The metabolic shifts in seven-week-old *A. caudatus* plants in response to a seven-day treatment with 300 µmol/L ZnSO_4_·7H_2_O in nutrient solution were organ-specific and more pronounced in roots. Most of the responsive metabolites were up-regulated and dominated by sugars and sugar acids. The revealed effects could be attributed to the involvement of these metabolites in osmotic regulation, antioxidant protection and Zn^2+^ complexation. A 59-fold up-regulation of gluconic acid in roots distinctly indicated enhanced glucose oxidation due to oxidative stress upon the Zn treatment. Gluconic acid might be further employed in Zn^2+^ complexation. Pronounced Zn-induced up-regulation of salicylic acid in roots and shoots suggested a key role of this hormone in stress signaling and activation of Zn stress tolerance mechanisms. Overall, our study provides the first insight into the general trends of Zn-induced biochemical rearrangements and main adaptive metabolic shifts in *A. caudatus*.

## 1. Introduction

In nature, plants are constantly exposed to a variety of abiotic stresses, including soil contamination with heavy metals (HM) due to the progressing anthropogenic activities [1]. An excess of HM in soils ultimately leads to pronounced physiological and metabolic disturbances in crops, accompanied by a decrease in their productivity and quality [2,3]. The damaging effects of HMs are generally explained by the high reactivity and toxicity of their ions, which can be primarily manifested in their direct interactions with proteins and enhanced production of ROS [4,5]. However, plants exhibit remarkable tolerance and high potential for adaptation to environmental stressors due to their impressive biochemical plasticity [6]. Crop plants essentially differ in their tolerance to HMs in the soil, which relies on a broad range of adaptive mechanisms, including those implemented at the biochemical level [7]. Stress-induced rearrangement of cellular metabolism, also referred to as a metabolic adjustment, represents one of the most important adaptive strategies [8]. The stress-related dynamics of some selected metabolites can be addressed by standardized chromatographic methods [9]. However, in a more efficient way, stress-induced metabolic shifts can be characterized by the state-of-the-art untargeted metabolomics approaches [10,11], which provide access to the molecular mechanisms of the stress response [12].

Currently, metabolomics is recognized as the method of choice to study the complex mechanisms underlying plant responses to HM stress [13]. Because of the complexity of the plant metabolome, its full coverage cannot be achieved with only one instrumental platform [14]. Among the full array of comprehensive profiling methods, GC-MS and LC-MS are complementary powerful metabolomics techniques providing an analysis of primarily metabolites (most often as trimethylsilyl—TMS derivatives) and secondary metabolites, respectively [14,15]. To date, the metabolomics studies of the HM stress responses have focused mostly on the impact of Cd in a limited selection of plants, namely *Arabidopsis thaliana* [16,17], *Brassica juncea* [18], *Cynodon dactylon* [19], *Raphanus sativus* [15] and *Lycopersicon esculentum* [20], whereas the effects of other HMs, including such a widely spread element as Zn, have been only minimally addressed so far [21,22]. Zn naturally presents in the environment as a trace metal [23] and is an essential micronutrient mandatory for plant metabolism and growth [24,25]. For most agricultural soils, total Zn contents are usually in the range of 10–300 ppm, while the concentrations of bioavailable Zn^2+^ in bulk soil solutions are much lower and typically do not exceed 4.0 µmol/L [26]. However, in Zn-polluted soils, the content of this metal can increase dramatically, affecting principal physiological parameters and ultimately inducing strong toxicity responses in plants [24,25]. Multiple toxic manifestations of Zn excess in plants caused by disorders of photosynthesis, respiration, water status, mineral nutrition and redox balance were comprehensively reviewed recently by Kaur and Garg [25]. According to studies conducted in solution culture, the threshold concentrations of Zn^2+^, inducing phytotoxic symptoms, ranged from 25–50 µmol/L to 250─500 µmol/L, strongly depending on plant species, developmental stage, duration of exposure and environmental conditions [23,24,25]. The narrow gap between the levels corresponding to Zn essentiality and toxicity represents an important feature of Zn physiology in various plant species [25].

Till now, characterization of the metabolic shifts induced by Zn stress has relied mostly on routine methods of targeted biochemical analysis. Such experiments typically covered only selected primary metabolites and addressed just a limited number of plant species [9,27,28]. Thus, the application of 0.5 mmol/L Zn^2+^ to *Triticum aestivum* and *Lactuca sativa* plants induced accumulation of a characteristic set of stress-related metabolites (proline, glycine betaine, soluble sugars, and free amino acids) [9,28]. This observation might indicate the involvement of these compounds in osmotic adjustment, which is critical for plant survival under Zn stress [9,28]. However, the same Zn dosage applied to *Brassica oleracea* resulted in pronounced down-regulation of proline and glycine betaine, while the content of GABA in plant tissues increased [9]. In sugar beet, overexposure to Zn induced essential shifts in carboxylate metabolism, which were manifested with up-regulation of organic acids, most strongly pronounced for citrate and malate [27]. This observation is in agreement with the fact that these highly efficient HM-chelators underlie one of the key mechanisms of HM tolerance in plants [29,30,31]. Obviously, implementation of the untargeted metabolomics approach would provide much better insight into the plant metabolic responses associated with Zn stress and the mechanisms conferring plant tolerance to this metal.

Due to their pronounced tolerance to environmental stresses and well-characterized potential for HM phytoremediation, *Amaranthus* species attract a special interest in the context of Zn stress [32,33,34], including Zn phytoextraction [35] and Zn phytostabilization [36]. These features of amaranth can be explained by its outstanding phenotypic plasticity, extreme adaptability to adverse environmental conditions and ability for efficient growth on agriculturally unfavorable territories [37]. Moreover, due to its high nutritional quality [38] and rich pattern of biologically active secondary metabolites [39,40], amaranth has been recognized as “a new millennium crop of nutraceutical values” [37]. Regarding the primary metabolism, amaranth species are featured with C4 type of photosynthesis [41] and enhanced production of oxalic acid [42,43]. However, to date, this genus is poorly characterized in terms of metabolic rearrangements under HM stress conditions, and comprehensive metabolomics studies of Zn-induced responses in amaranth are still lacking.

Therefore, here, we present a gas chromatography-mass spectrometry (GC-MS)-based metabolomics study addressing adaptive metabolic adjustment as a primary response of *A. caudatus* roots, young and mature leaves, to Zn stress. Such an approach seems to be adequate for a better understanding of the mechanisms underlying HM tolerance at the whole-plant level, as well as possible age-related changes in the dynamics of metabolic adjustment accompanying the tolerance onset. To address the stress-related dynamics of low-molecular-weight metabolites, we decided on GC-MS of their trimethylsylilated (TMS) derivatives. Due to the high chromatographic resolution, sensitivity and specificity of this technique, efficient and reliable detection and quantification of the metabolites critically involved in the stress-related metabolic adjustment (mono- and disaccharides, polyols, carboxylic acids, amino acids, etc.) could be achieved. We assume that these metabolites could act as osmoprotectors, chelating agents and regulators, maintaining the balance between biosynthetic and energy metabolic pathways under Zn stress.

## 2. Results

### 2.1. Physiological Responses of A. caudatus Plants to Zn-Exposure

*A. caudatus* plants grown in a hydroponic culture at the age of six weeks were exposed for a week to 300 μmol/L ZnSO_4_ added to a nutrient solution. Over this period, the plants did not show any serious alterations in root and leaf morphology in comparison to the untreated controls (Supplementary Information S1, Figure S1(1,2)). The only visible Zn-related effect could be observed for the young leaves, which turned light green upon the treatment (Figure S1(1A)). This, however, was not the case with the mature (i.e., fully expanded) leaves, which remained dark green even after Zn application. The non-destructive measurements of chlorophyll content, accomplished with the 3rd mature leaf, also did not reveal any Zn-induced enhancement of chlorophyll degradation. However, application of the Zn stress resulted in a low (approximately 6%) but significant decrease in the efficiency of photosystem II (PSII). On the other hand, the Zn treatment had no effect on the water budget of the amaranth plants: both stomatal conductivity and leaf relative water content (LRWC) did not show any alterations associated with Zn application (Figure S1(3A,4A)). These patterns of the plant morphological and physiological responses to Zn application were confirmed in the second independent experiment (Figure S1(2,3B,4B)).

As no inter-experimental differences in the phenotypes and physiological responses could be observed in two independent plant experiments (Figure S1(1–4), Supplementary Information S2, Table S2(1–4)), dynamics of metal content in tissues and levels of individual low-molecular weight metabolites upon the Zn stress application were measured for only one of them.

### 2.2. Patterns of Low-Molecular Weight Metabolites Detected by GC-MS in A. caudatus Leaves and Roots and Their Dynamics Upon Treatment with Zn^2+^

Analysis of the low-molecular-weight metabolites (represented mostly by primary compounds and low-molecular-weight phenolics) in the leaves and roots of *A. caudatus* was accomplished with gas chromatography-electron ionization-quadrupole-mass spectrometry (GC-EI-Q-MS). Despite the high potential of LC-MS for analysis of both primary and secondary metabolites, we decided here on GC-MS as this method allows targeting the compounds involved in the stress-protective metabolic adjustment (amino acids and carboxylic acids, polyols and sugars) in the most efficient way. In this context, GC-MS appeared to be completely sufficient for achieving the objectives of this study. The resulting patterns of metabolites were first compared at the qualitative level, i.e., the principal differences in the leaf and root responses to Zn stress were addressed.

In total, 419 individual trimethylsilyl (TMS) and methyloxime (MEOX)/TMS derivatives (i.e., chromatographic signals which we define here as features) were detected in the GC-MS data acquired for the leaf and root extracts of control and Zn^2+^-treated plants (Supplementary Information S1, Table S1(1)). Some annotated metabolites were represented by several features, which could result from different contributions of MEOX and TMS groups or sin/anti isomerism of sugar TMS-metoximes. Among them, 26 features could be unambiguously identified by electron ionization mass spectral (EI-MS) similarity search and co-elution with 21 authentic standards, whereas the other 118 features were putatively structurally annotated by EI-MS and retention index (RI) similarity search against available EI-MS public libraries (NIST, GMD) and in-house spectral libraries. Since in the GC-MS chromatograms some analytes were represented with several peaks of isomers, TMS or MEOX/TMS derivatives, the overall number of structurally annotated metabolites was 128. These metabolites could be assigned to the following classes: organic acids (di- and tricarboxylic acids of TCA cycle, other hydroxyacids and dicarboxylic acids, sugar acids, phenolic acids), short- and long-chain fatty acids, N-metabolites (amides, amines, proteinogenic and non-proteinogenic amino acids, purines/pyrimidines, nucleosides), sugars, polyols, phosphate-conjugated metabolites (first of all, sugar phosphates), and terpenes.

Annotation of the further 107 analytes relied on the presence of characteristic fragment ions (*m*/*z* ± 0.5 Da) in the EI mass spectra which might serve as indicators for the following substance classes [44]: fragment ions of *m*/*z* 174 and 100 prospectively indicated amino group-containing compounds; *m/z* 103, 160, 217, 319 were characteristic for C5- and C6-monosaccharides; *m*/*z* 319 and 204 for sugar alcohols, *m*/*z* 318 and 319 for stereoisomers of inositol, *m*/*z* 333, 292 and 319 for sugar acids, *m*/*z* 361, 437 or/and 451 for di- and oligosaccharides; *m*/*z* 299, 315, 357 or/and 387 for organic phosphates. An essential part (41%) of the metabolite pattern included yet uncharacterized metabolite features, which did not yield any match in public and in-house EI-MS databases. Most of these metabolites were detected in all inter-group paired comparisons (i.e., leaves and roots from Zn^2+^-treated vs. untreated plants). However, about 24 metabolites (boric acid, urea, 1-nitroso-3,5-dinitro-hexahydro-1,3,5-triazine, two unknown amines and 19 further unknowns) were missing (i.e., not detected under our experimental conditions) in one or more experimental groups. In particular, boric acid was not detected in young leaves and roots of the control plants, whereas urea was not detected in young leaves of Zn^2+^-treated plants.

**Figure 1:**
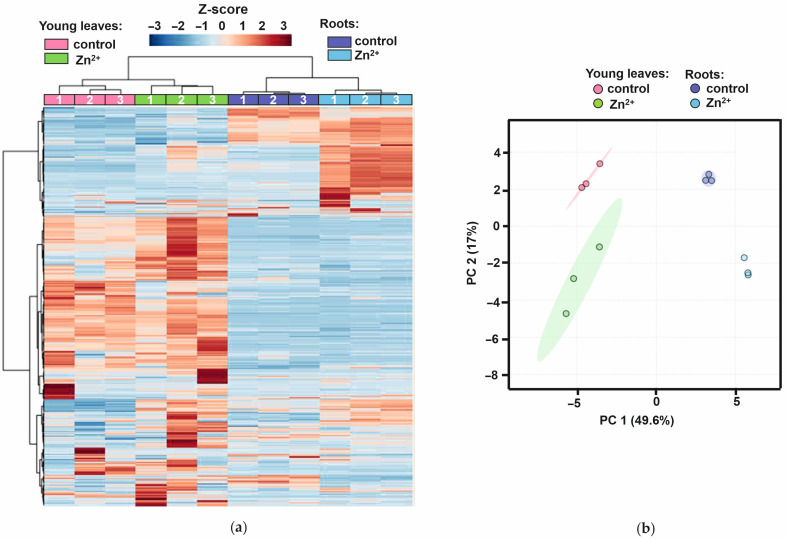
Results of hierarchical clustering with heat map representation (**a**) and principal component analysis (PCA), score plot built for the first two principal components, (**b**) accomplished for abundances of all 419 individual metabolites (features) detected as trimethylsilyl (TMS) and TMS-methyl oxime (MEOX) derivatives by untargeted GC-EI-Q-MS of aq. methanolic extracts of *A. caudatus* young leaves and roots. The PCA and Heatmap were built in MetaboAnalyst 4.0 (https://www.metaboanalyst.ca, accessed on 16 March 2020).

The relative abundances of individual metabolites detected in young leaves and roots of *A. caudatus* plants in the absence and presence of Zn stress were visualized by hierarchical clustering with heat map representation (Figure 1a). Although no significant stress-associated changes in leaf relative water contents, root and leaf wet and dry weights could be observed (Supplementary Information S1, Figure S1(4,5) and Supplementary Information S2, Table S2(4–6)), the metabolic responses in both leaves and roots demonstrated clear stress-induced patterns dominated with organic acids (predominantly sugar acids), sugars and, to some extent, amino acids (Table 1 and Table 2). Since all these compounds are well-known stress metabolites, the stress state of Zn-treated plants can further be considered as unambiguously confirmed.

**Figure 2:**
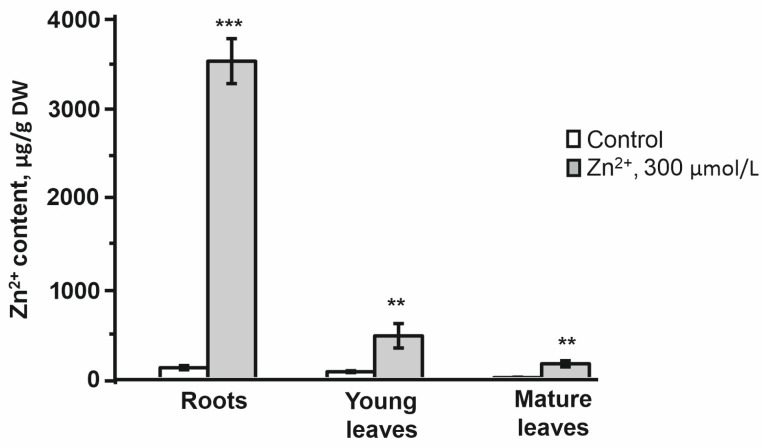
Impact of exogenous Zn^2+^ (300 μmol/L) on the contents of Zn^2+^ in roots, young and mature leaves harvested from seven-week-old *A. caudatus* plants (*n* = 3) grown in hydroponic nutrient solution in the presence (Zn^2+^-group) and absence (control group) of 300 μmol/L ZnSO_4_ for 1 week. ** and *** denote *p*-value (Student’s test) <0.01 and <0.001, respectively, for significance of the differences observed in Zn^2+^ content between Zn-treated groups and untreated controls.

On the other hand, the absence of any significant changes in organ weights and LRWC clearly indicates that we succeeded in achieving moderate stress, as was anticipated. This conclusion was strongly supported by the data on Zn^2+^ tissue contents (Figure 2, Supplementary Information S2, Table S2(7)). Indeed, the obtained data clearly indicated that Zn was efficiently absorbed by treated plants and accumulated in roots approximately 28-fold compared to untreated controls (3531 vs. 125 µg/g DW). Zn content was also significantly increased in the mature and young leaves of the Zn-treated plants, although these changes were less pronounced in comparison to roots and were only 12- and 6-fold, respectively (168 vs. 14 and 479 vs. 81 µg/g, respectively). Thus, the patterns of the Zn-dependently up-regulated metabolites were in agreement with the observed stress response. The Zn-induced metabolic response of both leaves and roots was dominated by a pronounced increase in abundance of multiple features, which were well-represented in both organs. However, as can be seen from the corresponding heatmap (Figure 1a), the stress response in leaves was more diverse in terms of the number of affected metabolites. Nevertheless, although the root response to stress was less diverse, it was characterized by lower intra-group variability in comparison to leaves. This observation was confirmed by the principal component analysis (PCA) performed with the same dataset. Thus, as can be seen from the corresponding score plot built for the two first principal components (PC1 and PC2, Figure 1b), all experimental groups (both organs and treatments) were clearly separated with the percentage of explained variance for the constructed model 49.6 and 17.0% for PC1 and PC2, respectively. In agreement with the results of hierarchical clustering, all leaf groups showed higher dispersion in score plots and, therefore, lower confidence of stress-related alterations in the metabolome. In contrast, the stress-related metabolic shifts observed in roots were comparable with inter-organ differences.

### 2.3. Relative Quantification of the Zn-Responsive Primary Metabolites in the Leaves and Roots of A. caudatus

**Figure 3:**
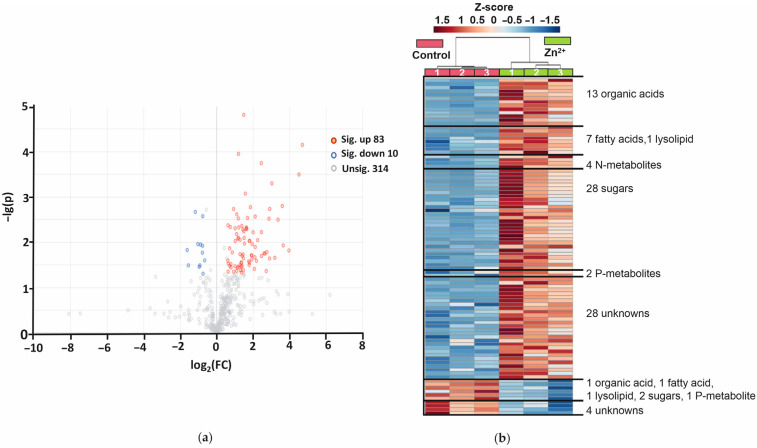
Metabolic responses of *A. caudatus* young leaves to a seven-day-long treatment with 300 μmol/L ZnSO_4_ (*n* = 3) in comparison to untreated controls (*n* = 3). (**a**) Volcano plot representing the numbers of the features differentially (≥1.5-fold, *p* ≤ 0.05) abundant in *A. caudatus* young leaves in comparison to young leaves of the control plants. Colored dot marks represent the features that demonstrated significantly higher (Sig. up, red) or lower (Sig. down, blue) abundance in Zn^2+^-treated young leaves in comparison to the controls; grey dots correspond to the features that did not show any significant alteration in young leaves in response to Zn^2+^-treatment. (**b**) Hierarchical clustering with heat map representation of 83 and 10 metabolites whose tissue contents were significantly (*p* ≤ 0.05) higher and lower, respectively, in samples of the Zn^2+^-treated plants. The metabolites were grouped according to their chemical classes. The list of Zn^2+^-regulated metabolites is presented in Table 1 and Supplementary Information S1, Table S1(2). The volcano plot and the heatmap were constructed in MetaboAnalyst 4.0 (https://www.metaboanalyst.ca, accessed on 16 March 2020).

**Figure 4:**
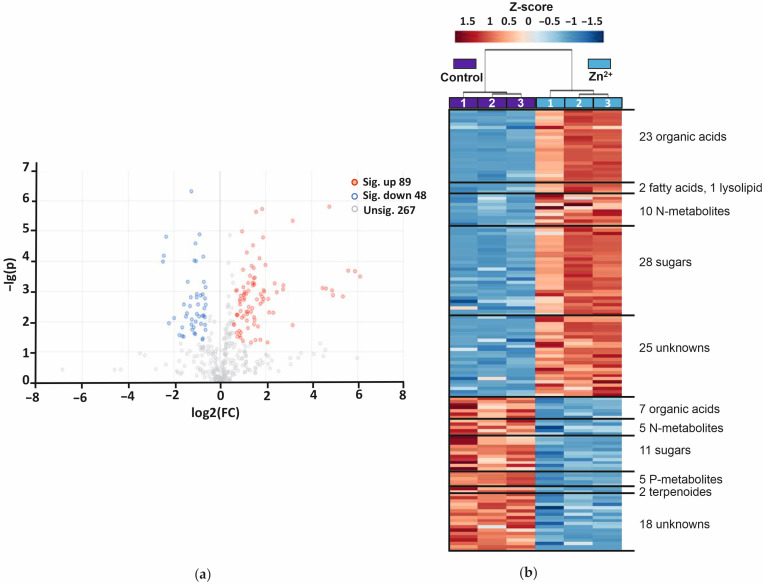
Metabolic responses of *A. caudatus* roots to a seven-day-long treatment with 300 μmol/L ZnSO_4_ (*n* = 3) in comparison to untreated controls (*n* = 3). (**a**) Volcano plot representing the numbers of features differentially (≥1.5-fold, *p* ≤ 0.05) abundant in *A. caudatus* roots in comparison to the roots of the control plants. Colored dot marks represent the features that demonstrated significantly higher (Sig. up, red) or lower (Sig. down, blue) abundances in Zn^2+^-treated roots in comparison to the controls; grey dots correspond to the features that did not show any significant regulation in roots in response to Zn^2+^-treatment. (**b**) Hierarchical clustering with heat map representation of the 89 and 48 metabolites whose tissue contents were significantly (*p* ≤ 0.05) higher and lower, respectively, in the samples of Zn^2+^-treated plants. The metabolites were grouped according to their chemical classes. The list of Zn^2+^-regulated metabolites is presented in Table 2 and Supplementary Information S1, Table S1(3). The volcano plot and the heatmap were constructed in MetaboAnalyst 4.0 (https://www.metaboanalyst.ca, accessed on 16 March 2020).

For further characterization of the metabolic rearrangements in *A. caudatus* plants in response to Zn^2+^ treatment, organ-specific paired comparisons were considered. Volcano plots and heatmaps were constructed for visualization of significantly affected metabolites in young leaves (Figure 3) and in roots (Figure 4). The volcano plots represent the features differentially (≥1.5-fold, *p* ≤ 0.05) abundant in Zn-treated *A. caudatus* young leaves and roots in comparison to the same organs in the control plants. Thereby, the confidence (*p*-value) and magnitude of the alterations (fold change, FC) are visualized.

In total, 93 metabolites were differentially abundant in young leaves of the Zn-stressed plants. Among them, 83 analytes showed higher and 10 lower abundance, compared to the control. Based on the heatmap representation, the Zn^2+^ -responsive metabolites could be grouped by their chemical classes (Figure 3b). The metabolites, which were more abundant in the young leaves, were represented by 13 organic acids, seven fatty acids, 28 sugars, four N-metabolites, two organic phosphates, one lysolipid and 28 unknown metabolites. Metabolites decreasing their content upon Zn treatment were represented with one organic acid, one fatty acid, two sugars, one organic phosphate, one lysolipid and four unknown compounds (see Table 1 and Supplementary Information S1, Table S1(2) for identified features and the total list of differentially abundant leaf metabolites).

**Table 1:** Zn^2+^-regulated structurally annotated metabolites in the young leaves of *A. caudatus*.

#	Metabolite Feature ^a^	Derivatives ^b^	RI ^c^	*m*/*z* ^d^	FC ^e^	*p* ^f^
Metabolites demonstrating higher abundances in Zn-treated leaves in comparison to untreated ones						
1	Glyoxylic acid	1MEOX, 1TMS	1142.6	218	22	0.015
2	**3-Hydroxy-3-methylglutaric acid**	3TMS	1598.9	247	3.6	0.002
3	**Gluconic acid δ-lactone**	4TMS	1878.5	319	2.8	0.008
4	Ribonic acid-1,4-lactone	3TMS	1645.9	117	6.2	0.018
5	Lyxonic acid-1,4-lactone	3TMS	1729.7	217	1.8	0.017
6	Arabinonic acid-1,4-lactone	3TMS	1622.2	217	2	0.005
7	**Gluconic acid**	6TMS	1990.7	333	10	0.003
8	**Shikimic acid**	4TMS	1808.5	204	2.5	0.041
9	**Salicylic acid**	2TMS	1504.2	267	23	≤0.001/0.026
10	Octanoic acid	1TMS	1269.1	117	>100	0.0003
11	Stearic acid	1TMS	2223.3	341	3	0.001
12	Arachidic acid	1TMS	2392.2	369	2.8	≤0.001/0.005
13	Myristic acid	1TMS	1846.1	285	2.6	0.02
14	Oleic acid	1TMS	2205.5	339	2	0.034
15	Heptadecanoic acid	1TMS	2134.3	327	2.1	0.002
16	**Behenic acid (docosanoic acid)**	1TMS	2547.0	397	2	0.009
17	1-Monostearateglycerol	2TMS	2651.2	399	2.2	0.036
18	**Pyroglutamic acid (5-oxoproline)**	1TMS	1496.4	84	5.5	0.006
19	**Ethanolamine**	3TMS	1232.1	174	4.2	0.038
20	***N*-acetyl-serine**	2TMS	1503.1	116	12	0.002
21	**5-Methylcytosine**	2TMS	1534.0	254	7.4	0.023
22	**Fructofuranose, peak 1**	5TMS	1807.0	217	4.3	0.013
23	**Fructose, peak 2**	1MEOX, 5TMS	1942.9	217	4.3	0.021
24	Fructofuranose, peak 2	5TMS	1798.7	217	3.9	0.011
25	**Fructose, peak 1**	1MEOX, 5TMS	1934.8	217	3.8	0.024
26	**Glucose, peak 1**	1MEOX, 5TMS	1990.5	319	3.7	0.019
27	**Glucose, peak 2**	1MEOX, 5TMS	2007.2	319	2.6	0.033
28	**Mannose**	1MEOX, 5TMS	1984.3	319	3.5	0.028
29	**Galactose**	1MEOX, 5TMS	1979.6	319	2.1	0.042
30	**Myo-inositol**	6TMS	2076.9	305	1.9	0.044
31	Sucrose	8TMS	2540.8	361	6.6	0.043
32	Phosphoric acid monomethyl ester	2TMS	1185.5	241	1.7	0.036
Metabolites demonstrating lower abundances in Zn-treated leaves in comparison to untreated ones						
1	**Succinic acid**	2TMS	1316.6	247	2.0	0.012
2	Linoleic acid	1TMS	2192.7	337	1.7	0.003
3	2-Oleoylglycerol	2TMS	2629.7	129	1.7	0.012
4	**Glycerol-3-phosphate**	4TMS	1758.8	299	1.7	0.017

^a^ Structurally annotated metabolites arranged by the following chemical classes: organic acids, fatty acids, lysolipids, N-metabolites, monosaccharides, polyols, disaccharides, organic phosphates. Numbers 1 or 2 next to the metabolite features with the same annotation indicate the peak (isomer) number of the metabolite. ^b^ The numbers of trimethylsilyl (TMS) and methyloxime (MEOX) groups. ^c^ RI, Kovach retention index. ^d^ The *m*/*z* value refers to the fragment selected as compound-characteristic for quantification by integration of peak areas at characteristic extracted ion chromatograms. ^e^ FC, fold change (at least 1.5-fold) in metabolite relative abundances in Zn-treated young leaves compared to controls. ^f^ *t*-test *p*-value ≤ 0.05. The symbol “/” indicates the *p*-values calculated without and with false discovery rate (FDR) correction by the Benjamini–Hochberg method and did not exceed the *p*-value threshold of 0.05 after application of the FDR correction. The total list of Zn-regulated differentially abundant metabolites, which includes structurally annotated metabolite features (36 species), presented in the table, the unknown features (32 species) and features (25 species) annotated only to a specific chemical class, is presented in Supplementary Information S1, Table S1(2). Bold fond marks metabolites which were also Zn-regulated in roots.

The total number of root metabolites, which were differentially (≥1.5-fold, *p* ≤ 0.05) abundant in Zn^2+^-treated plants in comparison to the control ones, was 137, i.e., 47% higher than in leaves. Among them, 89 metabolites were more abundant and 48 less abundant (Figure 4a). As can be seen in Figure 4a and Supplementary Information S1, Table S1(3), differential abundance of multiple metabolites could be confirmed with higher confidence in roots than in leaves (*p* ≤ 0.001 vs. *p* ≤ 0.05). Also, in contrast to the leaf data, the lowest *p*-values, observed in the up- and down-regulated metabolite groups were comparable for roots. The group of up-regulated root metabolites contained more organic acids (23), carbohydrates (28) and N-metabolites (10), but fewer fatty acids (2) compared to young leaves (Figure 3b and Figure 4b). Also, no phosphates could be identified among up-regulated root metabolites (see Table 2 and Table S1(3) for identified features and the total list of differentially abundant root metabolites). Overall, sugars and organic acids were the most representative metabolite classes in both young leaves and roots of the Zn-stressed amaranth plants.

**Table 2:** Zn^2+^-regulated structurally annotated metabolites in the roots of *A. caudatus*.

#	Metabolite Features ^a^	Derivatives ^b^	RI ^c^	*m*/*z* ^d^	FC ^e^	*p* ^f^
Metabolites demonstrating higher abundances in Zn-treated roots in comparison to untreated ones						
1	Malonic acid	2TMS	1211.3	233	2.1	0.002
2	Glyceric acid	3TMS	1330.4	292	2.5	≤0.001/0.004
3	Citric acid	4TMS	1814.1	273	2.7	0.0035/0.01
4	Citramalic acid	3TMS	1468.2	247	1.8	0.002/0.01
5	3,4-Dihydroxybutyric acid γ-lactone	-	1371.4	247	3.7	≤0.001/0.007
6	Adipic acid	2TMS	1504.9	111	1.9	0.023
7	Threonic acid	4TMS	1558.5	292	3.8	≤0.001/0.007
8	Erythronic acid	4TMS	1540.9	292	2.0	≤0.001/0.003
9	α-Hydroxyglutaric acid	3TMS	1573.8	247	2.4	0.003/0.014
10	**3-Hydroxy-3-methylglutaric acid**	3TMS	1598.9	247	2.4	≤0.001/0.007
11	**Gluconic acid**	6TMS	1990.7	333	59	≤0.001/0.003
12	**Gluconic acid δ-lactone**	4TMS	1878.5	319	25	≤0.001/0.007
13	Xylonic acid-1,4-lactone	3TMS	1629.5	117	2.8	≤0.001/0.005
14	**Shikimic acid**	4TMS	1808.5	204	2.7	≤0.001/0.005
15	**Salicylic acid**	2TMS	1504.2	267	27	≤0.001/≤ 0.001
16	2-Hydroxysebacic acid	2TMS	2525.2	317	5.2	≤0.001/0.005
17	**Behenic acid**	1TMS	2547.0	397	1.7	0.006/0.02
18	1-Monooleoylglycerol	2TMS	2632.9	129	1.7	0.006/0.02
19	Oleic acid amide	1TMS	2366.7	338	9.6	0.013
20	Proline [+CO_2_]	2TMS	1577.6	142	6.8	≤0.001
21	Alanine	3TMS	1357.0	188	2.9	0.04
22	Leucine	2TMS	1271.7	158	2.0	0.042
23	*N*,*N*-Dimethylglycine	1TMS	1040.4	58	1.9	0.004/0.02
24	**N-acetyl-serine**	2TMS	1503.1	116	1.5	0.012/0.035
25	**5-Methylcytosine**	2TMS	1534.0	254	3.7	≤0.001/≤0.001
26	Uridine	3TMS	2389.4	217	2.3	0.005/0.02
27	Adenine—derivative	2TMS	2096.2	264	1.8	0.0018
28	Arabinose 1	1MEOX, 4TMS	1755.0	307	2.3	≤0.001
29	Arabinose 2	1MEOX, 4TMS	1758.9	307	2.2	0.005/0.02
30	Arabino-hexos-2-ulose (2-ketoglucose)	4TMS	1477.2	234	2.3	0.02/0.044
31	**Fructofuranose**	5TMS	1807.0	217	2.8	≤0.001/0.005
32	**Fructose, peak 1**	1MEOX, 5TMS	1934.8	217	2.8	≤0.001/0.004
33	**Fructose, peak 2**	1MEOX, 5TMS	1942.9	217	2.7	≤0.001/0.006
34	**Glucose, peak 1**	1MEOX, 5TMS	1990.5	319	4.0	≤0.001/0.002
35	**Glucose, peak 2**	1MEOX, 5TMS	2007.2	319	2.0	≤0.001/0.006
36	**Mannose**	1MEOX, 5TMS	1984.3	319	3.0	≤0.001/≤0.001
37	**Galactose**	1MEOX, 5TMS	1979.6	319	9.0	≤0.001/≤0.001
38	**Myo-inositol**	6TMS	1979.6	319	2.0	0.001/0.008
39	Ribitol	5TMS	1720.7	217	2.8	≤0.001/0.003
40	2-*O*-Glycerol-α-*D*-galactopyranoside	6TMS	2283.0	204	2.2	0.001/0.008
41	Trehalose	8TMS	2675.5	361	2.7	≤0.001/≤0.001
Metabolites demonstrating lower abundances in Zn-treated roots in comparison to untreated ones						
1	Oxalic acid	2TMS	1145.5	190	1.6	0.004/0.015
2	**Succinic acid**	2TMS	1316.6	247	1.5	≤0.001/0.006
3	Methylmaleic acid	2TMS	1348.9	259	2.3	0.02/0.05
4	*trans*-Caffeic acid	3TMS	2137.6	219	4.0	0.007/0.02
5	Itaconic acid	2TMS	1342.9	183	2.4	0.012/0.035
6	**Ethanolamine**	3TMS	1232.1	174	2.8	0.007/0.023
7	**Pyroglutamic acid**	1TMS	1496.4	84	2.2	0.024
8	Oleic acid amide	-	2323.2	59	1.5	0.02/0.048
9	**Glycerol-3-phosphate**	4TMS	1758.8	299	2.2	≤0.001/0.002
10	Glycerophosphoglycerol	5TMS	2181.2	357	5.1	≤0.001/≤0.001
11	*myo*-Inositol phosphate	7TMS	2361.7	318	1.8	0.001/0.008
12	Phytol	1TMS	2159.7	143	2.3	0.008/0.03
13	β-Sitosterol	1TMS	3087.5	486	3.0	0.003/0.012

^a^ Structurally annotated metabolites arranged by the following chemical classes: organic acids, fatty acids, lysolipids, N-metabolites, monosaccharides, polyols, disaccharides, and organic phosphates. Numbers 1 or 2 next to the metabolite features with the same annotation indicate the peak (isomer) number of the metabolite. ^b^ The numbers of trimethylsilyl (TMS) and methyloxime (MEOX) groups. ^c^ RI, Kovach retention index. ^d^ The *m*/*z* value refers to the fragment selected as compound-characteristic for quantification by integration of peak areas at characteristic extracted ion chromatograms. ^e^ FC, fold change (at least 1.5-fold) in metabolite relative abundances in Zn-treated roots compared to controls. ^f^ *t*-test *p*-value ≤ 0.05. The symbol “/” indicates the *p*-values calculated without and with false discovery rate (FDR) correction by the Benjamini–Hochberg method and did not exceed the *p*-value threshold of 0.05 after application of the FDR correction. Bold fond marks metabolites which were also Zn-regulated in young leaves.

As can be seen from Figure 3a, most of the differentially abundant metabolites in young leaves were up-regulated upon Zn treatment. Octanoic acid was the most affected compound among them (FC > 100, Table 1). The others were glyoxylic (FC 22), salicylic (FC 23) and gluconic (FC 10) acids. The other sugar acids (besides gluconic) and their lactons demonstrated a lower abundance increase (1.8–6.2-fold) in response to Zn^2+^ treatment, whereas disaccharides were 5.4–9.1-fold up-regulated. Zn^2+^-related accumulation of monosaccharides, in particular galactose, glucose, mannose and fructose, was less pronounced and did not exceed 2.1–4.3-fold. Essential up-regulation was observed also for four N-metabolites—*N*-acetyl-serine (12.1-fold), 5-methylcytosine (7.4-fold), pyroglutamic acid (5.5-fold) and ethanolamine (4.2-fold). In contrast, only a few metabolites decreased their content in amaranth tissues upon Zn^2+^ treatment: succinic acid (2.0-fold), linoleic acid, 2-oleoylglycerol and glycerol-3-phosphate (each 1.7-fold, Table 1).

As can be seen from Figure 4a, the Zn^2+^ response in roots of *A. caudatus* was more balanced, although still dominated by up-regulation. The most pronounced abundance increase was observed for several sugar acids, especially for non-identified sugar acid RI1988 (69-fold), gluconic acid (59-fold) and gluconic acid-δ-lacton (25-fold) (Table S1(3) and Table 2). A strong up-regulation (27-fold) was also observed for salicylic acid. Thus, the Zn-induced increase in content of the above-mentioned compounds in roots was higher than in leaves. This fact might indicate stronger involvement of roots in the metabolic response to Zn^2+^ treatment in our study. For other (not sugar-related) organic acids (malonic, glyceric, citric and threonic), much less pronounced up-regulation (2.0–3.8-fold) was observed. Also, a Zn-induced accumulation was observed for disaccharides (1.8–3.7-fold), including trehalose (2.7-fold), as well as for monosaccharides (FC 1.8–4.0), such as arabinose, fructose, glucose and mannose, with only galactose being much strongly up-regulated (9.0-fold). The content of several fatty acids, as well as amino acids (proline, alanine, leucine) and some other N-metabolites, also increased in roots of Zn-treated plants, with oleic acid amide (9.6-fold) and proline (6.8-fold) identified as the most responsive compounds in these classes.

The list of structurally annotated down-regulated root metabolites included 13 compounds (Table 2). The Zn-related decrease in abundance could be confirmed for five organic acids (oxalic, succinic, itaconic, methylmaleic and *trans*-caffeic), two N-metabolites, three P-metabolites and two terpenoids. The FC values for most of the compounds were in the range of 1.5–3.0, although two metabolites, namely *trans*-caffeic acid and glycerophosphoglycerol, were more strongly affected and demonstrated 4.0–5.1-fold down-regulation. Among the other 35 structurally unannotated features decreasing their relative abundances, most were represented with unknowns (18 species). The second most abundant compound class was that of carbohydrates, with eleven species demonstrating 1.6–4.7-fold down-regulation in roots of Zn-treated plants (Figure 4b and Table S1(3)).

To address the discrepancies and similarities in the Zn-stress responses of young leaves and roots, we compared the lists of significantly (*p* ≤ 0.05) Zn-dependently regulated leaf and root metabolites, presented in Table 1 (Table S1(2)) and Table 2 (Table S1(3)). The comparison revealed 48 common features, most of which showed similar abundance dynamics in response to stress: 35 and 4 features, occurring both in roots and leaves, demonstrated concerted increase and decrease, respectively. Only nine metabolites showed different stress-related dynamics in the two compared plant organs, and among them, only two features (ethanolamine and pyroglutamic acid) were structurally annotated.

Among the features showing concerted stress-induced up-regulation, the following metabolites were structurally annotated: sugars and sugar-related substances (glucose, fructose, mannose, galactose, *myo*-inositol, gluconic acid), salicylic acid, shikimic acid, 3-hydroxy-3-methylglutaric acid, behenic acid, *N*-acetyl-serine and 5-methylcytosine. Some metabolites (salicylic acid, gluconic acid and its lactone, galactose) showed stronger Zn-dependent regulation in roots in comparison to leaves. Among the metabolites showing concerted Zn-induced abundance decrease in roots and leaves, succinic acid and glycerol-3-phosphate were structurally annotated.

To address the dynamics of Zn-dependently regulated metabolites in *A. caudatus* young leaves and roots, in more detail, all detected features can be classified in the following principal groups: (i) structurally annotated (36 and 54 features in young leaves and roots, respectively, Table 1 and Table 2), (ii) annotated only to a specific compound class without further structure annotation (25 and 40 features, respectively, Supplementary Information S1, Table S1(2,3)) and (iii) unknowns (32 and 43 features, respectively, Table S1(2,3)). To reveal the metabolic pathways involved in the process of metabolic adjustment under Zn-stress (see below), the features assigned to the first group were further subjected to pathway analysis. The features representing the groups i and ii are discussed in more detail in the following sections.

### 2.4. Structure Elucidation of Di- and Oligosaccharides Based on RI and EI Mass Spectra

As di- and oligosaccharides represented one of the most strongly dominating chemical groups among the detected Zn-responsive metabolites of amaranth roots and leaves (16 in roots, 12 and 11 in young and mature leaves, respectively, Supplementary Information S1, Table S1(2–4)), the correctness of their annotation became a critically important aspect. Therefore, here we describe our annotation algorithm, which was applied to such compounds throughout the whole work. In the following paragraphs, we describe this universal logic in detail and exemplify it with a typical representative analyte—RI2864 di- or oligosaccharide (Table S1(3)), which demonstrated a 6.8-fold abundance increase in Zn-treated roots compared to control.

This metabolite was annotated as a di-/oligosaccharide by the similarity of its analytical behavior to other compounds of this class. The annotation was based on the following two criteria. The first is the similarity of EI mass spectra to those typical for disaccharides and trisaccharides (as can be illustrated by the EI-MS spectra from our in-house library, Supplementary Information S1, Figure S1(6)). The second is the elution within the characteristic RI window close to sucrose (Table S1(1), in-house library RI2541), which is considered to be a representative disaccharide and to maltotriose (GDM RI2887)—a typical trisaccharide. It is known that EI spectra of di- and oligosaccharides (MEOX/TMS derivatives of di- and oligosaccharides) demonstrate multiple common fragments with the spectra of MEOX/TMS derivatives of monosaccharides. However, they are featured with several characteristic signals which might serve as indicators of the glycosidic linkages between the monosaccharide residues. The cleavage of the glycosidic linkage of MEOX/TMS derivatives of the reducing disaccharide yields two characteristic structural moieties: a ring structure and an open chain moiety (a MEOX/TMS derivative). Under the EI conditions, both yield characteristic fragments with their signals clearly seen in the spectra [45].

**Figure 5:**
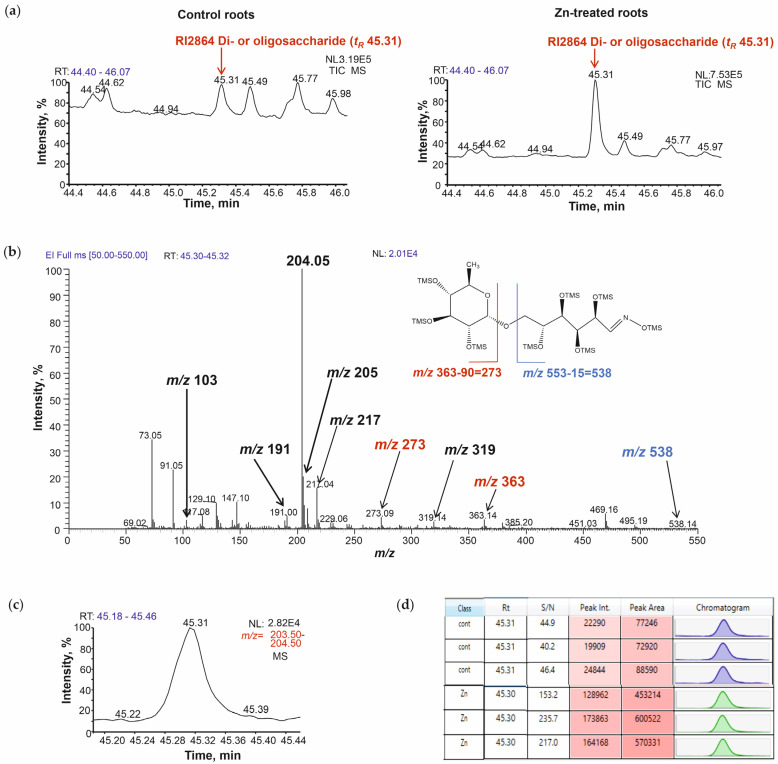
GC-MS information supporting annotation of a metabolite RI 2864 as an oligosaccharide, which showed a 6.8-fold abundance increase in Zn-treated *A. caudatus* roots as compared with the control. The analyte was eluted at the retention time (*t_R_*) of 45.31 min (RI 2864) and was annotated to the class of di-/oligosaccharide with its preliminary identification as rutinose. (**a**) Total ion chromatograms (TICs) of control and Zn-treated *A. caudatus* root extracts presented for the *t_R_* window of 44.4–46.1 min in which a peak of the RI2864 Di-/oligosaccharide located; (**b**) EI mass spectra of the metabolite (the fragments marked with red and blue arrows refer to the moieties of ring and open chain structure, respectively, those marked with black bold font presented fragments diagnostic for carbohydrates and showed in Supplementary Information S1, Table S1(5)); (**c**) Extracted ion chromatogram (XIC) for *m*/*z* 204 ± 0.5 (base peak in the EI spectrum) and *t_R_* 45.31 of the RI2864 Di-/oligosaccharide; (**d**) A screenshot from MSDial program presented the RI2864 Di-/oligosaccharide XIC peak area integration values for three control and three Zn-treated samples.

As can be seen in Figure 5, the peak of RI2864 at *t_R_* 45.31 showed much higher relative intensity in the total ion chromatogram (TIC) of the stressed root samples in comparison to the control ones (Figure 5a). The corresponding EI-MS spectrum (Figure 5b) delivered a rich pattern of signals. The base peak at *m*/*z* 204 appeared to be applicable for relative quantification by its extracted ion chromatogram (XIC) at *m*/*z* 204.0 ± 0.5 and *t_R_* 45.31 (Figure 5c), i.e., integration of the area under the curve (Figure 5d). The assignment of RI2864 as a disaccharide relied on the presence of two characteristic fragments in the spectrum: *m*/*z* 363 corresponding to the pyranosyl moiety and *m*/*z* 273 corresponding to a TMS loss from it (marked red, Figure 5b). These fragments indicate the presence of rhamnopyranosyl moiety in the structure of the di- or oligosaccharide [45,46]. The open chain moiety (containing a methyl oxime group) is characterized by the fragment ion (of trace abundance) at *m*/*z* 538 (marked blue in Figure 5b). This fragment indicates that RI2864 might be a reducing sugar. The third group of signals (marked with black bold) at *m*/*z* 191, 204, 205, 217 and 319 represents the common characteristic fragments for carbohydrates [44]. Their structures are presented in Supplementary Information S1, Table S1(5). Thus, according to the EI spectrum and the literature data on the amaranth metabolism [47], we may conclude that RI2864 di- or oligosaccharide most likely corresponds to rutinose. This assignment was additionally supported by the absence of the fragment at *m*/*z* 361 and low intensity of the fragment signal at *m*/*z* 319: these fragments are characteristic of non-reducing disaccharides and are absent in reducing disaccharides like rutinose [46]. Moreover, the intensity ratio of the fragments at *m*/*z* 217 and *m*/*z* 204 was below one, which indicated the existence of a 1 → 6 glycosidic linkage in the disaccharide molecule, which is the case for rutinose. However, as the authentic standard of rutinose was not available, the compound assignment was treated as “putative identification”. Annotation of two other di-/oligosaccharides is comprehensively described in Supplementary Information S1, Protocol S1-1 and illustrated in Figure S1(7,8).

### 2.5. Absolute Quantification of the Zn-Responsive Primary Metabolites in the Leaves and Roots of A. caudatus in Targeted GC-MS Experiments

To address the quantitative contribution of individual metabolites in the observed Zn-induced metabolic shifts, the absolute tissue contents of 29 principal metabolites representing several compound classes (carboxylic acids, amino acids, polyols, di- and monosaccharides) were addressed in the young leaves and roots of control and Zn-treated *A. caudatus* plants. For the targeted analysis, we decided on the external calibration method. Although this method is less expensive than the stable isotope dilution approach, it is material-, time- and work-consuming. Therefore, we decided to focus only on the metabolites that were earlier reported to be important in the mechanisms of HM tolerance in plants [9,29,31] and selected only 29 individual compounds for targeted analysis. Among these, 21 compounds (Table 3) were confirmed in young leaves and roots of control and Zn-treated *A. caudatus* plants. Additionally, it could be seen that most of the dissolved Glu standard formed pyroglutamate in solution. Therefore, pyroglutamate was used for the quantitation of Glu in plant samples.

The results of the absolute quantification survey conducted with external standardization are summarized in Table 3. As can be seen in the table, sucrose, as well as malic, benzoic, oxalic and pyroglutamic acids appeared to be the most abundant among the root and leaf primary metabolites selected for targeted analysis. Thus, their content in roots of untreated plants accounted for 7.6, 4.7, 9.1, 10.7 and 19.0 µmol/g DW, respectively. The levels of these metabolites in leaves of the untreated plants were 2–3 times lower. However, alanine showed approximately six-fold higher abundance in leaves compared to roots (7.9 vs. 1.4 µmol/g DW). Interestingly, the tissue content of the most abundant metabolites (malic, benzoic, oxalic and pyroglutamic acids and alanine) was only slightly and mostly insignificantly affected by Zn-induced stress. Indeed, more than a twofold accumulation could be observed only for pyroglutamate and alanine in young leaves and roots, respectively. The other analyzed metabolites were mostly up-regulated in the roots of Zn-treated plants, whereas this up-regulation was less pronounced in the leaves (Table 3). For several metabolites, the magnitude of the stress-induced responses was strikingly different between the plant organs. E.g., the relatively high content (7.6 µmol/g DW) of sucrose in roots was not changed upon the application of Zn^2+^ stress, whereas a relatively low amount of this sugar present in leaves (2.2 µmol/g DW) increased 7.6-fold under stress conditions.

Galactose, glucose and *myo*-inositol demonstrated a concerted increase in their content in both organs, with the most striking stress-related difference observed for galactose (7.8-fold in roots). The most abundant representatives of organic acids in both young leaves and roots were benzoic, oxalic, malic, and succinic acids. Among them, only succinate demonstrated concerted dynamics of Zn^2+^ stress-related response, i.e., a 1.9- and 1.6-fold decrease in its abundance in young leaves and roots, respectively (*p* ≤ 0.01, Table 3). Benzoic acid showed a slight (approximately 30%) abundance decrease in leaves and oxalic acid—a 50% decrease in roots of Zn-treated plants, whereas the content of malic acid demonstrated no significant changes in response to Zn^2+^ stress in both organs.

### 2.6. Annotation of Zn-Responsive Metabolic Pathways in A. caudatus Leaves and Roots

**Figure 6:**
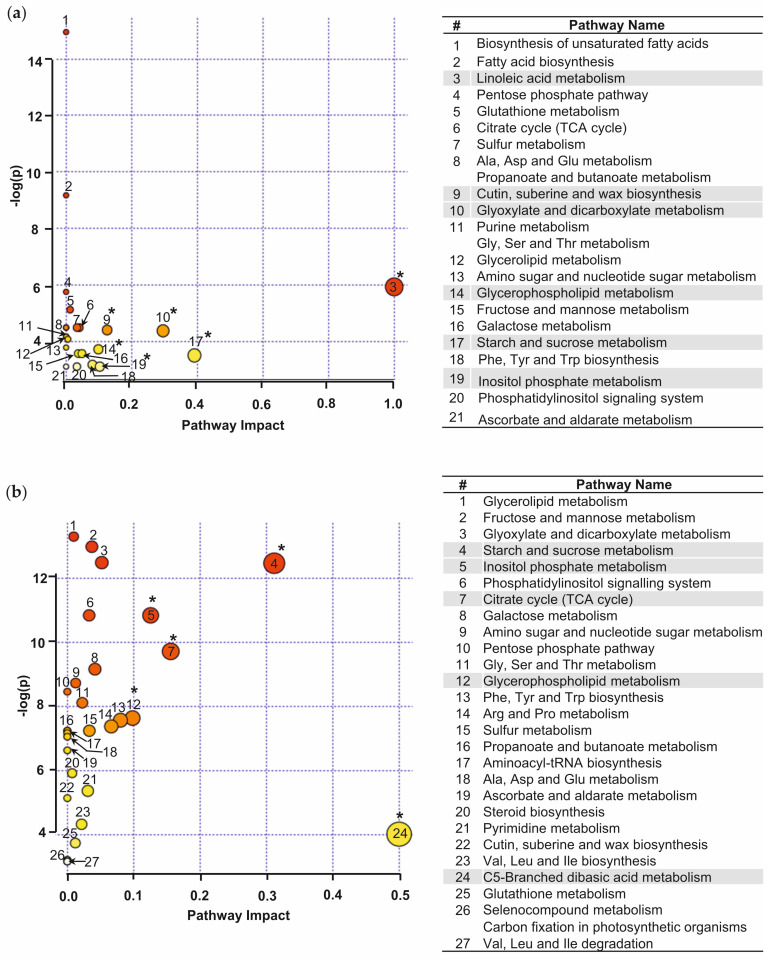
Pathway analysis of the Zn-regulated (≥1.5-fold, *t*-test *p* ≤ 0.05) metabolites of *A. caudatus* young leaves (**a**) and roots (**b**) annotated by untargeted GC-EI-Q-MS. The pathway analysis relied on the *Arabidopsis thaliana* pathway library (deposited on the KEGG on-line platform from 03.2020) and presents the results from combined pathway enrichment analysis (global test) and pathway topology analysis (relative-betweenness centrality) to highlight the most confident pathways related to the Zn^2+^-stress condition. Indicators such as higher circle position along the *Y*-axis and more intense red color of the circles indicate higher significance of the observed differences, whereas the other two indicators, such as circle size and position along the *X*-axis (pathway impact), indicate the impact of the annotated metabolites in the corresponding pathways. The pathways (marked numerically) listed in the corresponding tables on the right panels are ranked according to the results of pathway enrichment analysis. The symbol * marks pathways (their names are indicated by grey font) with their pathway impact value ≥0.1, which are considered as potential main contributors to metabolic response to Zn-exposure. More details for the pathway analysis are provided in Supplementary Information S1, Table S1(6,7) for young leaves and roots, respectively. To address the regulated metabolic pathways separately for the Zn-dependent high- and low-abundant (≥1.5-fold, *t*-test *p* ≤ 0.05) metabolites, please refer to the Supplementary Information S3, Parts S1 and S2. The Pathway analyses were performed in MetaboAnalyst 4.0 (https://www.metaboanalyst.ca, accessed on 19 March 2020) and details on the analyses are provided in Supplementary Information S4.

To address the role of individual metabolic pathways in the observed Zn-related responses, the data on stress-dependently regulated (≥1.5-fold, *t*-test *p* ≤ 0.05) primary metabolites annotated in *A. caudatus* young leaves (36 features, Table 1) and roots (54 features, Table 2) were subjected to the pathway analysis. This analysis relied on the *Arabidopsis thaliana* pathway library (deposited by KEGG on-line platform from 03.2020). The results of the combined pathway enrichment analysis (global test) and pathway topology analysis (relative-betweenness centrality test) allowed identification of the most confident pathways strongly contributing in the Zn-related metabolic responses in the young leaves (Figure 6a, Supplementary Information S1, Table S1(6) and Supplementary Information S3, Part S1) and in roots (Figure 6b, Supplementary Information S1, Table S1(7), Supplementary Information S3, Part S2) of *A. caudatus*.

As can be seen from the results, the number of pathways involved in Zn-induced metabolic responses in roots was higher than in leaves (27 vs. 21, Supplementary Information S1, Table S1(6,7)). Moreover, based on the comparison of the false discovery rate (FDR)-adjusted (Holm–Bonferroni method) *p*-values, annotation of the root Zn-inducible pathways was more confident. It might be a direct consequence of the higher confidence of the stress-induced changes observed in roots (Figure 6b). However, most of the pathways for both organs were represented with only one hit (15 and 16 for leaves and roots, respectively, Table S1(6,7)). Galactose and fatty acid metabolism were featured with the highest numbers of confidently matched leaf metabolites, whereas galactose and glyoxylate/dicarboxylate metabolism were confirmed as the most represented in roots. Finally, the highest impact values (≥0.1) were calculated for linoleic acid, starch and sucrose, glyoxylate and dicarboxylate metabolism, cutin, suberine and wax biosynthesis, glycerophospholipid metabolism and inositol phosphate metabolism in leaves, and for C5-branched dibasic acid metabolism, TCA cycle, starch and sucrose, glycerophospholipid and inositol phosphate metabolism in roots. Thus, the pathways which contributed most to the Zn-induced response in *A. caudatus* leaves and roots were represented mainly with sugars, sugar- and TCA-related acids and fatty acids, while the pathways of nitrogen metabolism were less involved and featured with low (<0.1) pathway impact values (Supplementary Information S1, Table S1(6,7)).

## 3. Discussion

### 3.1. Dynamics of the Metabolite Patterns in the Integrated Plant Response to Zn Stress

Metabolomics is an efficient tool in the studies of the plant responses to abiotic stress; in particular, for revealing the stress-induced metabolic rearrangements and adjustments in the plant metabolic network [48]. Although the untargeted metabolomics approach is widely used in the analysis of osmotic and drought stress response [10], such studies in the field of HM stress are still rare and focus mainly on the effects of Cd and Pb [15,19]. Meanwhile, contamination of agricultural soils with Zn^2+^ ions becomes an important environmental problem [25], and the metabolic shifts associated with Zn^2+^ toxicity require proper characterization. In the first step, it seems logical to characterize the stress-related metabolite patterns in a “static” mode using one time point and the conditions referred to in literature as Zn-stress. Therefore, here, for the first time, we employed the state-of-the-art GC-EI-Q-MS technique to characterize the metabolic responses in the roots and leaves of seven-week-old *A. caudatus* plants to a seven-day-long exposure to 300 µmol/L ZnSO_4_·7H_2_O in the nutrient solution. The choice of the current Zn concentration relied on the available literature data [24,25,49], including those for amaranth [35], which indicated that in various plants, Zn^2+^ concentrations in the range of 50–500 µmol/L triggered clear manifestations of a moderate metal stress accompanied by a well-defined adaptive response.

We showed that exposure to Zn^2+^ ions caused considerable changes in the metabolite profiles of different organs of *A. caudatus*. Indeed, 33 and 22% of the total number of MEOX/TMS derivatives detected in roots and young leaves, respectively, were differentially abundant in this experiment (Figure 1, Supplementary Information S1, Table S1(1)). Interestingly, the additional analyses performed with mature leaves revealed a lower number of differentially abundant metabolites, accounting for only 12% of the total list of detected compounds (Figure S1(9) and Table S1(4)).

The chemical patterns of the Zn^2+^ stress responses in young and mature leaves and roots were dominated by the up-regulated metabolites. This trend was more pronounced in leaves, regardless of their age, than in roots (Figure 3b, Supplementary Information S1, Figure S1(9) and Table S1(2–4)). Considering the metabolite dynamics in all three organs (roots and leaves of different ages) might provide access to the spatial dimension of metabolomics, i.e., to the distribution of metabolites at the level of the whole plant to ensure Zn^2+^ stress tolerance in *A. caudatus*.

The pathway analysis, accomplished for the differentially abundant leaf and root metabolites with unambiguous structural annotation, allowed revealing the metabolic processes involved in the Zn-induced responses. The pathways with the highest impact were mostly related to sugar, organic acid and lipid metabolism, whereas the metabolism of nitrogen-containing compounds was less affected (Table S1(6–8)). Although many of the revealed pathways were represented by only one hit, the pathway analysis procedure ensures the reliability of such results. Hits are based on the *p*-values calculated from the enrichment analysis, and the pathway impact (PI) values directly depend on the *t*-test *p*-values of the hit metabolites and their location/properties of the corresponding enzyme reactions in the pathways. Both these parameters rely on *t*-statistics. Thus, if a particular enzyme regulates the whole pathway, then the corresponding metabolite up- or downstream of that enzyme would define the impact of the whole pathway. Therefore, the lower pathway *p*-value and higher pathway impact value indicate that this pathway may have a higher contribution in the whole observed plant response.

Some of the hits obtained from the pathway analysis are common for several related pathways (Table S1(6–8)), implying cross-talk between different metabolic routes. The fact that the same compounds may be involved in different biochemical processes reflects the high complexity of the plant metabolome network. However, these metabolites may have different PI values in different pathways, and the pathway where the compound has the highest PI is expected to contribute most to the plant response to Zn-stress; e.g., among all confident pathways, the “starch and sucrose metabolism” demonstrated the highest impact for young leaves, roots, and mature leaves of *A. caudatus*.

Notably, the metabolites annotated as sugars and demonstrating higher abundance in Zn-treated plants compared to the control ones were much more diverse in young leaves and roots than in mature leaves. By contrast, the number of Zn-dependently accumulated organic acids was twice as high in roots as in leaves of any age (Figure 3b and Figure 4b and Supplementary Information S1, Figure S1(9B)). This is in agreement with the results of the pathway analysis (especially those based on the root data), which indicated sugar- and organic acid-related pathways as the potential main contributors in the Zn-related stress response (Figure 6, Supplementary Information S1, Table S1(6–8) and Figure S1(10), Supplementary Information S3). Induction of organic acids and soluble sugars as “stress metabolites” is a general feature common for plant responses to different stresses [50], including Cd^2+^ stress [15,16,17]. However, in relation to Zn^2+^ stress, this aspect is still insufficiently addressed.

Therefore, based on the number of differentially abundant metabolites, their FC-values, and the data of the pathway enrichment analysis, we may conclude that up-regulation of sugars and organic acids is a key feature in the metabolic signature of the amaranth response to the Zn^2+^ stress.

### 3.2. Di- and Monosaccharides in the Response of Amaranth Plants to Zn Stress

The involvement of sugars in stress responses is attributed mainly to their role as osmolytes and osmoprotectants in maintaining cell turgor and stabilizing membranes of the plant cell [51]. We suggest that this was also the case in our study, as the trend towards a decrease in fresh (but not dry) biomass gain was shown for Zn-exposed plants (Supplementary Information S1, Figure S1(5)). Sugars are also known as ROS scavengers [50,51,52]. Moreover, due to their higher ROS reactivity and lower amenability to hydroxyl radical-related damage, disaccharides and sugar alcohols are recognized as more efficient antioxidants than monosaccharides [53,54]. In addition, sugars may contribute sufficiently to maintaining the redox environment in the cell [55]. Improvement of their availability under stress conditions may stimulate the pentose phosphate pathway (PPP) reactions and thus boost NADPH biosynthesis. Enhanced generation of NADPH was shown to dramatically increase the ROS scavenging capacity in plant cells [56].

The intracellular accumulation of Zn^2+^ ions might cause redox imbalance and overproduction of ROS due to interaction with redox proteins and disturbance of electron transport mechanisms [4,25]. This is recognized as a trigger for intracellular accumulation of soluble sugars (especially oligosaccharides) and their engagement in ROS scavenging [25]. Indeed, we observed up to nine- and seven-fold up-regulation of oligosaccharides in Zn-stressed *A. caudatus* young leaves and roots, respectively (Table S1(2,3)). This was accompanied by the sugar up-regulation up to five-fold in mature leaves (Table S1(4)). Soluble sugars may be formed as a result of stress-induced degradation of storage polysaccharides and/or enhancement of gluconeogenesis and the glyoxylate cycle [57]. However, among the annotated disaccharides increasing their abundance upon the Zn treatment, only sucrose in young and mature leaves and trehalose in roots were unambiguously identified by co-elution with authentic standards, whereas no exact structures could be annotated to the remaining unknown di- and oligosaccharides (Table S1(2–4)). Currently, a stress-induced increase in sucrose accumulation is considered to be associated with the activation of the enzymes involved in sugar metabolism, as well as up-regulation of sucrose transporters [58,59]. Our results revealed a more pronounced increase in sucrose content in the young leaves of treated plants compared to mature leaves (Table 3, Supplementary Information S1, Table S1(9)). Such an imbalance is unlikely to be caused solely by greater enhancement of in situ sucrose production in young leaves, which are typically regarded as mostly heterotrophic sinks dependent on phloem sugar supply [60,61]. Therefore, enhancement in sucrose translocation from mature (source) to young (sink) leaves should be considered as the second possible mechanism. A similar stress-induced redistribution of carbohydrates was previously shown for arabidopsis leaves [62]. At the systemic level, these results may indicate the stress-induced enhancement of metabolite allocation to the sinks as an adaptive response [62]. This phenomenon has recently been reported as an element of plant responses to specific abiotic stresses [63].

Zn-induced up-regulation of monosaccharides (Table S1(2–4)) is in agreement with the metabolic adjustment events reported in the leaves of *Camellia sinensis*, where these metabolites were assumed to be involved in osmoprotection [21]. The presence of pentoses in the Zn^2+^-associated up-regulation patterns is also in agreement with the earlier published assumption that HMs (Cd^2+^ in particular) can trigger the switch of carbohydrate oxidative catabolism from glycolysis to the PPP [18]. A two-fold up-regulation of *myo*-inositol in the roots and young leaves of Zn-treated *A. caudatus* might be a part of the general stress response—this effect was earlier reported for *A. hypochondriacus* subjected to viral infection and was interpreted as a signaling event [64].

In this study, galactose appeared to be the most Zn^2+^-responsive sugar in *A. caudatus* roots (Table 2). Galactose is known to play an essential role as a component of cell wall polysaccharides [65]. Roots can respond to trace metals by actively remodeling their metabolism and up-regulating specific biosynthetic pathways [66]. Thus, the walls of epidermal and cortical root cells were shown to thicken in the presence of Zn^2+^ excess, which was explained by enhancement of pectin biosynthesis [67,68]. Due to the presence of multiple carboxyl groups in the galacturonyl monomers of pectins, these polymers can efficiently scavenge Zn^2+^ ions, and wall thickening might essentially increase the capacity of root cells for Zn^2+^ binding [69]. This feature of pectins underlies one of the most efficient mechanisms of HM detoxification and HM-stress tolerance [70]. It is assumed that this mechanism might explain 30–40% of the total Zn^2+^ scavenging capacity of the root cells [69]. This corresponds well with our AAS data (Figure 2), which indicates that the major part of the absorbed Zn^2+^ was deposited in amaranth roots, thus preventing Zn^2+^ translocation to the shoot. Given the possible role of galactose as a precursor in ascorbic acid biosynthesis [65] via the *L*-galactose pathway [71], an increase in galactose content in the amaranth roots may also be associated with the involvement of ascorbate in the response to abiotic stress [72].

In addition to the above-mentioned aspects, galactose could contribute to the synthesis of galactolipids. Activation of the galactolipid metabolism was reported in maize and arabidopsis roots under phosphate deficiency conditions [73] and in the leaves of *N. caerulescens* treated with excess Zn [74]. This mechanism is considered to be an adaptive strategy, which involves remodeling of membrane lipids in response to phosphate deprivation: substitution of phospholipids with galactolipids [73,75]. Depletion of the available phosphate pools in the Zn-treated amaranth root cells may occur due to the direct Zn chelation by phosphate or/and increased demands for energy metabolism. This, in turn, might promote the synthesis of galactolipids instead of phospholipids.

Thus, the patterns of Zn-induced up-regulation in the metabolomes of *A. caudatus* roots and leaves may indicate the following roles of sugars in the HM-stress response: (i) osmotic protection, (ii) allocation of metabolites (in particular, sucrose) from mature leaves to young leaves, (iii) ROS scavenging (relying predominantly on oligosaccharides), (iv) switching the carbohydrate oxidative catabolism from glycolysis to the PPP (that is manifested with characteristic dynamics of pentoses), signaling (accomplished by *myo*-inositol), (v) remodeling of cell walls and, most likely, membranes to enhance the Zn-binding capacity of these structures (galactosyl residues in membrane lipids, pectins and other polymers of the cell wall).

### 3.3. Organic Acids in the Response of Amaranth Plants to Zn Stress

Organic acids represent the second compound class in the metabolome of *A. caudatus*, which was strongly affected by Zn^2+^ treatment. Among these compounds, salicylic acid (SA) was especially strongly up-regulated in young leaves and roots (Table 1 and Table 2). This metabolite is one of the key plant hormones involved in activation of diverse stress responses [76,77,78], including HM-induced metabolic shifts such as the enhancement of redox metabolism and activation of antioxidant defense, in particular, up-regulation of secondary metabolism and biosynthesis of ROS-scavenging osmoprotectors [78,79].

Although plants’ responses to HM stress are generally well characterized, Zn-induced biochemical alterations are still understudied. It was suggested that SA can induce alternative oxidase (AOX), which contributes to redox and metabolic homeostasis and signaling in plant cells [80]. Thus, SA might be involved in both ROS production and scavenging [81]. In particular, AOX1 was found to modulate oxidative stress triggered by Cd^2+^ exposure [17]. However, to date, this mechanism of stress tolerance has not been experimentally confirmed for other HMs. The strong induction of SA in *A. caudatus* roots and leaves exposed to Zn^2+^ (Table 1 and Table 2) suggests that this metabolite may be involved in AOX up-regulation. This assumption is further supported by a significant increase in the content of shikimic acid (which is known as the key intermediate in the biosynthesis of aromatic acids) in young leaves and roots (Table 1 and Table 2) [82]. The pathways of the SA biosynthesis in plants are still not fully understood. Currently, two pathways are discussed: the first relies on cinnamic acid and phenylalanine ammonia-lyase (PAL), while the second originates from isochorismate (IC) and relies on IC synthase [83]. The second pathway is typical for plants and is characteristic, for example, of arabidopsis [84].

The observed Zn-dependent up-regulation of shikimic acid in young leaves and roots is in good agreement with the stress-related dynamics of phenylpropanoids in mature leaves. In particular, the increased levels of *p*- and *o*-coumaric acids (Supplementary Information S1, Table S1(1–4) might indicate the enhancement of lignin biosynthesis, which underlies the improvement of cell wall rigidity [85]. On the other hand, in roots, the metabolites of this group (e.g., caffeic acid, Table 2) showed mostly a down-regulation pattern in response to Zn stress. Caffeic acid is an important intermediate in lignin biosynthesis, a pathway which is known to be involved in the HM-response [86]. A similar decrease in caffeic acid content in response to application of Cu, Cd and Pb was reported for tomatoes [87]. Interestingly, the opposite effect was shown for *Matricaria chamomilla* under Ni-exposure [88]. It cannot be ruled out that the decrease in the level of caffeic acid is associated with a strong enhancement of SA biosynthesis in *A. caudatus* exposed to Zn stress and the switching of the phenylpropanoid pathway to its production.

Gluconic acid and five unknown sugar acids demonstrated a dramatic increase in their content in roots of Zn-exposed *A. caudatus*, whereas a similar response in leaves was less pronounced (Supplementary Information S1, Table S1(2,3)). Accumulation of gluconic acid was earlier reported in radish roots in response to application of Cd^2+^ and Pb^2+^ [15] and in leaves of corns upon the application of Cu^2+^ [89]. An especially strong (up to 34-fold) increase in gluconate content was shown in leaves of Zn hyperaccumulator *N. caerulescens* upon the application of 0.5 mmol/L Zn^2+^ [74]. The ability of this metabolite to form stable complexes with Cd^2+^ has been intensively discussed. However, reliable assignment of the Zn^2+^ coordination chemistry in planta is challenging [74].

While the shifts in plant metabolism underlying the above-described responses remain largely unknown, the pathways of gluconate biosynthesis have been well studied in yeast. In these organisms, the biosynthesis of gluconate relies on the oxidation of glucose in the so-called NADP^+^-dependent glucose dehydrogenase—gluconate shunt of PPP [90]. Since this reaction does not involve glucose-6-phosphate dehydrogenase (G6PDH), the regulatory enzyme controlling the rate of the entire cycle, it can be considered as an alternative route for the glucose entry into the PPP. A similar shunt is believed to function in plants [91], although the specific mechanisms for its Zn-dependent activation and its relevance for other aldonic acids still need investigation. Meanwhile, G6PDH was shown to be a Cd^2+^- [92] and Zn^2+^-inducible metabolite [93]. Thus, assuming an ubiquitous character of this phenomenon, both pathways of gluconate biosynthesis might be involved in the plant response to Zn stress.

It should be emphasized that non-enzymatic autoxidation of glucose might contribute to the development of metal-induced oxidative stress. Thus, enhanced ROS generation and associated overproduction of reactive carbonyl compounds (RCCs) can be expected under stress conditions [94]. These highly reactive molecules readily interact with biopolymers [95]. Although the resulting conjugates were confirmed *in vivo* [48], their physiological effects in plants still remain unknown [96]. Our data indicate stronger accumulation of gluconate in roots of *A. caudatus* compared to leaves (Table 1 and Table 2), which suggests the involvement of gluconic acid in Zn^2+^ chelation directly in roots.

Organic acids of the TCA cycle (malate, citrate) and oxalate are involved in the well-known mechanism of HM tolerance in plants [29,30,31]. It was shown that citrate is more efficient than malate in the chelating of Zn^2+^ ions [29]. However, in response to Zn-stress, the tissue level of both acids in young leaves did not change, while only a slight increase in their content was detected in roots and mature leaves. Oxalate is known to considerably contribute to Zn^2+^ complexation in plants with high tissue content of this acid [97], such as *A. caudatus* [30]. In our study, we showed that oxalate was one of the abundant metabolites in all tested organs of amaranth (Table 3, Supplementary Information S1, Table S1(9)). However, Zn application did not result in any changes in the content of soluble oxalate in young leaves and caused a slight decrease in its level in mature leaves and roots. The latter observation could be attributed to the formation of insoluble Zn^2+^ oxalate complexes in the walls of root cells [97]. This phenomenon can lead to a sharp decrease in oxalate concentration in tissue liquids. At the analytical level, it might even result in recognition of this metabolite as “below detection limit” by the conventional GC-MS employed here.

To date, the HM-related shifts in the tissue pools of organic acids have been reported only for the specific case of Cd-induced plant stress [17,19]. This aspect was mostly discussed in terms of the stress-induced activation of mitochondrial AOX [17]. The dynamics of some TCA cycle intermediates observed here, together with similar data on Cd^2+^ stress [17,19], suggest Zn-dependent stimulation of the mitochondrial activity via enhancement of ATP, NAD(P)H and antioxidant biosynthesis. Our data are also in line with the recently published assumption of Kaur and Garg [25]. The authors proposed that plant respiration can be enhanced under high Zn^2+^ levels that might result in the overproduction of multiple organic acids via the TCA cycle. This might underlie the onset of Zn^2+^ tolerance, since most of the accumulated acids can be involved in the sequestration of excess Zn^2+^ in vacuoles. Moreover, Zn-associated changes in organic acid content (i.e., up-regulation of malate and citrate and down-regulation of succinate) are consistent with the concept of stress-induced metabolic adjustment through the switch of the TCA cycle from a “closed” to an “open” configuration with the formation of malate and citrate “valves” provoked by increase in cell oxidative status [98]. Since amaranth is a C4 plant, its pools of C3 and C4 organic acids represent reserves of energy (ATP) and reducing equivalents (NADPH) in adaptation to environmental stresses [99].

Strong up-regulation of glyoxylic acid in young leaves (Table 1) can be explained in the context of the following considerations. This toxic intermediate of the glyoxylate cycle is detoxified in planta with the formation of oxalate and may thus be considered as a putative precursor for oxalate synthesis in oxalate-rich plants [100]. As this mechanism can be relevant for amaranth, it needs to be studied in more detail in the context of Zn^2+^ stress. In addition, glyoxylate might impact the prospective glycation-based regulatory pathways in plants [52].

Overall, the accumulation of salicylic, shikimic, and gluconic acids, some TCA cycle intermediates, oxalate and glyoxylate observed in this study might be a result of Zn-dependent activation of the phenylpropanoid pathway, gluconate shunt, PPP, cell respiration and glyoxylate cycle. These organic acids are involved in the processes of redox signaling and homeostasis (salicylic acid), ATP and NADPH synthesis, chelating and sequestrating of Zn excess into vacuoles (gluconate, citrate, malate), and cell wall rigidification (phenylpropanoids).

### 3.4. Fatty Acids in the Response of Amaranth Plants to Zn Stress

Fatty acids (FAs) represent another group of amaranth metabolites affected by Zn^2+^ stress. Most of the differentially accumulated FAs were saturated (octanoic, behenic, stearic, arachidic, myristic and heptadecanoic acids), whereas unsaturated FAs were represented only by oleic acid and linoleic acids. Many saturated acids accumulated in young leaves of zinc-stressed plants, while linoleic acid showed the opposite trend (Table 1). These results are consistent with the data for spinach leaves [101], which demonstrated an increase in saturated FAs accompanied by a decrease in unsaturated FAs in response to increasing doses of Cd^2+^.

Among FAs, the octanoic acid was most strongly affected, displaying >100-fold accumulation in young leaves. The more pronounced effect of Zn^2+^ on FA contents in young leaves compared to other organs might be explained by the role of octanoic acid in (i) *de novo* fatty acid biosynthesis in mitochondria and (ii) biosynthesis of lipoic acid, an important cofactor of mitochondrial oxo-acid dehydrogenase complexes and glycine decarboxylase [102]. Furthermore, the up-regulation of long-chain saturated FAs (e.g., stearic and myristic acids) might indicate the enhancement of wax and cutin biosynthesis, which is known to be more active in growing tissues [103].

The pathway analysis confirmed a more efficient stress-induced involvement of FAs in the pathways of lipid, cutin, wax, and suberin biosynthesis in young leaves compared to roots (Figure 6). Although the pathway analysis accomplished for up- and down-regulated intermediates of lipid biosynthesis gave the most confident results for roots, the impact of FAs in these pathways (especially the linoleic acid metabolism) was still higher for the young leaves (Figure 6; Supplementary Information S1, Table S1(2,3); Supplementary Information S3, Parts S1 and S2). In this regard, a slight decrease in the level of linoleic acid (C18:2) in young leaves of *A. caudatus* could be caused by its enhanced involvement in the synthesis of cutin under Zn^2+^ stress [104].

To summarize, the increase in the content of saturated FAs in response to the accumulation of Zn^2+^ in tissues may be considered as an adaptive feature of amaranth plants. These metabolic rearrangements might contribute to the deposition of cutin and wax in the walls of leaf cells, but also might improve the rigidity of leaf and root cell membranes to make them less Zn^2+^-permeable.

### 3.5. Nitrogen Metabolites in the Response of Amaranth Plants to Zn Stress

The dynamics of nitrogen-containing metabolites were intensively discussed in the context of plant responses to HM stress [9,105] with special attention paid to the role of proline as a key molecule contributing to stress tolerance. This metabolite is universally recognized as one of the most important plant osmolytes, a stress-associated signaling molecule, a potent HM chelator and ROS scavenger [105,106]. Thus, a two-fold increase in proline content was shown for Zn-stressed *Vigna unguiculata* seedlings [107] and up to four-fold for *Sinapis alba* seedlings [108]. However, in some plants, Zn^2+^ appeared to be less efficient in triggering proline accumulation compared to other HMs [9,28].

Our study revealed a Zn-induced increase in the levels of proteinogenic amino acids such as leucine, alanine and proline only in roots, but not in young and mature leaves of *A. caudatus* (Table 1 and Table 2, Supplementary Information S1, Table S1(4)). These results correspond well with the pattern of metabolic responses reported for *Arabidopsis thaliana* under Cd^2+^ stress [17]. Keunen and co-workers attributed the up-regulation of N-containing metabolites to the primary role of roots as the first contact site for HMs in plants, and discussed it in the context of related signaling pathways and protective mechanisms.

Regarding the zinc effect, it has been reported that 0.5 mmol/L Zn^2+^ induced the accumulation of proline in the leaves of stress-sensitive *Lactuca sativa* plants but not in stress-tolerant species such as *Brassica oleracea* [9]. Thus, the up-regulation of proline in plant tissues might be considered as a symptom of Zn stress rather than a marker of tolerance to Zn toxicity. So far, the genus *Amaranthus* has not been addressed in terms of the proline response to Zn-induced stress. However, this aspect was considered in the context of salinity stress. Thus, Wouyou and co-workers reported that the salt-induced increase in proline content in a tolerant cultivar of *A. cruentus* could be expressed only in roots [109]. Therefore, an increase in the proline content in Zn-treated *A. caudatus* plants (which was manifested mostly in roots) could be considered as an adaptive response driven by stress-induced metabolic adjustments.

An unexpected result was the Zn-induced increase in the content of pyroglutamic acid (PG) in young amaranth leaves, accompanied by a decrease in its level in the roots (Table 1 and Table 2). Currently, the biosynthesis of this cyclic lactam in plants is discussed in the context of its production via glutathione (GSH) reduction-oxidation and degradation [110]. Since GSH metabolism is affected under HM stress, changes in PG dynamics may be observed [110]. Moreover, PG was suggested to be a putative precursor of glutamate, and it was hypothesized that this compound is a major contributor to the steady-state glutamate level in plant leaves and is a main reservoir for glutamate and glutathione [111]. Thus, the conversion of PG to proline under stress conditions seems likely [112]. However, the formation of PG can also be a method-related artifact. Indeed, at least partly, the measured PG levels can be underlined by the spontaneous conversion of glutamic acid to PG under high temperature conditions of the GC injector.

Ethanolamine is one more analyte to be discussed. This compound attracted our attention due to a Zn-dependent up-regulation, which was observed; however, this was only in young leaves (Table 1). Despite its well-known importance in stress response [113], the volatility of this metabolite raises doubts about the possibility of its reliable detection by our GC-MS protocol. Indeed, most likely, this compound might be evacuated during freeze drying and/or evaporation under reduced pressure (which are the ultimate steps of the sample preparation procedure). Therefore, we suggest that ethanolamine appears as a method-related artifact, a degradation product of thermolabile N-metabolites, which readily degrade under high temperature conditions of the splitless-split injector.

Three non-proteinogenic amino acid derivatives, namely *N*-acetyl-serine, 5-methyl-cytosine and *N*,*N*-dimetylglycine, were up-regulated in the cells of Zn-treated amaranth plants. This was also the case for adenine and several essential nucleosides—guanosine and uridine (Table 1 and Table 2, Supplementary Information S1, Table S1(4)). Most of these metabolites are known as effectors and secondary messengers directly involved in the transduction of intracellular signals and hormone regulation of stress responses in plants [114,115]. The patterns and the degree of this up-regulation in Zn-treated amaranth depended on the leaf age (Table 1, Supplementary Information S1, Table S1(4)). Generally, results of the pathway analysis indicate that nitrogen metabolism is more strongly involved in the Zn-induced responses in roots than in leaves of *A. caudatus*. Nevertheless, young leaves were also considerably affected (Figure 6, Supplementary Information S1, Figure S1(10), Supplementary Information S3). Notably, pronounced up-regulation of *N*-acetyl-serine and 5-methyl-cytosine in young leaves might imply the enhanced methylation of the corresponding substrates in response to Zn exposure. *N*-acetyl-serine may originate from its positioning isomer, *O*-acetyl-serine, and both these isomers are involved in the biosynthesis of the sulfur-containing amino acids [115]. Therefore, the increase in the abundance of *N*-acetyl-serine shown in our research might relate to the active turnover of *S*-adenosylmethionine, a universal donor of methyl groups. An increase in the content of 5-methyl-cytosine might indicate the activation of DNA methylation under Zn exposure. It is known that activation of DNA methylation is involved in epigenetic regulation underlying the tolerance to different stress conditions [116], including the HM stress [117,118].

Thus, alterations in N-metabolism in Zn-exposed plants might indicate involvement of (i) proline-related signaling, (ii) ROS scavenging in roots, (iii) induction of PG (and/or glutamate) biosynthesis in young leaves and roots and (iv) DNA methylation in Zn-induced stress tolerance of *A. caudatus*.

**Figure 7:**
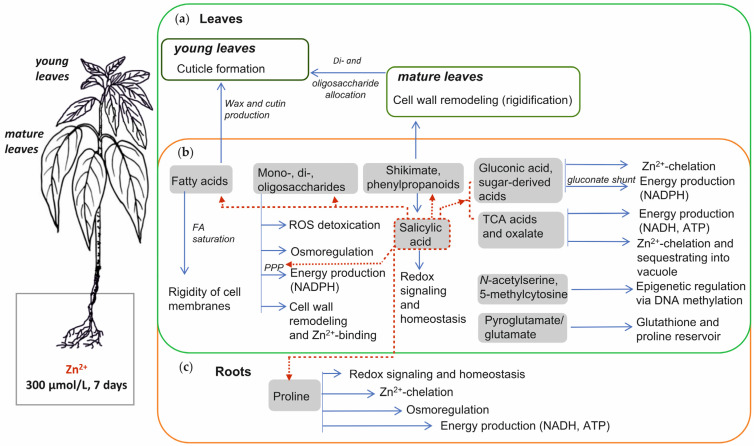
*Amaranthus caudatus* metabolic shifts and possible biological responses induced by Zn-treatment (Zn^2+^ 300 μmol/L, 7 days). The leaf-specific (**a**), leaf and root common (**b**) and root-specific (**c**) metabolic responses to the Zn excess exposure. Red dashed line and arrows highlight the proposed on the basis of literature [119,120,121,122,123] central role of salicylic acid and its regulations in the metabolic response of *A. caudatus*.

Finally, regarding the possible cross-talk between the biochemical processes and individual metabolites discussed above, it is important to note that the accumulation of salicylic acid might be a crucial event in the metabolic response of *A. caudatus* roots and young leaves to Zn stress. This signaling molecule may serve as a key node controlling various metabolic pathways. The primary function of the plant hormone is regulating the induction of antioxidant systems to balance increased ROS production under stressful conditions [81]. Not less importantly, SA can regulate sugar metabolism, triggering soluble sugar accumulation in leaves and roots [119]. These sugars function as osmotic regulators, ROS scavengers, and carbon and energy substrates. Moreover, SA is able to sustain a high energy level in plant cells by redirecting sugar substrates from glycolysis to the PPP, thus providing NADPH, which is necessary to cope with oxidative stress and is required for various biosynthetic processes [120]. The key intermediate of the PPP, 6-phosphogluconic acid, may be a precursor of gluconic acid, a chelator of Zn^2+^ ions. SA may also trigger the synthesis of proline in roots [121], activate the phenylpropanoid pathway leading to lignin biosynthesis in mature leaves [122] and probably might induce alterations in lipid metabolism leading to cutin and wax synthesis in young leaves similar to those shown in fruits [123]. Most of the putative SA effects enhancing stress tolerance in plants were studied under stresses other than HM exposure. Thus, a strong SA up-regulation in leaves and roots of Zn-treated plants found in our work may open new perspectives to further examine the role of this hormone as a stress metabolite. An overview of the Zn-related metabolic responses of *A. caudatus* leaves and roots is presented in Figure 7.

## 4. Materials and Methods

### 4.1. Reagents

Unless stated otherwise, materials were obtained from the following manufacturers: Conlac GmbH (Leipzig, Germany): hexane (puriss p. a.); Macherey-Nagel GmbH and Co KG (Düren, Germany): *N*-methyl-*N*-(trimethylsilyl)trifluoroacetamide (MSTFA, MS grade); Reanal (Budapest, Hungary): *L*-aspartic acid, 2-oxoglutaric acid; Vekton (Saint-Petersburg, Russia): methanol (LC grade). All other chemicals were purchased from Merck KGaA (Darmstadt, Germany). Water was purified in-house with a water conditioning and purification system Millipore Milli-Q Gradient A10 system (resistance 18 mΩ/cm, Merck Millipore, Darmstadt, Germany).

### 4.2. Plant Culturing and Zn^2+^ Stress Application

*Amaranthus caudatus* L.-variety Karwa dauta plants were used in the study. The seeds were obtained from the collection of vegetable crops of Vavilov All-Russia Institute of Plant Genetic Resources (Saint Petersburg, Russia). The seeds were surface sterilized with 3% (*v*/*v*) H_2_O_2_ solution for 20 min, washed with deionized water and germinated in containers filled with calcined quartz sand. The plants were grown in two independent experiments with an identical setup (*n* = 9 per treatment group, in total 27 plants in nine vessels) under a 16:8 day/night regimen, 70–75% relative humidity, and day/night temperatures of 24/18 °C. Light was provided by fluorescent lamps with a wavelength range of 320–780 nm and a photosynthetic photon flux density of 120 µmol m^−2^ s^−1^.

During the first seven days after germination, the seedlings were watered with a ten-fold diluted nutrient solution (0.1 n.s., Supplementary Information S1, Protocol S1-2) three times a day. For the next three weeks, the concentration of the solution was increased on a weekly basis to 0.2 n.s., 0.5 n.s. and 1.0 n.s, respectively. The four-week-old plants were transferred to new 3 L hydroponic vessels filled with the full-strength (1.0 n.s.) nutrient solution (the setup relied on three plants per vessel).

After two weeks of culturing in the hydroponic system, the vessels with six-week-old plants with well-developed (i.e., mature) leaves that could be easily distinguished from young leaves were randomly split into three equal groups (*n* = 3). Plants from the first group were harvested before stress application for a separate biomass assessment of their roots, young and mature leaves. The second group of plants was designated as “Zn-treated”. These plants were subjected to Zn^2+^ stress for one week, which was accomplished by supplementation of 300 µmol/L ZnSO_4_·7H_2_O in the nutrient solution. This choice for Zn^2+^ concentration and the Zn^2+^ stress duration was based on the available literature data [24,25,49], including those for amaranth [35]. The plants of the third group were referred to as “controls” and remained untreated. The experiments were set in three biological replicates, i.e., three vessels with a total of nine plants in each group. The Zn-treated and control plants were characterized by an array of physiological parameters (stomatal conductivity, chlorophyll content, photosystem II activity, leaf relative water contents) prior (Day 0) and after the Zn-stress exposure (Day 7), and harvested afterwards for further biomass assessment, atomic absorption spectroscopy and metabolomics experiments.

### 4.3. Physiological Assays

Chlorophyll content, photosystem II activity and stomatal conductivity were assessed in a nondestructive way as described in [124,125,126], respectively, using the 3rd (counting from the plant top) fully expanded mature leaf of each experimental and control plant. The measurements relied on the portable devices: chlorophyll meter atLEAF (FT Green LLC, Wilmington, DE, USA), fluorometer Junior-PAM (HeinzWalz GmbH, Effeltrich, Germany), porometer SC-1 (Delta-T Devices Ltd., Cottbus, Germany), respectively, according to the manufacturer’s instructions. The obtained atLEAF units were used to calculate chlorophyll content (mg · cm^2^) [124]. The leaf relative water contents (LRWC) were as follows: LRWC (%) = (fresh weight − dry weight) × 100%/fresh weight. For the determination of this parameter for young and mature leaves, the 4th young (i.e., not fully expanded) leaf and 4th mature (fully expanded) leaf, respectively, as indicated in Supplementary Information S1, Table S1(4), were collected from each plant.

### 4.4. Plant Fresh and Dry Biomass Determination

Roots, young and mature leaves of six- and seven-week-old plants were collected separately. The roots were sequentially rinsed for 5 min with 0.1 mmol/L CaCl_2_ and deionized water. Afterwards, the roots, young and mature leaves were weighed, oven-dried at 105 °C for 1 h and dried at 70 °C for the following 24 h. Thus, the fresh and dry weights were determined prior to and after the Zn^2+^ application.

### 4.5. Atomic Absorption Spectroscopy (AAS)-Based Analysis of Zn in Plant Organs

The contents of Zn in roots, young and mature leaves of Zn-treated and control plants were determined in the relevant dried and ground plant samples by atomic absorption spectroscopy (AAS, Shimadzu AA-7000, Kyoto, Japan). Plant samples (100 mg) were placed in heat-resistant glass flasks and digested with 5 mL of concentrated HNO_3_/HClO_4_ 4:1 (*v*/*v*) at 160 °C (electric stove), followed by subsequent dilution to 100 mL with deionized water and further Zn analysis [127].

The fresh, dry weights and metal contents were assessed for pooled samples (three leaf or root samples to build one experimental replicate, in total *n* = three per group).

### 4.6. GC-MS-Based Metabolite Profiling

Profiling of polar primary metabolites relied on the in-house established protocol, including (i) extraction of plant material with aq. methanol, (ii) subsequent derivatization of the extracted primary metabolites and (iii) their analysis by gas chromatography-electron ionization-quadrupole-mass spectrometry (GC-EI-Q-MS) as described elsewhere [128] with a few modifications indicated in Supplementary Information S1, Protocol S1-3. The prepared samples (1 μL) containing derivatized metabolites were analyzed by a GC2010 gas chromatograph coupled online to a quadrupole mass selective detector, Shimadzu GCMS QP201, operating under the instrumental settings summarized in Table S1(10). An assessment of the method performance relied on quality controls (QCs, i.e., aliquots of the pool prepared by mixing all individual extracts), which were included in the sequence for the GC-MS analysis [128]. The quality of the acquired chromatograms was assessed by verification of the baseline regularity, background MS noise, as well as the symmetry, width and height of peaks. To obtain qualitative information about individual metabolites, the chromatograms were processed by AMDIS (version 2.66 from 08.08.2008, www.amdis.net, accessed on 5 March 2020). This software was used to accomplish deconvolution of mass spectra, peak picking, Kovach retention indices (RI) calculation by retention times (*t_R_*) of C_8_–C_20_ alkanes and annotation of analytes. The further analysis relied on the untargeted approach, i.e., unbiased TIC-based identification of all signals with the signal-to-noise ratio (S/N) ≥ 3. For this, the trimethylsilyl (TMS) and methyl oxime (MEOX)-TMS derivatives underlying all detected individual chromatographic peaks (corresponding to individual MEOX/TMS derivatives, further defined as features) were annotated by *t_R_*, RI and EI-MS data—the results of spectral similarity search with the NIST Search engine against available electron ionization (EI)-MS spectral libraries such as National Institute of Standards and Technology (NIST, https://webbook.nist.gov/chemistry/, accessed on 10 March 2020), Golm Metabolome Database (GMD, http://gmd.mpimp-golm.mpg.de/, accessed on 10 March 2020), and in-house spectral library. The similarity between the experimental spectra and the reference EI-MS entries in external libraries was assessed by the match factor. Its values, such as >800, 799–700, 699–600, 599–500 and <500, were interpreted as excellent, very good, good, satisfactory and unsatisfactory results, respectively (Table S1(1) and Figure S1(11)). Relative quantification was accomplished via direct comparison of individual analyte abundances, derived as integral areas of corresponding peaks in extracted ion chromatograms (XIC, *m*/*z* ± 0.5 Da) for representative intense signals at specific *t_R_* XIC. The analyte quantification procedure was accomplished with Xcalibur^TM^ and LCquan^TM^ (version 2.5.6, ThermoFisher Scientific Inc., Bremen, Germany) and MSDial software (https://systemsomicslab.github.io/compms/msdial/main.html, accessed on 10 March 2020), which performs alignment of chromatograms by *t_R_* of analytes and the integration of analyte peak areas. The quantitation results (i.e., integrated peak areas detected in each sample) were organized into a digital matrix and were statistically processed by using the Metaboanalyst 4.0 (https://www.metaboanalyst.ca/, accessed on 16 March 2020). Prior to the statistical analysis, the data were normalized by dry weight of samples and filtered to exclude the features not detected in ≥20% of the samples. For metabolites which were not detected in less than 20% of samples (and less than 30% per experimental group), imputation of missing values was performed by the random forest algorithm with the help of MetImp 1.2 on-line tool [129]. The quantitative metabolomics data were analyzed by principal component analysis (PCA). To visualize changes in the levels of individual assigned metabolites, hierarchical clustering analysis with heatmap representation was accomplished.

Targeted absolute quantitative analysis relied on external standardization with 29 authentic standards (oxalic acid, malonic acid, succinic acid, tartaric acid, malic acid, aconitic acid, citric acid, fumaric acid, benzoic acid, ascorbic acid, erythronic acid, glycerol, arabinose, glucose, galactose, *myo*-inositol, sucrose, urea, Ala, Trp, Ile, Leu, Asn, Asp, Glu, Pro, Val, Ser, Thr) prepared as a total mix serially diluted in the range from 0.2 μmol/L to 0.2 mmol/L. The statistical interpretation and bioinformatics post-processing of the acquired quantitative data relied on MetaboAnalyst 5.0 (https://www.metaboanalyst.ca accessed on 10 September 2021) online platform [130].

### 4.7. Metabolic Pathway Analysis

Annotation of individual metabolites to Zn-responsive pathways relied on the *Arabidopsis thaliana* pathway library (deposited by KEGG on-line platform from 03.2020). The pathway analysis was accomplished with MetaboAnalyst 4.0 software (https://www.metaboanalyst.ca, accessed on 19 March 2020) and employed the combination of pathway enrichment analysis by the globaltest method and pathway topology analysis [131]. As the pathway enrichment analysis relies on the compound-specific abundance values, only significantly (*t*-test *p* ≤ 0.05) differentially abundant structurally annotated metabolites were included in the input list. The pathway topology analysis gives access to the pathways, in which the changes in more important positions of the enzyme network more significant impact on the observed differences. To assess the node centrality (i.e., to estimate node importance), the betweenness centrality method was applied.

### 4.8. Statistics

The quantitative metabolomics data were analyzed by the methods of multivariate (principal component analysis, PCA) and univariate (volcano plot) statistics. Results of the PCA are presented as score plots built for the first two principal components. The plots were obtained for abundances of individual metabolites detected in the form of TMS and TMS-MEOX derivatives by untargeted GC-EI-Q-MS of *A. caudatus* aq. methanolic extracts, which were prepared from young leaves and roots. The PCA score plots were built in MetaboAnalyst 4.0 (https://www.metaboanalyst.ca, accessed on 16 March 2020). The results of the univariate statistical analysis (presented as figures and tables) were expressed as mean ± standard deviation (StD) of three biological replicates. Significance of the differences in the contents of individual metabolites between Zn-treated groups and untreated controls was estimated by *t*-test (Student’s test). Thereby, for the untargeted metabolite profiling, the threshold values for the *p*-value and fold change (FC) were 0.05 and 1.5, respectively, as they are often in metabolomics studies [132]. For the targeted analysis, whose main focus is highly sensitive, precise absolute quantification of selected metabolites, the FC threshold was decreased to 1.3. To address the reliability of the observed Zn^2+^-related changes in the abundances of individual metabolites, the false discovery rates (FDRs) were assessed at *p* ≤ 0.05 with the Benjamini–Hochberg method for all comparisons [133].

The results obtained by the targeted approach were expressed in μmol/g DW as mean ± standard deviation (StD) of three biological replicates (each pooled from three individual leaves or root samples). All calculations were performed within the linear dynamic ranges (LDRs, R^2^ ≥ 0.95) derived in additional dilution experiments (*n* = 3) with individual authentic standards. The resulting individual calibration curves were built on a logarithmic scale for the analyte peak areas as a function of the applied concentration (0.2, 0.5, 1, 2, 5, 10, 20, 50, 100 and 200 μmol/L, Supplementary Information S1, Figure S1(12)).

## 5. Conclusions

In this study, we provided a first insight into the metabolic responses of *A. caudatus* plants to moderate Zn-stress. Our metabolomics survey revealed strong changes in the contents of multiple primary metabolites in Zn-stressed plants. The more pronounced changes in the metabolic profiles of amaranth roots compared to leaves indicate a clear organ specificity of the stress-induced biochemical rearrangements. At least in part, this effect may be explained by the crucial role of roots in Zn uptake and detoxication in plants. Sugars and organic acids were the principal up-regulated metabolites in all organs of *A. caudatus*, which may indicate their key role during the early stages of the plant’s biochemical adaptation to Zn-related toxicity. The specific dynamics of sugar metabolites in different organs may reflect the activation of source-sink relocation of reserve di- and oligosaccharides. The involvement of mono- and disaccharides in the stress response can be attributed to their role in ROS scavenging and osmoregulation. The observed increase in galactose content in the roots suggests that root cell walls contribute to the scavenging of Zn^2+^ ions. However, an increased demand for galactose to maintain the synthesis of membrane galactolipids under stress cannot be ruled out. The higher responsivity of organic acids to Zn^2+^ in roots might be attributed to their active participation in Zn^2+^ chelation. The extremely high (59-fold) up-regulation of gluconic acid in roots, which can be caused either by direct glucose oxidation or by the putative gluconate shunt of the pentose phosphate pathway, may prevent free Zn^2+^ transfer from roots to leaves. The impact of the TCA cycle metabolites in stress-induced metabolic adjustments was much less pronounced. The accumulation of salicylic acid both in young leaves and roots suggests a possible role of this signaling molecule in the activation of Zn-stress tolerance mechanisms in *A. caudatus* plants. Increased saturated fatty acids in Zn-stressed plants may cause rigidification of cell membranes in both leaves and roots, as well as cuticle formation in young leaves. Finally, the up-regulation of the phenylpropanoid pathway intermediates may underlie the enhancement of lignin biosynthesis and stiffening of the cell walls. Among N-metabolites, proline was the most affected compound in the roots of *A. caudatus*. The accumulation of *N*-acetylserine and 5-metylcitosine in roots and leaves may be an indicator of DNA-methylation and epigenetic regulation induced by exposure to Zn^2+^. In general, our results showed that adaptation of *A. caudatus* to Zn-stress was accompanied by considerable metabolic shifts involving a large set of metabolic pathways. Since biochemical rearrangements can be carried out at both posttranslational and transcriptional levels, in the next step of our study, we examined the proteomic response of *A. caudatus* to Zn exposure.

## Figures and Tables

**Table 3:** Tissue contents of the metabolites identified as differentially regulated in young leaves and roots of *A. caudatus* in response to Zn^2+^ treatment ^a^.

Metabolite	Average Content, μmol/g DW ^b^								YL (Zn-Treated v. Cont.)		R (Zn-Treated v. Cont.)	
	YL Cont		YL Zn		R Cont		R Zn					
	μmol/g DW	±StD	μmol/g DW	±StD	μmol/g DW	±StD	μmol/g DW	±StD	FC ^c^	*p* ^d^	FC	*p*
Metabolites demonstrating an increase in tissue contents in response to Zn^2+^ treatment in comparison to control												
Young leaf and root metabolites												
Galactose (1MEOX, 5TMS)	0.05	0.01	0.10	0.03	0.05	0.004	0.38	0.02	**↑ 2.0**	**0.04**	**↑ 7.8**	**≤0.001**
Glucose (1MEOX, 5TMS)	0.26	0.02	0.76	0.2	0.93	0.06	3.3	0.28	**↑ 2.9**	**0.02**	**↑ 3.5**	**≤0.001**
*myo*-Inositol (6TMS)	0.31	0.06	0.59	0.2	0.36	0.03	0.74	0.08	**↑ 1.9**	**0.04**	**↑ 2.0**	**0.001**
Young leaf metabolites												
Pyroglutamic acid (1&2TMS)	10.0	6.1	25.0	7.2	19.0	1.9	18.0	3.8	**↑ 2.5**	**0.05**	↓ ≤1.3	0.46
Sucrose (8TMS)	2.2	0.7	17.0	9.1	7.6	0.3	8.2	0.83	**↑ 7.6**	**0.05**	**↑** ≤1.3	0.31
Root metabolites												
Malonic acid (2TMS)	0.74	0.11	0.8	0.2	0.5	0.04	0.9	0.10	1.0	0.90	**↑ 1.9**	**0.002**
Citric acid (4TMS)	0.31	0.08	0.4	0.2	0.2	0.003	0.4	0.05	**↑** 1.3	0.51	**↑ 2.1**	**0.003**
Erythronic acid (4TMS)	2.5	0.2	3.0	0.7	1.0	0.08	2.0	0.09	**↑** ≤1.3	0.38	**↑ 1.9**	**≤0.001**
Alanine (2&3TMS)	7.9	3.3	3.8	0.9	1.4	0.07	2.9	0.47	↓ 2.1	0.11	**↑ 2.1**	**0.005**
Valine (2TMS)	0.59	0.20	0.4	0.09	0.5	0.03	1.1	0.34	↓ 1.6	0.15	**↑ 2.1**	**0.051**
Leucine (2TMS)	0.80	0.05	0.6	0.2	1.2	0.13	2.1	0.47	↓ ≤1.3	0.21	**↑ 1.7**	**0.036**
Proline (2TMS)	0.09	0.15	0.1	0.04	0.2	0.14	0.9	0.03	**↑** ≤1.3	0.86	**↑ 5.1**	**0.001**
Glycerol (3TMS)	1.4	0.1	2.0	0.8	3.0	0.10	4.3	0.11	**↑** 1.4	0.25	**↑ 1.4**	**≤0.001**
Arabinose (1MEOX, 4TMS)	0.21	0.02	0.2	0.05	0.07	0.01	0.14	0.02	1.0	0.87	**↑ 1.9**	**0.004**
Metabolites demonstrating a decrease in tissue contents in response to Zn^2+^ treatment in comparison to control												
Young leaf and root metabolites												
Succinic acid (2TMS)	4.2	0.14	2.2	0.7	2.6	0.1	1.7	0.13	**↓ 1.9**	**0.01**	**↓ 1.6**	**0.001**
Young leaf metabolites												
Benzoic acid (1TMS)	10.0	0.5	8.1	0.2	9.1	2.5	10.0	0.25	**↓ 1.3**	**0.002**	**↑** ≤1.3	0.58
Root metabolites												
Oxalic acid (2TMS)	4.9	2.1	4.6	1.3	10.7	0.6	7.3	0.79	↓ 1.1	0.85	**↓ 1.5**	**0.004**
Metabolites without significant changes in content in response to Zn^2+^ treatment in comparison to control												
Fumaric acid (2TMS)	0.44	0.04	0.4	0.1	0.8	0.03	0.8	0.05	↓ ≤1.3	0.37	1.0	0.93
Malic acid (2TMS)	2.2	0.2	2.3	1.1	4.7	0.1	5.6	0.56	**↑** ≤1.3	0.80	**↑** ≤1.3	0.057
Aconitic acid (3TMS)	1.4	0.5	1.1	0.4	0.05	0.02	0.1	0.13	↓ ≤1.3	0.55	**↑** 2.2	0.484
Isoleucine (1&2TMS)	0.16	0.03	0.1	0.06	0.4	0.04	0.5	0.07	↓ 1.7	0.17	**↑** 1.3	0.062
Urea (2TMS)	3.7	5.8	0.3	0.00	1.5	1.3	3.9	2.12	↓ 11.8	0.37	**↑** 2.5	0.18

^a^ The analysis relied on the targeted GC-EI-Q-MS assay; ^b^ The average contents of metabolites in μmol/g DW (dry weight) and their standard deviations (StD) found in young leaves (YL cont) and roots (R cont) of control plants as well as in the organs of the plants exposed to Zn^2+^ excess, YL Zn and R Zn, respectively, are presented. ^c^ FC, fold change (not less than 1.3-fold) in the metabolite content in Zn-treated samples in comparison to controls. Arrows indicate increased (↑) or decreased (↓) contents of individual metabolites in the Zn-treated sample in comparison to the controls. ^d^ *T*-test, *p*-value. Statistically significant (*t*-test, *p* ≤ 0.05) differences are marked in bold font. FC value 1.0 indicates no difference between the compared Zn-treated and untreated samples.

## Data Availability

All relevant data are available within the article and Supplementary Materials. The raw GC-MS data files related to the research are available on the NIH Common Fund’s National Metabolomics Data Repository (NMDR) website, the Metabolomics Workbench, https://www.metabolomicsworkbench.org, accessed on 29 January 2024, where it has been assigned Project ID PR001910. The data can be accessed directly via its Project DOI: 10.21228/M8TT6W. Additionally, all the initial analytical data obtained when estimating the peak areas on chromatograms are also available and could be provided by request.

## Supplementary Materials

The following supporting information can be downloaded at: https://www.mdpi.com/article/10.3390/plants14142119/s1, Supplementary Information S1: Protocols, Figures and Tables; Supplementary Information S2: Measurements and calculations of chlorophyll content (Table S2(1)), photosystem II activity (Table S2(2)), stomatal conductivity (Table S2(3)), LRWC (Table S2(4)), fresh weight (Table S2(5)), dry weight (Table S2(6)), Zn^2+^ content (Table S2(7)); Supplementary Information S3: Metabolic Pathways revealed by the Pathway Analysis; Supplementary Information S4: Pathway Analysis detailed information.

## Abbreviations

AOX, alternative oxidase; DW, dry weight; EI, electron ionization; EI-MS, electron ionization mass spectrum; FA, fatty acid; FC, fold change; FDR, false discovery rate; G6PDH, glucose-6-phosphate dehydrogenase; GC-EI-Q-MS, gas chromatography-electron ionization-quadrupole mass spectrometry; GDM, Golm Metabolome Database; GSH, glutathione; HM, heavy metals; HMDB, Human Metabolome Database; KEGG, Kyoto Encyclopedia of Genes and Genomes; LDR, linear dynamic ranges; LRWC, leaf related water content; MEOX-TMS, methyl oxime-trimethylsilyl; MSTFA, *N*-methyl-*N*-(trimethylsilyl) trifluoroacetamide; n.s., nutrient solution; N-metabolites, nitrogen containing metabolites; NIST, National Institute of Standards and Technology; PC, principal component; PCA, principal component analysis; PG, pyroglutamic acid; P-metabolites, phosphate containing metabolites; PPP, pentose phosphate pathway; RI, retention index; ROS, reactive oxygen species; SA, Salicylic acid; StD, standard deviation; TCA cycle, tricarboxylic acid cycle; TIC, total ion current; TMS, trimethylsilyl; *t_R_*, retention time; XIC, extracted ion chromatograms.

## References

[1] Ghori N.-H., Ghori T., Hayat M.Q., Imadi S.R., Gul A., Altay V., Ozturk M. (2019). Heavy metal stress and responses in plants. Int. J. Environ. Sci. Technol..

[2] Goyal D., Yadav A., Prasad M., Singh T.B., Shrivastav P., Ali A., Dantu P.K., Mishra S., Naeem M., Ansari A.A., Gill S.S. (2020). Effect of Heavy Metals on Plant Growth: An Overview. Contaminants in Agriculture: Sources, Impacts and Management.

[3] El-Sappah A.H., Zhu Y., Huang Q., Chen B., Soaud S.A., Abd Elhamid M.A., Yan K., Li J., El-Tarabily K.A. (2024). Plants’ molecular behavior to heavy metals: From criticality to toxicity. Front. Plant Sci..

[4] Sharma S.S., Dietz K.-J. (2009). The relationship between metal toxicity and cellular redox imbalance. Trends Plant Sci..

[5] Hossain M.A., Liu F., Burritt D.J., Fujita M., Huang B. (2020). Priming-Mediated Stress and Cross-Stress Tolerance in Crop Plants.

[6] Gratani L. (2014). Plant Phenotypic Plasticity in Response to Environmental Factors. Adv. Bot..

[7] Singh M., Kumar J., Singh S., Singh V.P., Prasad S.M., Singh M.P.V.V.B. (2015). Adaptation Strategies of Plants against Heavy Metal Toxicity: A Short Review. Biochem. Pharmacol..

[8] Zhang H., Zhu J., Gong Z., Zhu J.-K. (2022). Abiotic stress responses in plants. Nat. Rev. Genet..

[9] Paradisone V., Barrameda-Medina Y., Montesinos-Pereira D., Romero L., Esposito S., Ruiz J.M. (2015). Roles of some nitrogenous compounds protectors in the resistance to zinc toxicity in *Lactuca sativa* cv. Phillipus and *Brassica oleracea* cv. Bronco. Acta Physiol. Plant..

[10] Patel M.K., Pandey S., Kumar M., Haque M.I., Pal S., Yadav N.S. (2021). Plants Metabolome Study: Emerging Tools and Techniques. Plants.

[11] Allwood J.W., Williams A., Uthe H., van Dam N.M., Mur L.A.J., Grant M.R., Pétriacq P. (2021). Unravelling Plant Responses to Stress—The Importance of Targeted and Untargeted Metabolomics. Metabolites.

[12] Carrera F.P., Noceda C., Maridueña-Zavala M.G., Cevallos-Cevallos J.M. (2021). Metabolomics, a Powerful Tool for Understanding Plant Abiotic Stress. Agronomy.

[13] Jamla M., Khare T., Joshi S., Patil S., Penna S., Kumar V. (2021). Omics approaches for understanding heavy metal responses and tolerance in plants. Curr. Plant Biol..

[14] Jorge T.F., Rodrigues J.A., Caldana C., Schmidt R., van Dongen J.T., Thomas-Oates J., António C. (2016). Mass spectrometry-based plant metabolomics: Metabolite responses to abiotic stress. Mass Spectrom. Rev..

[15] Wang Y., Xu L., Shen H., Wang J., Liu W., Zhu X., Wang R., Sun X., Liu L. (2015). Metabolomic analysis with GC-MS to reveal potential metabolites and biological pathways involved in Pb & Cd stress response of radish roots. Sci. Rep..

[16] Sun X., Zhang J., Zhang H., Ni Y., Zhang Q., Chen J., Guan Y. (2010). The responses of *Arabidopsis thaliana* to cadmium exposure explored via metabolite profiling. Chemosphere.

[17] Keunen E., Florez-Sarasa I., Obata T., Jozefczak M., Remans T., Vangronsveld J., Fernie A.R., Cuypers A. (2016). Metabolic responses of *Arabidopsis thaliana* roots and leaves to sublethal cadmium exposure are differentially influenced by ALTERNATIVE OXIDASE1a. Environ. Exp. Bot..

[18] D’Alessandro A., Taamalli M., Gevi F., Timperio A.M., Zolla L., Ghnaya T. (2013). Cadmium Stress Responses in *Brassica juncea*: Hints from Proteomics and Metabolomics. J. Proteome Res..

[19] Xie Y., Hu L., Du Z., Sun X., Amombo E., Fan J., Fu J. (2014). Effects of Cadmium Exposure on Growth and Metabolic Profile of Bermudagrass [*Cynodon dactylon* (L.) Pers.]. PLoS ONE.

[20] Hédiji H., Djebali W., Cabasson C., Maucourt M., Baldet P., Bertrand A., Boulila Zoghlami L., Deborde C., Moing A., Brouquisse R. (2010). Effects of long-term cadmium exposure on growth and metabolomic profile of tomato plants. Ecotoxicol. Environ. Saf..

[21] Zhang Y., Wang Y., Ding Z., Wang H., Song L., Jia S., Ma D. (2017). Zinc stress affects ionome and metabolome in tea plants. Plant Physiol. Biochem. PPB.

[22] Zemanová V., Pavlíková D., Novák M., Hnilička F. (2024). The Dual Role of Zinc in Spinach Metabolism: Beneficial × Toxic. Plants.

[23] Kopittke P.M., Blamey F.P.C., Asher C.J., Menzies N.W. (2010). Trace metal phytotoxicity in solution culture: A review. J. Exp. Bot..

[24] Tsonev T., Lidon F. (2012). Zinc in plants—An overview. Emir. J. Food Agric..

[25] Kaur H., Garg N. (2021). Zinc toxicity in plants: A review. Planta.

[26] Broadley M.R., White P.J., Hammond J.P., Zelko I., Lux A. (2007). Zinc in plants. New Phytol..

[27] Sagardoy R., Morales F., Rellán-Álvarez R., Abadía A., Abadía J., López-Millán A.F. (2011). Carboxylate metabolism in sugar beet plants grown with excess Zn. J. Plant Physiol..

[28] Li X., Yang Y., Jia L., Chen H., Wei X. (2013). Zinc-induced oxidative damage, antioxidant enzyme response and proline metabolism in roots and leaves of wheat plants. Ecotoxicol. Environ. Saf..

[29] Anjum N.A., Hasanuzzaman M., Hossain M.A., Thangavel P., Roychoudhury A., Gill S.S., Rodrigo M.A.M., Adam V., Fujita M., Kizek R. (2015). Jacks of metal/metalloid chelation trade in plants—An overview. Front. Plant Sci..

[30] Osmolovskaya N., Vu D.V., Kuchaeva L. (2018). The role of organic acids in heavy metal tolerance in plants. Biol. Commun..

[31] Panchal P., Miller A.J., Giri J. (2021). Organic acids: Versatile stress-response roles in plants. J. Exp. Bot..

[32] Bosiacki M., Kleiber T., Kaczmarek J. (2013). Evaluation of Suitability of *Amaranthus Caudatus* L. and *Ricinus Communis* L. in Phytoextraction of Cadmium and Lead from Contaminated Substrates. Arch. Environ. Prot..

[33] Watanabe T., Murata Y., Osaki M. (2009). Amaranthus Tricolor Has the Potential for Phytoremediation of Cadmium-Contaminated Soils. Commun. Soil Sci. Plant Anal..

[34] Ko C.-H., Chang F.-C., Wang Y.-N., Chung C.-Y. (2014). Extraction of Heavy Metals from Contaminated Soil by Two *Amaranthus* spp.. CLEAN-Soil Air Water.

[35] Lukatkin A.S., Bashmakov D.I., Al Harbawee W.E.Q., Teixeira da Silva J.A. (2021). Assessment of physiological and biochemical responses of *Amaranthus retroflexus* seedlings to the accumulation of heavy metals with regards to phytoremediation potential. Int. J. Phytoremediat..

[36] Hunková J., Lisinovičová M., Lancikova V., Szabóová M. (2025). A comparative analysis of heavy metal stress responses in different grain amaranth cultivars. ResearchGate.

[37] Rastogi A., Shukla S. (2013). Amaranth: A new millennium crop of nutraceutical values. Crit. Rev. Food Sci. Nutr..

[38] Achigan-Dako E.G., Sogbohossou O.E.D., Maundu P. (2014). Current knowledge on *Amaranthus* spp.: Research avenues for improved nutritional value and yield in leafy amaranths in sub-Saharan Africa. Euphytica.

[39] Ragasa C.Y., Austria J.P.M., Subosa A.F., Torres O.B., Shen C.-C. (2015). Chemical Constituents of *Amaranthus viridis*. Chem. Nat. Compd..

[40] Joshi D.C., Sood S., Hosahatti R., Kant L., Pattanayak A., Kumar A., Yadav D., Stetter M.G. (2018). From zero to hero: The past, present and future of grain amaranth breeding. Theor. Appl. Genet..

[41] Ludwig M. (2016). The Roles of Organic Acids in C4 Photosynthesis. Front. Plant Sci..

[42] Gélinas B., Seguin P. (2007). Oxalate in grain amaranth. J. Agric. Food Chem..

[43] Osmolovskaya N.G., Dung V.V., Kudryashova Z.K., Kuchaeva L.N., Popova N.F. (2018). Effect of Cadmium on Distribution of Potassium, Calcium, Magnesium, and Oxalate Accumulation in *Amaranthus cruentus* L. Plants. Russ. J. Plant Physiol..

[44] Harvey D.J., Vouros P. (2020). Mass spectrometric fragmentation of trimethylsilyl and related alkylsilyl derivatives. Mass Spectrom. Rev..

[45] Boldizsár I., Füzfai Z., Molnár-Perl I. (2011). Characteristic fragmentation patterns of trimethylsilyl and trimethylsilyl-oxime derivatives of plant disaccharides as obtained by gas chromatography coupled to ion-trap mass spectrometry. J. Chromatogr. A.

[46] Füzfai Z., Boldizsár I., Molnár-Perl I. (2008). Characteristic fragmentation patterns of the trimethylsilyl and trimethylsilyl-oxime derivatives of various saccharides as obtained by gas chromatography coupled to ion-trap mass spectrometry. J. Chromatogr. A.

[47] Kalinova J., Dadakova E. (2009). Rutin and total quercetin content in amaranth (*Amaranthus* spp.). Plant Foods Hum. Nutr. (Dordr. Neth.).

[48] Paudel G., Bilova T., Schmidt R., Greifenhagen U., Berger R., Tarakhovskaya E., Stöckhardt S., Balcke G.U., Humbeck K., Brandt W. (2016). Osmotic stress is accompanied by protein glycation in *Arabidopsis thaliana*. J. Exp. Bot..

[49] Chen H., Song L., Zhang H., Wang J., Wang Y., Zhang H. (2022). Cu and Zn Stress affect the photosynthetic and antioxidative systems of alfalfa (*Medicago sativa*). J. Plant Interact..

[50] Keunen E., Peshev D., Vangronsveld J., Van Den Ende W., Cuypers A. (2013). Plant sugars are crucial players in the oxidative challenge during abiotic stress: Extending the traditional concept. Plant Cell Environ..

[51] Pommerrenig B., Ludewig F., Cvetkovic J., Trentmann O., Klemens P.A.W., Neuhaus H.E. (2018). In Concert: Orchestrated Changes in Carbohydrate Homeostasis Are Critical for Plant Abiotic Stress Tolerance. Plant Cell Physiol..

[52] Shumilina J., Kusnetsova A., Tsarev A., Janse van Rensburg H.C., Medvedev S., Demidchik V., Van den Ende W., Frolov A. (2019). Glycation of Plant Proteins: Regulatory Roles and Interplay with Sugar Signalling?. Int. J. Mol. Sci..

[53] Peshev D., Vergauwen R., Moglia A., Hideg E., Van den Ende W. (2013). Towards understanding vacuolar antioxidant mechanisms: A role for fructans?. J. Exp. Bot..

[54] Gangola M.P., Ramadoss B.R., Wani S.H. (2018). Chapter 2—Sugars Play a Critical Role in Abiotic Stress Tolerance in Plants. Biochemical, Physiological and Molecular Avenues for Combating Abiotic Stress Tolerance in Plants.

[55] Couée I., Sulmon C., Gouesbet G., El Amrani A. (2006). Involvement of soluble sugars in reactive oxygen species balance and responses to oxidative stress in plants. J. Exp. Bot..

[56] Bolouri-Moghaddam M.R., Le Roy K., Xiang L., Rolland F., Van den Ende W. (2010). Sugar signalling and antioxidant network connections in plant cells. FEBS J..

[57] Wu D., Cai S., Chen M., Ye L., Chen Z., Zhang H., Dai F., Wu F., Zhang G. (2013). Tissue Metabolic Responses to Salt Stress in Wild and Cultivated Barley. PLoS ONE.

[58] Du Y., Zhao Q., Chen L., Yao X., Zhang W., Zhang B., Xie F. (2020). Effect of drought stress on sugar metabolism in leaves and roots of soybean seedlings. Plant Physiol. Biochem. PPB.

[59] Thomas A., Beena R., Laksmi G., Soni K.B., Alex S., Viji M.M. (2022). Changes in sucrose metabolic enzymes to water stress in contrasting rice genotypes. Plant Stress.

[60] Turgeon R. (1989). The Sink-Source Transition in Leaves. Annu. Rev. Plant Physiol. Plant Mol. Biol..

[61] Geiger D. (2011). Plant Sucrose Transporters from a Biophysical Point of View. Mol. Plant.

[62] Dong S., Zhang J., Beckles D.M. (2018). A pivotal role for starch in the reconfiguration of 14C-partitioning and allocation in *Arabidopsis thaliana* under short-term abiotic stress. Sci. Rep..

[63] Paul M.J., Watson A., Griffiths C.A. (2020). Linking fundamental science to crop improvement through understanding source and sink traits and their integration for yield enhancement. J. Exp. Bot..

[64] Srivastava S., Bisht H., Sidhu O.P., Srivastava A., Singh P.C., Pandey R.M., Raj S.K., Roy R., Nautiyal C.S. (2012). Changes in the metabolome and histopathology of *Amaranthus hypochondriacus* L. in response to *Ageratum enation* virus infection. Phytochemistry.

[65] Zhang G.-Y., Liu R.-R., Zhang C.-Q., Tang K.-X., Sun M.-F., Yan G.-H., Liu Q.-Q. (2015). Manipulation of the Rice L-Galactose Pathway: Evaluation of the Effects of Transgene Overexpression on Ascorbate Accumulation and Abiotic Stress Tolerance. PLoS ONE.

[66] Krzesłowska M. (2011). The cell wall in plant cell response to trace metals: Polysaccharide remodeling and its role in defense strategy. Acta Physiol. Plant..

[67] Glińska S., Gapińska M., Michlewska S., Skiba E., Kubicki J. (2016). Analysis of *Triticum aestivum* seedling response to the excess of zinc. Protoplasma.

[68] Doyama K., Yamaji K., Haruma T., Ishida A., Mori S., Kurosawa Y. (2021). Zn tolerance in the evergreen shrub, *Aucuba japonica*, naturally growing at a mine site: Cell wall immobilization, aucubin production, and Zn adsorption on fungal mycelia. PLoS ONE.

[69] Caldelas C., Weiss D.J. (2017). Zinc Homeostasis and isotopic fractionation in plants: A review. Plant Soil.

[70] Krzesłowska M., Rabęda I., Basińska A., Lewandowski M., Mellerowicz E.J., Napieralska A., Samardakiewicz S., Woźny A. (2016). Pectinous cell wall thickenings formation—A common defense strategy of plants to cope with Pb. Environ. Pollut..

[71] Castro J.C., Castro C.G., Cobos M. (2023). Genetic and biochemical strategies for regulation of L-ascorbic acid biosynthesis in plants through the L-galactose pathway. Front. Plant Sci..

[72] Xiao M., Li Z., Zhu L., Wang J., Zhang B., Zheng F., Zhao B., Zhang H., Wang Y., Zhang Z. (2021). The Multiple Roles of Ascorbate in the Abiotic Stress Response of Plants: Antioxidant, Cofactor, and Regulator. Front. Plant Sci..

[73] Wang F., Ding D., Li J., He L., Xu X., Zhao Y., Yan B., Li Z., Xu J. (2020). Characterisation of genes involved in galactolipids and sulfolipids metabolism in maize and Arabidopsis and their differential responses to phosphate deficiency. Funct. Plant Biol..

[74] Foroughi S., Baker A.J.M., Roessner U., Johnson A.A.T., Bacic A., Callahan D.L. (2014). Hyperaccumulation of zinc by *Noccaea caerulescens* results in a cascade of stress responses and changes in the elemental profile. Metallomics.

[75] Härtel H., Dormann P., Benning C. (2000). DGD1-independent biosynthesis of extraplastidic galactolipids after phosphate deprivation in Arabidopsis. Proc. Natl. Acad. Sci. USA.

[76] Agnihotri A., Gupta P., Dwivedi A., Seth C.S. (2018). Counteractive mechanism (s) of salicylic acid in response to lead toxicity in *Brassica juncea* (L.) Czern. cv. Varuna. Planta.

[77] Yu L.-L., Liu Y., Zhu F., Geng X.-X., Yang Y., He Z.-Q., Xu F. (2020). The enhancement of salt stress tolerance by salicylic acid pretreatment in *Arabidopsis thaliana*. Biol. Plant.

[78] Yang H., Fang R., Luo L., Yang W., Huang Q., Yang C., Hui W., Gong W., Wang J. (2023). Uncovering the mechanisms of salicylic acid-mediated abiotic stress tolerance in horticultural crops. Front. Plant Sci..

[79] Sharma A., Sidhu G.P.S., Araniti F., Bali A.S., Shahzad B., Tripathi D.K., Brestic M., Skalicky M., Landi M. (2020). The Role of Salicylic Acid in Plants Exposed to Heavy Metals. Molecules.

[80] Saha B., Borovskii G., Panda S.K. (2016). Alternative oxidase and plant stress tolerance. Plant Signal. Behav..

[81] Poór P. (2020). Effects of Salicylic Acid on the Metabolism of Mitochondrial Reactive Oxygen Species in Plants. Biomolecules.

[82] Chen Z., Zheng Z., Huang J., Lai Z., Fan B. (2009). Biosynthesis of salicylic acid in plants. Plant Signal. Behav..

[83] Zhong Q., Hu H., Fan B., Zhu C., Chen Z. (2021). Biosynthesis and Roles of Salicylic Acid in Balancing Stress Response and Growth in Plants. Int. J. Mol. Sci..

[84] Torrens-Spence M.P., Bobokalonova A., Carballo V., Glinkerman C.M., Pluskal T., Shen A., Weng J.-K. (2019). PBS3 and EPS1 Complete Salicylic Acid Biosynthesis from Isochorismate in Arabidopsis. Mol. Plant.

[85] Liu Q., Luo L., Zheng L. (2018). Lignins: Biosynthesis and Biological Functions in Plants. Int. J. Mol. Sci..

[86] Riaz U., Kharal M.A., Murtaza G., Zaman Q.U., Javaid S., Malik H.A., Aziz H., Abbas Z. (2019). Prospective Roles and Mechanisms of Caffeic Acid in Counter Plant Stress: A Mini Review. Pak. J. Agric. Res..

[87] Kisa D., Kayir O., Saglam N., Şahin S., Öztürk L., Elmastaş M. (2019). Changes of phenolic compounds in tomato associated with the heavy metal stress. J. Nat. Appl. Sci..

[88] Kovácik J., Klejdus B., Backor M. (2009). Phenolic metabolism of *Matricaria chamomilla* plants exposed to nickel. J. Plant Physiol..

[89] Zhao L., Huang Y., Keller A.A. (2018). Comparative Metabolic Response between Cucumber (*Cucumis sativus*) and Corn (*Zea mays*) to a Cu(OH)2 Nanopesticide. J. Agric. Food Chem..

[90] Corkins M.E., Wilson S., Cocuron J.-C., Alonso A.P., Bird A.J. (2017). The gluconate shunt is an alternative route for directing glucose into the pentose phosphate pathway in fission yeast. J. Biol. Chem..

[91] Kornecki J.F., Carballares D., Tardioli P.W., Rodrigues R.C., Berenguer-Murcia Á., Alcántara A.R., Fernandez-Lafuente R. (2020). Enzyme production of D-gluconic acid and glucose oxidase: Successful tales of cascade reactions. Catal. Sci. Technol..

[92] Yang Y., Fu Z., Su Y., Zhang X., Li G., Guo J., Que Y., Xu L. (2014). A cytosolic glucose-6-phosphate dehydrogenase gene, ScG6PDH, plays a positive role in response to various abiotic stresses in sugarcane. Sci. Rep..

[93] Van Assche F., Cardinaels C., Clijsters H. (1988). Induction of enzyme capacity in plants as a result of heavy metal toxicity: Dose-response relations in *Phaseolus vulgaris* L., treated with zinc and cadmium. Environ. Pollut. (Barking, Essex: 1987).

[94] Ávila F., Schmeda-Hirschmann G., Silva E. (2017). The Major Chromophore Arising from Glucose Degradation and Oxidative Stress Occurrence during Lens Proteins Glycation Induced by Glucose. Molecules.

[95] Antonova K., Vikhnina M., Soboleva A., Mehmood T., Heymich M.-L., Leonova T., Bankin M., Lukasheva E., Gensberger-Reigl S., Medvedev S. (2019). Analysis of Chemically Labile Glycation Adducts in Seed Proteins: Case Study of Methylglyoxal-Derived Hydroimidazolone 1 (MG-H1). Int. J. Mol. Sci..

[96] Soboleva A., Frolova N., Bureiko K., Shumilina J., Balcke G., Zhukov V., Tikhonovich I., Frolov A. (2022). Dynamics of Reactive Carbonyl Species in Pea Root Nodules in Response to Polyethylene Glycol (PEG)-Induced Osmotic Stress. Int. J. Mol. Sci..

[97] Rauser W.E. (1999). Structure and function of metal chelators produced by plants: The case for organic acids, amino acids, phytin, and metallothioneins. Cell Biochem. Biophys..

[98] Igamberdiev A.U., Eprintsev A.T. (2016). Organic Acids: The Pools of Fixed Carbon Involved in Redox Regulation and Energy Balance in Higher Plants. Front. Plant Sci..

[99] Igamberdiev A.U., Bykova N.V. (2018). Role of organic acids in the integration of cellular redox metabolism and mediation of redox signalling in photosynthetic tissues of higher plants. Free Radic. Biol. Med..

[100] Zarei A., Brikis C.J., Bajwa V.S., Chiu G.Z., Simpson J.P., DeEll J.R., Bozzo G.G., Shelp B.J. (2017). Plant Glyoxylate/Succinic Semialdehyde Reductases: Comparative Biochemical Properties, Function during Chilling Stress, and Subcellular Localization. Front. Plant Sci..

[101] Zemanová V., Pavlík M., Pavlíková D., Kyjaková P. (2015). Changes in the contents of amino acids and the profile of fatty acids in response to cadmium contamination in spinach. Plant Soil Environ..

[102] Wada H., Shintani D., Ohlrogge J. (1997). Why do mitochondria synthesize fatty acids? Evidence for involvement in lipoic acid production. Proc. Natl. Acad. Sci. USA.

[103] Koch K., Barthlott W. (2006). Plant Epicuticular Waxes: Chemistry, Form, Self-Assembly and Function. Nat. Prod. Commun..

[104] He M., Ding N.-Z. (2020). Plant Unsaturated Fatty Acids: Multiple Roles in Stress Response. Front. Plant Sci..

[105] Sharma S.S., Dietz K.-J. (2006). The significance of amino acids and amino acid-derived molecules in plant responses and adaptation to heavy metal stress. J. Exp. Bot..

[106] Singh S., Singh P., Tomar R.S., Sharma R.A., Singh S.K., Rani M., Chaudhary B.S., Jamal S., Kumar P. (2022). Proline: A Key Player to Regulate Biotic and Abiotic Stress in Plants. Towards Sustainable Natural Resources: Monitoring and Managing Ecosystem Biodiversity.

[107] Singh J., Hembram P., Basak J. (2014). Potential of *Vigna unguiculata* as a Phytoremediation Plant in the Remediation of Zn from Contaminated Soil. Am. J. Plant Sci..

[108] Repkina N., Nilova I., Kaznina N. (2023). Effect of Zinc Excess in Substrate on Physiological Responses of *Sinapis alba* L.. Plants.

[109] Wouyou A., Prodjinoto H., Zanklan A.S., Vanpee B., Lutts S., Gandonou C.B. (2019). Implication of Ions and Organic Solutes Accumulation in Amaranth (*Amaranthus cruentus* L.) Salinity Resistance. Am. J. Plant Sci..

[110] Dorion S., Ouellet J.C., Rivoal J. (2021). Glutathione Metabolism in Plants under Stress: Beyond Reactive Oxygen Species Detoxification. Metabolites.

[111] Kumar A., Bachhawat A.K. (2012). Pyroglutamic acid: Throwing light on a lightly studied metabolite. Curr. Sci..

[112] Jiménez-Arias D., García-Machado F.J., Morales-Sierra S., Luis J.C., Suarez E., Hernández M., Valdés F., Borges A.A. (2019). Lettuce plants treated with L-pyroglutamic acid increase yield under water deficit stress. Environ. Exp. Bot..

[113] Moussa H.R., Selem E.E.-S.M., Ghramh H.A. (2019). Ethanolamine affects physiological responses of salt-treated jute plants. Int. J. Veg. Sci..

[114] Van Damme T., Blancquaert D., Couturon P., Van Der Straeten D., Sandra P., Lynen F. (2014). Wounding stress causes rapid increase in concentration of the naturally occurring 2′,3′-isomers of cyclic guanosine- and cyclic adenosine monophosphate (cGMP and cAMP) in plant tissues. Phytochemistry.

[115] Watanabe M., Chiba Y., Hirai M.Y. (2021). Metabolism and Regulatory Functions of O-Acetylserine, S-Adenosylmethionine, Homocysteine, and Serine in Plant Development and Environmental Responses. Front. Plant Sci..

[116] Abdulraheem M.I., Xiong Y., Moshood A.Y., Cadenas-Pliego G., Zhang H., Hu J. (2024). Mechanisms of Plant Epigenetic Regulation in Response to Plant Stress: Recent Discoveries and Implications. Plants.

[117] Gallo-Franco J.J., Sosa C.C., Ghneim-Herrera T., Quimbaya M. (2020). Epigenetic Control of Plant Response to Heavy Metal Stress: A New View on Aluminum Tolerance. Front. Plant Sci..

[118] Fasani E., Giannelli G., Varotto S., Visioli G., Bellin D., Furini A., DalCorso G. (2023). Epigenetic Control of Plant Response to Heavy Metals. Plants.

[119] Dong C.-J., Wang X.-L., Shang Q.-M. (2011). Salicylic acid regulates sugar metabolism that confers tolerance to salinity stress in cucumber seedlings. Sci. Hortic..

[120] Chen Y., Sun J., Lin H., Lin M., Lin Y., Wang H., Hung Y.-C. (2020). Salicylic acid reduces the incidence of *Phomopsis longanae* Chi infection in harvested longan fruit by affecting the energy status and respiratory metabolism. Postharvest Biol. Technol..

[121] (2019). Regulatory Role of Proline in Heat Stress Tolerance: Modulation by Salicylic Acid. Plant Signaling Molecules.

[122] Wang Z., Guo J., Luo W., Niu S., Qu L., Li J., Chen Y., Li G., Yang H., Lu D. (2025). Salicylic Acid Cooperates with Lignin and Sucrose Signals to Alleviate Waxy Maize Leaf Senescence Under Heat Stress. Plant Cell Environ..

[123] Jiang B., Liu R., Fang X., Tong C., Chen H., Gao H. (2022). Effects of salicylic acid treatment on fruit quality and wax composition of blueberry (*Vaccinium virgatum* Ait). Food Chem..

[124] Richardson A.D., Duigan S.P., Berlyn G.P. (2002). An Evaluation of Noninvasive Methods to Estimate Foliar Chlorophyll Content. New Phytol..

[125] Murchie E.H., Lawson T. (2013). Chlorophyll fluorescence analysis: A guide to good practice and understanding some new applications. J. Exp. Bot..

[126] Monteith J.L., Campbell G.S., Potter E.A. (1988). Theory and performance of a dynamic diffusion porometer. Agric. For. Meteorol..

[127] Hina B., Rizwani G.-H.-, Naseeb U., Huma A., Hyder Z. (2023). Application of Atomic Absorption Spectroscopy to determine Mineral and Heavy Metal distribution level of Medicinal Plants. J. Anal. Tech. Res..

[128] Leonova T., Popova V., Tsarev A., Henning C., Antonova K., Rogovskaya N., Vikhnina M., Baldensperger T., Soboleva A., Dinastia E. (2020). Does Protein Glycation Impact on the Drought-Related Changes in Metabolism and Nutritional Properties of Mature Pea (*Pisum sativum* L.) Seeds?. Int. J. Mol. Sci..

[129] Wei R., Wang J., Su M., Jia E., Chen S., Chen T., Ni Y. (2018). Missing Value Imputation Approach for Mass Spectrometry-based Metabolomics Data. Sci. Rep..

[130] Pang Z., Chong J., Zhou G., de Lima Morais D.A., Chang L., Barrette M., Gauthier C., Jacques P.-É., Li S., Xia J. (2021). MetaboAnalyst 5.0: Narrowing the gap between raw spectra and functional insights. Nucleic Acids Res..

[131] Goeman J.J., Bühlmann P. (2007). Analyzing gene expression data in terms of gene sets: Methodological issues. Bioinformatics.

[132] Cochran C., Martin K., Rafferty D., Choi J., Leontyev A., Shetty A., Kurup S., Puthanveetil P. (2023). Untargeted Metabolomics Based Prediction of Therapeutic Potential for Apigenin and Chrysin. Int. J. Mol. Sci..

[133] Benjamini Y., Hochberg Y. (1995). Controlling the False Discovery Rate: A Practical and Powerful Approach to Multiple Testing. J. R. Stat. Soc. Ser. B (Methodol.).

