# Supplementary material for: Metabolic Responses of Amaranthus caudatus Roots and Leaves to Zinc Stress
Source: Plants (Basel). 2025 Jul 9;14(14):2119. doi: 10.3390/plants14142119 (PMC12300844; doi:10.3390/plants14142119)
Supplement: Supplementary file 1 [file plants-14-02119-s001.zip › Osmolovskaya_et_al_Supplementary_S4.1_Young_leaves_PathwayAnalysis_Report.pdf]

Metabolomic Data Analysis with MetaboAnalyst 4.0

Name: guest447328871261268466

March 22, 2020

1 Background

The Pathway Analysis module combines results from powerful pathway enrichment analysis with pathway topology analysis to help researchers identify the most relevant pathways involved in the conditions under study.

There are many commercial pathway analysis software tools such as Pathway Studio, MetaCore, or Ingenuity Pathway Analysis (IPA), etc. Compared to these commercial tools, the pathway analysis module was specifically developed for metabolomics studies. It uses high-quality KEGG metabolic pathways as the backend knowledgebase. This module integrates many well-established (i.e. univariate analysis, over-representation analysis) methods, as well as novel algorithms and concepts (i.e. Global Test, GlobalAncova, network topology analysis) into pathway analysis. Another feature is a Google-Map style interactive visualization system to deliver the analysis results in an intuitive manner.

2 Data Input

The Pathway Analysis module accepts either a list of compound labels (common names, HMDB IDs or KEGG IDs) with one compound per row, or a compound concentration table with samples in rows and compounds in columns. The second column must be phenotype labels (binary, multi-group, or continuous). The table is uploaded as comma separated values (.csv).

3 Compound Name Matching

The first step is to standardize the compound labels used in user uploaded data. This is a necessary step since these compounds will be subsequently compared with compounds contained in the pathway library. There are three outcomes from the step - exact match, approximate match (for common names only), and no match. Users should click the textbfView button from the approximate matched results to manually select the correct one. Compounds without match will be excluded from the subsequently pathway analysis.

Table 1 shows the conversion results. Note: *1* indicates exact match, *2* indicates approximate match, and *0* indicates no match. A text file contain the result can be found the downloaded file *name_map.csv*

Table 1: Result from Compound Name Mapping

	Query	Match	HMDB	PubChem	KEGG	SMILES
1	HMDB0000119	Glyoxylic acid	HMDB0000119	760	C00048	C(=O)C(=O)O
2	HMDB0000267	Pyroglutamic acid	HMDB0000267	7405	C01879	C1CC(=O)N[C@@H]1C(=O)O
3	HMDB0000355	3-Hydroxymethylglutaric acid	HMDB0000355	1662	C03761	CC(CC(=O)O)(CC(=O)O)O
4	HMDB0000150	Gluconolactone	HMDB0000150	7027	C00198	C([C@@H]1[C@H]([C@@H]([C@H](C(=O)O1)O)
5	HMDB0001900	Ribonolactone	HMDB0001900	111064	C02674	C([C@@H]1[C@H]([C@H](C(=O)O1)O)O)O
6	HMDB0000625	Gluconic acid	HMDB0000625	10690	C00257	C([C@H]([C@H]([C@@H]([C@H](C(=O)O)O)O)O)
7	HMDB0003070	Shikimic acid	HMDB0003070	8742	C00493	C1[C@H]([C@@H]([C@@H](C=C1C(=O)O)O)O)
8	HMDB0001895	Salicylic acid	HMDB0001895	338	C00805	C1=CC=C(C(=C1)C(=O)O)O
9	HMDB0000482	Caprylic acid	HMDB0000482	379	C06423	CCCCCCCC(=O)O

10	HMDB0000827	Stearic acid	HMDB0000827	5281	C01530	CCCCCCCCCCCCCCCCCC(=O)O
11	HMDB0002212	Arachidic acid	HMDB0002212	10467	C06425	CCCCCCCCCCCCCCCCCCCC(=O)O
12	HMDB0000806	Myristic acid	HMDB0000806	11005	C06424	CCCCCCCCCCCCCC(=O)O
13	HMDB0000207	Oleic acid	HMDB0000207	445639	C00712	CCCCCCCC/C=C\CCCCCCCC(=O)O
14	HMDB0002259	Heptadecanoic acid	HMDB0002259	10465		CCCCCCCCCCCCCCCCC(=O)O
15	HMDB0000944	Behenic acid	HMDB0000944	8215	C08281	CCCCCCCCCCCCCCCCCCCCCC(=O)O
16	HMDB0011131	MG(18:0/0:0/0:0)	HMDB0011131	15560610		CCCCCCCCCCCCCCCCCC(=O)OC[C@H](CO
17	HMDB0000149	Ethanolamine	HMDB0000149	700	C00189	C(CO)N
18	HMDB0002931	N-Acetylserine	HMDB0002931	65249		CC(=O)N[C@@H](CO)C(=O)O
19	HMDB0002894	5-Methylcytosine	HMDB0002894	65040	C02376	CC1=C(NC(=O)N=C1)N
20	HMDB0000660	D-Fructose	HMDB0000660	439709	C02336	C([C@@H]1[C@H]([C@@H]([C@](O1)(CO)O)O)
21	HMDB0000122	D-Glucose	HMDB0000122	5793	C00031	C([C@@H]1[C@H]([C@@H]([C@H](C(O1)O)O)O
22	HMDB0000169	D-Mannose	HMDB0000169	18950	C00159	C([C@@H]1[C@H]([C@@H]([C@@H](C(O1)O)O)
23	HMDB0000143	D-Galactose	HMDB0000143	439357	C00984	C([C@@H]1[C@@H]([C@@H]([C@H]([C@H](O1)
24	HMDB0000211	myo-Inositol	HMDB0000211		C00137	O[C@H]1[C@H](O)[C@@H](O)[C@H](O)[C@H](
25	HMDB0000258	Sucrose	HMDB0000258	5988	C00089	C([C@@H]1[C@H]([C@@H]([C@H]([C@H](O1)O
26	HMDB0061711	NA	NA	NA	NA	NA
27	HMDB0000254	Succinic acid	HMDB0000254	1110	C00042	C(CC(=O)O)C(=O)O
28	HMDB0002107	Phthalic acid	HMDB0002107	1017	C01606	C1=CC=C(C(=C1)C(=O)O)C(=O)O
29	HMDB0094660	NA	NA	NA	NA	NA
30	HMDB0000673	Linoleic acid	HMDB0000673	5280450	C01595	CCCCC/C=C\C/C=C\CCCCCCCC(=O)O
31	HMDB0011537	MG(0:0/18:1(9Z)/0:0)	HMDB0011537	5319879		CCCCCCCC/C=C\CCCCCCCC(=O)OC(CO)
32	HMDB0000126	Glycerol 3-phosphate	HMDB0000126	439162	C00093	C([C@H](COP(=O)(O)O)O)O

4 Pathway Analysis

In this step, users are asked to select a pathway library, as well as specify the algorithms for pathway enrichment analysis and pathway topology analysis.

4.1 Pathway Library

There are 15 pathway libraries currently supported, with a total of 1173 pathways :

- Homo sapiens (human) [80]
- Mus musculus (mouse) [82]
- Rattus norvegicus (rat) [81]
- Bos taurus (cow) [81]
- Danio rerio (zebrafish) [81]
- Drosophila melanogaster (fruit fly) [79]
- Caenorhabditis elegans (nematode) [78]
- Saccharomyces cerevisiae (yeast) [65]
- Oryza sativa japonica (Japanese rice) [83]
- Arabidopsis thaliana (thale cress) [87]
- Escherichia coli K-12 MG1655 [87]
- Bacillus subtilis [80]
- Pseudomonas putida KT2440 [89]
- Staphylococcus aureus N315 (MRSA/VSSA)[73]
- Thermotoga maritima [57]

Your selected pathway library code is **ath** (KEGG organisms abbreviation).

4.2 Pathway Enrichment Analysis

Pathway enrichment analysis usually refers to quantitative enrichment analysis directly using the compound concentration values, as compared to compound lists used by over-representation analysis. As a result, it is more sensitive and has the potential to identify **subtle but consistent** changes amongst compounds involved in the same biological pathway.

Many procedures have been developed in the last decade for quantitative enrichment analysis, the most famous being the Gene Set Enrichment Analysis. Many new and improved methods have been implemented since. The enrichment analysis is based on GlobalTest and GlobalAncova. Both methods support enrichment analysis with binary, multi-group, as well as continuous phenotypes. The p-values can be approximated based on the asymptotic distribution without using permutations which is computationally very intensive and is not suitable for web applications. Please note, when sample sizes are small, the approximated p values may be slightly less accurate compared to p values obtained by using a permutation-based method (for details, please refer to the paper by Goeman, J.J. et al. [1] and by

[1] Jelle J. Goeman and Peter Buhlmann. *Analyzing gene expression data in terms of gene sets: methodological issues*, Bioinformatics 2007 23(8):980-987

Hummel, M. et al. [2]) However, since our focus is to identify the most relevant pathways within the pathways in the library, we are more interested in the rank of the pathway, not its absolute p-value. Therefore, this disadvantage may be tolerated.

The selected pathway enrichment analysis method is **Globaltest**.

4.3 Pathway Topology Analysis

The structure of biological pathways represent our knowledge about the complex relationships among molecules within a cell or a living organism. However, most pathway analysis algorithms fail to take structural information into consideration when estimating which pathways are significantly changed under conditions of study. It is well-known that changes in more important positions of a network will trigger a more severe impact on the pathway than changes occurred in marginal or relatively isolated positions.

The pathway topology analysis uses two well-established node centrality measures to estimate node importance - **degree centrality** and **betweenness centrality**. Degree centrality is defined as the number of links occurred upon a node. For a directed graph there are two types of degree: in-degree for links come from other nodes, and out-degree for links initiated from the current node. Metabolic networks are directed graph. Here we only consider the out-degree for node importance measure. It is assumed that nodes upstream will have regulatory roles for the downstream nodes, not vice versa. The betweenness centrality measures the number of shortest paths going through the node. Since the metabolic network is directed, we use the relative betweenness centrality for a metabolite as the importance measure. The degree centrality measure focuses more on local connectivities, while the betweenness centrality measure focuses more on global network topology. For more detailed discussions on various graph-based methods for analyzing biological networks, please refer to the article by Tero Aittokallio, T. et al. [3]

Please note, for comparison among different pathways, the node importance values calculated from centrality measures are further normalized by the sum of the importance of the pathway. Therefore, the total/maximum importance of each pathway is 1; the importance measure of each metabolite node is actually the percentage w.r.t the total pathway importance, and the pathway impact value is the cumulative percentage from the matched metabolite nodes.

Your selected node importance measure for topological analysis is **relative betweenness centrality**.

5 Pathway Analysis Result

The results from pathway analysis are presented graphically as well as in a detailed table.

A Google-map style interactive visualization system was implemented to facilitate data exploration. The graphical output contains three levels of view: **metabolome view**, **pathway view**, and **compound view**. Only the metabolome view is shown below. Pathway views and compound views are generated dynamically based on your interactions with the visualization system. They are available in your downloaded files.

[2]Manuela Hummel, Reinhard Meister and Ulrich Mansmann. *GlobalANCOVA: exploration and assessment of gene group effects*, Bioinformatics 2008 24(1):78-85

[3]Tero Aittokallio and Benno Schwikowski. *Graph-based methods for analyzing networks in cell biology*, Briefings in Bioinformatics 2006 7(3):243-255

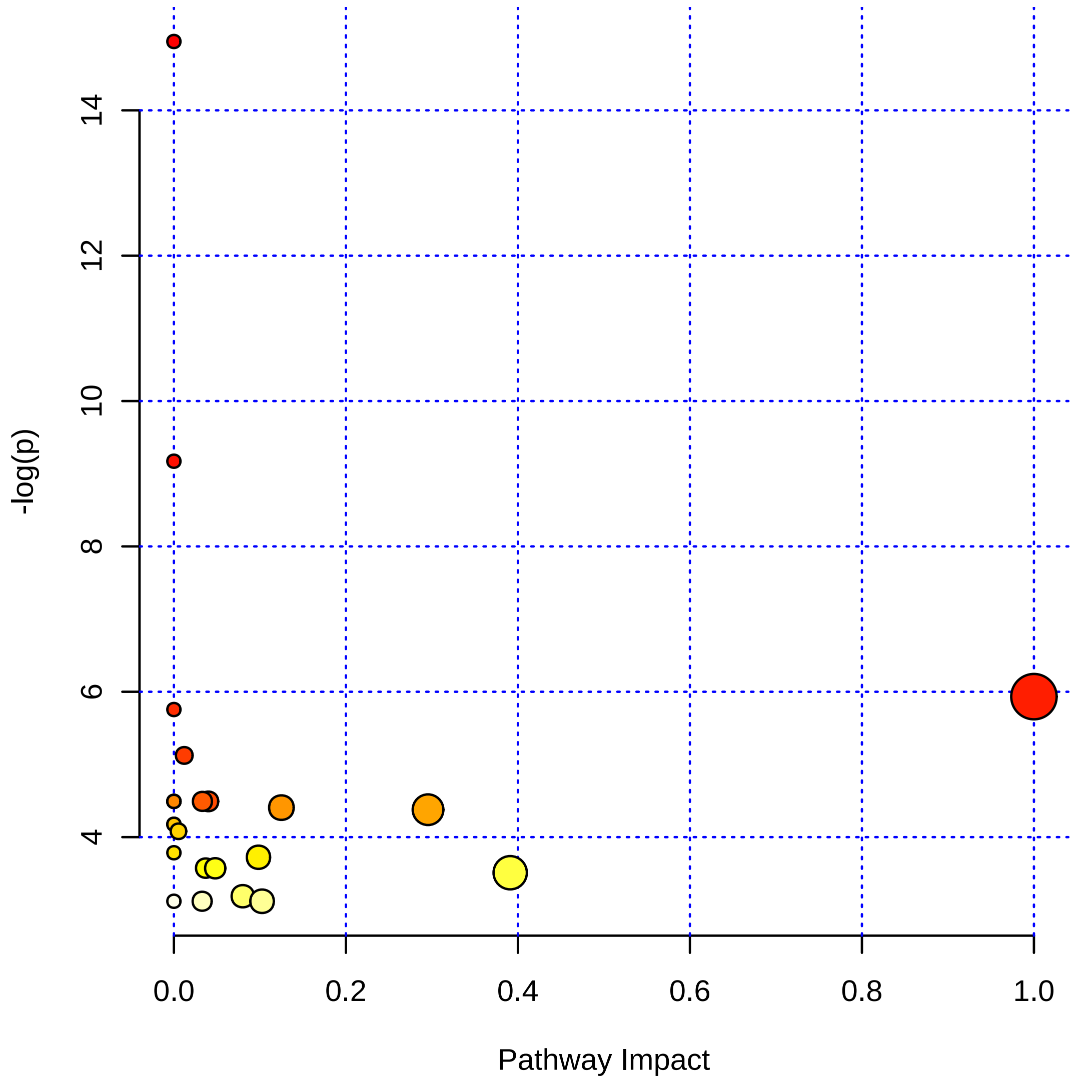

Figure 1: Summary of Pathway Analysis

The table below shows the detailed results from the pathway analysis. Since we are testing many pathways at the same time, the statistical p values from enrichment analysis are further adjusted for multiple testings. In particular, the **Total** is the total number of compounds in the pathway; the **Hits** is the actually matched number from the user uploaded data; the **Raw p** is the original p value calculated from the enrichment analysis; the **Holm p** is the p value adjusted by Holm-Bonferroni method; the **FDR p** is the p value adjusted using False Discovery Rate; the **Impact** is the pathway impact value calculated from pathway topology analysis.

Table 2: Result from Pathway Analysis

	Total Cmpd	Hits	Raw p	-log(p)	Holm adjust	FDR	Impact
Biosynthesis of unsaturated fatty acids	22	4	3.22E-07	1.49E+01	7.74E-06	7.74E-06	0.00
Fatty acid biosynthesis	56	4	1.04E-04	9.17E+00	2.39E-03	1.25E-03	0.00
Linoleic acid metabolism	4	1	2.65E-03	5.93E+00	5.83E-02	1.90E-02	1.00
Pentose phosphate pathway	19	1	3.16E-03	5.76E+00	6.65E-02	1.90E-02	0.00
Glutathione metabolism	26	1	5.95E-03	5.12E+00	1.19E-01	2.51E-02	0.01
Citrate cycle (TCA cycle)	20	1	1.12E-02	4.49E+00	2.13E-01	2.51E-02	0.04
Sulfur metabolism	15	1	1.12E-02	4.49E+00	2.13E-01	2.51E-02	0.03
Alanine, aspartate and glutamate metabolism	22	1	1.12E-02	4.49E+00	2.13E-01	2.51E-02	0.00
Propanoate metabolism	20	1	1.12E-02	4.49E+00	2.13E-01	2.51E-02	0.00
Butanoate metabolism	17	1	1.12E-02	4.49E+00	2.13E-01	2.51E-02	0.00
Cutin, suberine and wax biosynthesis	18	2	1.22E-02	4.41E+00	2.13E-01	2.51E-02	0.12
Glyoxylate and dicarboxylate metabolism	29	2	1.26E-02	4.38E+00	2.13E-01	2.51E-02	0.30
Purine metabolism	63	1	1.54E-02	4.18E+00	2.13E-01	2.63E-02	0.00
Glycine, serine and threonine metabolism	33	1	1.54E-02	4.18E+00	2.13E-01	2.63E-02	0.00
Glycerolipid metabolism	21	1	1.69E-02	4.08E+00	2.13E-01	2.70E-02	0.01
Amino sugar and nucleotide sugar metabolism	50	2	2.27E-02	3.79E+00	2.13E-01	3.41E-02	0.00
Glycerophospholipid metabolism	37	2	2.42E-02	3.72E+00	2.13E-01	3.41E-02	0.10
Fructose and mannose metabolism	20	1	2.81E-02	3.57E+00	2.13E-01	3.55E-02	0.04
Galactose metabolism	27	5	2.81E-02	3.57E+00	2.13E-01	3.55E-02	0.05
Starch and sucrose metabolism	22	2	2.99E-02	3.51E+00	2.13E-01	3.59E-02	0.39
Phenylalanine, tyrosine and tryptophan biosynthesis	22	1	4.13E-02	3.19E+00	2.13E-01	4.43E-02	0.08
Inositol phosphate metabolism	28	1	4.43E-02	3.12E+00	2.13E-01	4.43E-02	0.10
Phosphatidylinositol signaling system	26	1	4.43E-02	3.12E+00	2.13E-01	4.43E-02	0.03
Ascorbate and aldarate metabolism	18	1	4.43E-02	3.12E+00	2.13E-01	4.43E-02	0.00

6 Appendix: R Command History

```
 [1] "mSet<-InitDataObjects(\"conc\", \"pathqea\", FALSE)"
 [2] "mSet<-Read.TextData(mSet, \"Replacing_with_your_file_path\", \"rowu\", \"disc\");"
 [3] "mSet<-CrossReferencing(mSet, \"hmdb\");"
 [4] "mSet<-CreateMappingResultTable(mSet)"
 [5] "mSet<-SanityCheckData(mSet)"
 [6] "mSet<-ImputeVar(mSet, method=\"min\")"
 [7] "mSet<-PreparePrenormData(mSet)"
 [8] "mSet<-Normalization(mSet, \"NULL\", \"NULL\", \"RangeNorm\", ratio=FALSE, ratioNum=20)"
 [9] "mSet<-PlotNormSummary(mSet, \"norm_0_\", \"png\", 72, width=NA)"
[10] "mSet<-PlotSampleNormSummary(mSet, \"snorm_0_\", \"png\", 72, width=NA)"
[11] "mSet<-SetKEGG.PathLib(mSet, \"ath\", \"current\")"
[12] "mSet<-SetMetabolomeFilter(mSet, F);"
[13] "mSet<-CalculateQeaScore(mSet, \"rbc\", \"gt\")"
[14] "mSet<-PlotPathSummary(mSet, \"path_view_0_\", \"png\", 72, width=NA)"
[15] "mSet<-SaveTransformedData(mSet)"
[16] "mSet<-PreparePDFReport(mSet, \"guest447328871261268466\")\n"
```

The report was generated on Sun Mar 22 10:01:51 2020 with R version 3.6.3 (2020-02-29).
