# Supplementary material for: Metabolic Responses of Amaranthus caudatus Roots and Leaves to Zinc Stress
Source: Plants (Basel). 2025 Jul 9;14(14):2119. doi: 10.3390/plants14142119 (PMC12300844; doi:10.3390/plants14142119)
Supplement: Supplementary file 1 [file plants-14-02119-s001.zip › Osmolovskaya_et_al_Supplementary_S4.3_Mature_leaves_PathwayAnalysis_Report.pdf]

Metabolomic Data Analysis with MetaboAnalyst 5.0

Name: guest17477237295990508420

February 3, 2022

1 Background

The Pathway Analysis module combines results from powerful pathway enrichment analysis with pathway topology analysis to help researchers identify the most relevant pathways involved in the conditions under study.

There are many commercial pathway analysis software tools such as Pathway Studio, MetaCore, or Ingenuity Pathway Analysis (IPA), etc. Compared to these commercial tools, the pathway analysis module was specifically developed for metabolomics studies. It uses high-quality KEGG metabolic pathways as the backend knowledgebase. This module integrates many well-established (i.e. univariate analysis, over-representation analysis) methods, as well as novel algorithms and concepts (i.e. Global Test, GlobalAncova, network topology analysis) into pathway analysis. Another feature is a Google-Map style interactive visualization system to deliver the analysis results in an intuitive manner.

2 Data Input

The Pathway Analysis module accepts either a list of compound labels (common names, HMDB IDs or KEGG IDs) with one compound per row, or a compound concentration table with samples in rows and compounds in columns. The second column must be phenotype labels (binary, multi-group, or continuous). The table is uploaded as comma separated values (.csv).

3 Compound Name Matching

The first step is to standardize the compound labels used in user uploaded data. This is a necessary step since these compounds will be subsequently compared with compounds contained in the pathway library. There are three outcomes from the step - exact match, approximate match (for common names only), and no match. Users should click the textbfView button from the approximate matched results to manually select the correct one. Compounds without match will be excluded from the subsequently pathway analysis.

Table 1 shows the conversion results. Note: *1* indicates exact match, *2* indicates approximate match, and *0* indicates no match. A text file contain the result can be found the downloaded file *name_map.csv*

Table 1: Result from Compound Name Mapping

	Query	Match	HMDB	PubChem	KEGG	SMILES
1	HMDB0000156	L-Malic acid	HMDB0000156	222656	C00149	C([C@@H](C(=O)O)O)C(=O)O
2	HMDB0000613	Erythronic acid	HMDB0000613	2781043		C([C@H]([C@H](C(=O)O)O)O)O
3	HMDB0000337	(S)-3,4-Dihydroxybutyric acid	HMDB0000337	150929		C(C(CO)O)C(=O)O
4	HMDB0000150	Gluconolactone	HMDB0000150	7027	C00198	C([C@@H]1[C@H]([C@@H]([C@H](C(=O)O1)[illegible]
5	HMDB0001895	Salicylic acid	HMDB0001895	338	C00805	C1=CC=C(C(=C1)C(=O)O)O
6	HMDB0030677	cis-p-Coumaric acid	HMDB0030677	1549106	C06738	C1=CC(=CC=C1/C=C\C(=O)O)O
7	HMDB0002641	2-Hydroxycinnamic acid	HMDB0002641	637540	C01772	C1=CC=C(C(=C1)/C=C/C(=O)O)O
8	HMDB0000424	2-Hydroxydecanedioic acid	HMDB0000424	128458		C(CCCC(C(=O)O)O)CCCC(=O)O
9	HMDB0250603	NA	NA	NA	NA	NA

10	HMDB0000092	Dimethylglycine	HMDB0000092	673	C01026	CN(C)CC(=O)O
11	HMDB0000133	Guanosine	HMDB0000133	6802	C00387	C1=NC2=C(N1[C@H]3[C@@H]([C@@H]([C@H
12	HMDB0002894	5-Methylcytosine	HMDB0002894	65040	C02376	CC1=C(NC(=O)N=C1)N
13	HMDB0000660	D-Fructose	HMDB0000660	439709	C02336	C([C@@H]1[C@H]([C@@H]([C@](O1)(CO)O)O
14	HMDB0003449	Beta-D-Galactose	HMDB0003449	439353	C00962	C([C@@H]1[C@@H]([C@@H]([C@H]([C@@H](O
15	HMDB0000143	D-Galactose	HMDB0000143	439357	C00984	C([C@@H]1[C@@H]([C@@H]([C@H]([C@H](O1
16	HMDB0000143.1	NA	NA	NA	NA	NA
17	HMDB0304632	NA	NA	NA	NA	NA
18	HMDB0003345	Alpha-D-Glucose	HMDB0003345	79025	C00267	C([C@@H]1[C@H]([C@@H]([C@H]([C@H](O1)O
19	HMDB0000122	D-Glucose	HMDB0000122	5793	C00221	C([C@@H]1[C@H]([C@@H]([C@H](C(O1)O)O)
20	HMDB0000258	Sucrose	HMDB0000258	5988	C00089	C([C@@H]1[C@H]([C@@H]([C@H]([C@H](O1)O
21	HMDB0304157	NA	NA	NA	NA	NA
22	HMDB0000254	Succinic acid	HMDB0000254	1110	C00042	C(CC(=O)O)C(=O)O
23	HMDB0000126	Glycerol 3-phosphate	HMDB0000126	439162	C00093	C([C@H](COP(=O)(O)O)O)O

4 Pathway Analysis

In this step, users are asked to select a pathway library, as well as specify the algorithms for pathway enrichment analysis and pathway topology analysis.

4.1 Pathway Library

There are 15 pathway libraries currently supported, with a total of 1173 pathways :

- Homo sapiens (human) [80]
- Mus musculus (mouse) [82]
- Rattus norvegicus (rat) [81]
- Bos taurus (cow) [81]
- Danio rerio (zebrafish) [81]
- Drosophila melanogaster (fruit fly) [79]
- Caenorhabditis elegans (nematode) [78]
- Saccharomyces cerevisiae (yeast) [65]
- Oryza sativa japonica (Japanese rice) [83]
- Arabidopsis thaliana (thale cress) [87]
- Escherichia coli K-12 MG1655 [87]
- Bacillus subtilis [80]
- Pseudomonas putida KT2440 [89]
- Staphylococcus aureus N315 (MRSA/VSSA)[73]
- Thermotoga maritima [57]

Your selected pathway library code is **ath** (KEGG organisms abbreviation).

4.2 Pathway Enrichment Analysis

Pathway enrichment analysis usually refers to quantitative enrichment analysis directly using the compound concentration values, as compared to compound lists used by over-representation analysis. As a result, it is more sensitive and has the potential to identify **subtle but consistent** changes amongst compounds involved in the same biological pathway.

Many procedures have been developed in the last decade for quantitative enrichment analysis, the most famous being the Gene Set Enrichment Analysis. Many new and improved methods have been implemented since. The enrichment analysis is based on GlobalTest and GlobalAncova. Both methods support enrichment analysis with binary, multi-group, as well as continuous phenotypes. The p-values can be approximated based on the asymptotic distribution without using permutations which is computationally very intensive and is not suitable for web applications. Please note, when sample sizes are small, the approximated p values may be slightly less accurate compared to p values obtained by using a permutation-based method (for details, please refer to the paper by Goeman, J.J. et al. [1] and by

[1] Jelle J. Goeman and Peter Buhlmann. *Analyzing gene expression data in terms of gene sets: methodological issues*, Bioinformatics 2007 23(8):980-987

Hummel, M. et al. [2]) However, since our focus is to identify the most relevant pathways within the pathways in the library, we are more interested in the rank of the pathway, not its absolute p-value. Therefore, this disadvantage may be tolerated.

The selected pathway enrichment analysis method is **Globaltest**.

4.3 Pathway Topology Analysis

The structure of biological pathways represent our knowledge about the complex relationships among molecules within a cell or a living organism. However, most pathway analysis algorithms fail to take structural information into consideration when estimating which pathways are significantly changed under conditions of study. It is well-known that changes in more important positions of a network will trigger a more severe impact on the pathway than changes occurred in marginal or relatively isolated positions.

The pathway topology analysis uses two well-established node centrality measures to estimate node importance - **degree centrality** and **betweenness centrality**. Degree centrality is defined as the number of links occurred upon a node. For a directed graph there are two types of degree: in-degree for links come from other nodes, and out-degree for links initiated from the current node. Metabolic networks are directed graph. Here we only consider the out-degree for node importance measure. It is assumed that nodes upstream will have regulatory roles for the downstream nodes, not vice versa. The betweenness centrality measures the number of shortest paths going through the node. Since the metabolic network is directed, we use the relative betweenness centrality for a metabolite as the importance measure. The degree centrality measure focuses more on local connectivities, while the betweenness centrality measure focuses more on global network topology. For more detailed discussions on various graph-based methods for analyzing biological networks, please refer to the article by Tero Aittokallio, T. et al. [3]

Please note, for comparison among different pathways, the node importance values calculated from centrality measures are further normalized by the sum of the importance of the pathway. Therefore, the total/maximum importance of each pathway is 1; the importance measure of each metabolite node is actually the percentage w.r.t the total pathway importance, and the pathway impact value is the cumulative percentage from the matched metabolite nodes.

Your selected node importance measure for topological analysis is **relative betweenness centrality**.

5 Pathway Analysis Result

The results from pathway analysis are presented graphically as well as in a detailed table.

A Google-map style interactive visualization system was implemented to facilitate data exploration. The graphical output contains three levels of view: **metabolome view**, **pathway view**, and **compound view**. Only the metabolome view is shown below. Pathway views and compound views are generated dynamically based on your interactions with the visualization system. They are available in your downloaded files.

[2] Manuela Hummel, Reinhard Meister and Ulrich Mansmann. *GlobalANCOVA: exploration and assessment of gene group effects*, Bioinformatics 2008 24(1):78-85

[3] Tero Aittokallio and Benno Schwikowski. *Graph-based methods for analyzing networks in cell biology*, Briefings in Bioinformatics 2006 7(3):243-255

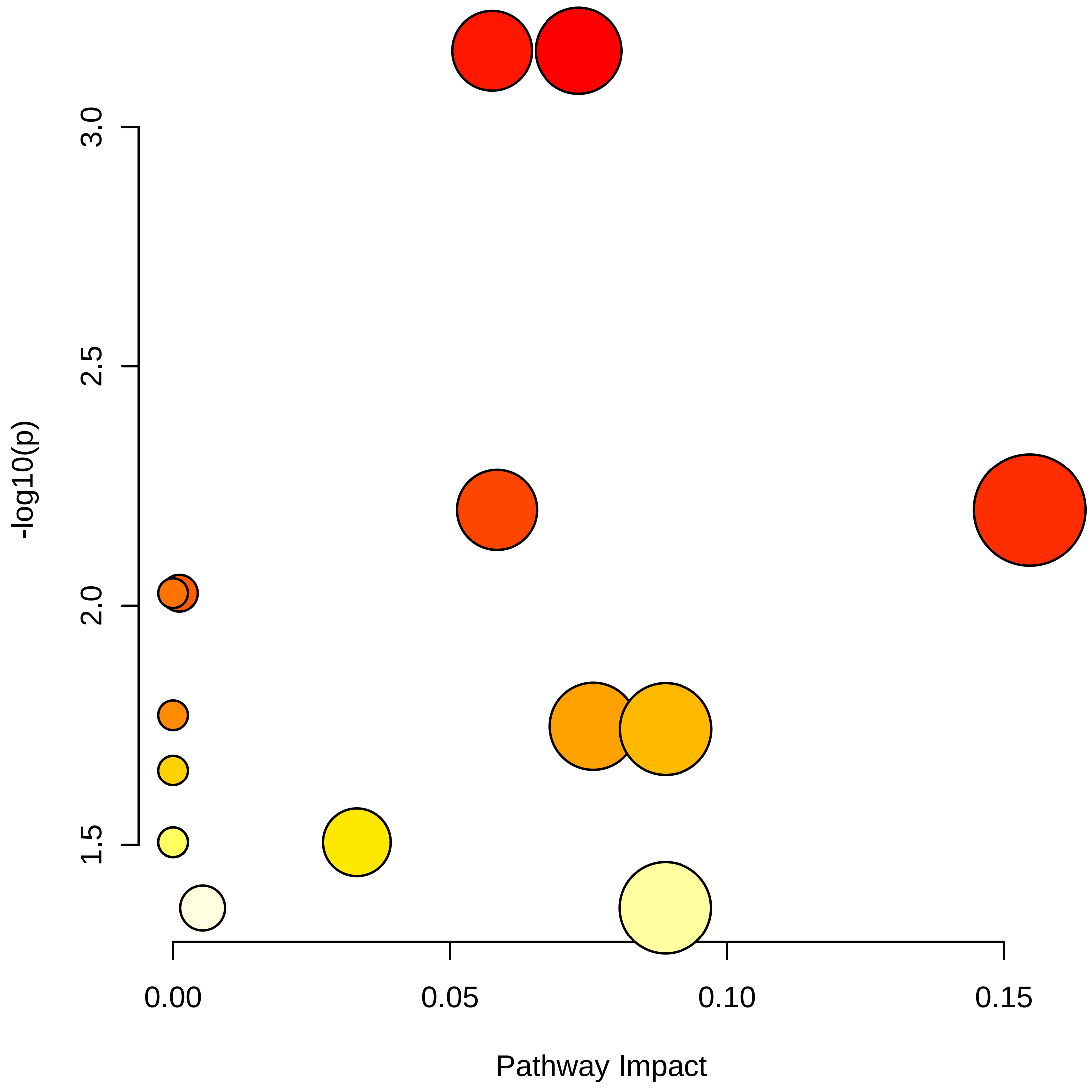

Figure 1: Summary of Pathway Analysis

The table below shows the detailed results from the pathway analysis. Since we are testing many pathways at the same time, the statistical p values from enrichment analysis are further adjusted for multiple testings. In particular, the **Total** is the total number of compounds in the pathway; the **Hits** is the actually matched number from the user uploaded data; the **Raw p** is the original p value calculated from the enrichment analysis; the **Holm p** is the p value adjusted by Holm-Bonferroni method; the **FDR p** is the p value adjusted using False Discovery Rate; the **Impact** is the pathway impact value calculated from pathway topology analysis.

Table 2: Result from Pathway Analysis

	Total Cmpd	Hits	Raw p	-log10(p)	Holm adjust	FDR	Impact
Citrate cycle (TCA cycle)	20	2	6.94E-04	3.16E+00	1.11E-02	5.55E-03	0.07
Glyoxylate and dicarboxylate metabolism	29	2	6.94E-04	3.16E+00	1.11E-02	5.55E-03	0.06
Pyruvate metabolism	22	1	6.31E-03	2.20E+00	8.84E-02	2.51E-02	0.15
Carbon fixation in photosynthetic organisms	21	1	6.31E-03	2.20E+00	8.84E-02	2.51E-02	0.06
Glycolysis / Gluconeogenesis	26	2	9.41E-03	2.03E+00	1.13E-01	2.51E-02	0.00
Fructose and mannose metabolism	20	1	9.41E-03	2.03E+00	1.13E-01	2.51E-02	0.00
Purine metabolism	63	1	1.69E-02	1.77E+00	1.69E-01	3.22E-02	0.00
Galactose metabolism	27	3	1.79E-02	1.75E+00	1.69E-01	3.22E-02	0.08
Starch and sucrose metabolism	22	1	1.81E-02	1.74E+00	1.69E-01	3.22E-02	0.09
Amino sugar and nucleotide sugar metabolism	50	2	2.21E-02	1.66E+00	1.69E-01	3.54E-02	0.00
Sulfur metabolism	15	1	3.12E-02	1.51E+00	1.87E-01	3.57E-02	0.03
Alanine, aspartate and glutamate metabolism	22	1	3.12E-02	1.51E+00	1.87E-01	3.57E-02	0.00
Propanoate metabolism	20	1	3.12E-02	1.51E+00	1.87E-01	3.57E-02	0.00
Butanoate metabolism	17	1	3.12E-02	1.51E+00	1.87E-01	3.57E-02	0.00
Glycerophospholipid metabolism	37	1	4.28E-02	1.37E+00	1.87E-01	4.28E-02	0.09
Glycerolipid metabolism	21	1	4.28E-02	1.37E+00	1.87E-01	4.28E-02	0.01

6 Appendix: R Command History

```
 [1] "mSet<-InitDataObjects(\"conc\", \"pathqea\", FALSE)"
 [2] "mSet<-Read.TextData(mSet, \"Replacing_with_your_file_path\", \"rowu\", \"disc\");"
 [3] "mSet<-SanityCheckData(mSet)"
 [4] "mSet<-ReplaceMin(mSet);"
 [5] "mSet<-CrossReferencing(mSet, \"hmdb\");"
 [6] "mSet<-CreateMappingResultTable(mSet)"
 [7] "mSet<-PreparePrenormData(mSet)"
 [8] "mSet<-GetGroupNames(mSet, \"\")"
 [9] "feature.nm.vec <- c(\"\")"
[10] "smpl.nm.vec <- c(\"LM_Zn_2\")"
[11] "grp.nm.vec <- c(\"\")"
[12] "mSet<-UpdateData(mSet)"
[13] "mSet<-PreparePrenormData(mSet)"
[14] "mSet<-Normalization(mSet, \"NULL\", \"NULL\", \"ParetoNorm\", ratio=FALSE, ratioNum=20)"
[15] "mSet<-PlotNormSummary(mSet, \"norm_0_\", \"png\", 72, width=NA)"
[16] "mSet<-PlotSampleNormSummary(mSet, \"snorm_0_\", \"png\", 72, width=NA)"
[17] "mSet<-SetKEGG.PathLib(mSet, \"ath\", \"current\")"
[18] "mSet<-SetMetabolomeFilter(mSet, F);"
[19] "mSet<-CalculateQeaScore(mSet, \"rbc\", \"gt\")"
[20] "mSet<-PlotPathSummary(mSet, F, \"path_view_0_\", \"png\", 72, width=NA, NA, NA )"
[21] "mSet<-SaveTransformedData(mSet)"
[22] "mSet<-PreparePDFReport(mSet, \"guest17477237295990508420\")\n"
```

The report was generated on Thu Feb 3 06:15:06 2022 with R version 4.0.2 (2020-06-22).
