# Supplementary material for: Metabolic Responses of Amaranthus caudatus Roots and Leaves to Zinc Stress
Source: Plants (Basel). 2025 Jul 9;14(14):2119. doi: 10.3390/plants14142119 (PMC12300844; doi:10.3390/plants14142119)
Supplement: Supplementary file 1 [file plants-14-02119-s001.zip › plants-3701072 Osmolovskaya_et_al_Supplementary_S3_(Pathway schemes).pdf]

Metabolic responses of *Amaranthus caudatus* roots and leaves to zinc stress

Natalia Osmolovskaya [1,†,*], Tatiana Bilova [1,2,†], Anastasia Gurina [1], Anastasia Orlova [2], Viet D. Vu [1,3], Stanislav Sukhikh [4], Tatiana Zhilkina [5], Nadezhda Frolova [2,*], Elena Tarakhovskaya [1,6], Anastasia Kamionskaya [5] and Andrej Frolov [2]

Supplementary Information 3

[1] Department of Plant Physiology and Biochemistry, St. Petersburg State University, 199034 St. Petersburg, Russia;

[2] Laboratory of Analytical Biochemistry and Biotechnology, K.A. Timiryazev Institute of Plant Physiology of the Russian Academy of Science, 127276 Moscow, Russia;

[3] Coast Branch - Vietnam Russian Tropical Center, Khanh Hoa, Nha Trang City, Vietnam

[4] Laboratory of Microbiology and Biotechnology, Immanuel Kant Baltic Federal University, 236041, Kaliningrad, Russia;

[5] Federal Research Centre Fundamentals of Biotechnology of the Russian Academy of Science, 119071 Moscow, Russia;

[6] Vavilov Institute of General Genetics, St. Petersburg Branch, Russian Academy of Sciences, 199034 St. Petersburg, Russia.

[†]These authors contributed equally to the manuscript

*Corresponding authors:

Dr. Nadezhda Frolova

Laboratory of Analytical Biochemistry and Biotechnology, K.A. Timiryazev Institute of Plant Physiology of the Russian Academy of Science, 127276 Moscow, Russia

Tel. +7 (499) 678-54-00

Email: frolovanadja@yandex.ru

Dr. Natalia Osmolovskaya

St. Petersburg State University

Department of Plant Physiology and Biochemistry, Universitetskaya nab. 7/9, 199034, St Petersburg, Russia

Tel. +7 (812) 3289695

Email: natalia_osm@mail.ru

Directory

Part 1. Metabolic pathways of Pathway Analysis for Zn-regulated metabolites in young leaves of *Amaranthus caudatus*

Figure S3.1-1. KEGG scheme 1. Biosynthesis of unsaturated fatty acids……………………………….…...4
Figure S3.1-2. KEGG scheme 2. Fatty acid biosynthesis………………………………………….……...5
Figure S3.1-3. KEGG scheme 3. Linoleic acid metabolism………………………………………………6
Figure S3.1-4. KEGG scheme 4. Pentose phosphate pathway……………………………………………7
Figure S3.1-5. KEGG scheme 5. Glutathione metabolism……………………………………………….8
Figure S3.1-6. KEGG scheme 6. Citrate cycle………………………………………………………......9
Figure S3.1-7. KEGG scheme 7. Sulfur metabolism……………………………………………………10
Figure S3.1-8. KEGG scheme 8a. Alanine, aspartate and glutamate metabolism……………………….11
Figure S3.1-9. KEGG scheme 8b. Propanoate metabolism……………………………………………..12
Figure S3.1-10. KEGG scheme 8c. Butanoate metabolism……………………………………………..13
Figure S3.1-11. KEGG scheme 9. Cutin, suberine and wax biosynthesis……………………………...14
Figure S3.1-12. KEGG scheme 10. Glyoxylate and dicarboxylate metabolism………………………..15
Figure S3.1-13. KEGG scheme 11a. Purine metabolism………………………………………………..16
Figure S3.1-14. KEGG scheme 11b. Glycine, serine and threonine metabolism…………………….....17
Figure S3.1-15. KEGG scheme 12. Glycerolipid metabolism…………………………………………..18
Figure S3.1-16. KEGG scheme 13. Amino sugar and nucleotide sugar metabolism…………………………19
Figure S3.1-17. KEGG scheme 14. Glycerophospholipid metabolism……………………………….....20
Figure S3.1-18. KEGG scheme 15. Fructose and mannose metabolism…………………………….......21
Figure S3.1-19. KEGG scheme 16. Galactose metabolism…………………………………………......22
Figure S3.1-20. KEGG scheme 17. Starch and sucrose metabolism…………………………………...23
Figure S3.1-21. KEGG scheme 18. Phe, Tyr, Trp metabolism…………………………………………24
Figure S3.1-22. KEGG scheme 19. Inositol phosphate metabolism…………………………………....25
Figure S3.1-23. KEGG scheme 20. Phosphatidylinositol signaling……………………………………26
Figure S3.1-24. KEGG scheme 21. Ascorbate and aldarate metabolism………………………………...27

Part 2. Metabolic pathways of Pathway Analysis for Zn-regulated metabolites in roots of *A. caudatus*

Figure S3.2-1. KEGG scheme 1. Glycerolipid metabolism ……………………………………………..28
Figure S3.2-2. KEGG scheme 2. Fructose and mannose metabolism…………………………………..29
Figure S3.2-3. KEGG scheme 3. Glyoxylate and dicarboxylate metabolism……………………….…30
Figure S3.2-4. KEGG scheme 4. Starch and sucrose metabolism……………………………………...31
Figure S3.2-5. KEGG scheme 5. Inositol phosphate metabolism. ……………………………………..32
Figure S3.2-6. KEGG scheme 6. Phosphatidylinositol signalling system………………..........................33
Figure S3.2-7. KEGG scheme 7. Citrate cycle (TCA Cycle). …………………………………………..34
Figure S3.2-8. KEGG scheme 8. Galactose metabolism……………………………………………….35
Figure S3.2-9. KEGG scheme 9. Amino acid and nucleotide sugar metabolism……………………….36
Figure S3.2-10. KEGG scheme 10. Pentose Phosphate pathway……………………………………….37
Figure S3.2-11. KEGG scheme 11. Glycine, serine threonine metabolism…………………………….38
Figure S3.2-12. KEGG scheme 12. Glycerophospholipid metabolism………………………………....39
Figure S3.2-13. KEGG scheme 13. Shikimate pathway………………………………………………...40
Figure S3.2-14. KEGG scheme 14. Arginine and proline metabolism………………………………….41
Figure S3.2-15. KEGG scheme 15. Sulfur metabolism…………………………………………………42
Figure S3.2-16. KEGG scheme 16a. Propanoate metabolism…………………………………………..43
Figure S3.2-17. KEGG scheme 16b. Butanoate metabolism…………………………………………....44
Figure S3.2-18. KEGG scheme 17. Aminoacyl-tRNA biosynthesis……………………………..........45
Figure S3.219. KEGG scheme 18. Ala, Asp, Glu metabolism………………………………………….46
Figure S3.2-20. KEGG scheme 19. Ascorbate and aldarate metabolism……………………………….47
Figure S3.2-21. KEGG scheme 20. Steroid biosynthesis………………………………………………..48
Figure S3.2-22. KEGG scheme 21. Pyrimidine metabolism.…………………………………………....49

Figure S3.2-23. KEGG scheme 22. Cutin, suberin and wax biosynthesis…………………………………..…50
Figure S3.2-24. KEGG scheme 23. Val, Leu, Ile biosynthesis ………………………………………………51

Part 3. Metabolic pathways of Pathway Analysis for Zn-regulated metabolites in <u>mature leaves</u> of *A. caudatus*

Figure S3.3-1. KEGG scheme 1. Citrate cycle ……………………………………………………….……....52
Figure S3.3-2. KEGG scheme 2. Glyoxylate and Dicarboxylate metabolism…………………………………53
Figure S3.3-3. KEGG scheme 3. Pyruvate metabolism…………………………………………………….....54
Figure S3.3-4. KEGG scheme 4. Carbon fixation…………………………………………………………..…55
Figure S3.3-5. KEGG scheme 5. Glycolysis/Gluconeogenesis. ………………………………….……..….56
Figure S3.3-6. KEGG scheme 6. Fructose and mannose metabolism………………….................................57
Figure S3.3-7. KEGG scheme 7. Purine metabolism……………………………………….……..……58
Figure S3.3-8. KEGG scheme 8. Galactose metabolism……………………………………..……......59
Figure S3.3-9. KEGG scheme 9. Starch and sucrose metabolism………………………………….…...…60
Figure S3.3-10. KEGG scheme 10. Amino sugar and nucleotide sugar metabolism…………………………61

Part 1. Metabolic pathways of Pathway Analysis performed for Zn-regulated metabolites in young leaves of *Amaranthus caudatus*

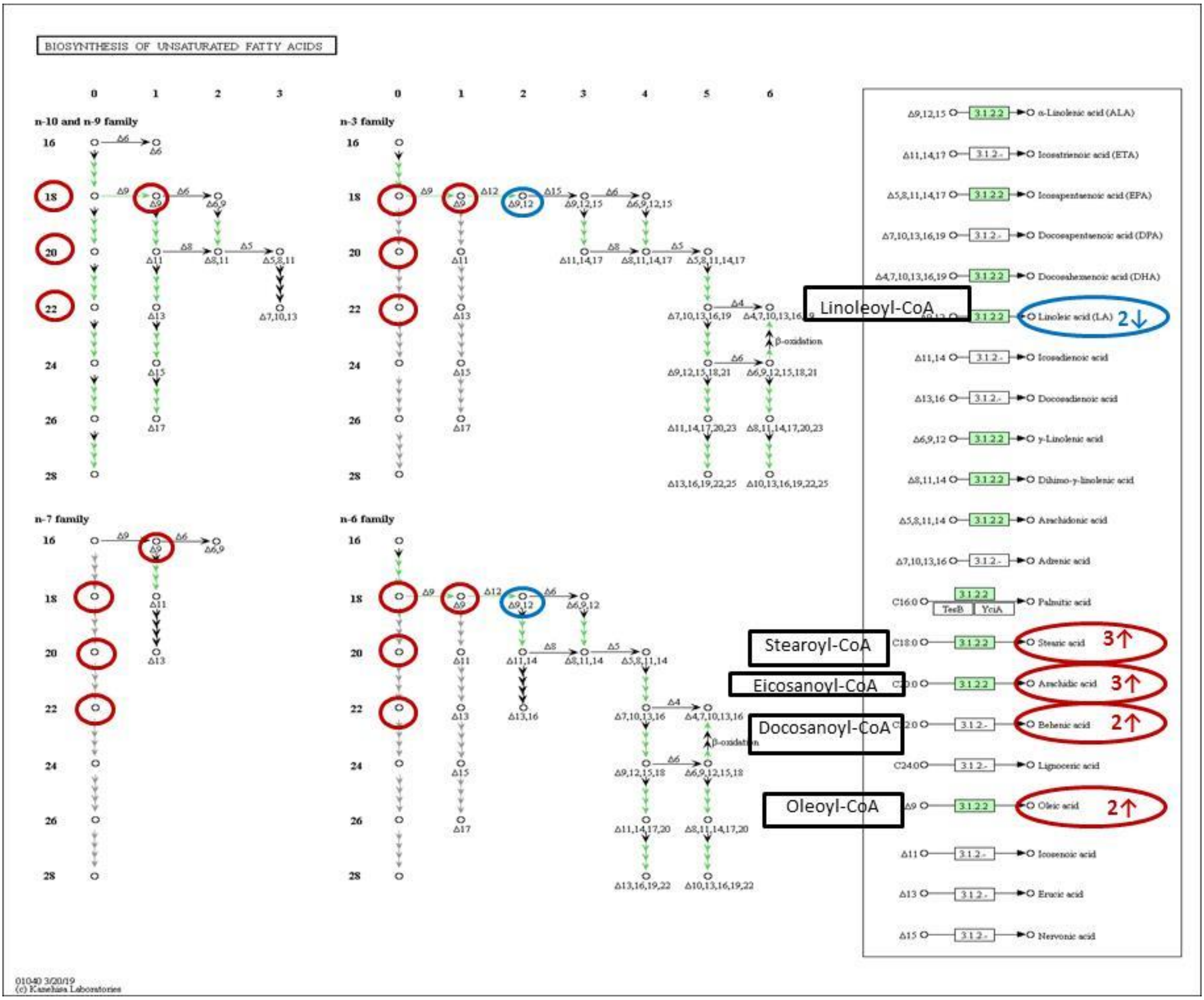

Figure S3.1-1. KEGG scheme 1. Biosynthesis of unsaturated fatty acids.

Circles mark Zn-related metabolites, red and blue circle colors denote up- and down-regulated metabolites, respectively. Value and arrow in the circles indicate fold and direction of the changes, respectively, in comparison with controls. To address Zn-related metabolites (t-test, $p \leq 0.05$) in young leaves quantified by untargeted and targeted methods refer to Tables 1 and 3, respectively.

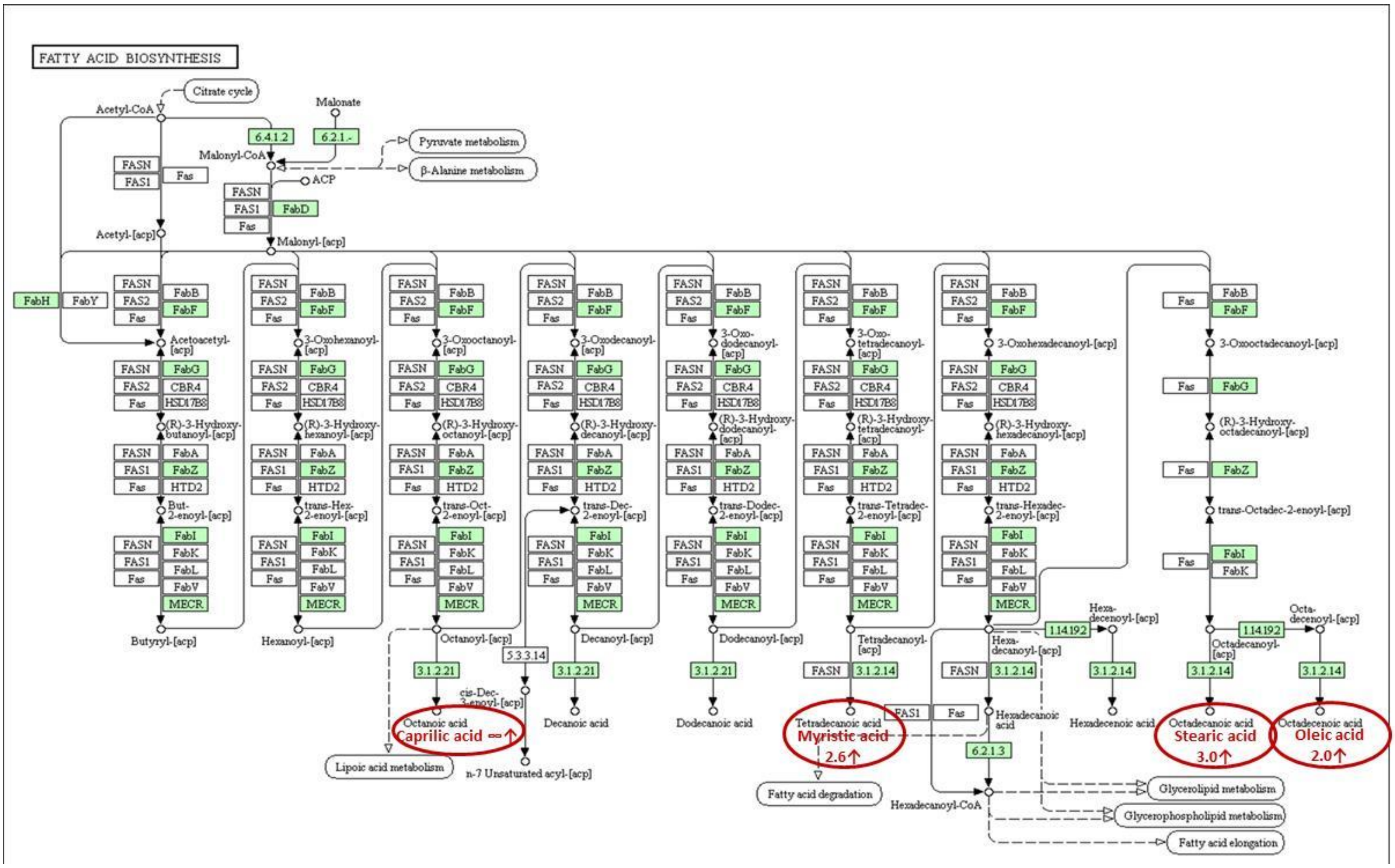

Figure S3.1-2. KEGG scheme 2. Fatty acid biosynthesis.

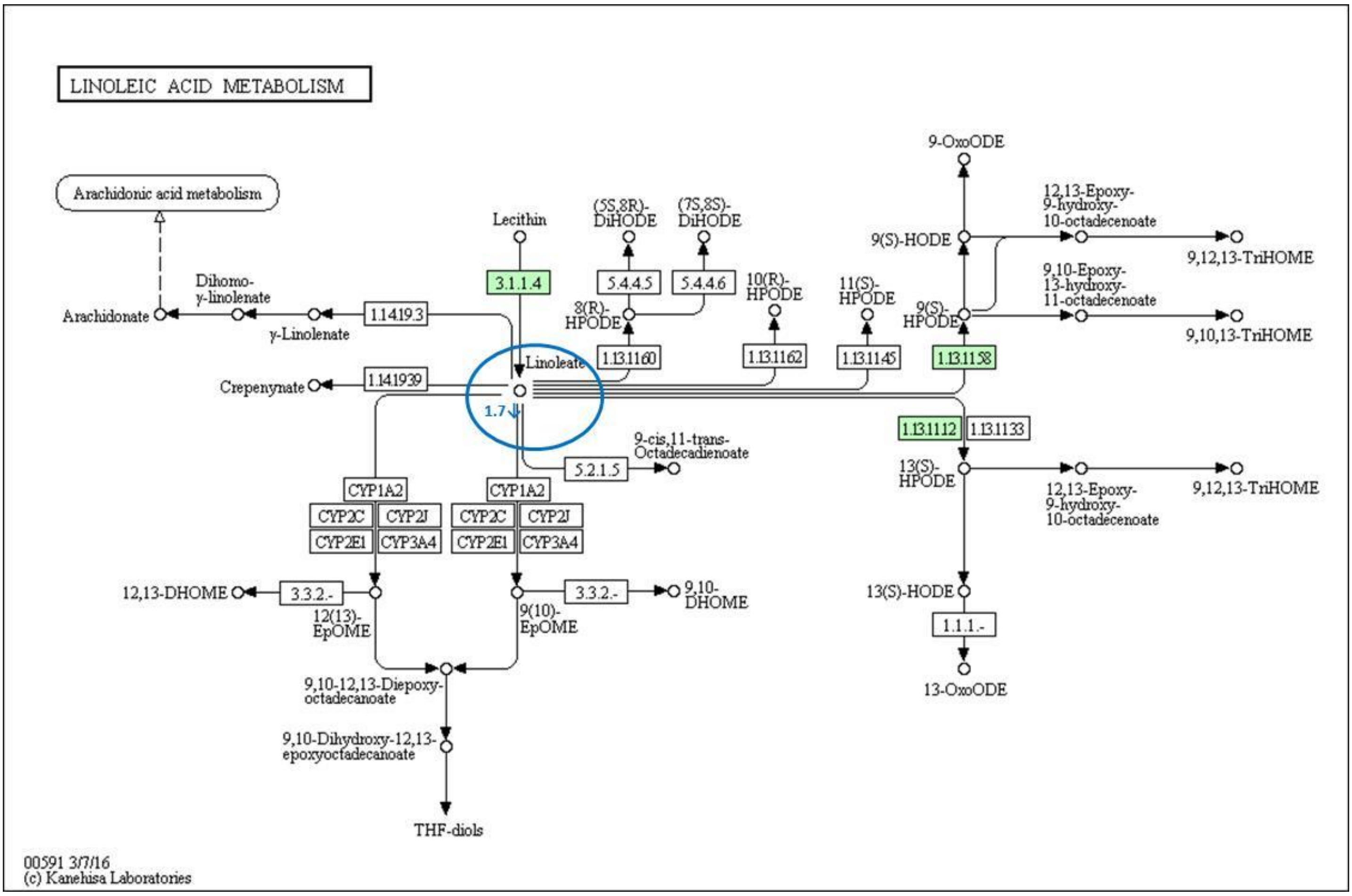

Figure S3.1-3. KEGG scheme 3. Linoleic acid metabolism.

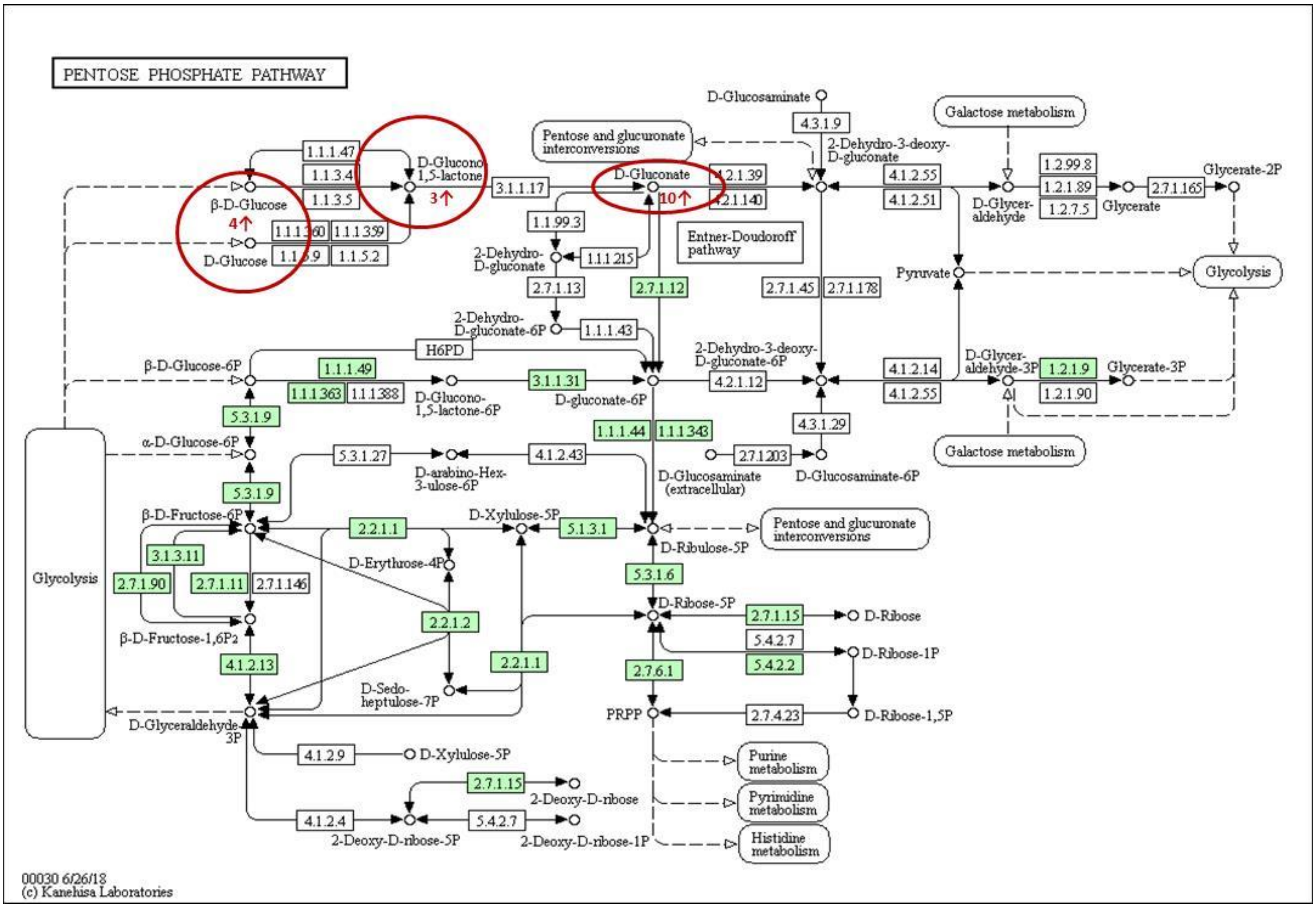

Figure S3.1-4. KEGG scheme 4. Pentose phosphate pathway.

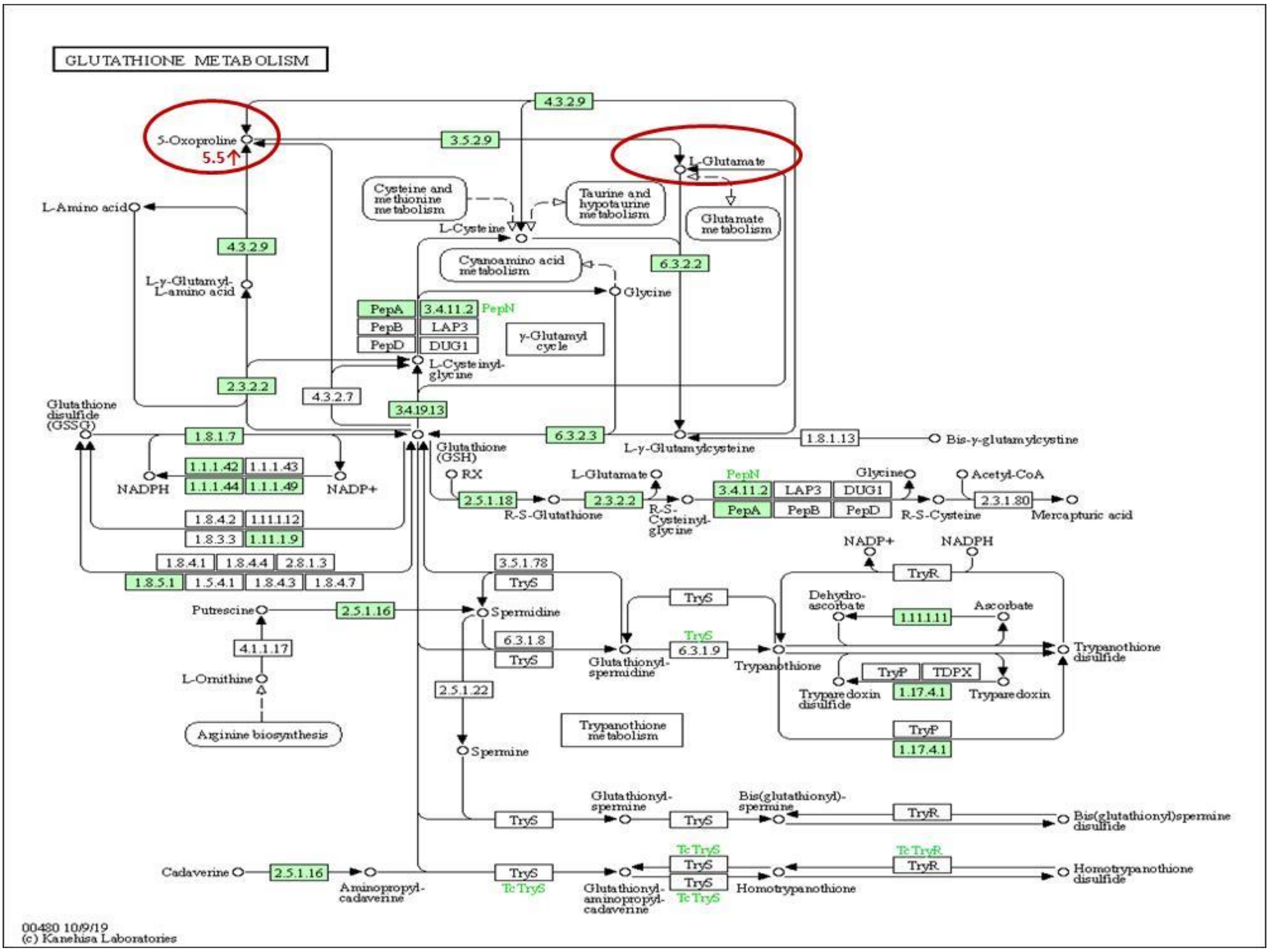

Figure S3.1-5. KEGG scheme 5. Glutathione metabolism.

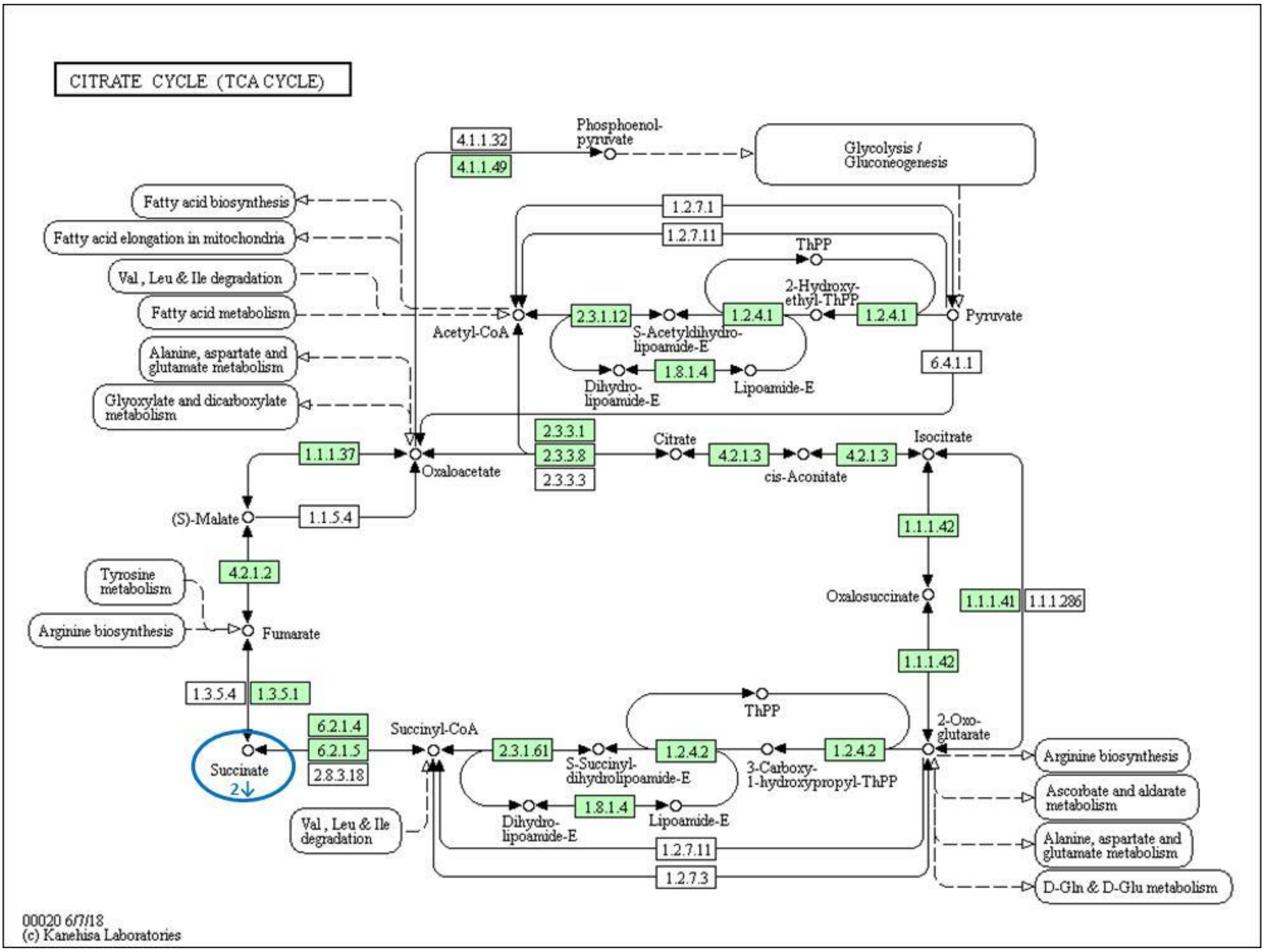

Figure S3.1-6. KEGG scheme 6. Citrate cycle.

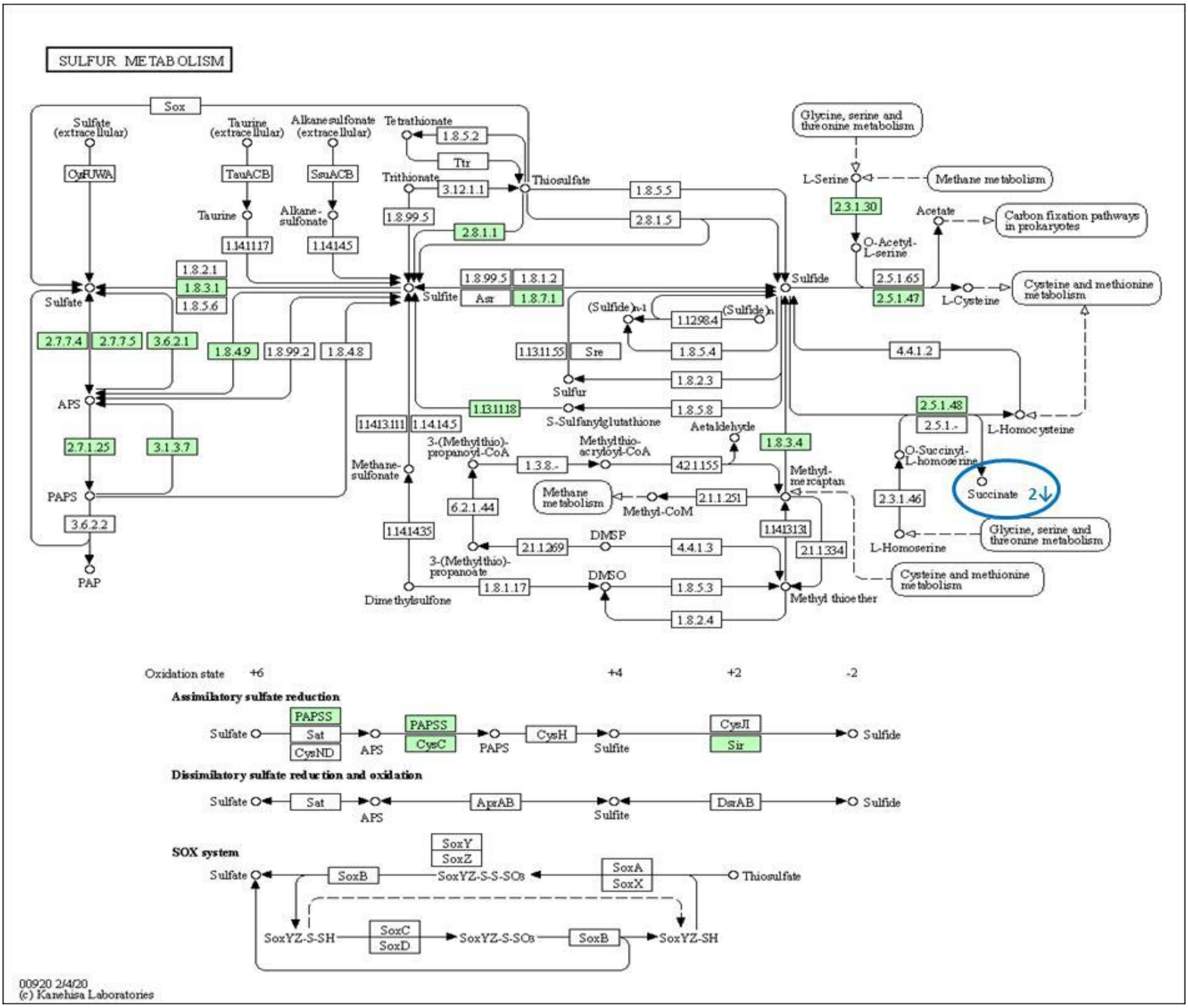

Figure S3.1-7. KEGG scheme 7. Sulfur metabolism.

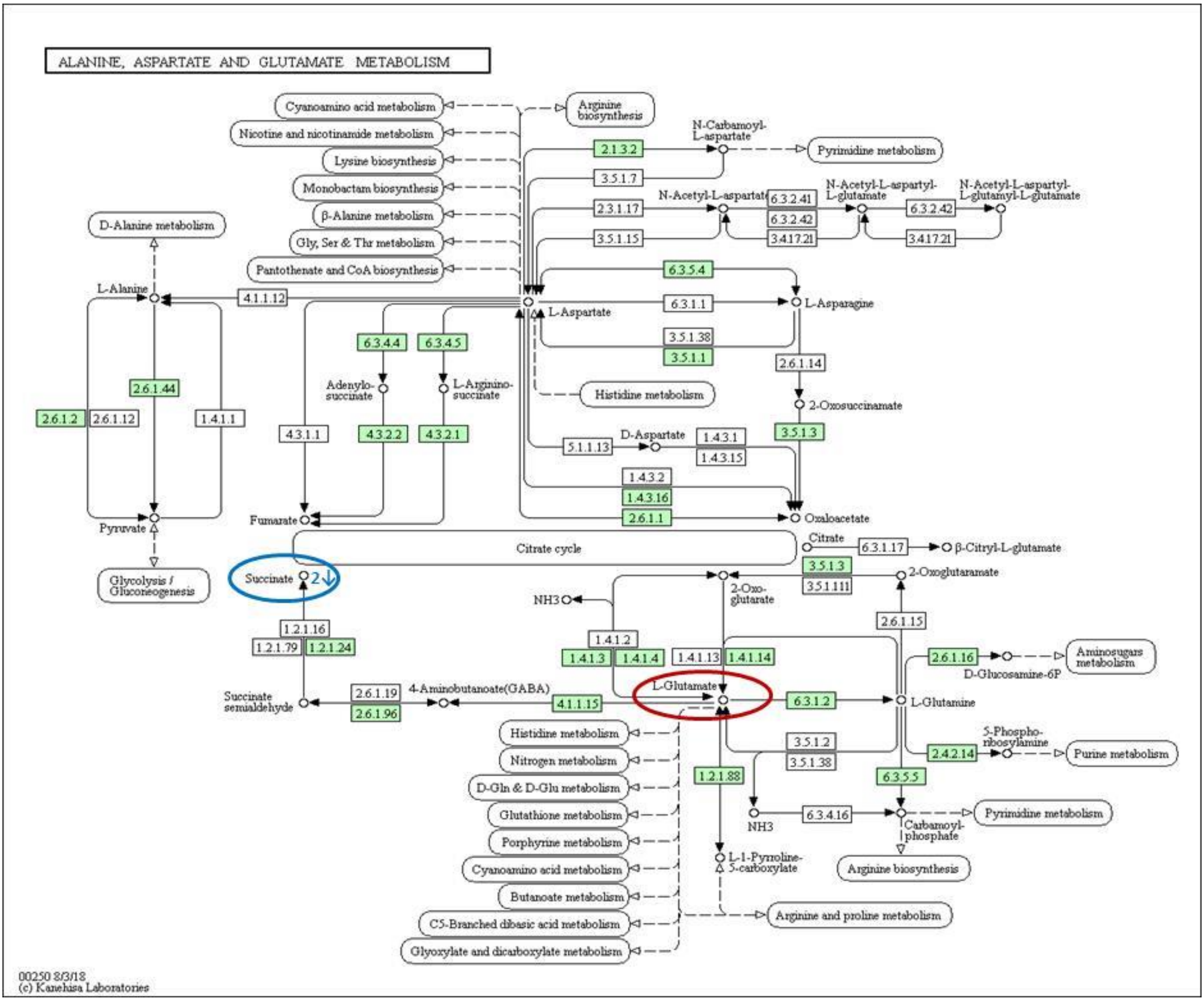

Figure S3.1-8. KEGG scheme 8a. Alanine, aspartate and glutamate metabolism.

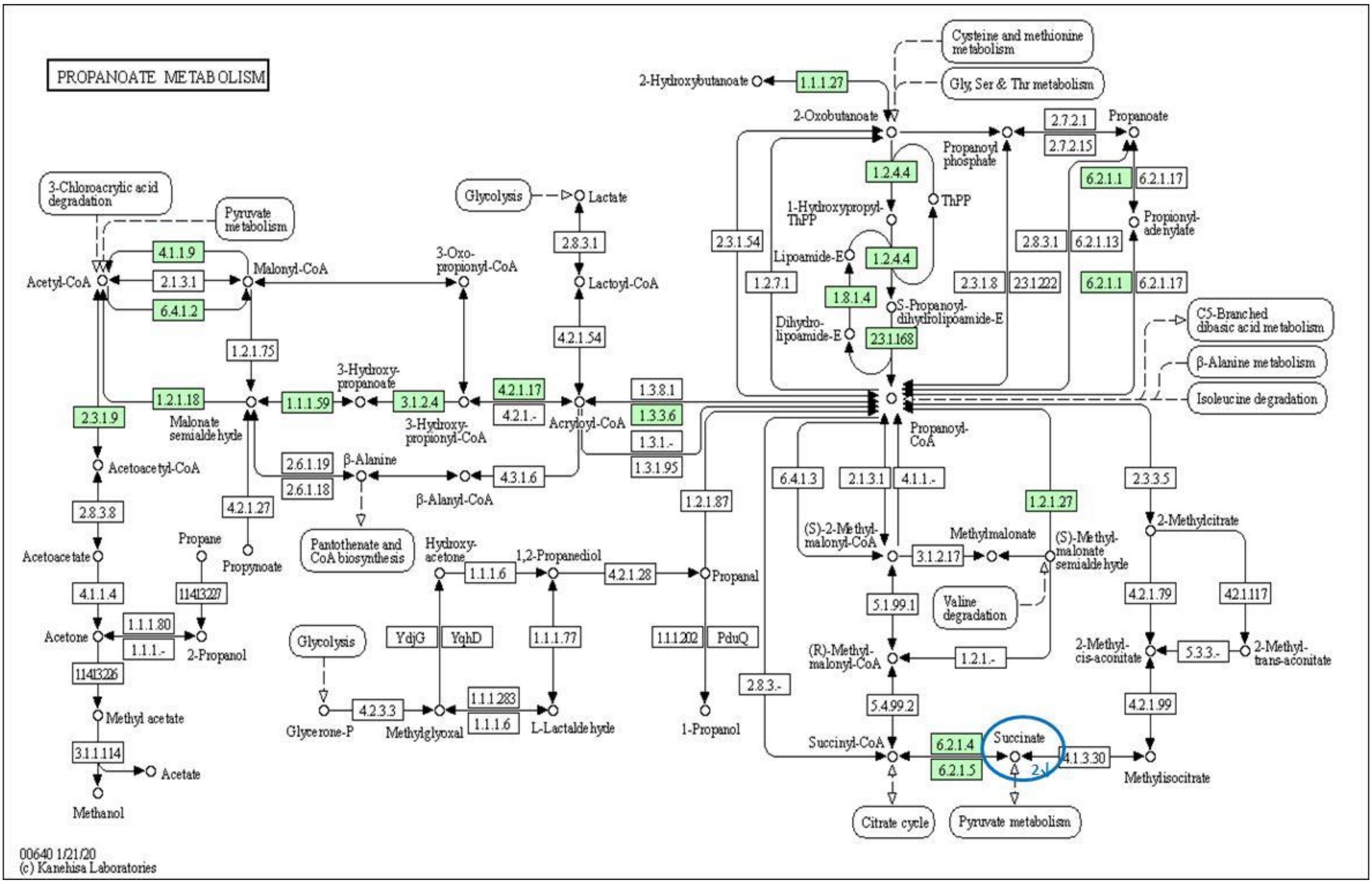

Figure S3.1-9. KEGG scheme 8b. Propanoate metabolism.

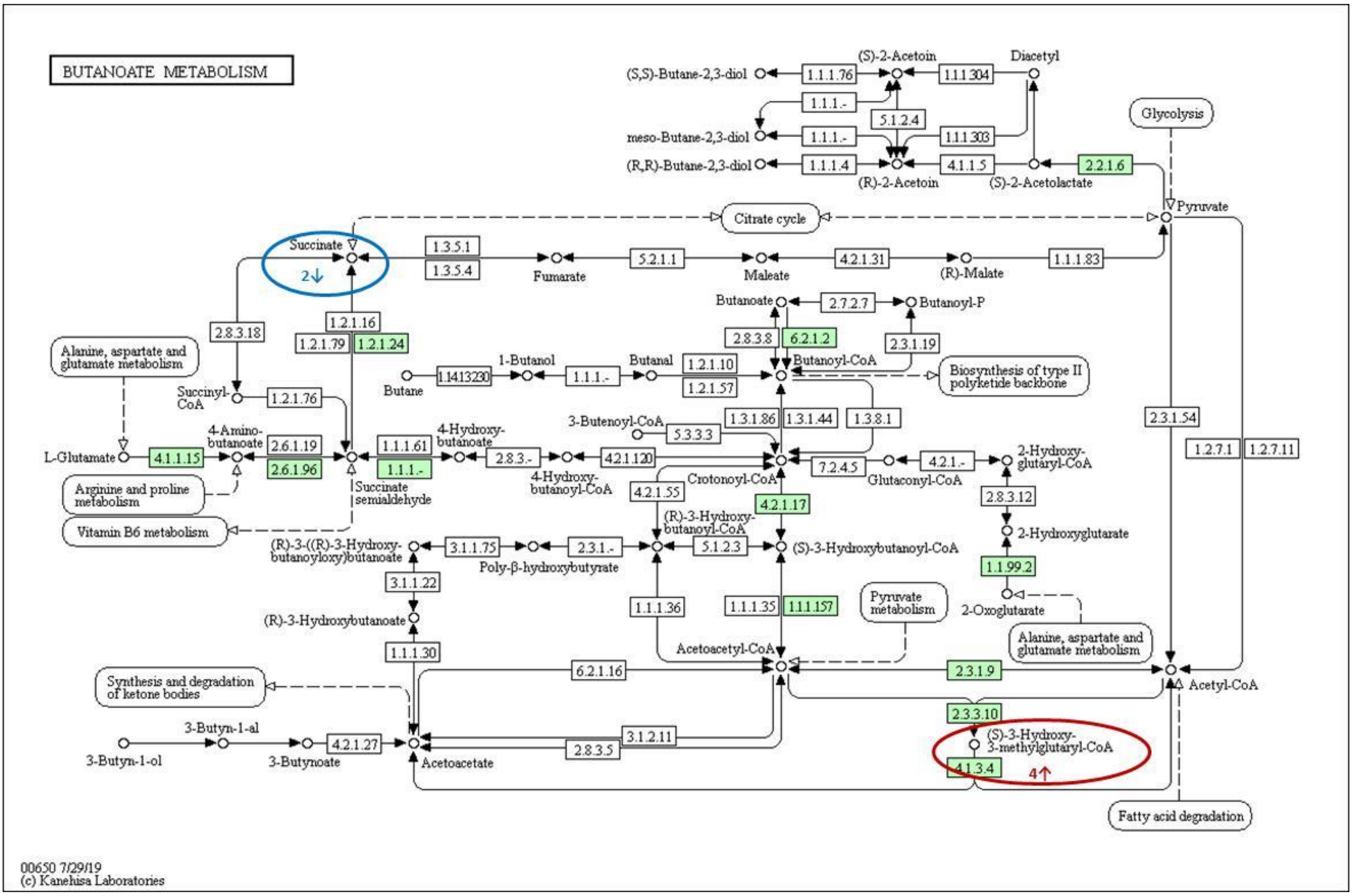

Figure S3.1-10. KEGG scheme 8c. Butanoate metabolism.

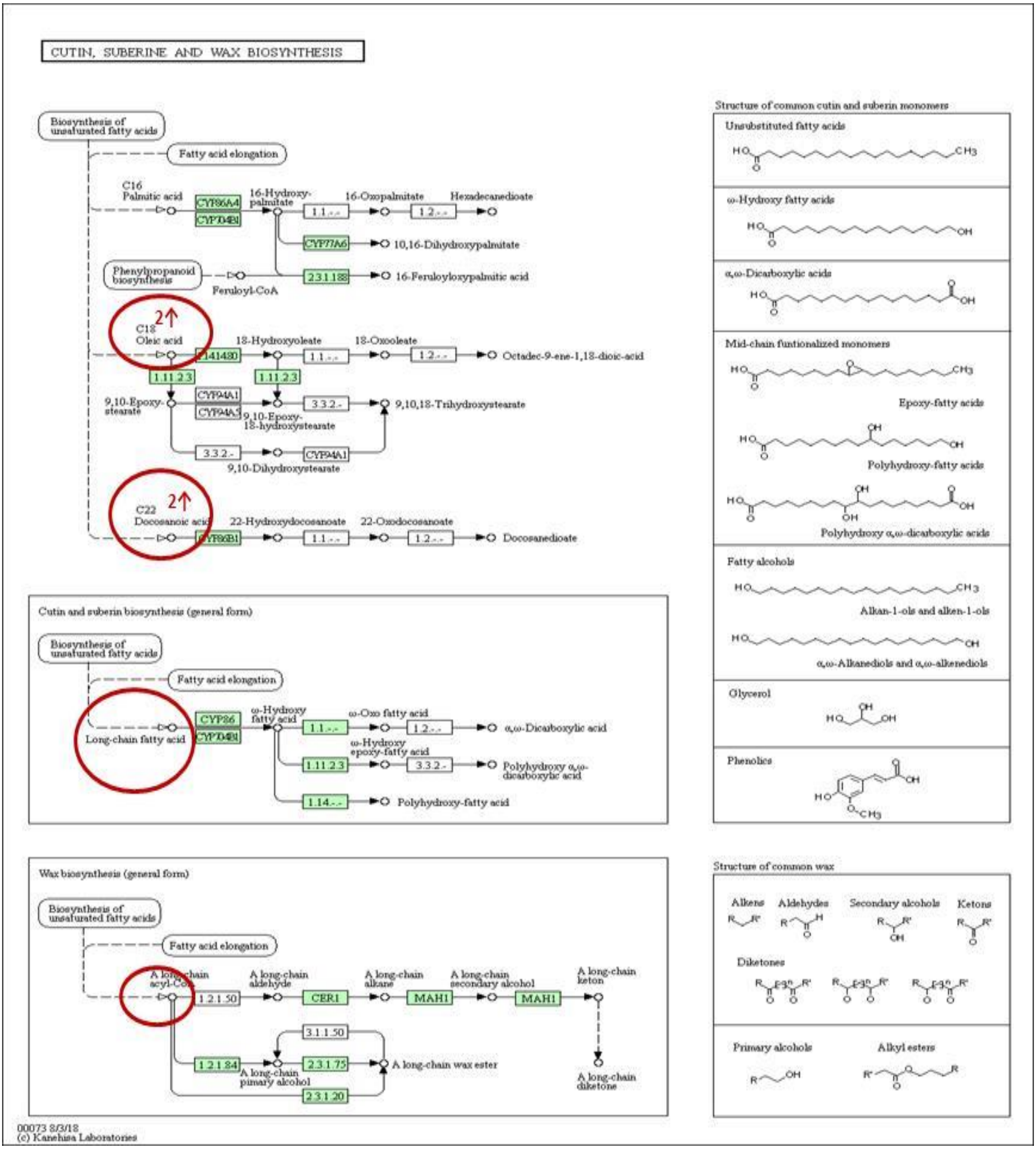

Figure S3.1-11. KEGG scheme 9. Cutin, suberine and wax biosynthesis.

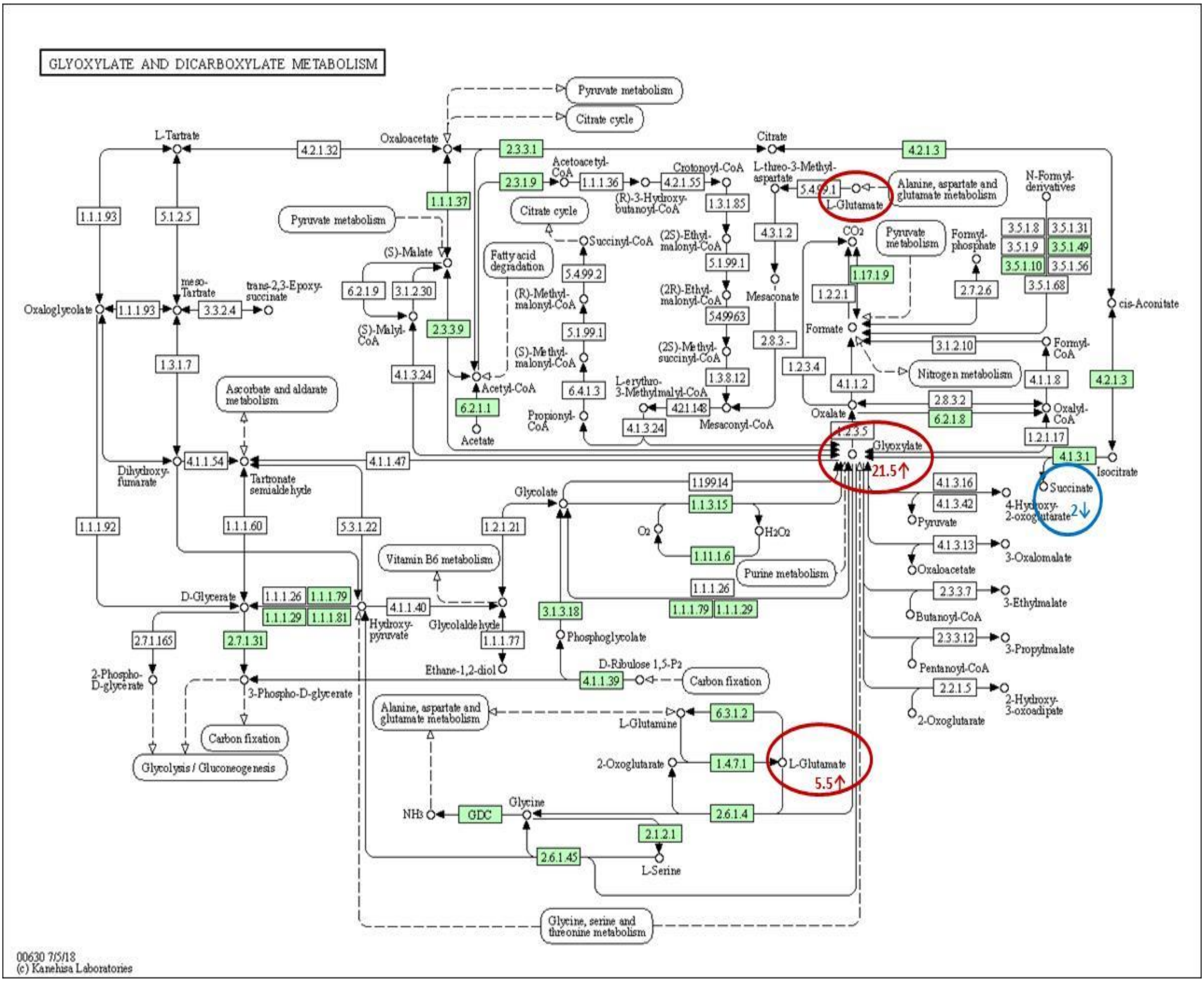

Figure S3.1-12. KEGG scheme 10. Glyoxylate and dicarboxylate metabolism

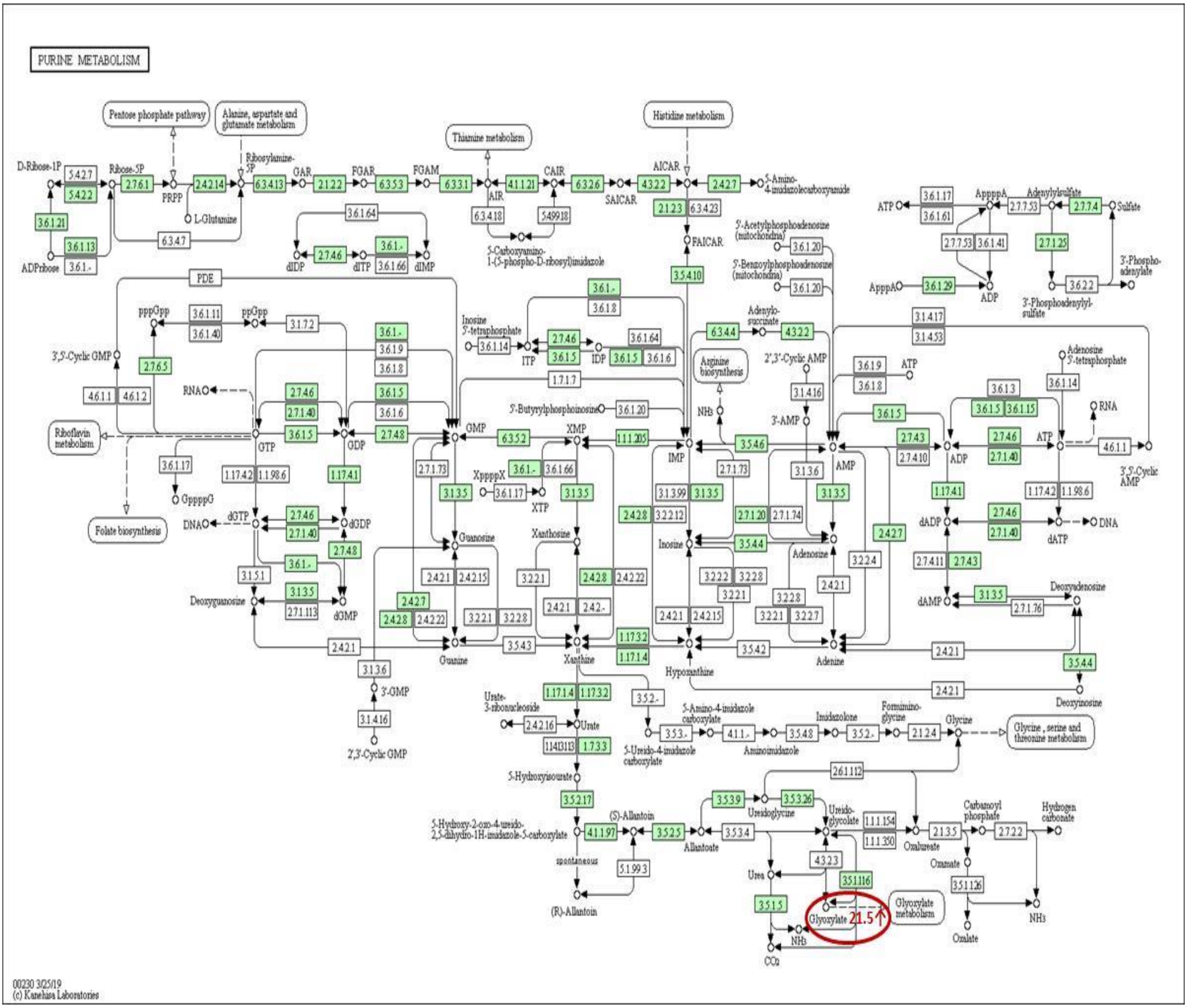

Figure S3.1-13. KEGG scheme 11a. Purine metabolism

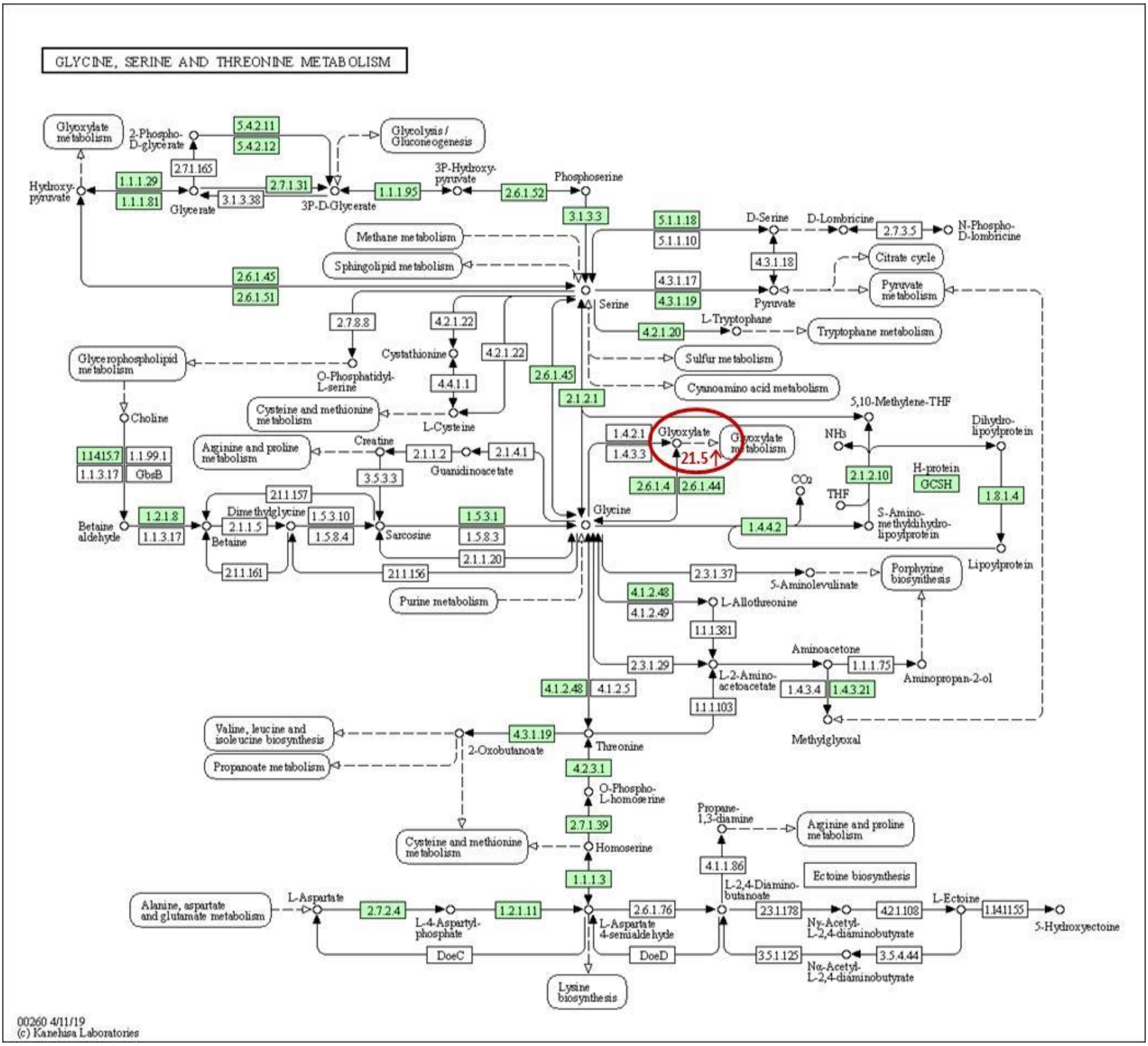

Figure S3.1-14. KEGG scheme 11b. Glycine, serine and threonine metabolism.

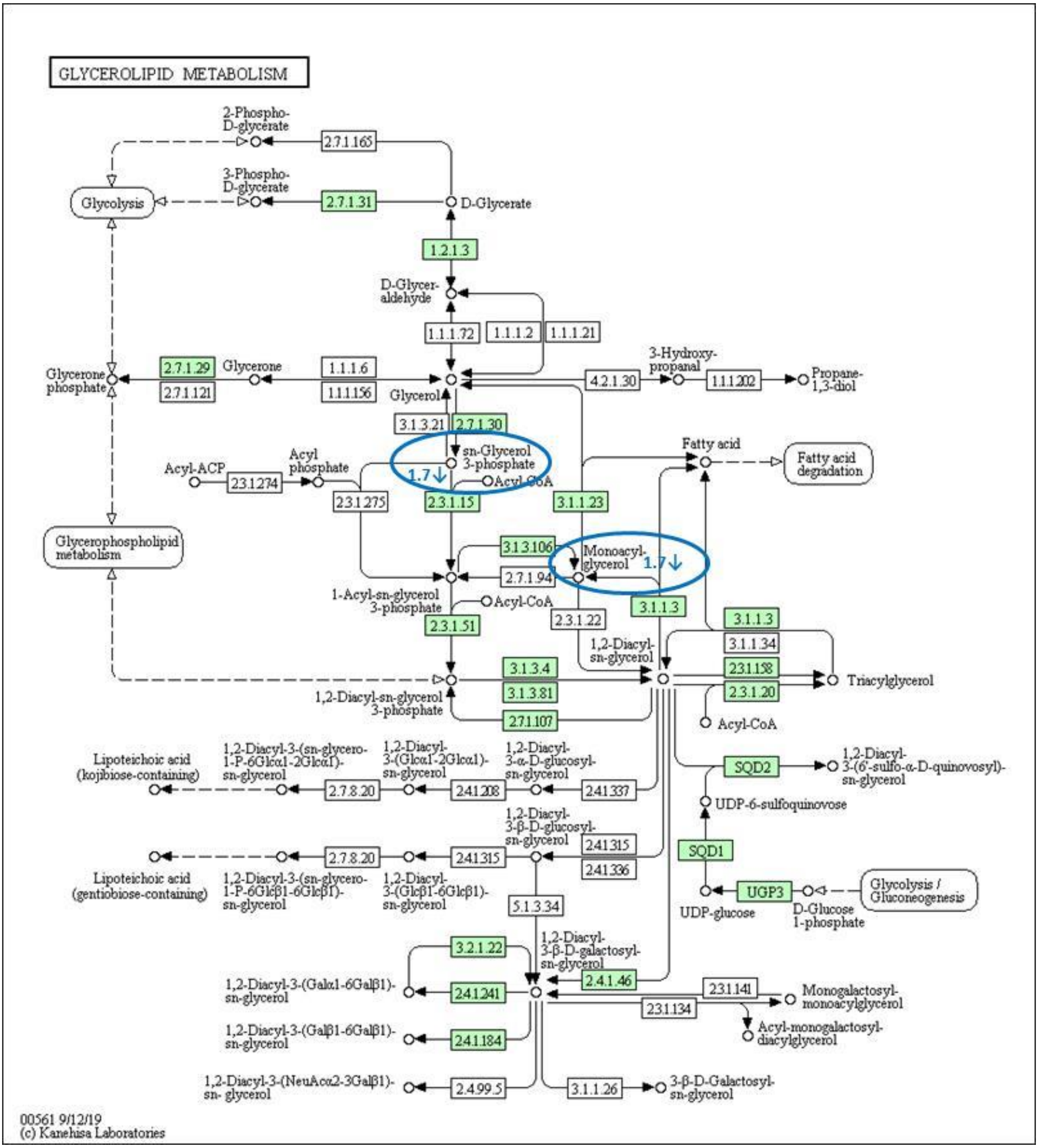

Figure S3.1-15. KEGG scheme 12. Glycerolipid metabolism.

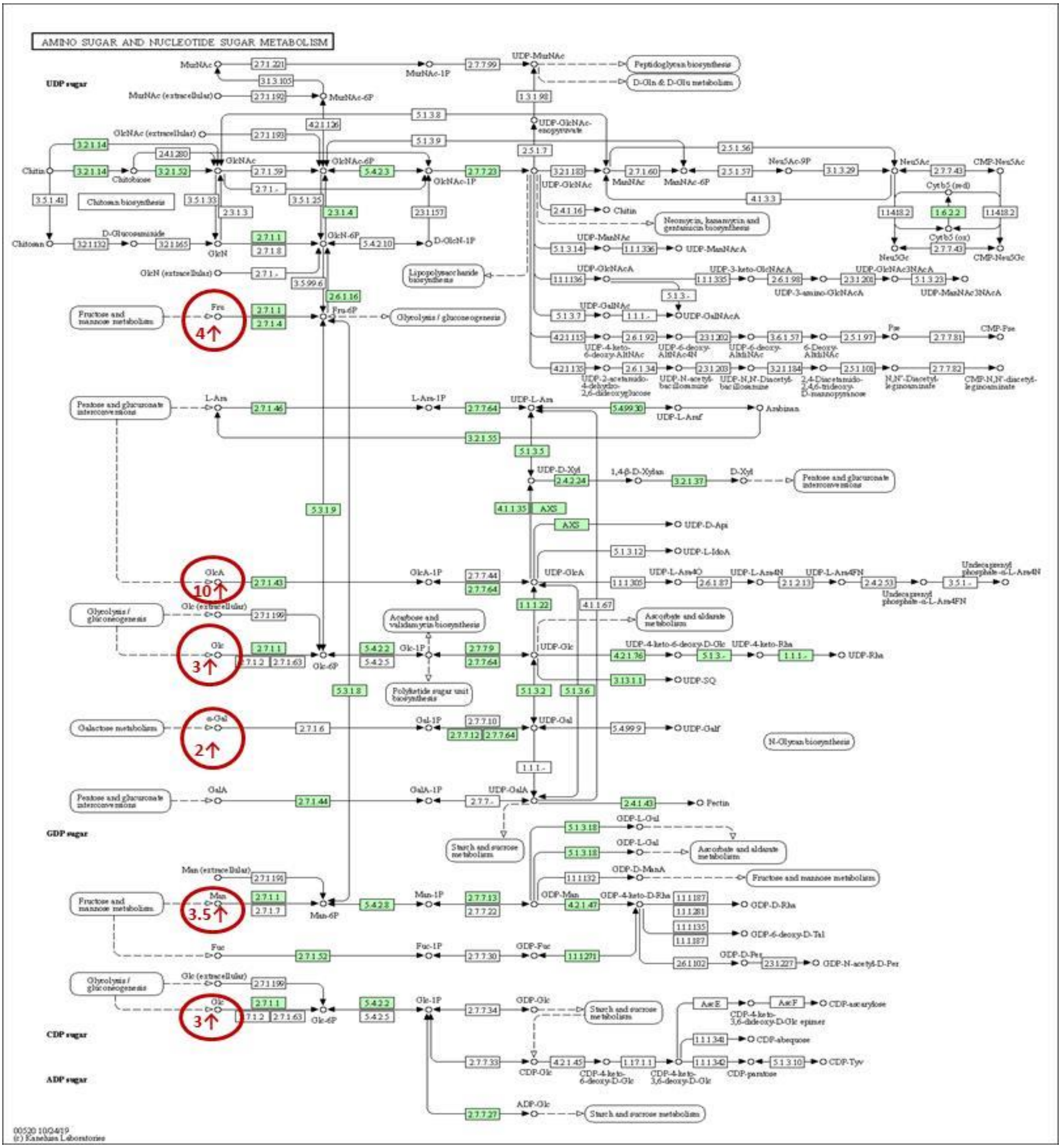

Figure S3.1-16. KEGG scheme 13. Amino sugar and nucleotide sugar metabolism.

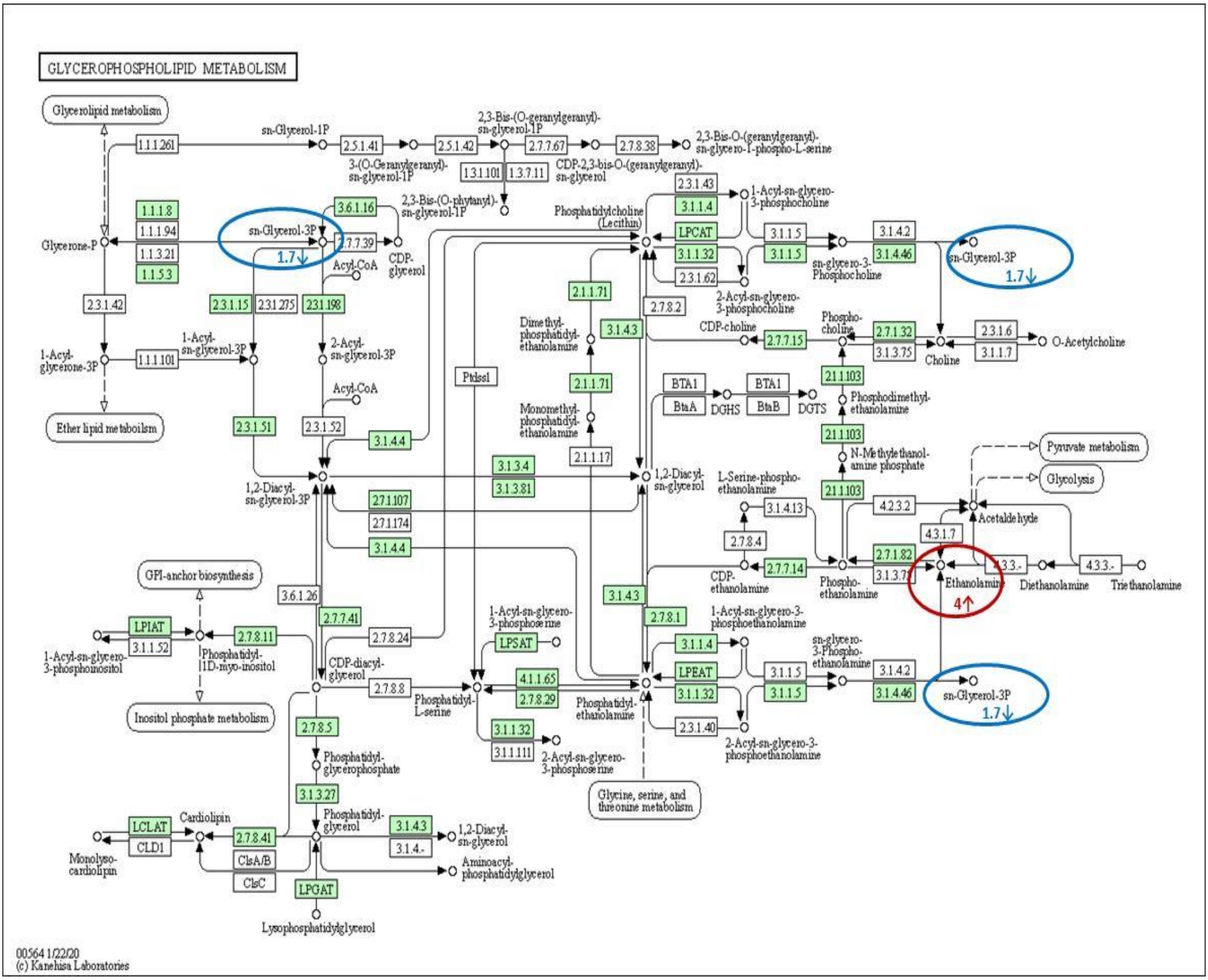

Figure S3.1-17. KEGG scheme 14. Glycerophospholipid metabolism

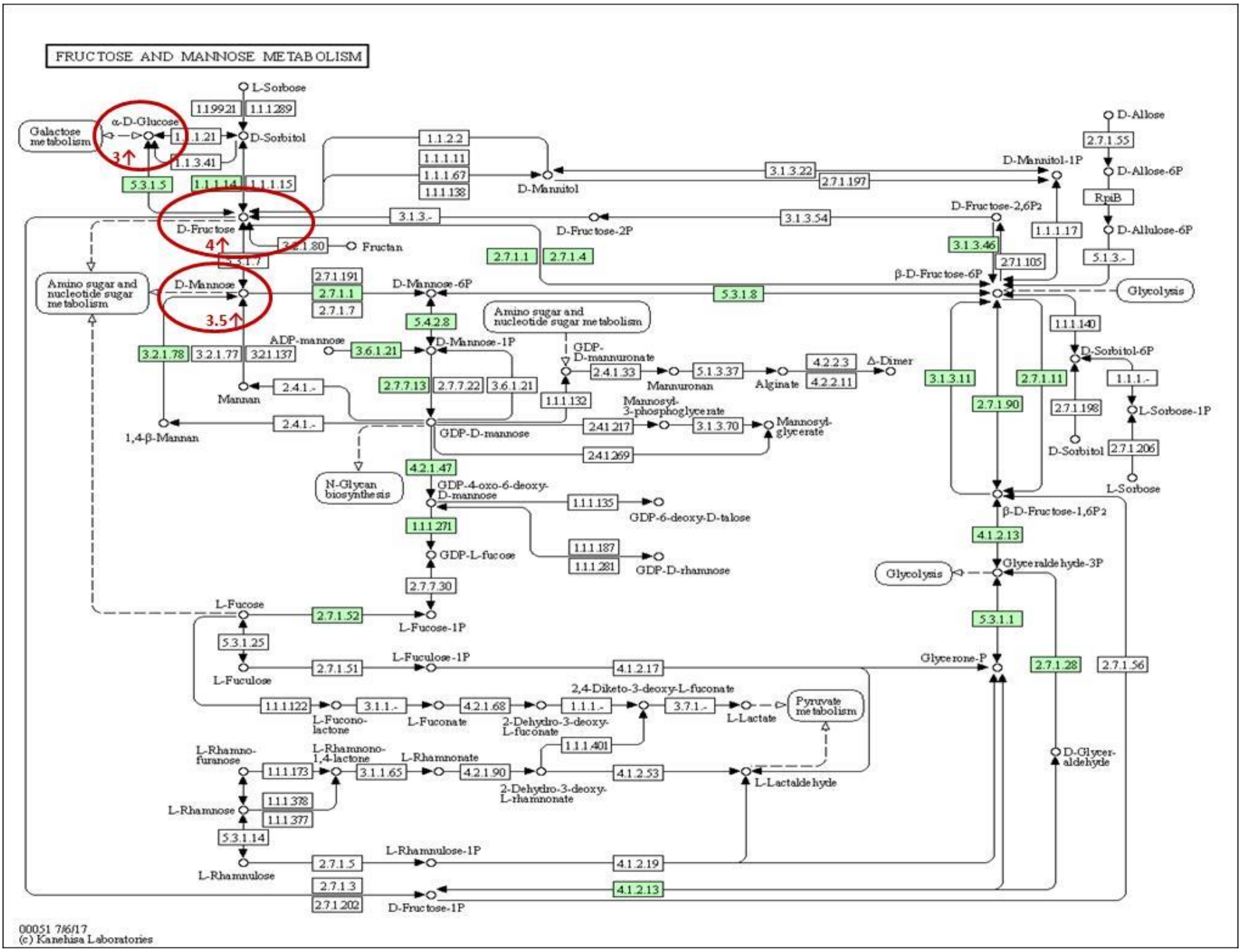

Figure S3.1-18. KEGG scheme 15. Fructose and mannose metabolism

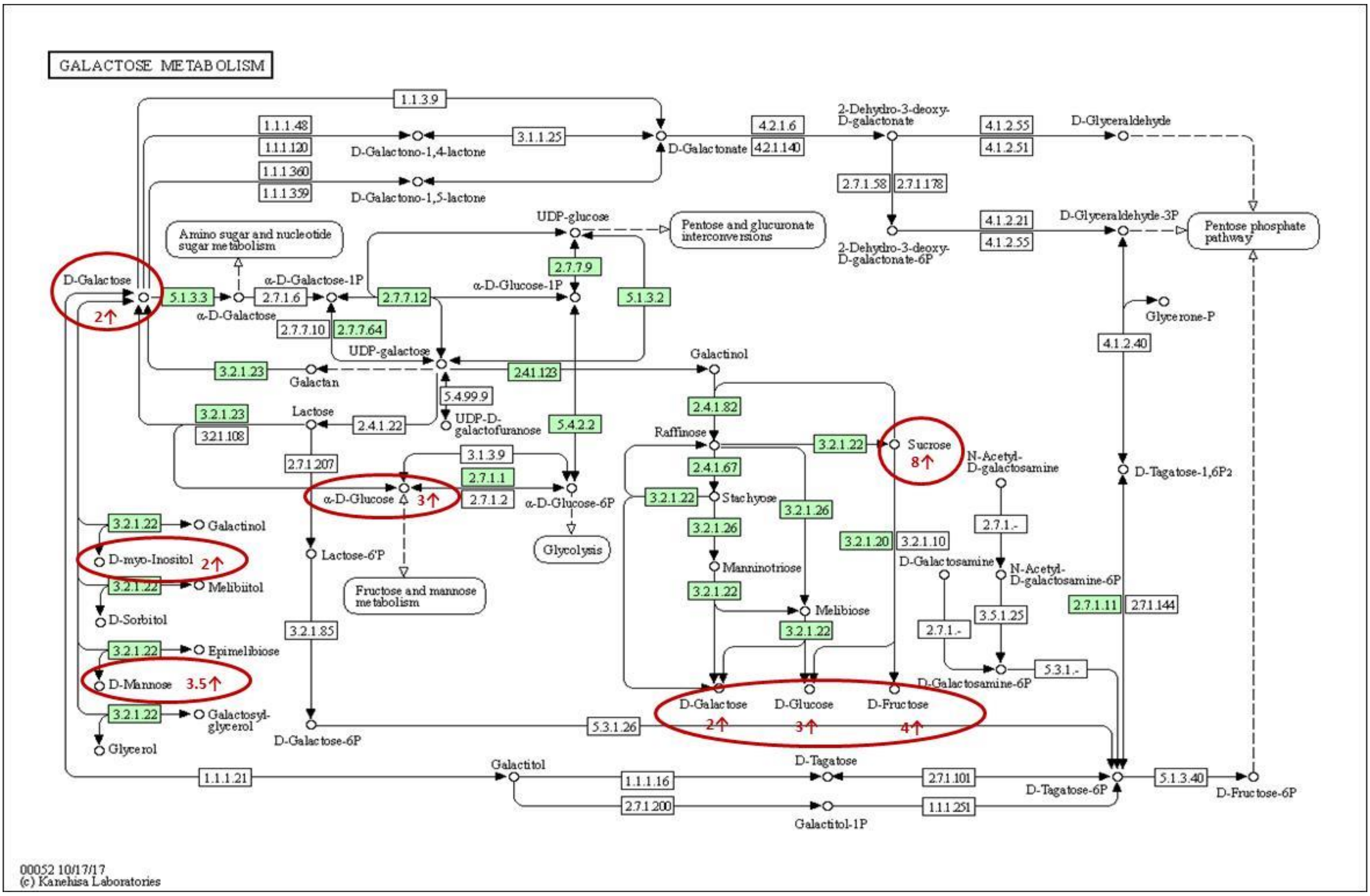

Figure S3.1-19. KEGG scheme 16. Galactose metabolism

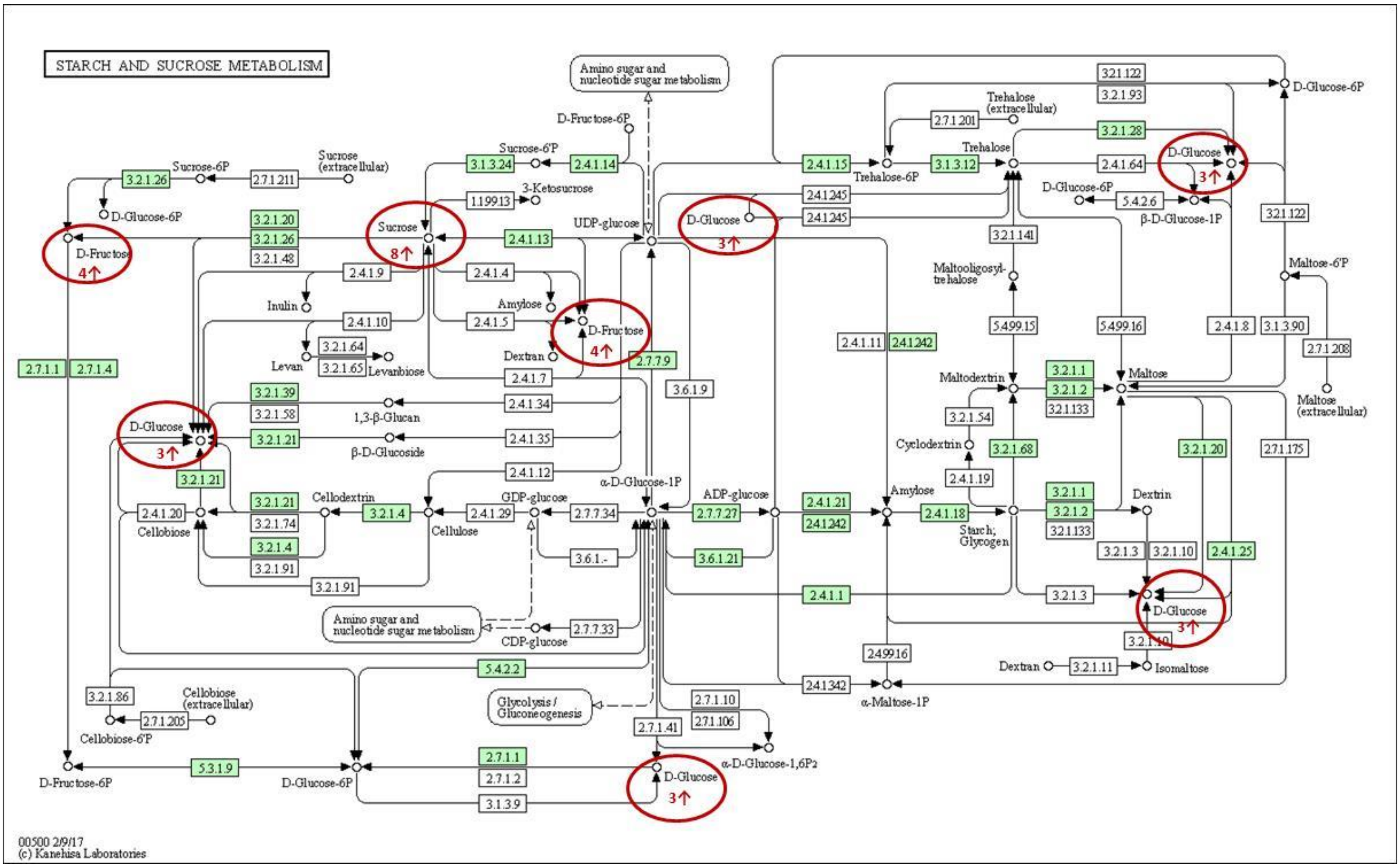

Figure S3.1-20. KEGG scheme 17. Starch and sucrose metabolism.

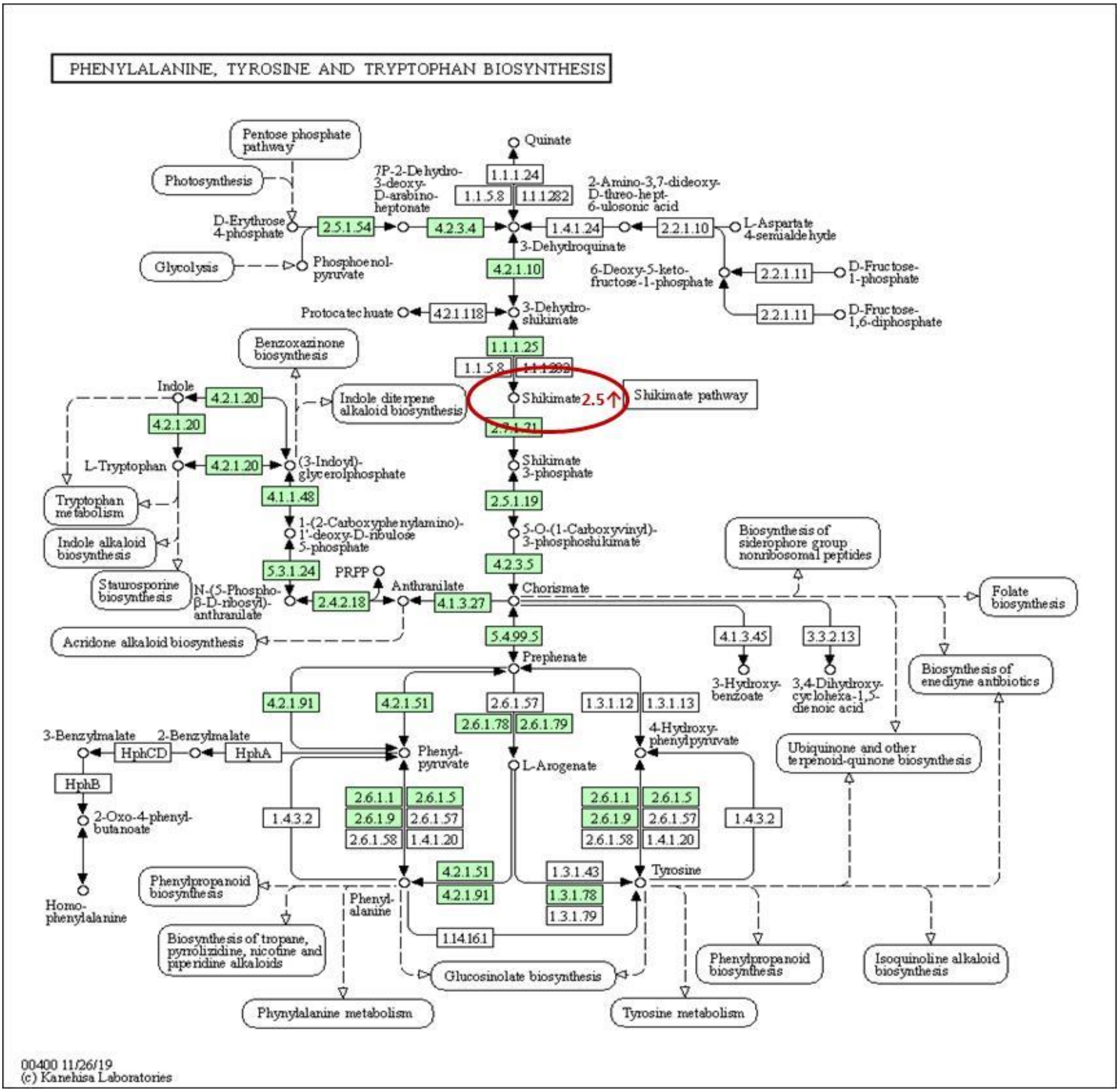

Figure S3.1-21. KEGG scheme 18. Phe, Tyr, Trp metabolism.

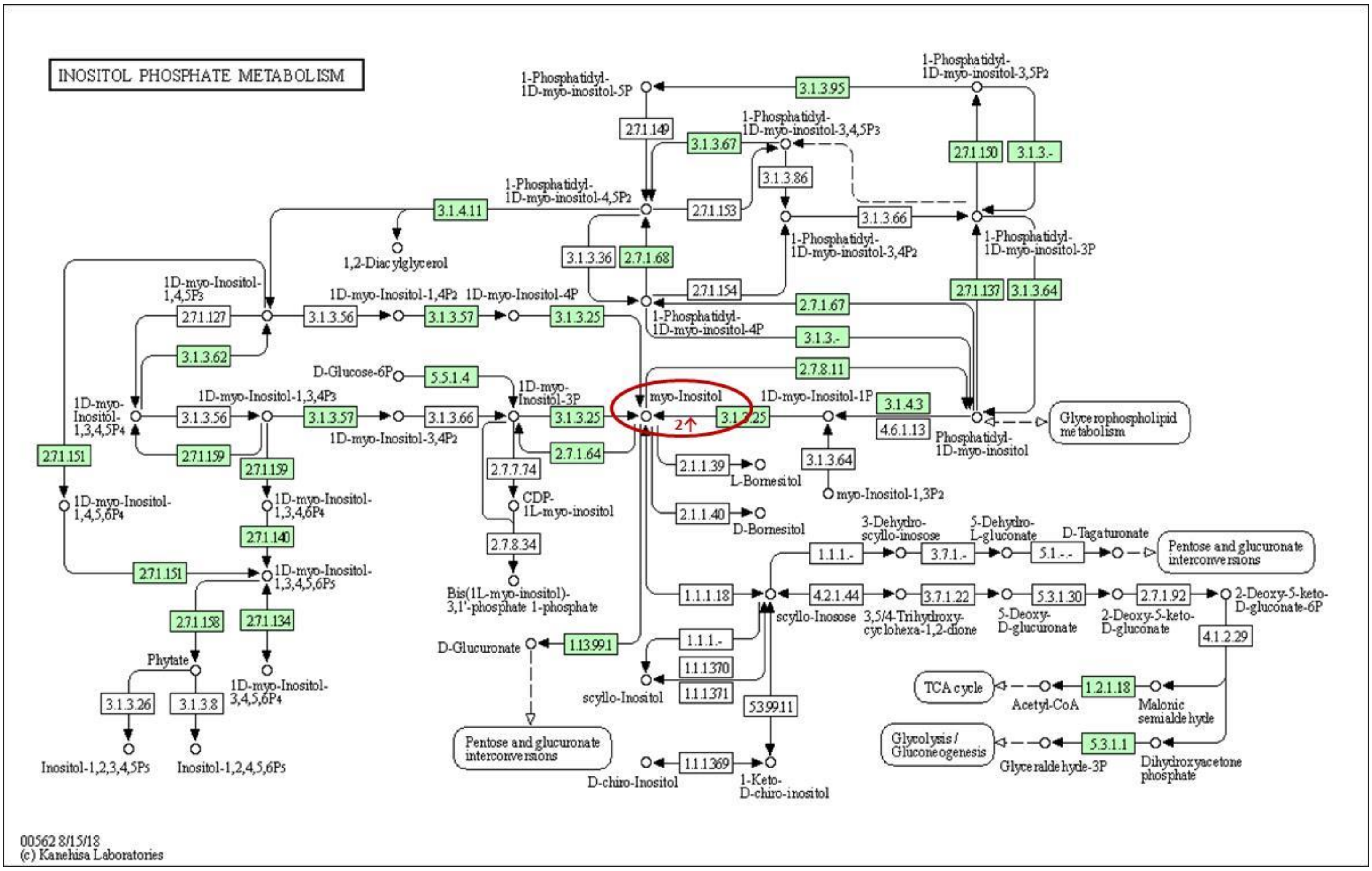

Figure S3.1-22. KEGG scheme 19. Inositol phosphate metabolism

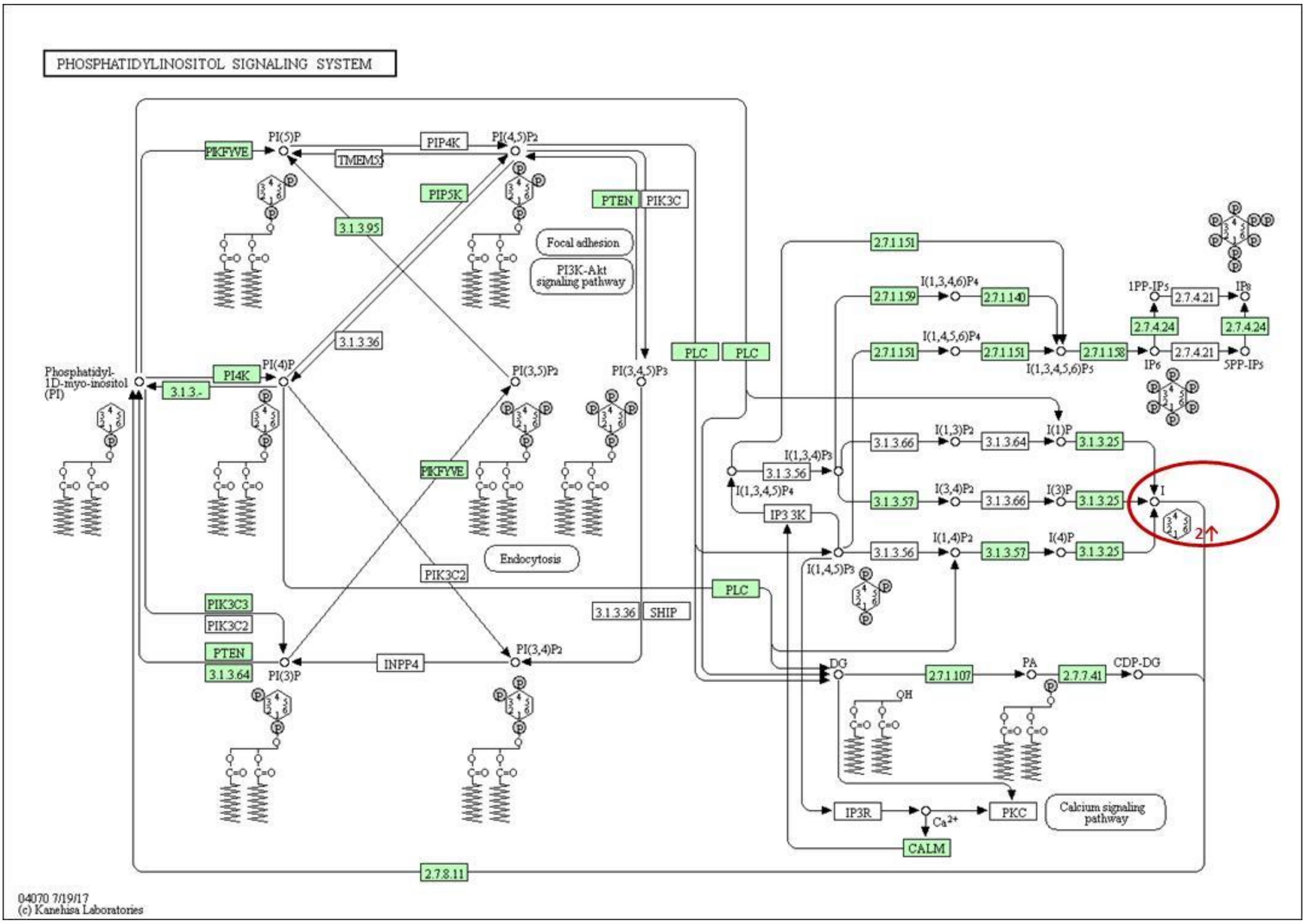

Figure S3.1-23. KEGG scheme 20. Phosphatidylinositol signaling

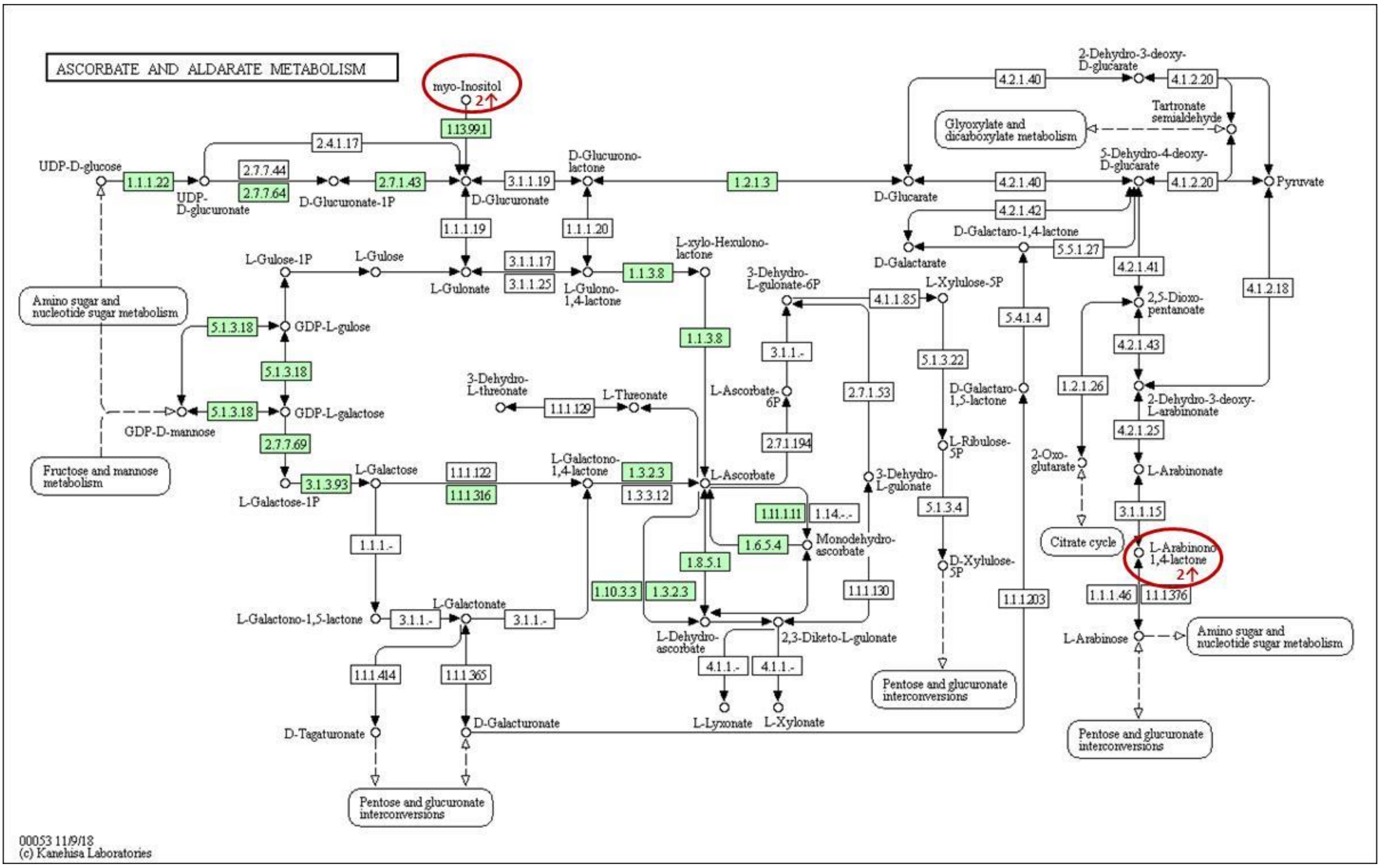

Figure S3.1-24. KEGG scheme 21. Ascorbate and aldarate metabolism.

Part 2. Metabolic pathways of Pathway Analysis for Zn-regulated metabolites in roots of *A. caudatus*

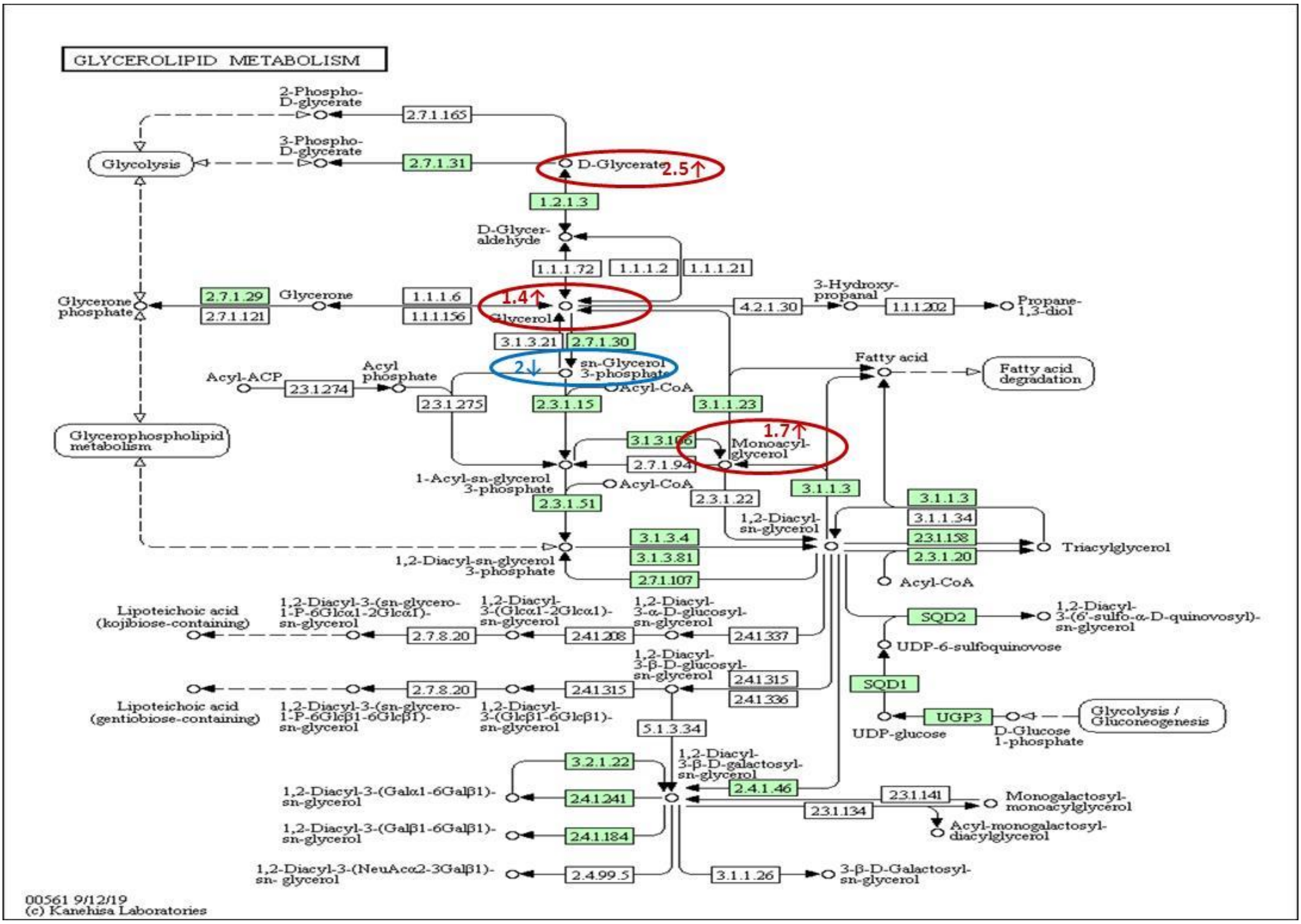

Figure S3.2-1. KEGG scheme 1. Glycerolipid metabolism**.**

Circles mark Zn^{2+}-related metabolites, red and blue circle colors denote up- and down-regulated metabolites, respectively. Value and arrow in the circles indicate fold and direction of the changes, respectively, in comparison with controls. To address Zn^{2+}-related metabolites (t-test $p \leq 0.05$) in roots quantified by untargeted and targeted methods refer to Tables 2 and 3, respectively.

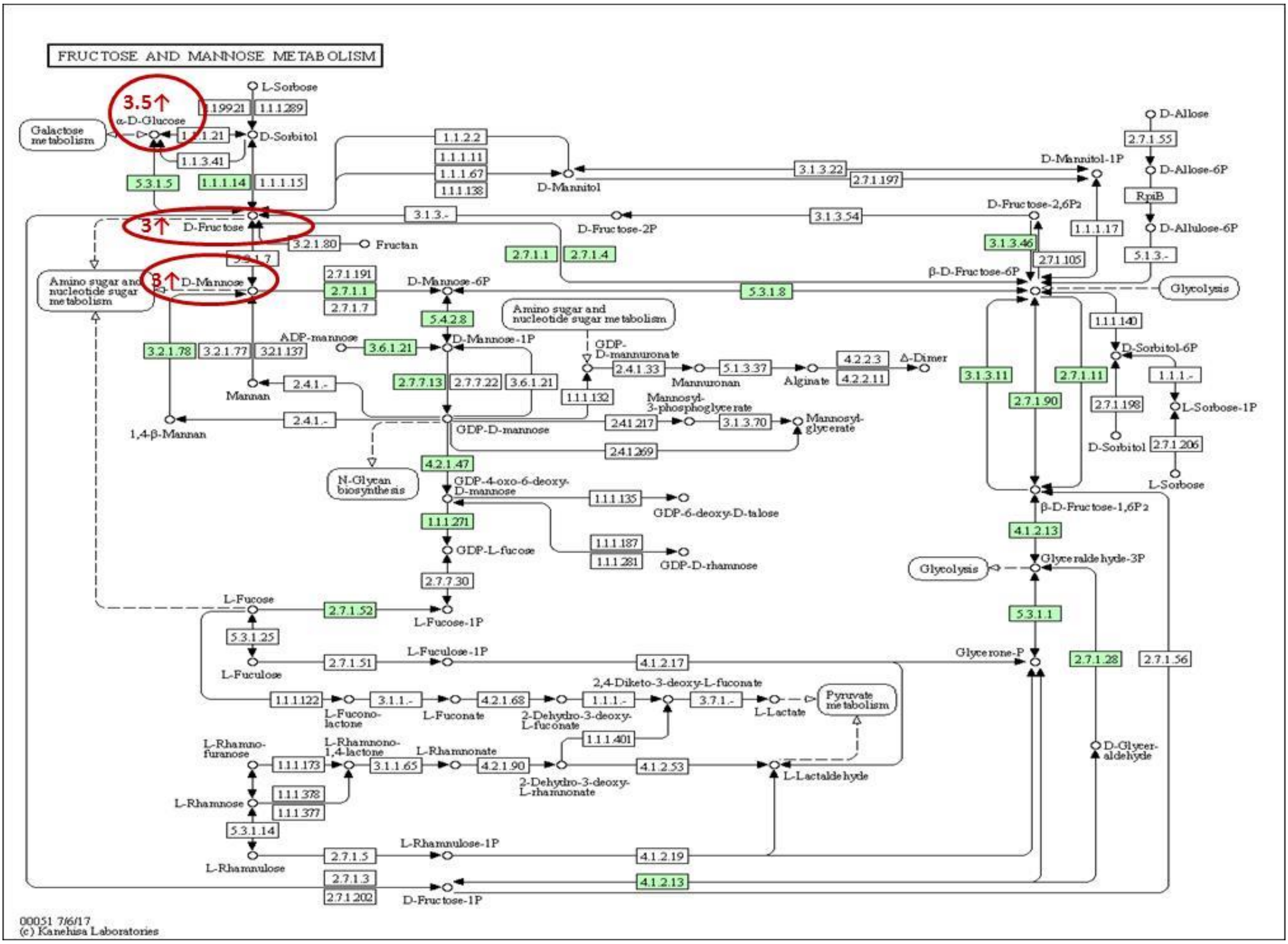

Figure S3.2-2. KEGG scheme 2. Fructose and mannose metabolism.

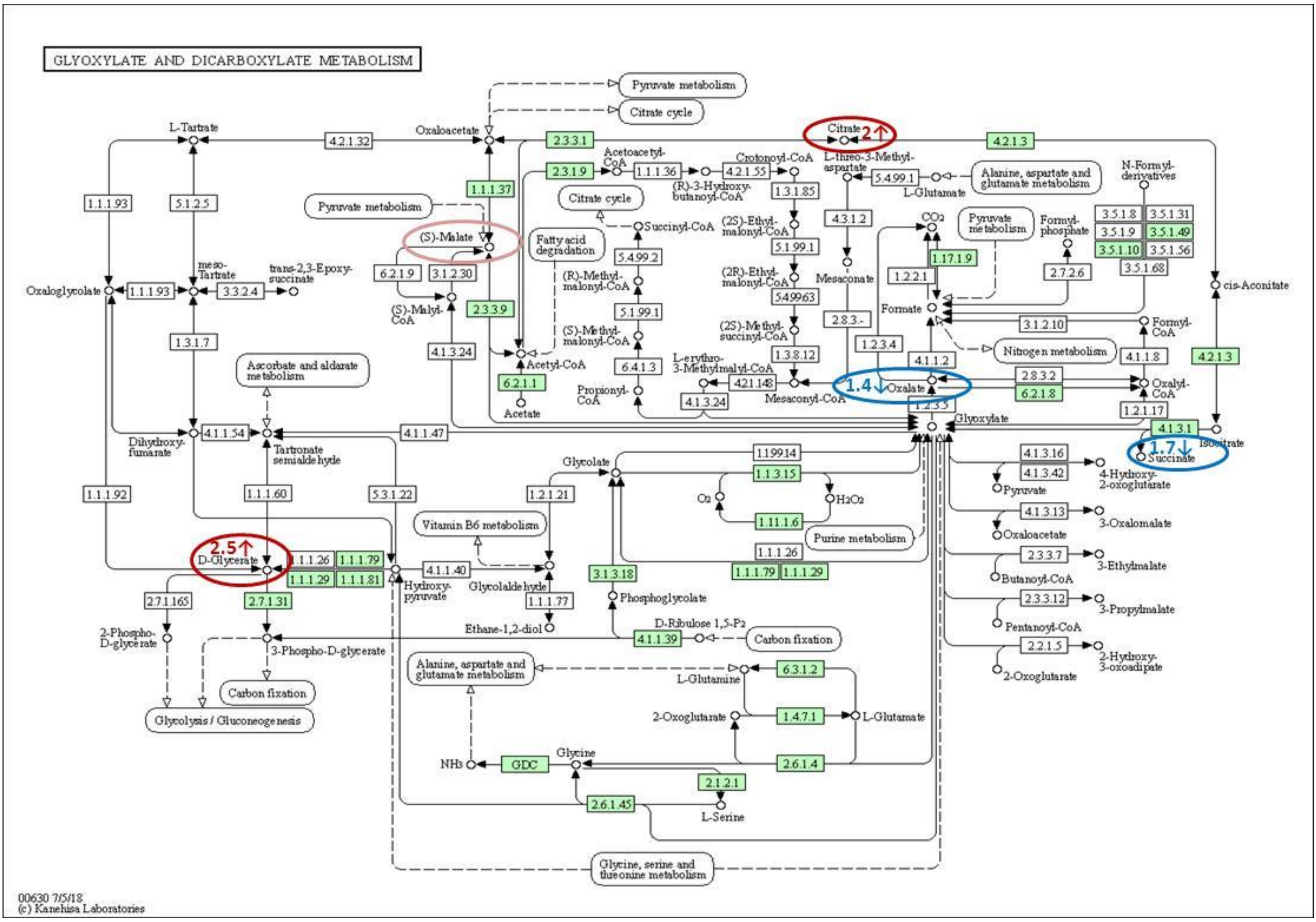

Figure S3.2-3. KEGG scheme 3. Glyoxylate and dicarboxylate metabolism

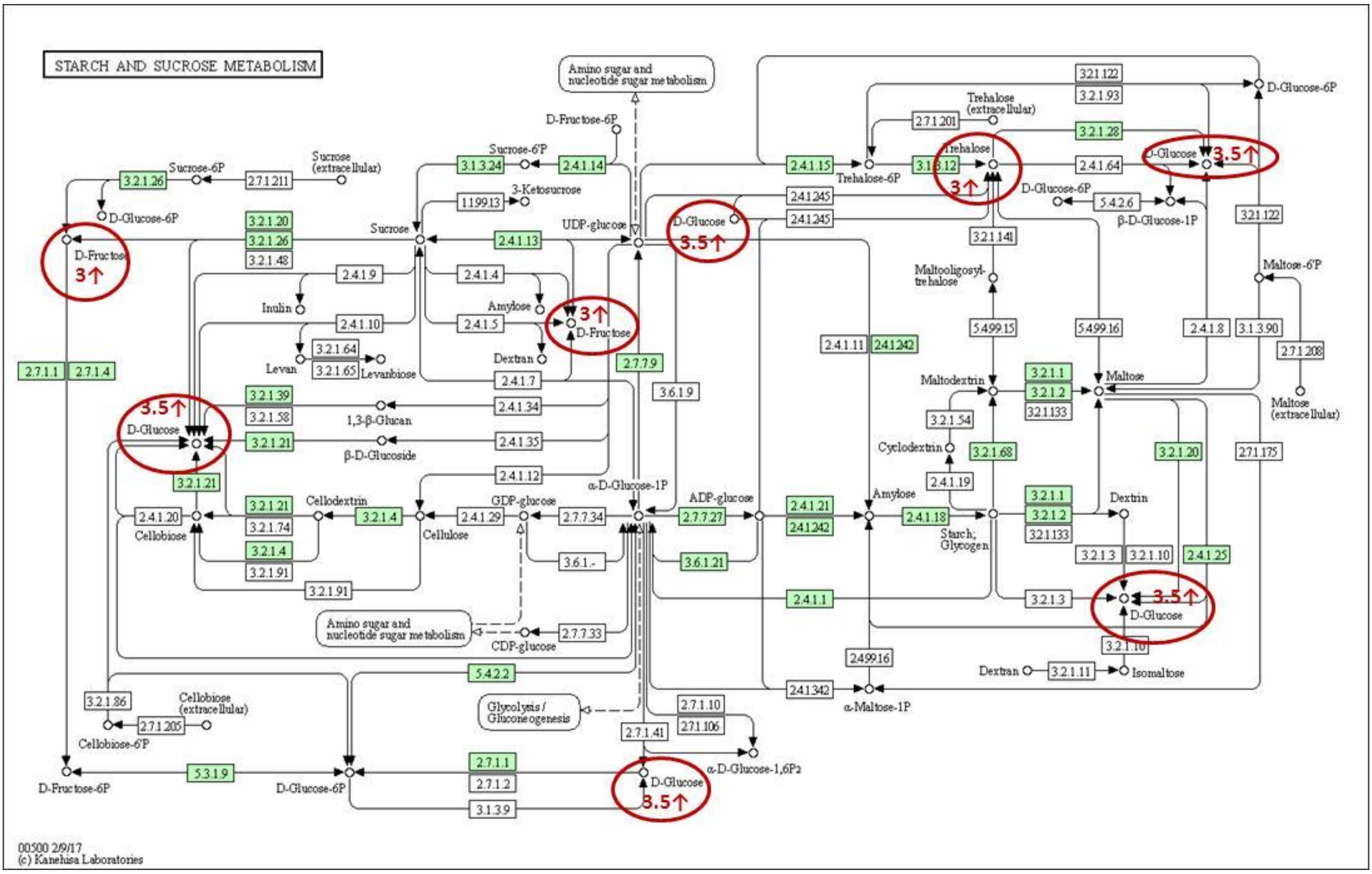

Figure S3.2-4. KEGG scheme 4. Starch and sucrose metabolism.

.

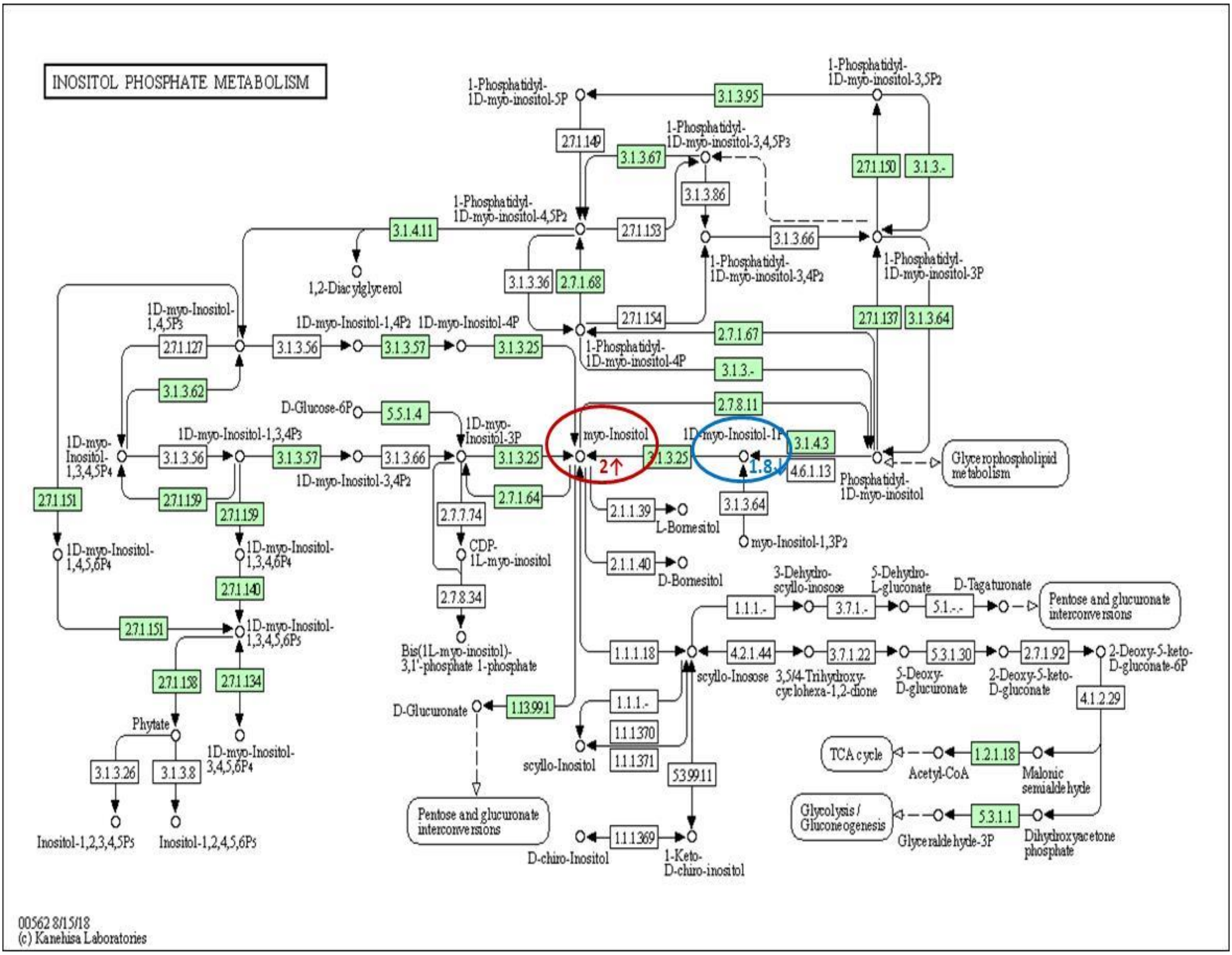

Figure S3.2-5. KEGG scheme 5. Inositol phosphate metabolism.

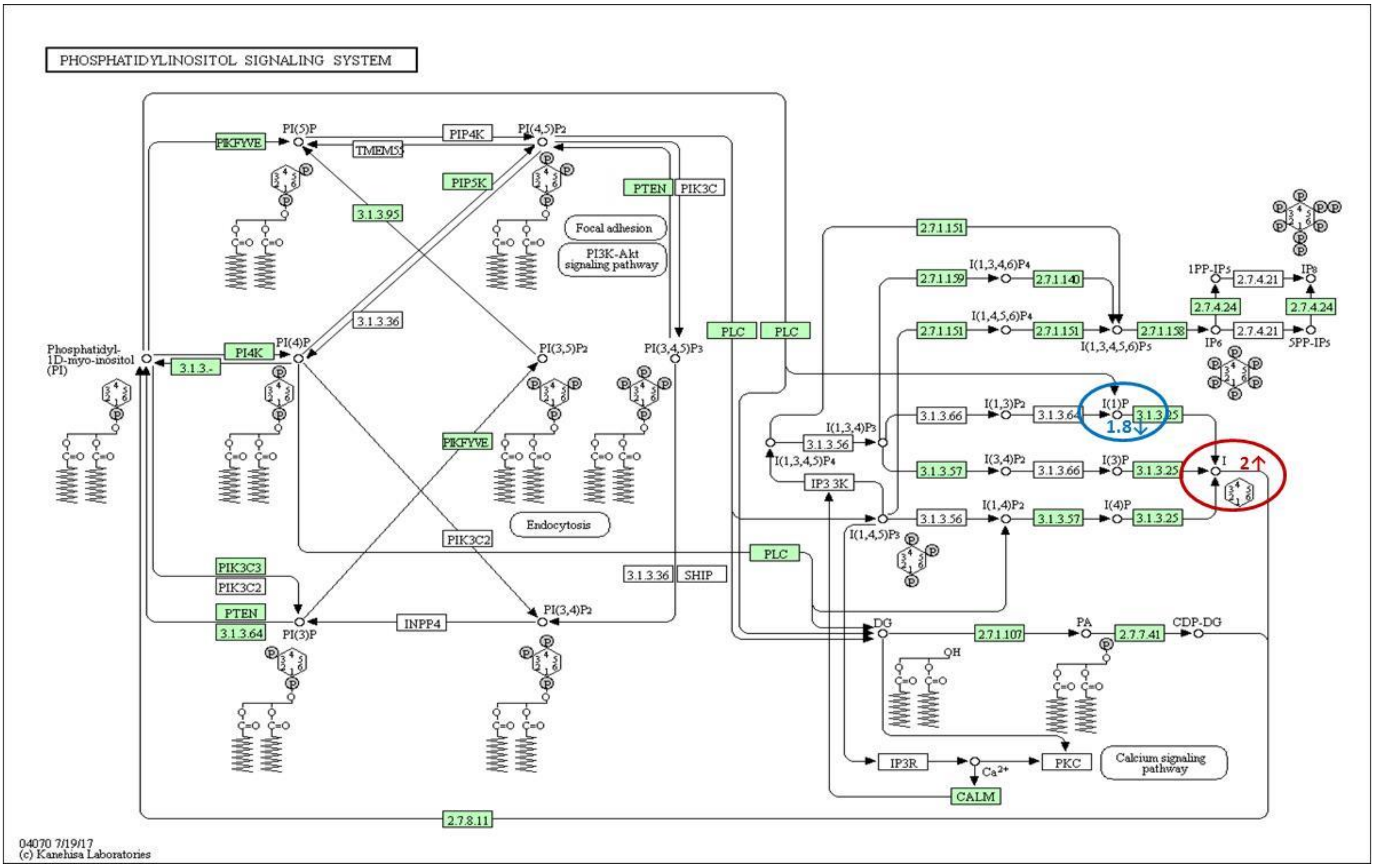

Figure S3.2-6. KEGG scheme 6. Phosphatidylinositol signaling system.

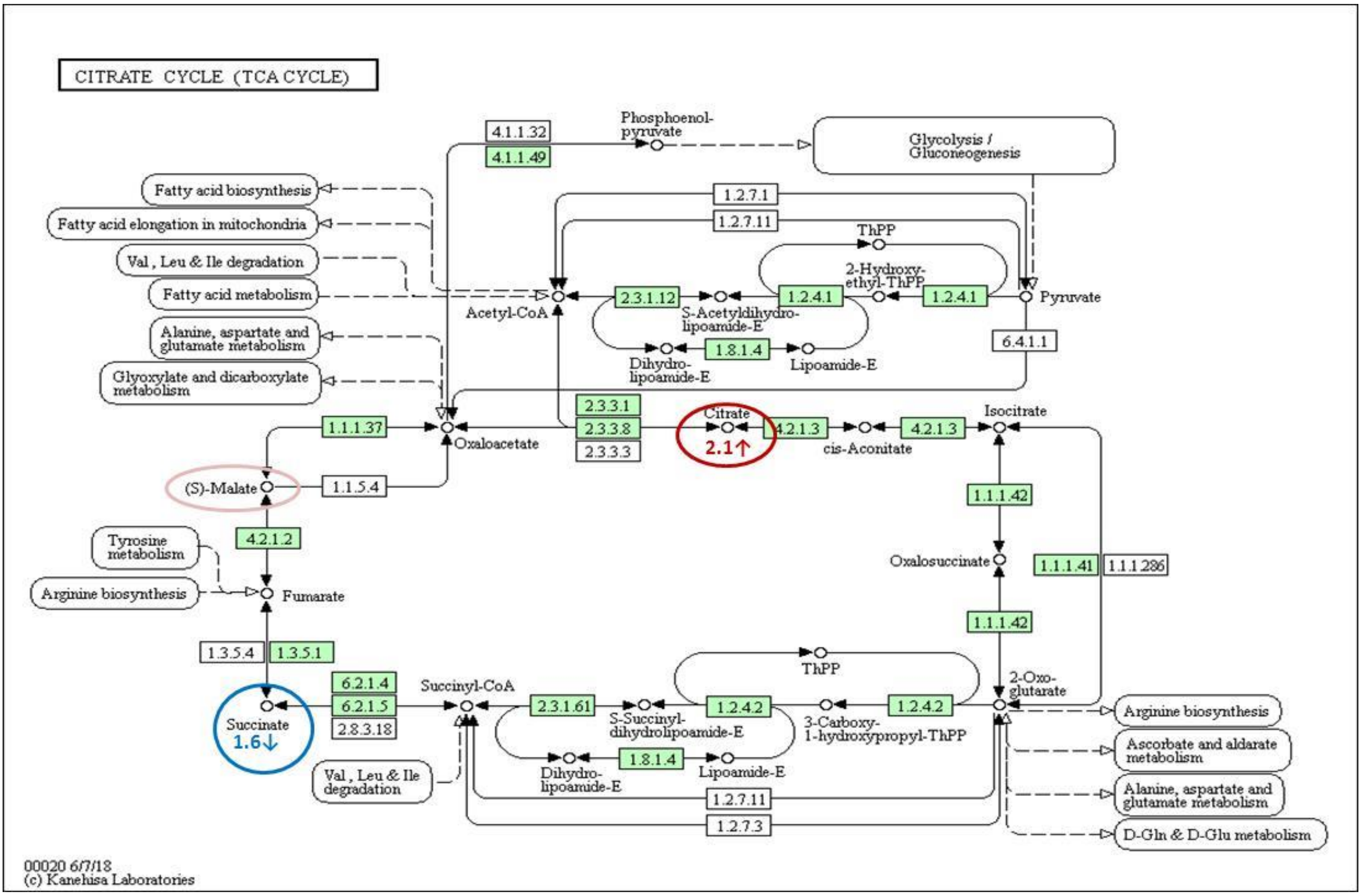

Figure S3.2-7. KEGG scheme 7. Citrate cycle (TCA Cycle).

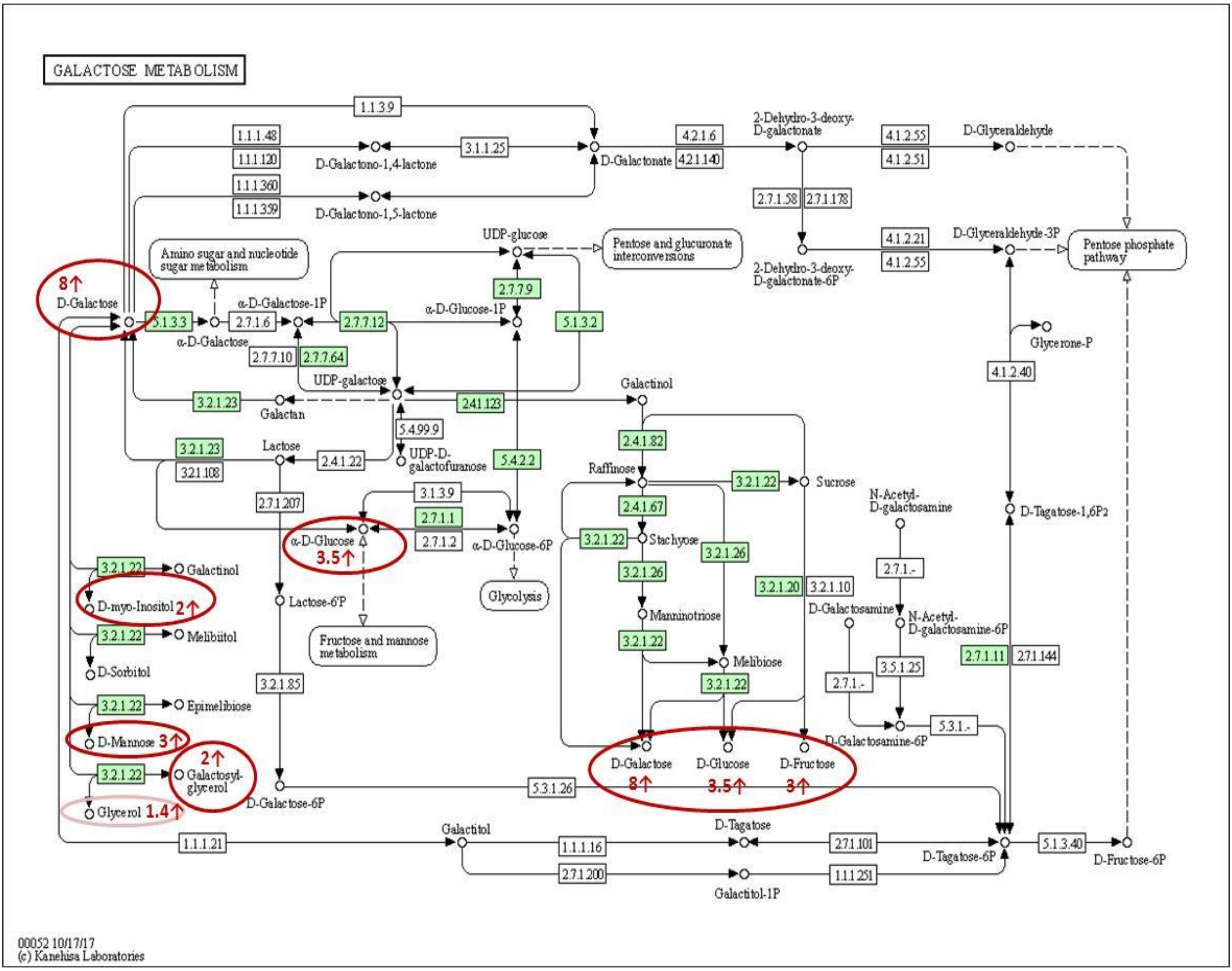

Figure S3.2-8. KEGG scheme 8. Galactose metabolism.

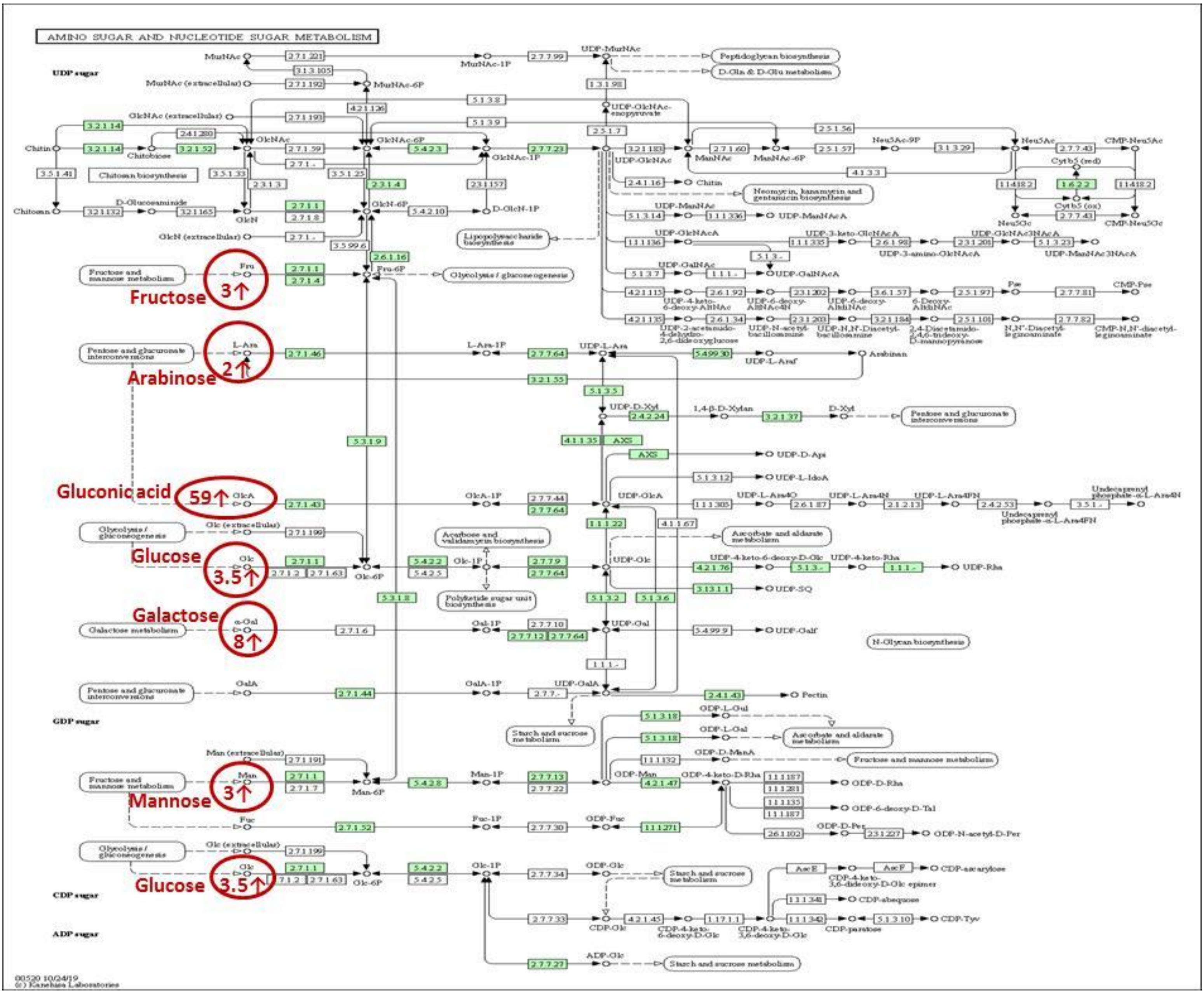

Figure S3.2-9. KEGG scheme 9. Amino acid and nucleotide sugar metabolism

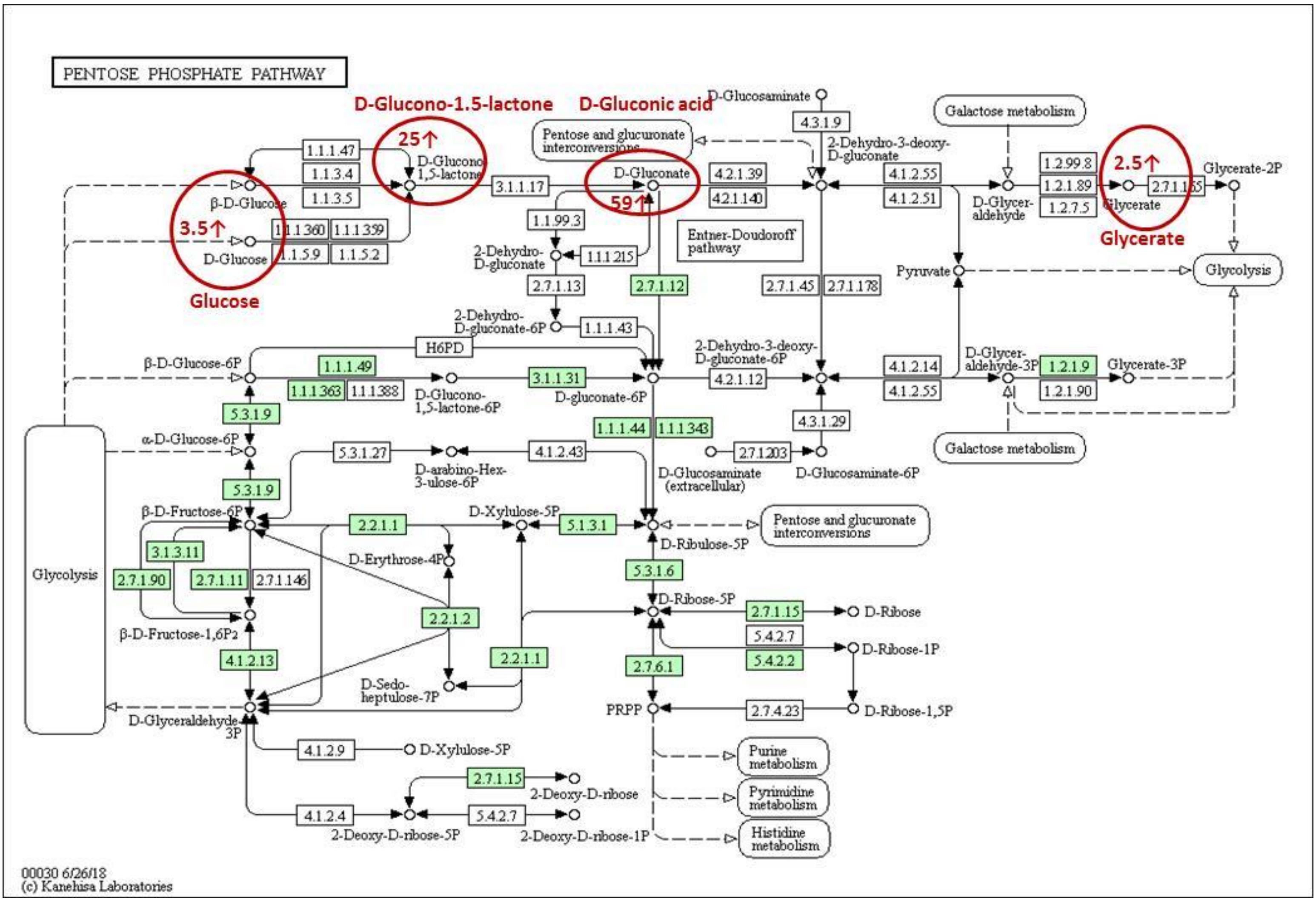

Figure S3.2-10. KEGG scheme 10. Pentose Phosphate pathway.

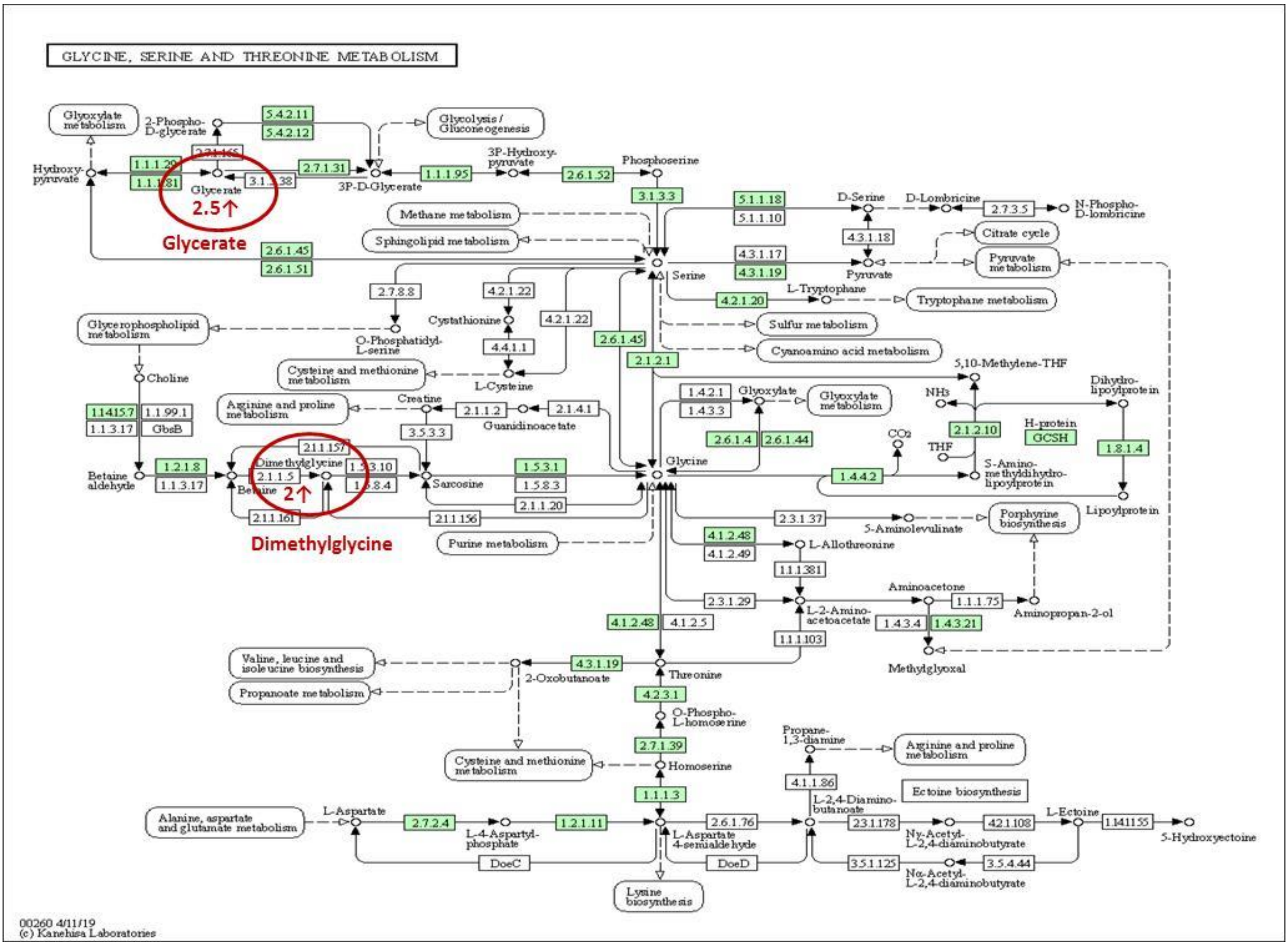

Figure S3.2-11. KEGG scheme 11. Glycine, serine threonine metabolism.

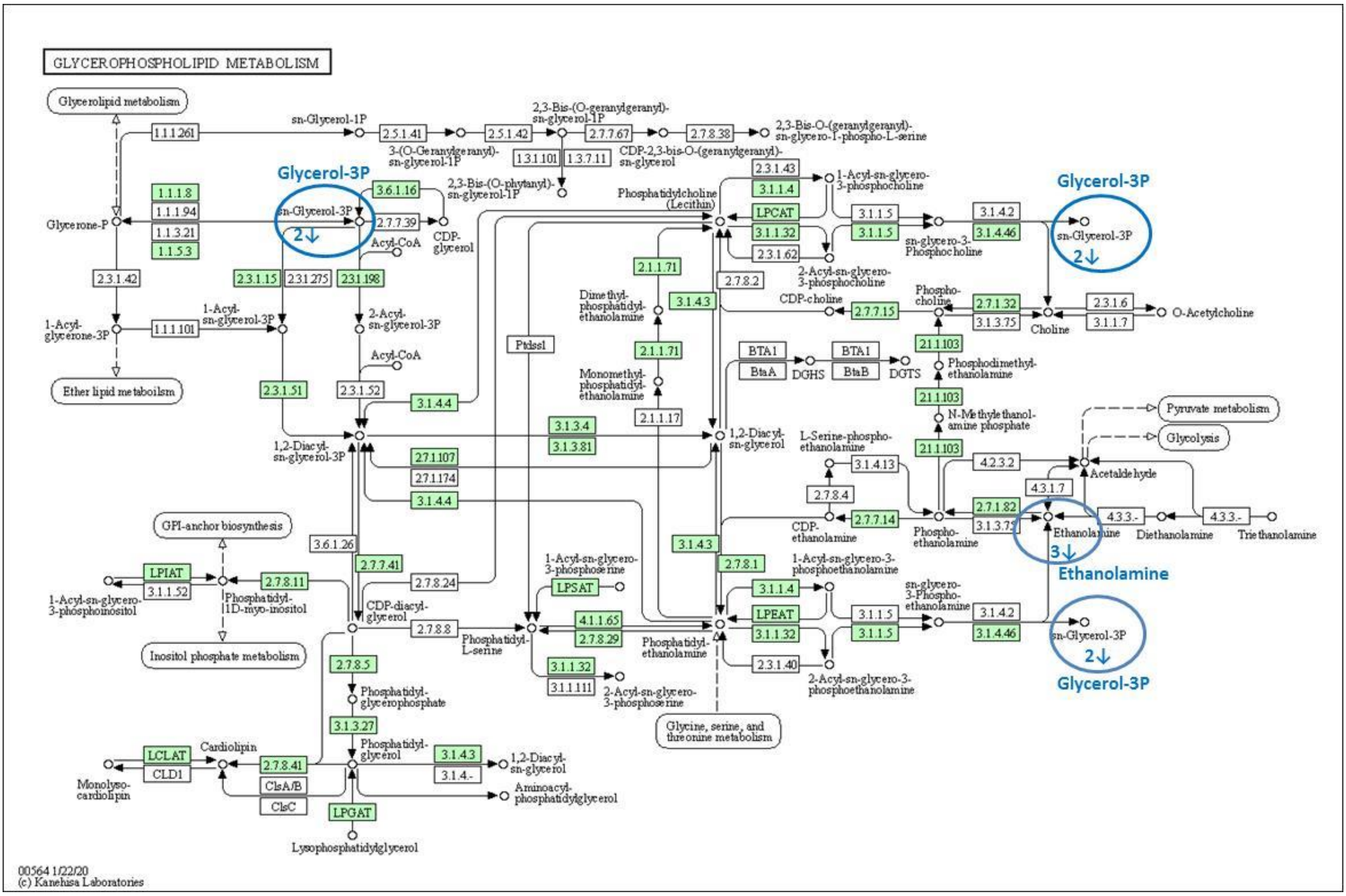

Figure S3.2-12. KEGG scheme 12. Glycerophospholipid metabolism.

Circles mark Zn-related metabolites, red and blue circle colors denote up- and down-regulated metabolites, respectively. Value and arrow in the circles indicate fold and direction of the changes, respectively, in comparison with controls. To address Zn-related metabolites (t-test, $p \leq 0.05$) in roots quantified by untargeted and targeted methods refer to Tables 2 and 3, respectively.

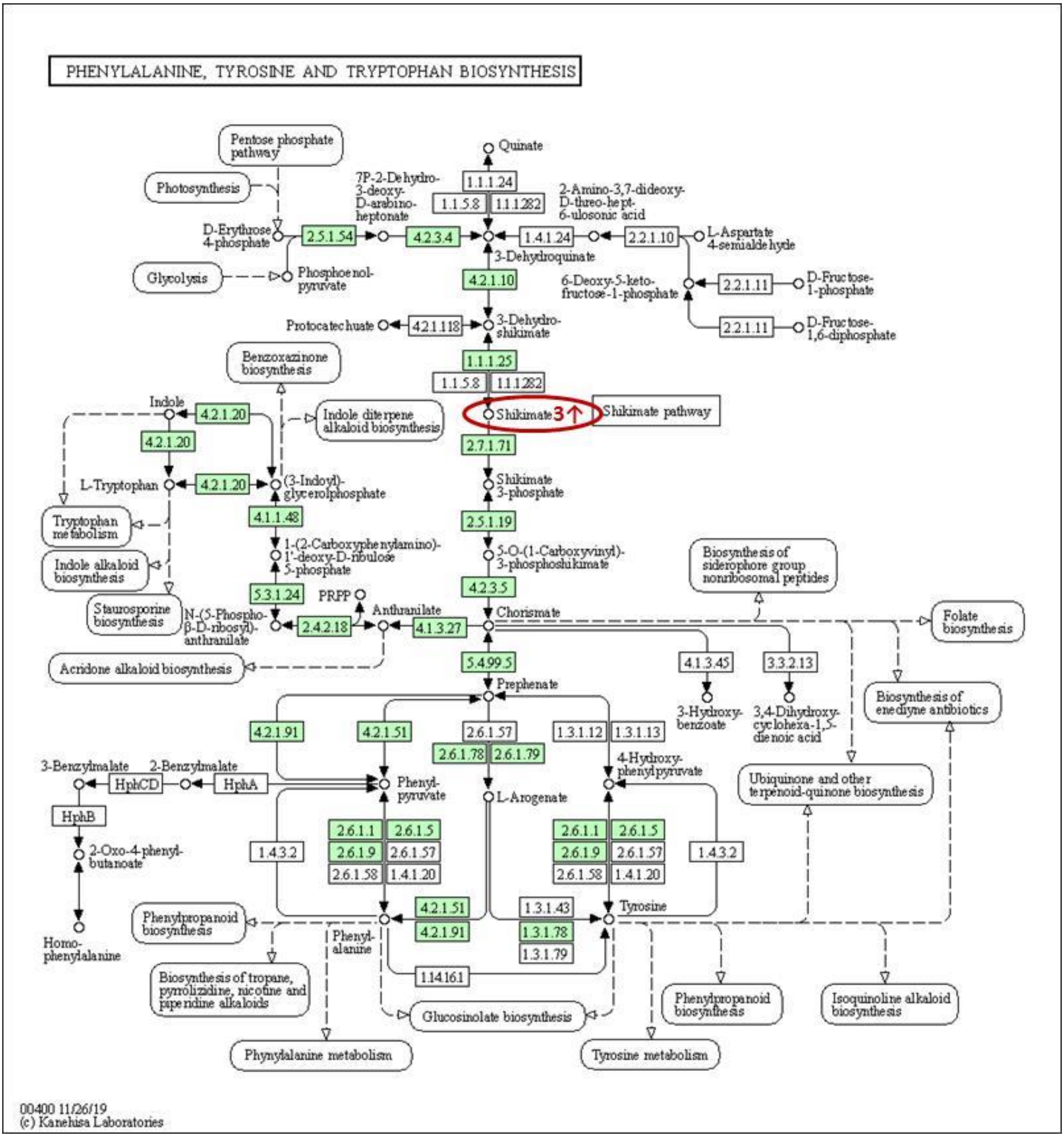

Figure S3.2-13. KEGG scheme 13. Shikimate pathway.

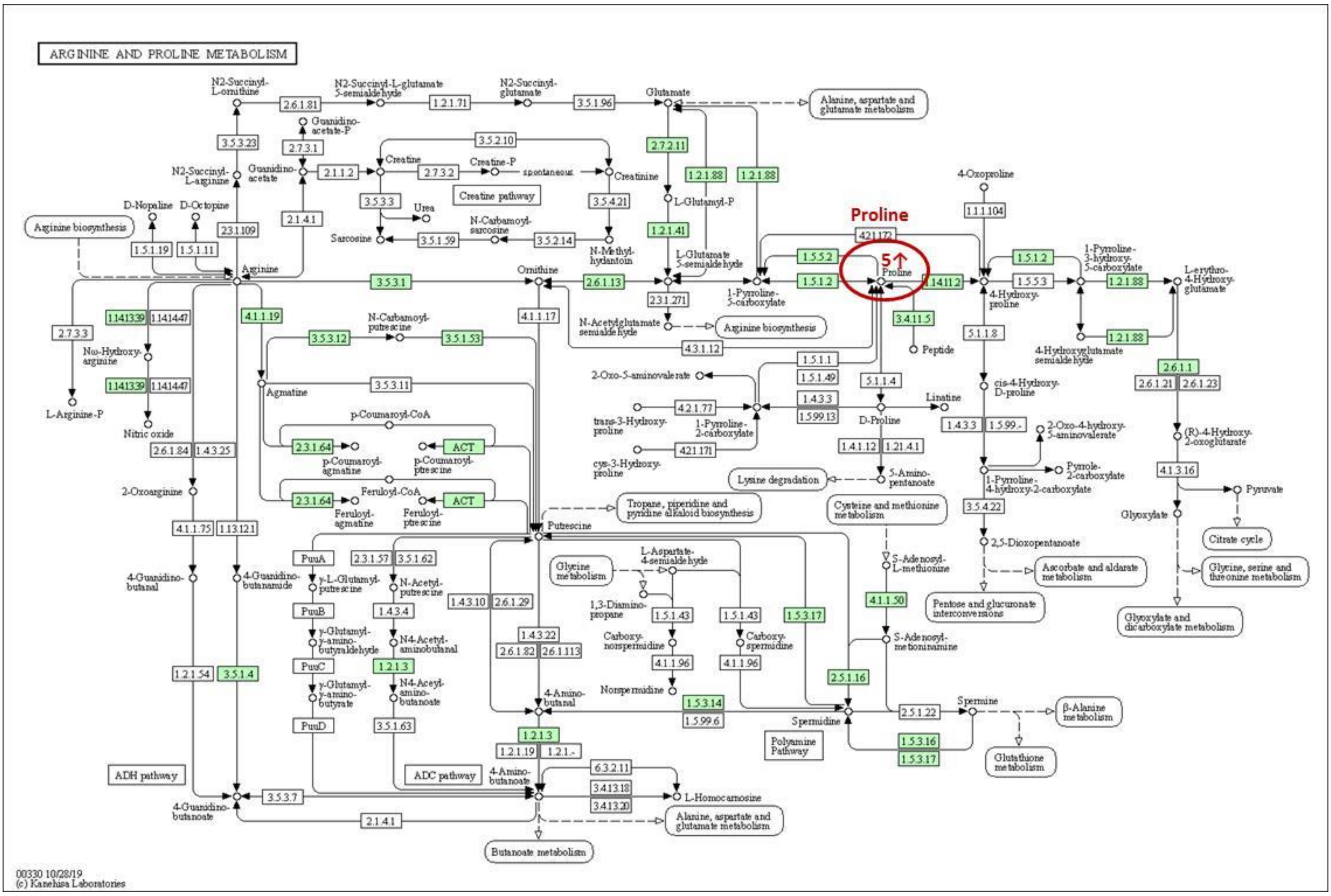

Figure S3.2-14. KEGG scheme 14. Arginine and proline metabolism.

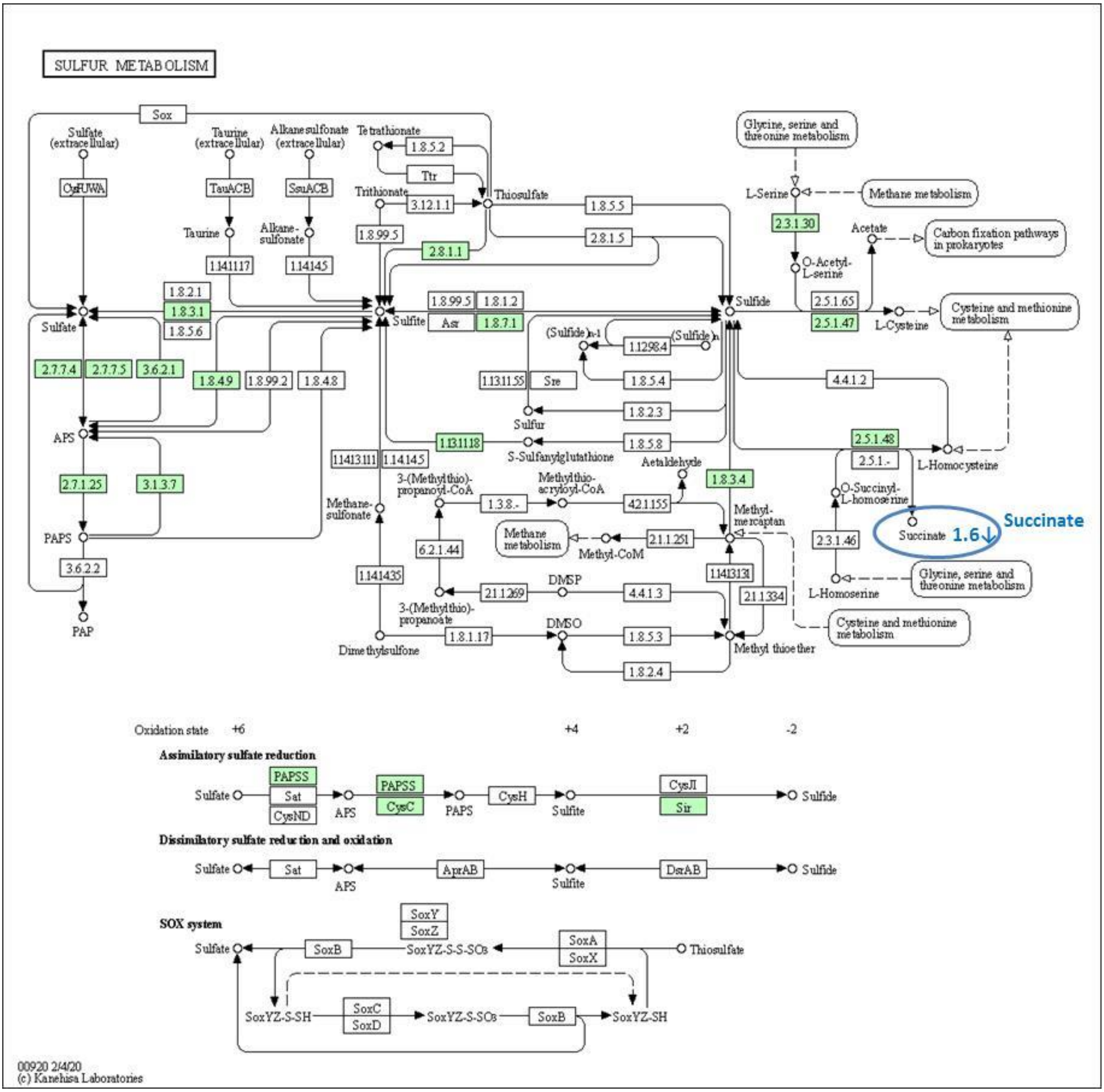

Figure S3.2-15. KEGG scheme 15. Sulfur metabolism.

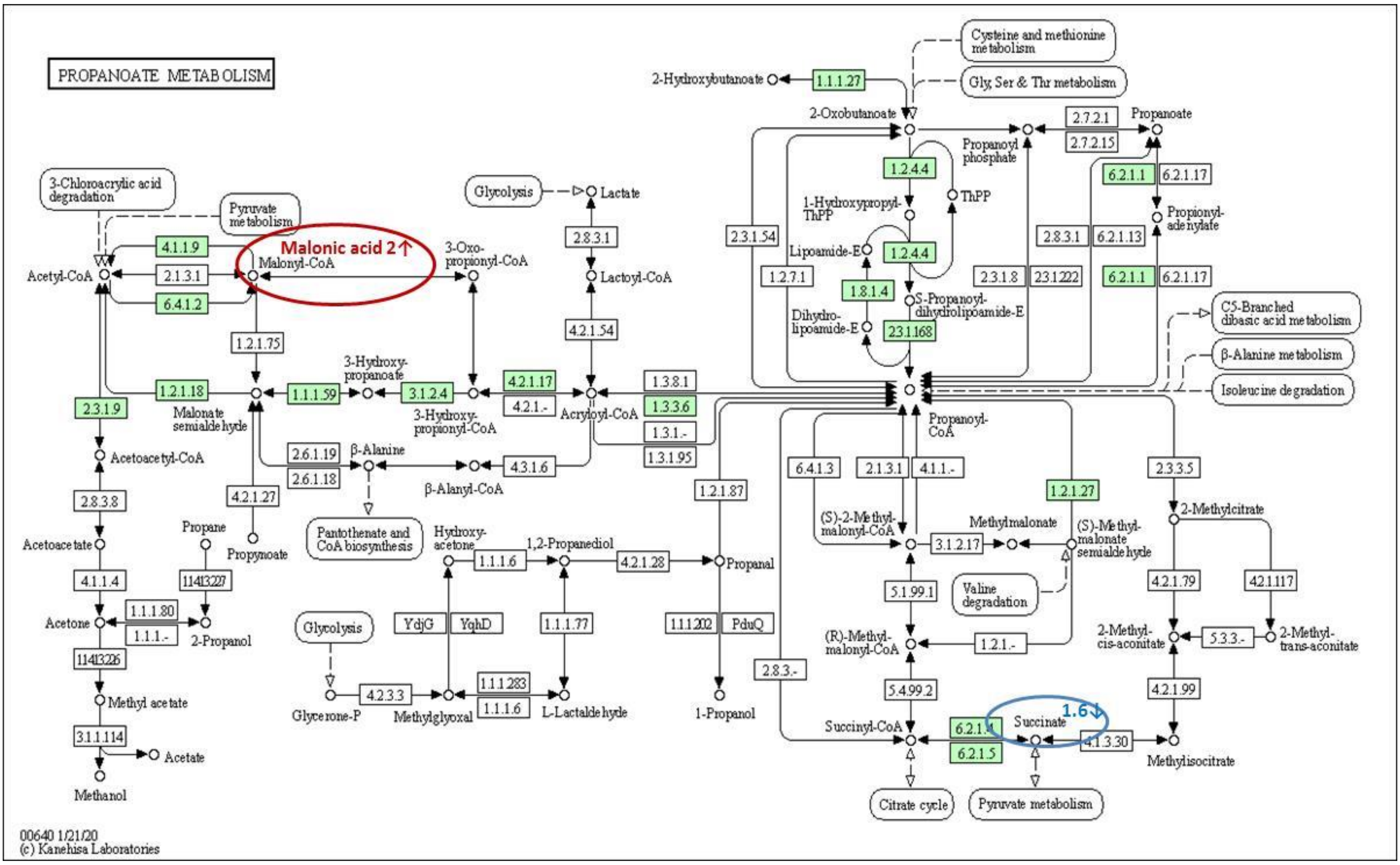

Figure S3.2-16. KEGG scheme 16a. Propanoate metabolism.

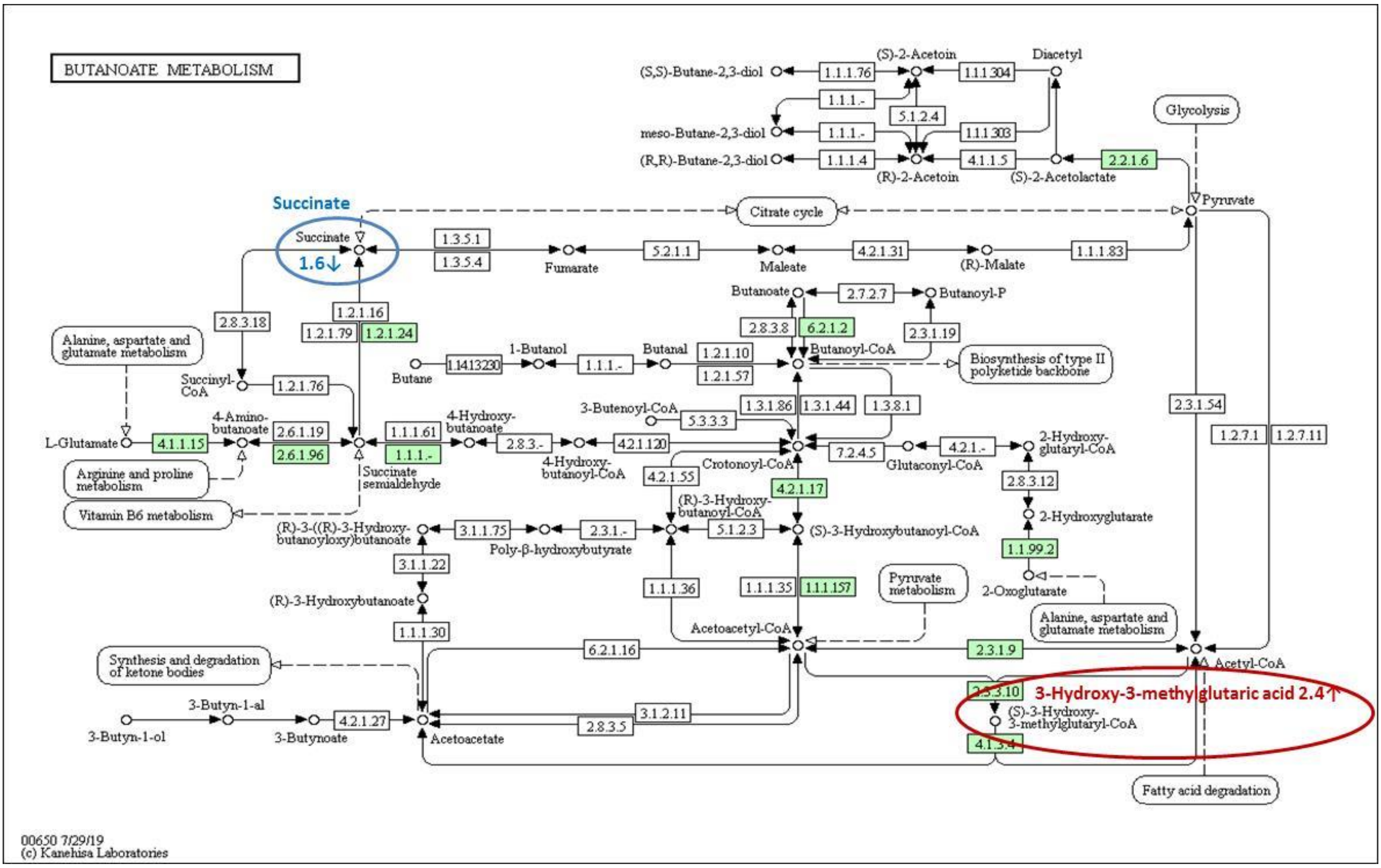

Figure S3.2-17. KEGG scheme 16b. Butanoate metabolism.

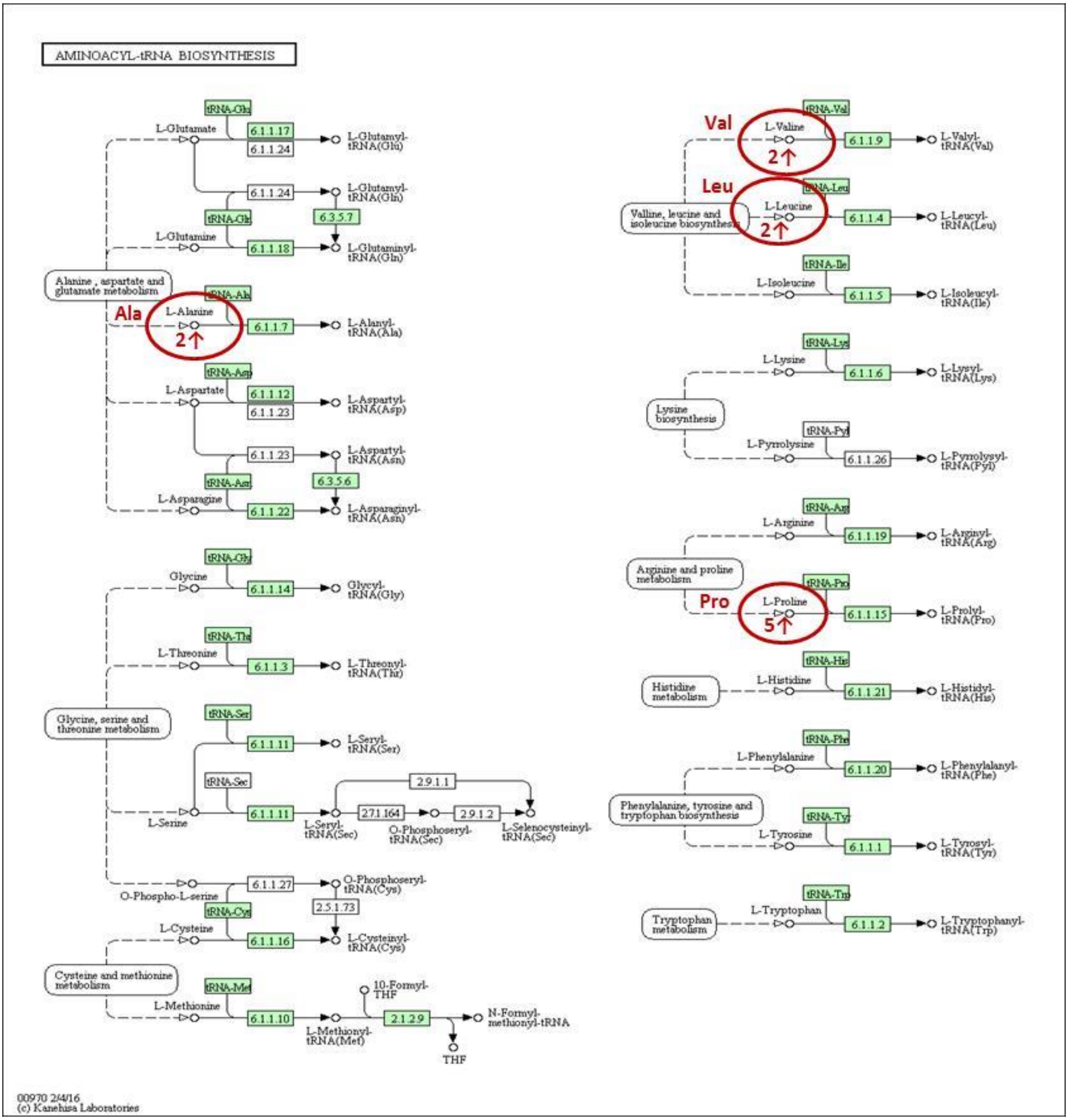

Figure S3.2-18. KEGG scheme 17. Aminoacyl-tRNA biosynthesis.

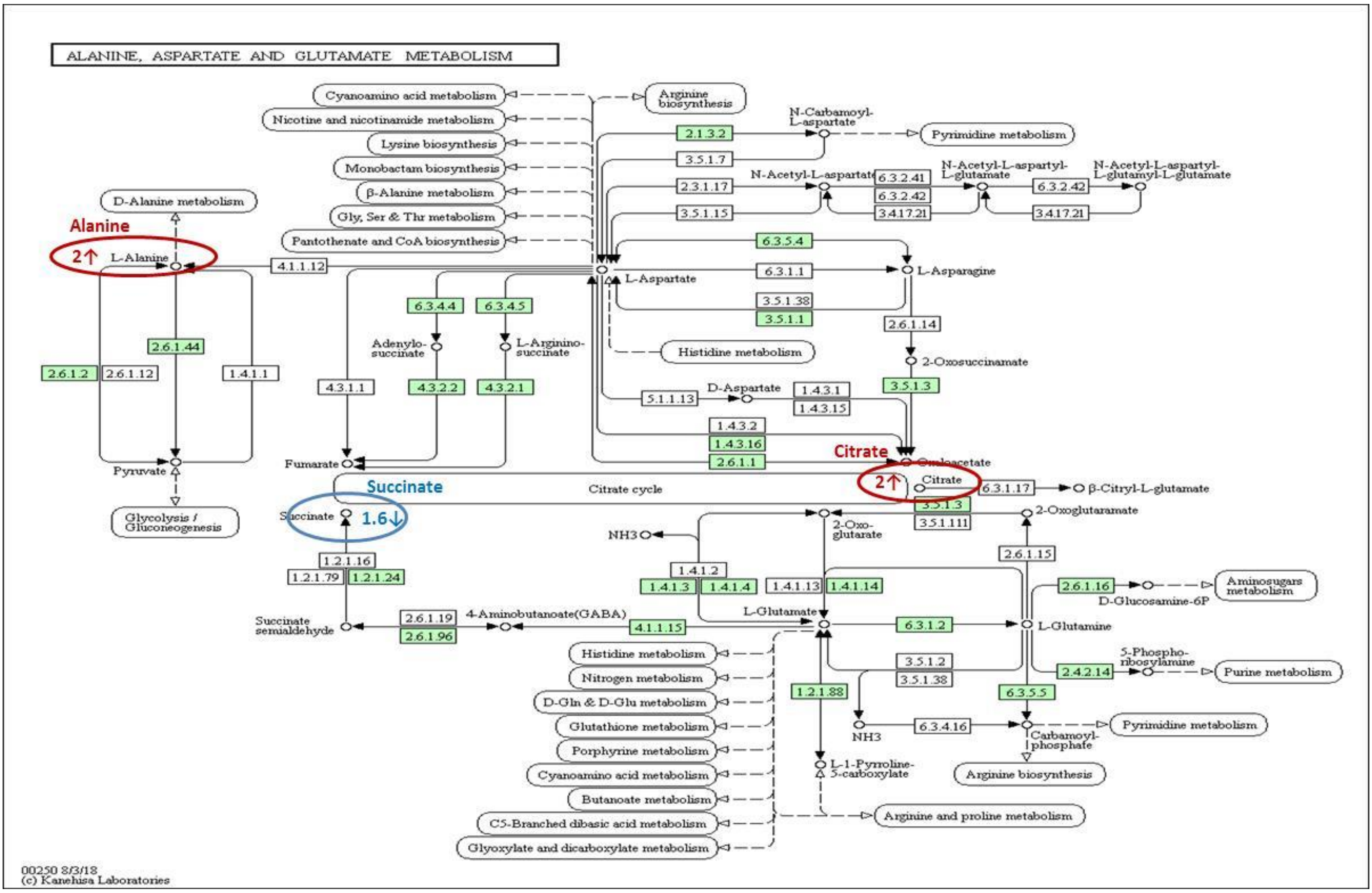

Figure S3.219. KEGG scheme 18. Ala, Asp, Glu metabolism.

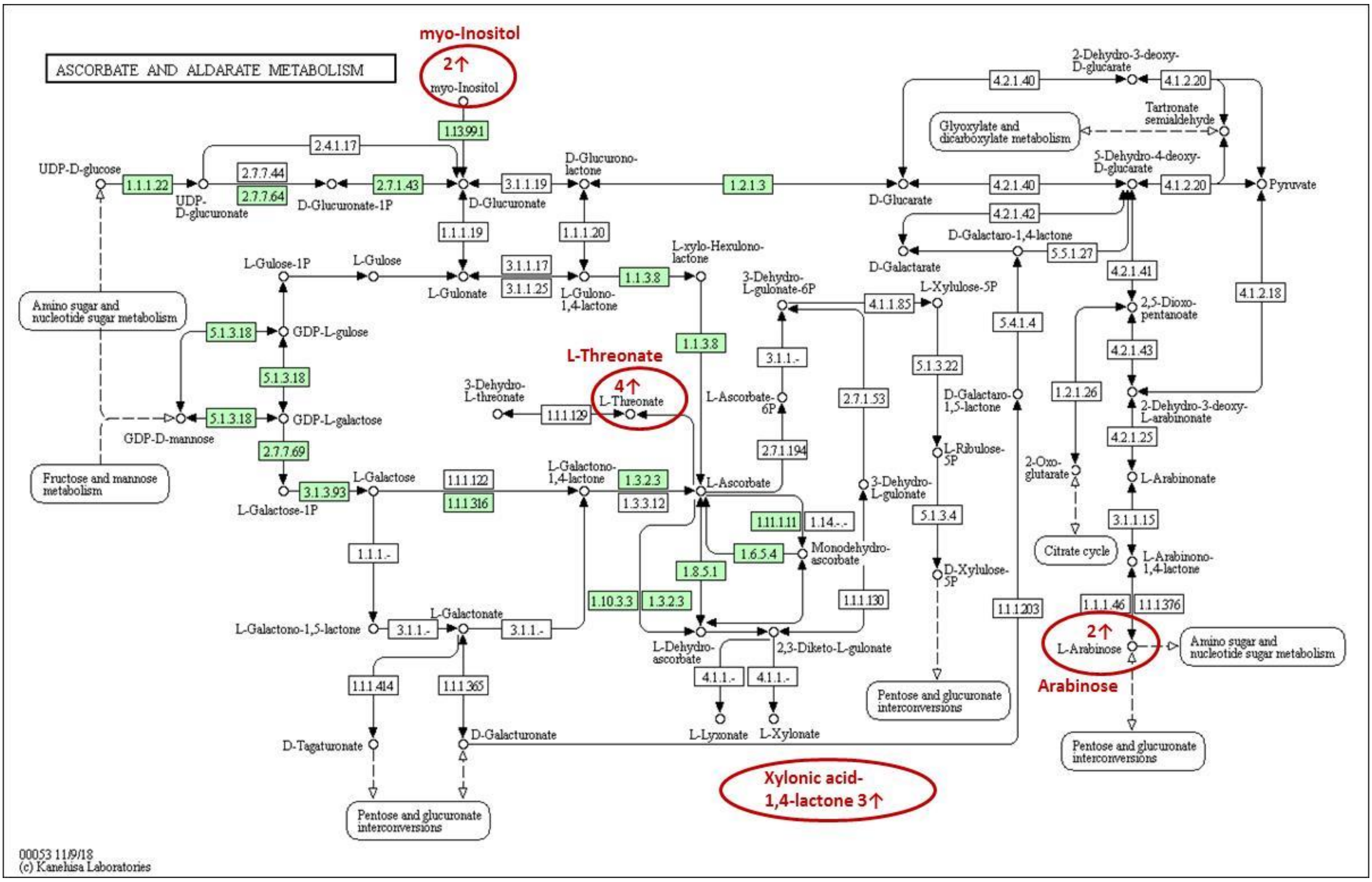

Figure S3.2-20. KEGG scheme 19. Ascorbate and aldarate metabolism.

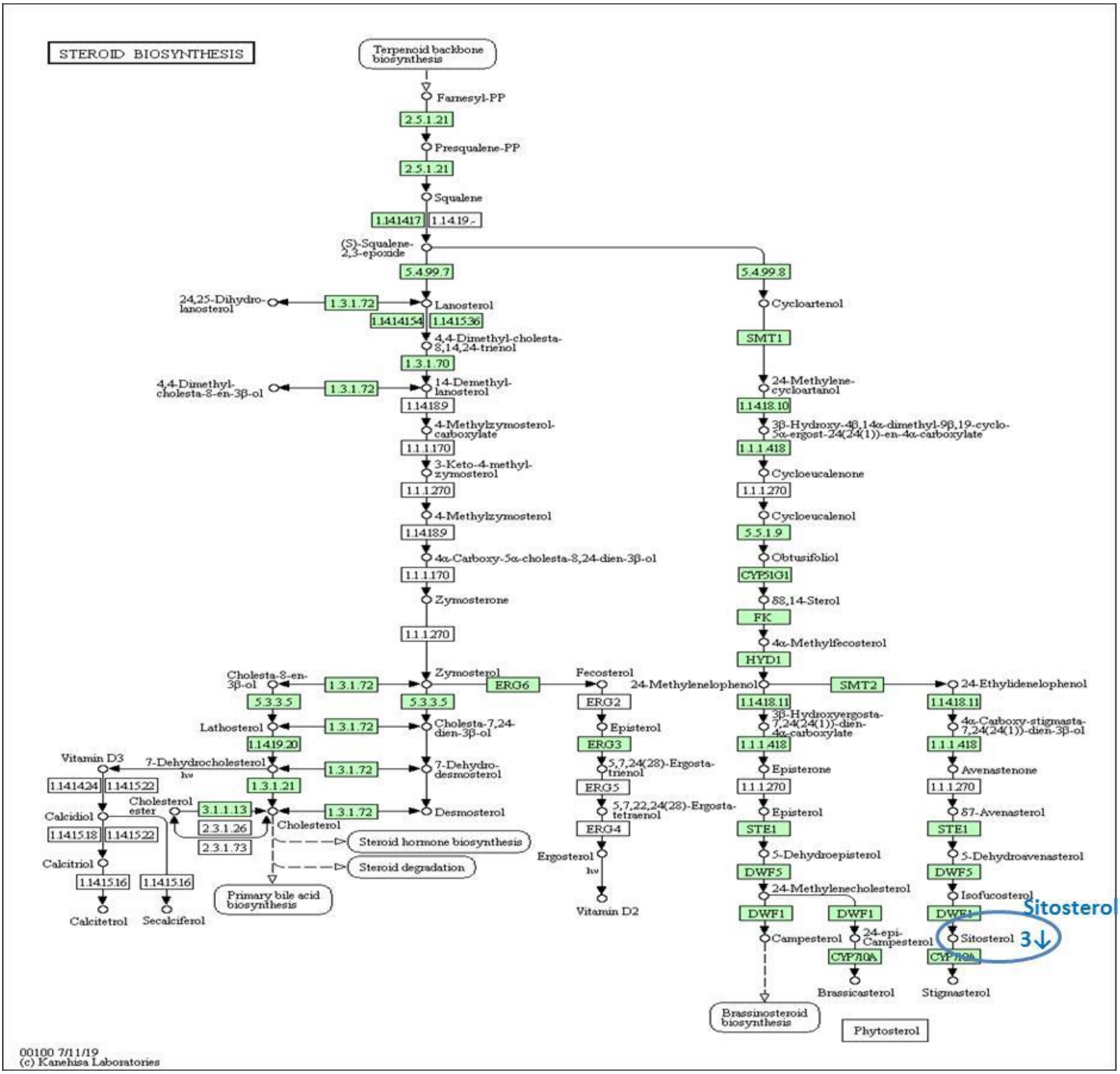

Figure S3.2-21. KEGG scheme 20. Steroid biosynthesis.

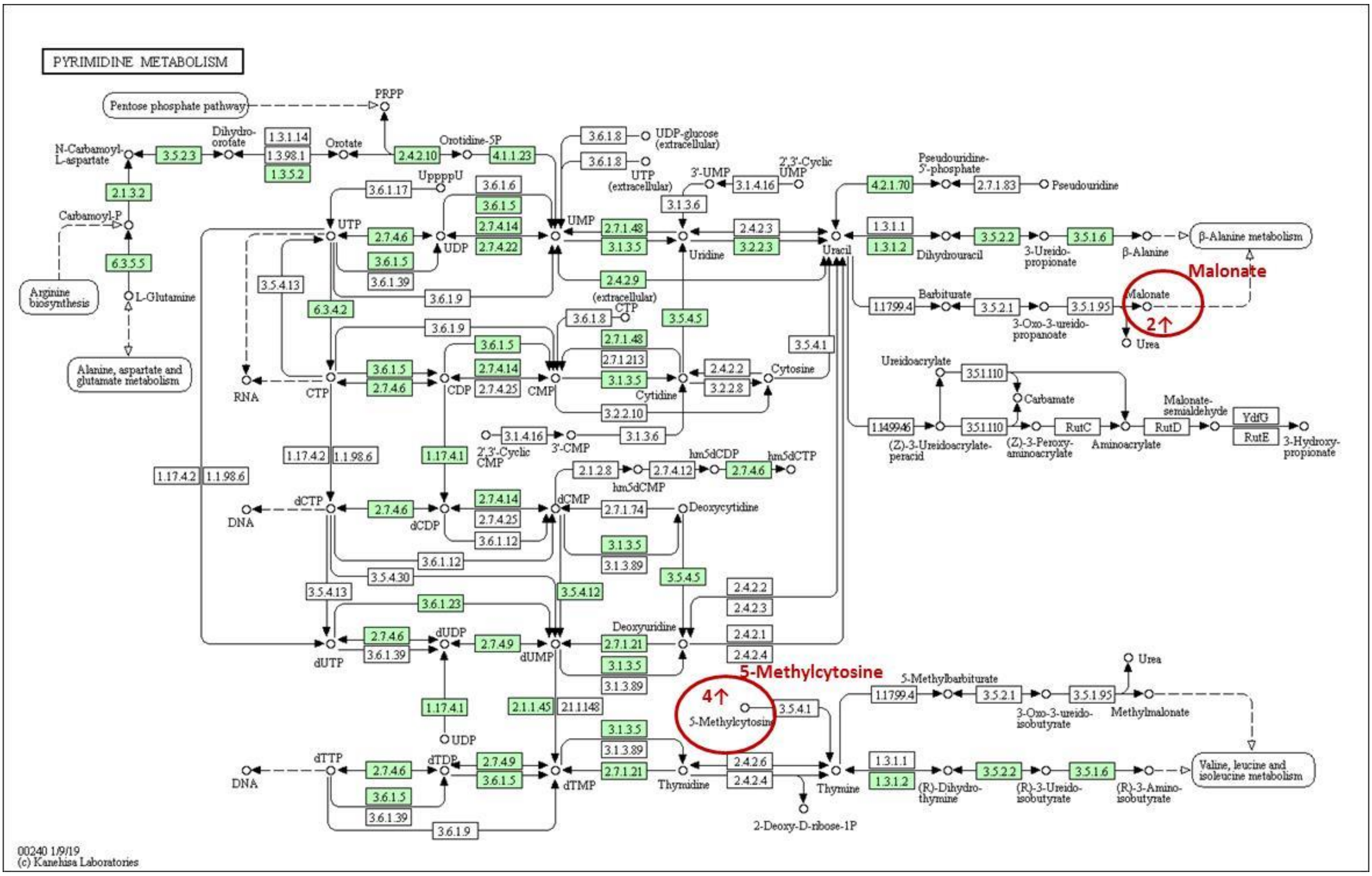

Figure S3.2-22. KEGG scheme 21. Pyrimidine metabolism.

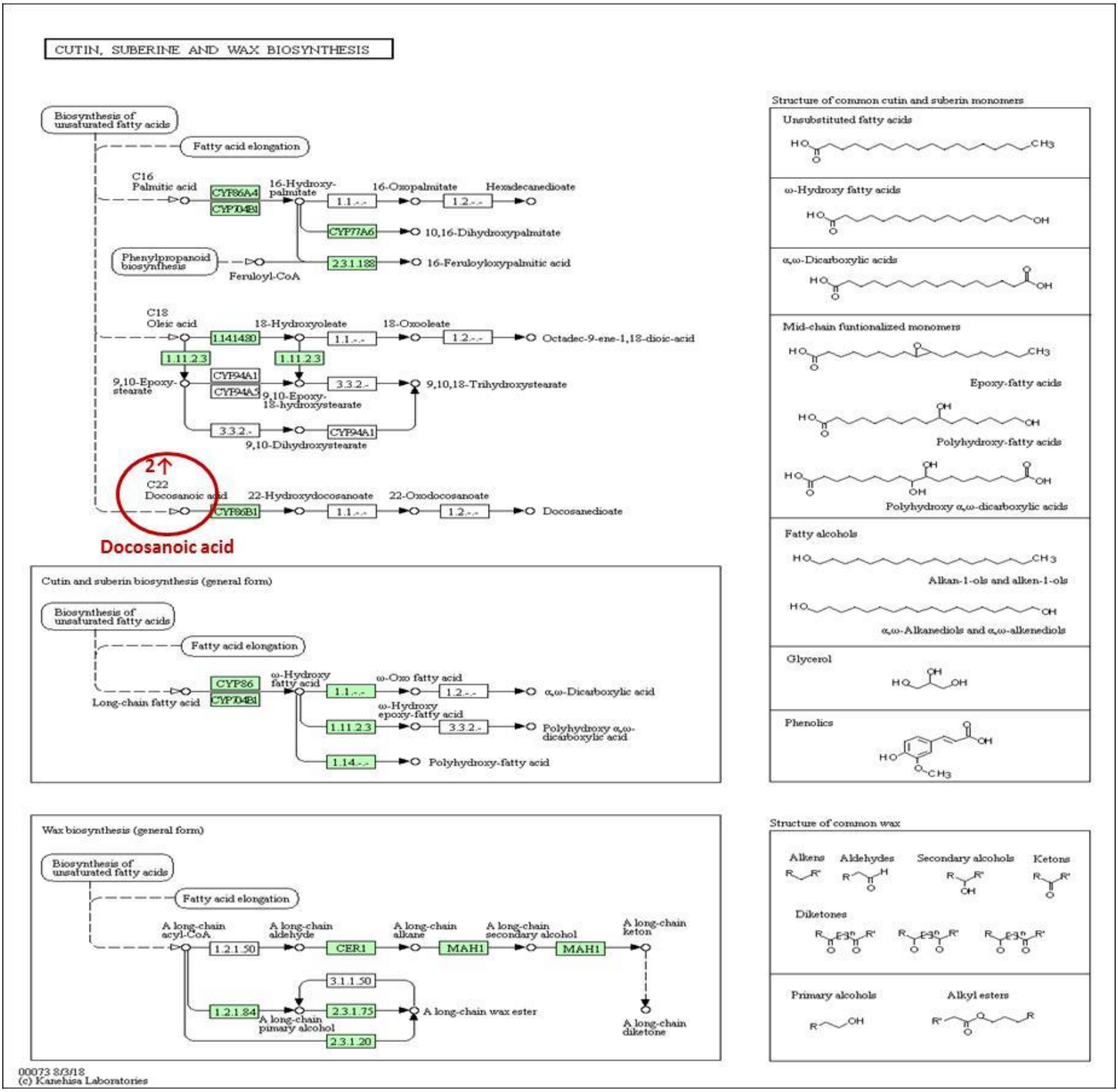

Figure S3.2-23. KEGG scheme 22. Cutin, suberin and wax biosynthesis

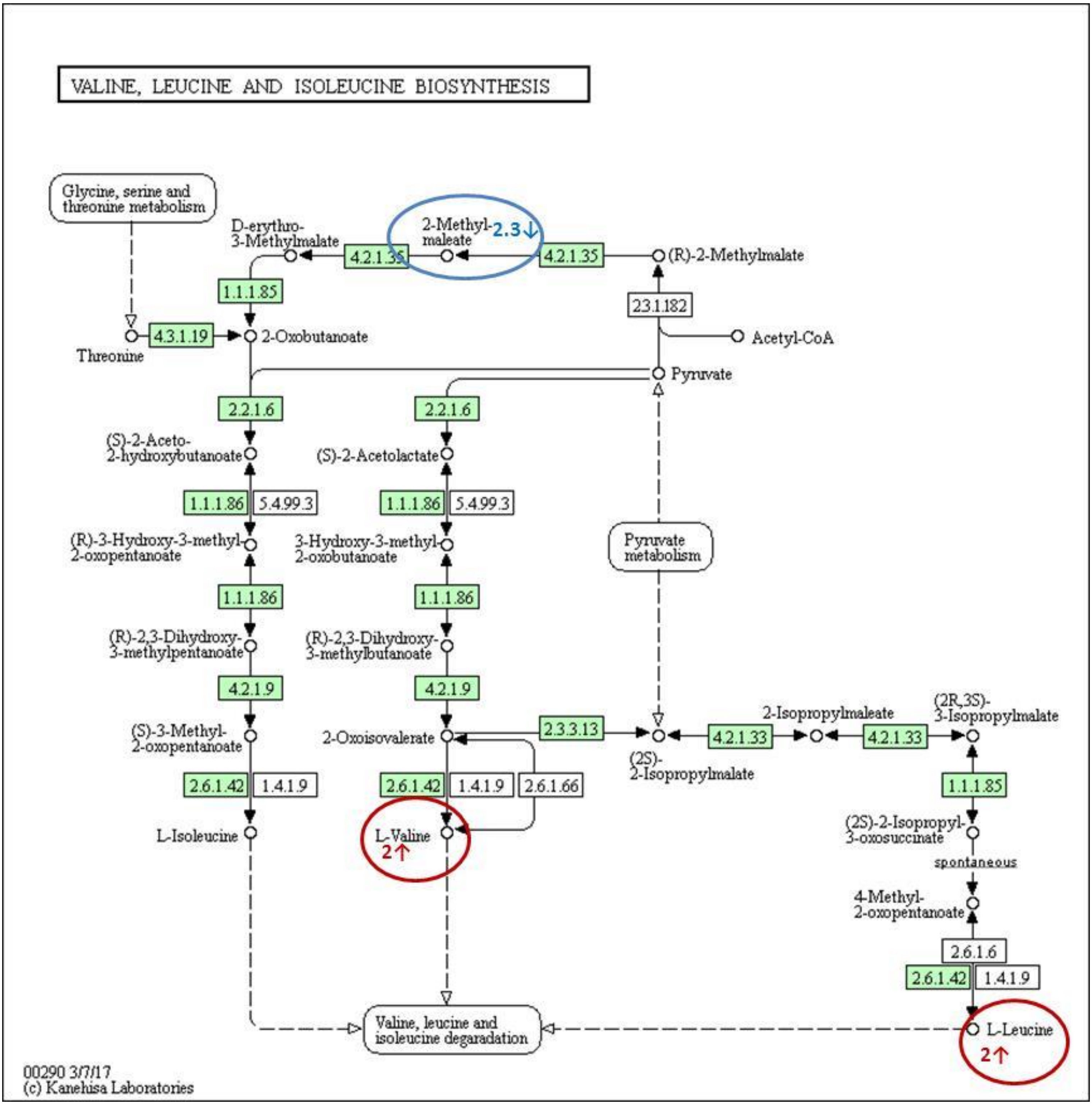

Figure S3.2-24. KEGG scheme 23. Val, Leu, Ile biosynthesis.

Part 3. Metabolic pathways of Pathway Analysis for Zn-regulated metabolites in mature leaves of *A. caudatus*

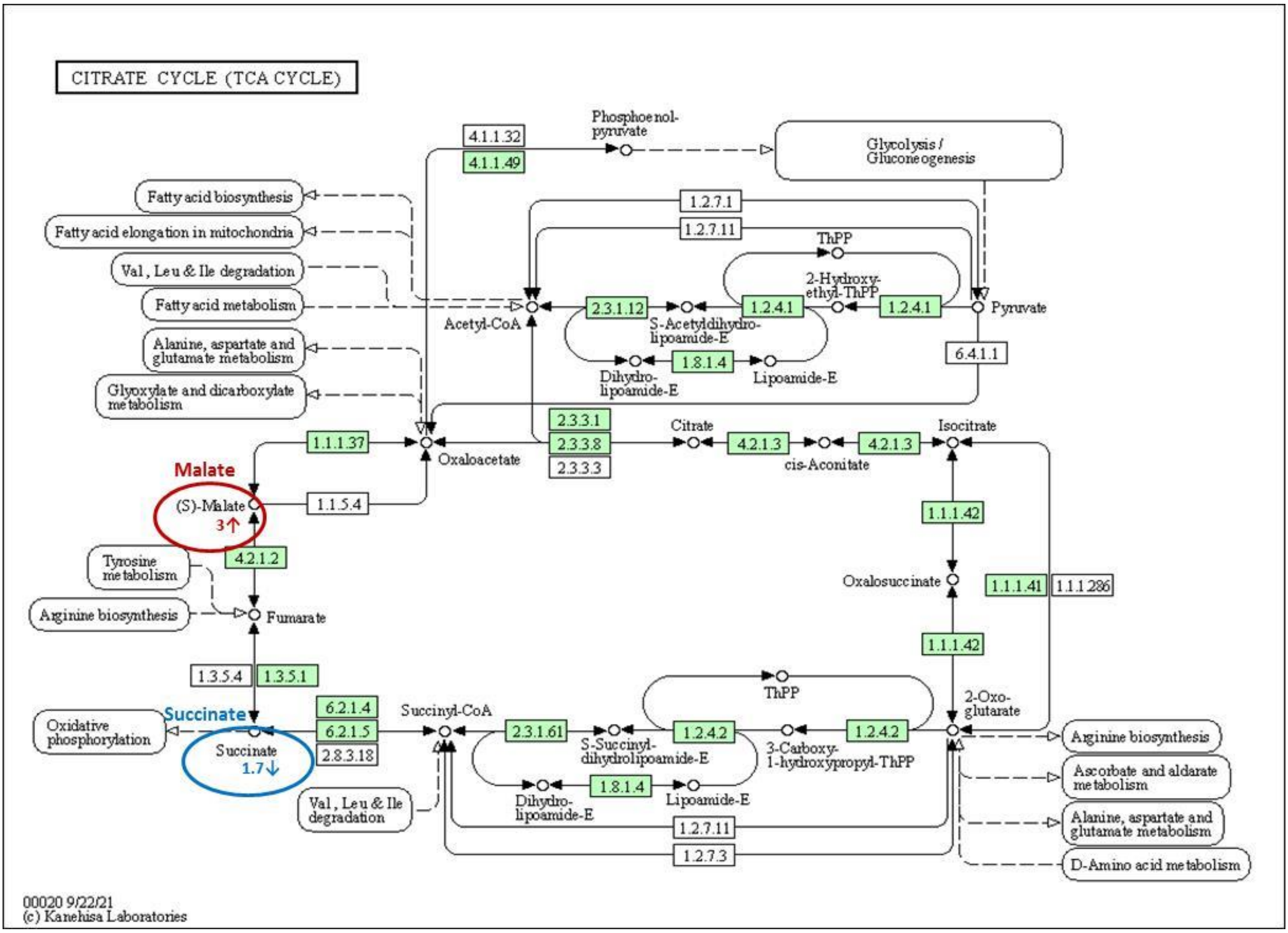

Figure S3.3-1. KEGG scheme 1. Citrate cycle

Circles mark Zn-related metabolites, red and blue circle colors denote up- and down-regulated metabolites, respectively. Value and arrow in the circles indicate fold and direction of the changes, respectively, in comparison with controls. To address Zn-related metabolites (t-test, $p \leq 0.05$) in mature leaves quantified by untargeted and targeted methods refer to Supplementary information 1, Tables S1-8 and S1-9, respectively.

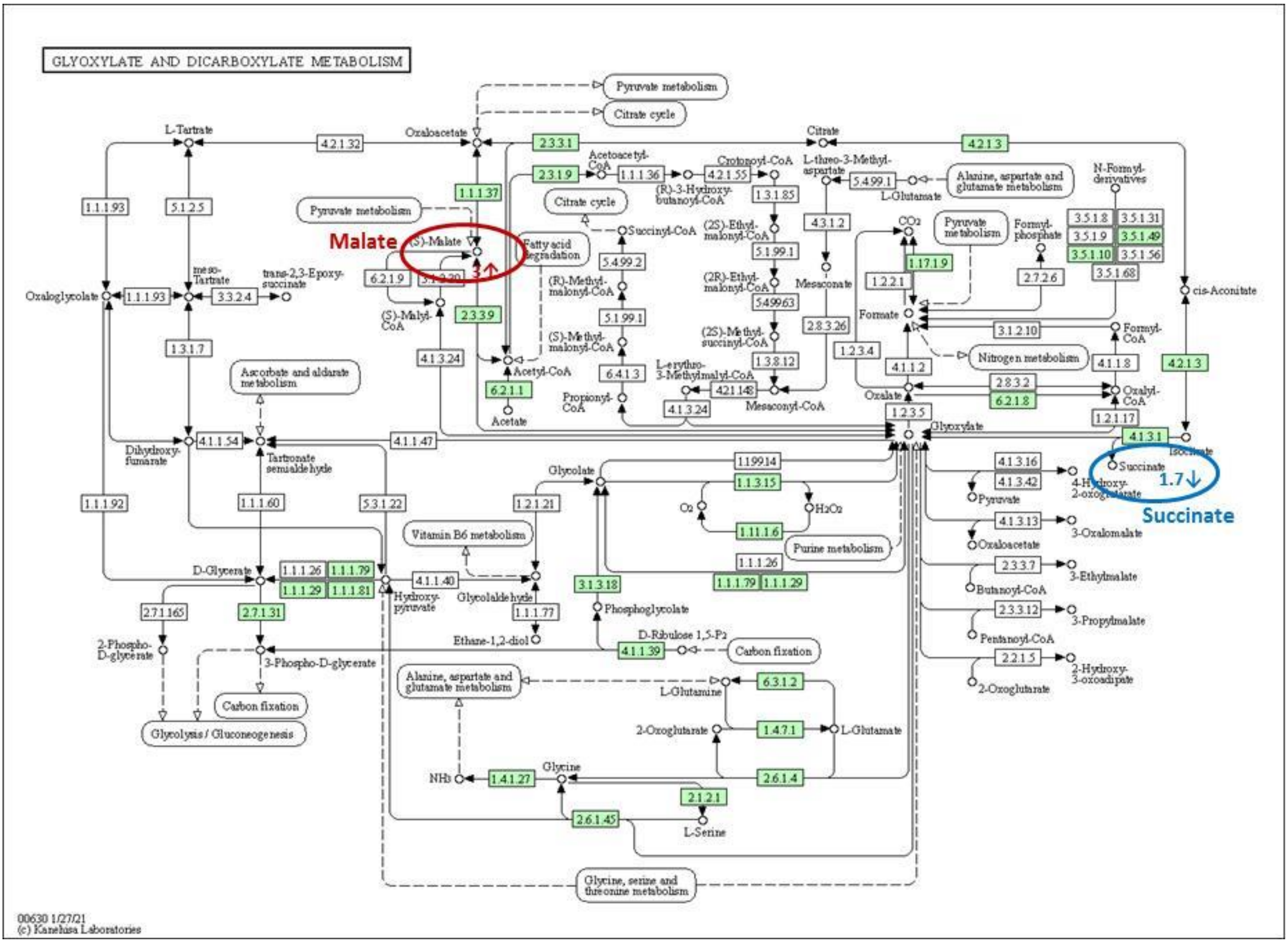

Figure S3.3-2. KEGG scheme 2. Glyoxylate and Dicarboxylate metabolism.

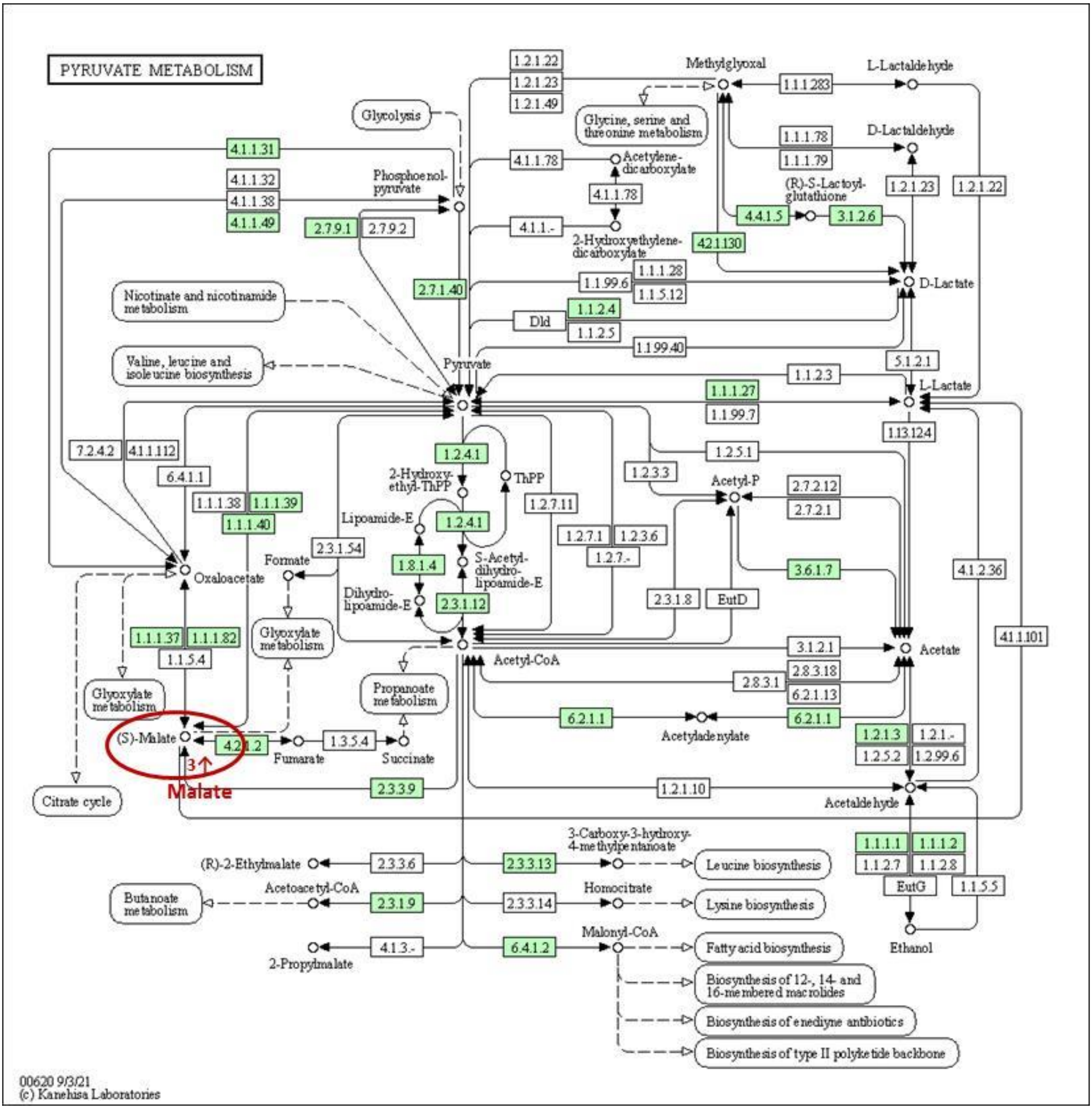

Figure S3.3-3. KEGG scheme 3. Pyruvate metabolism.

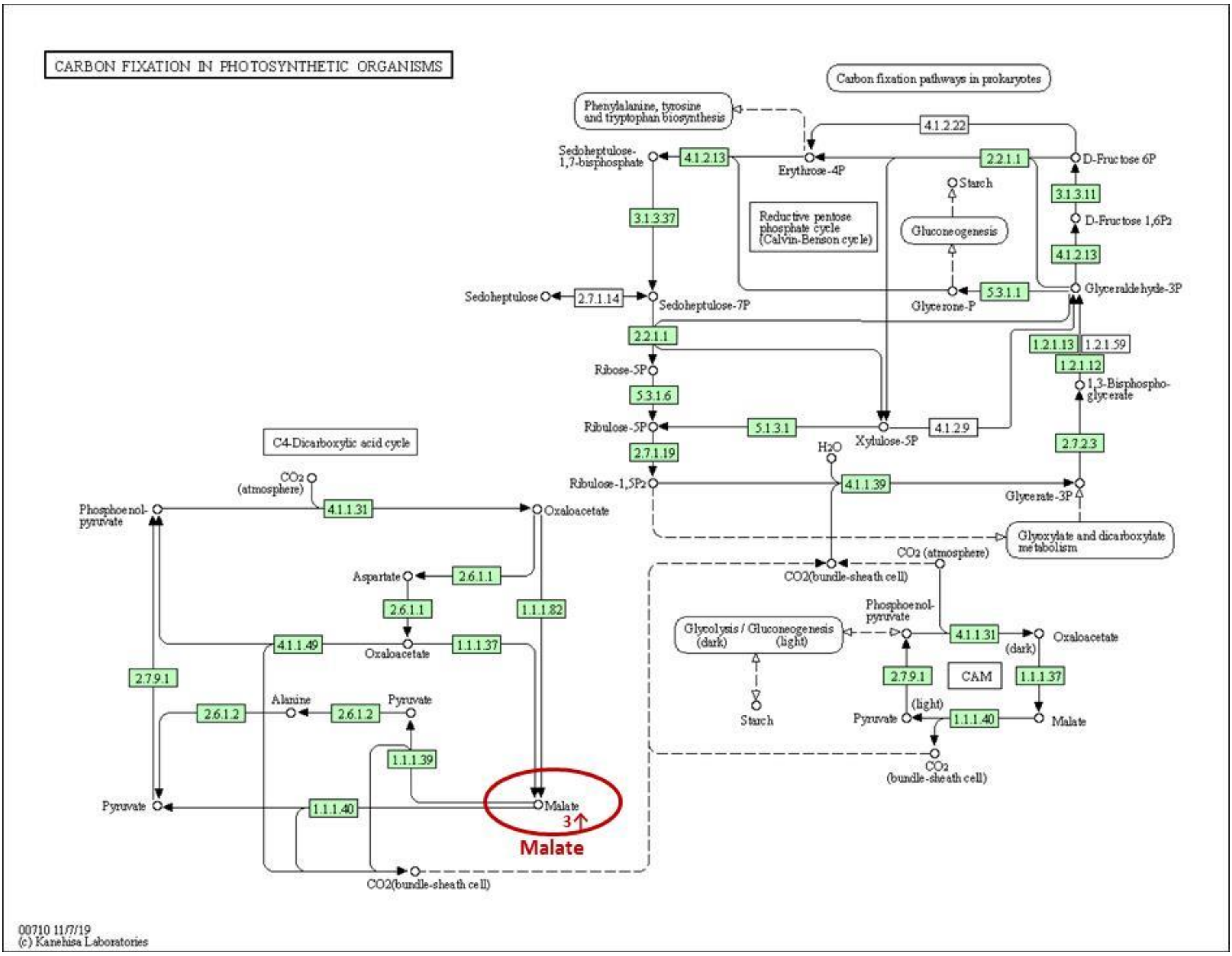

Figure S3.3-4. KEGG scheme 4. Carbon fixation.

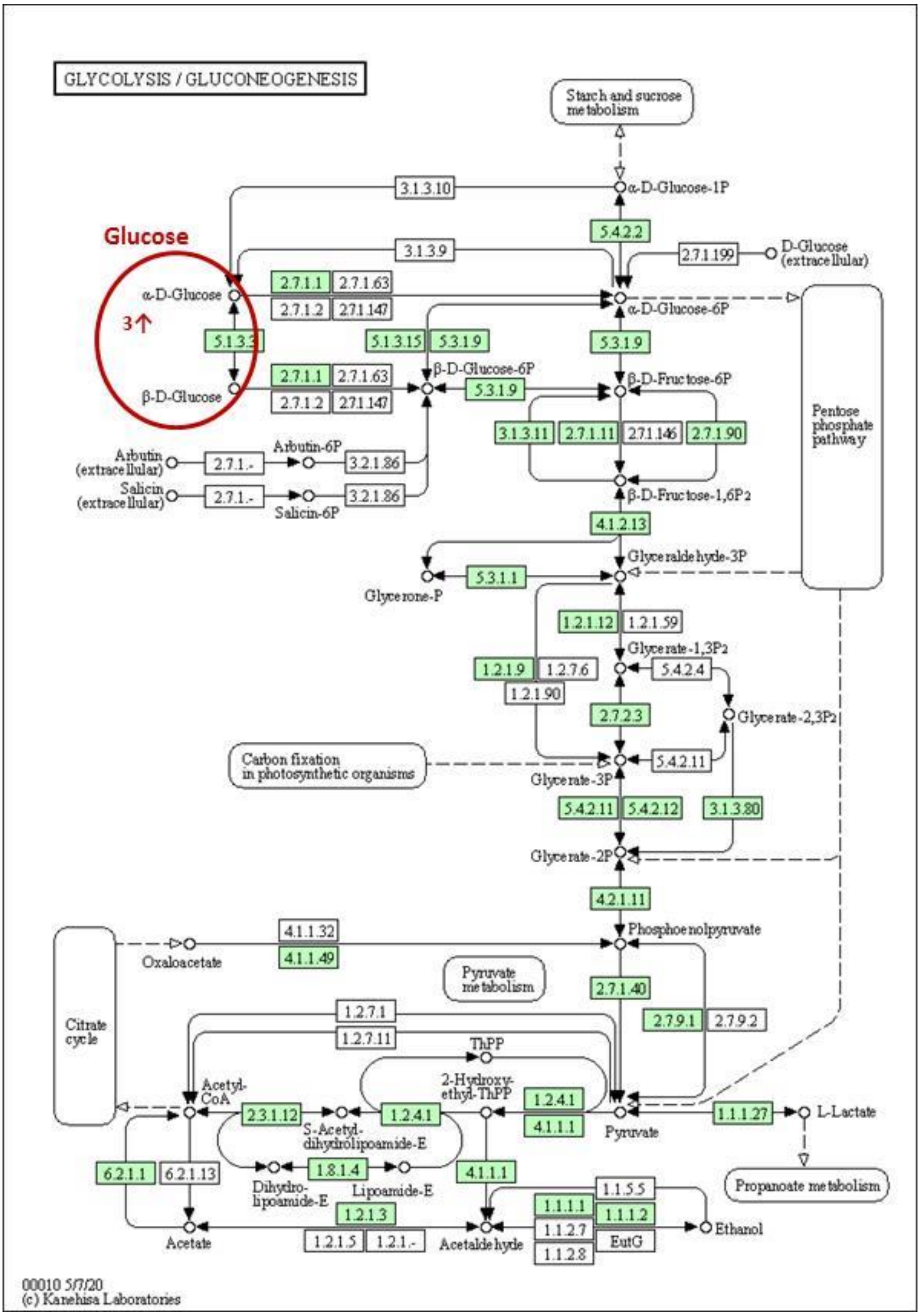

Figure S3.3-5. KEGG scheme 5. Glycolysis/Gluconeogenesis.

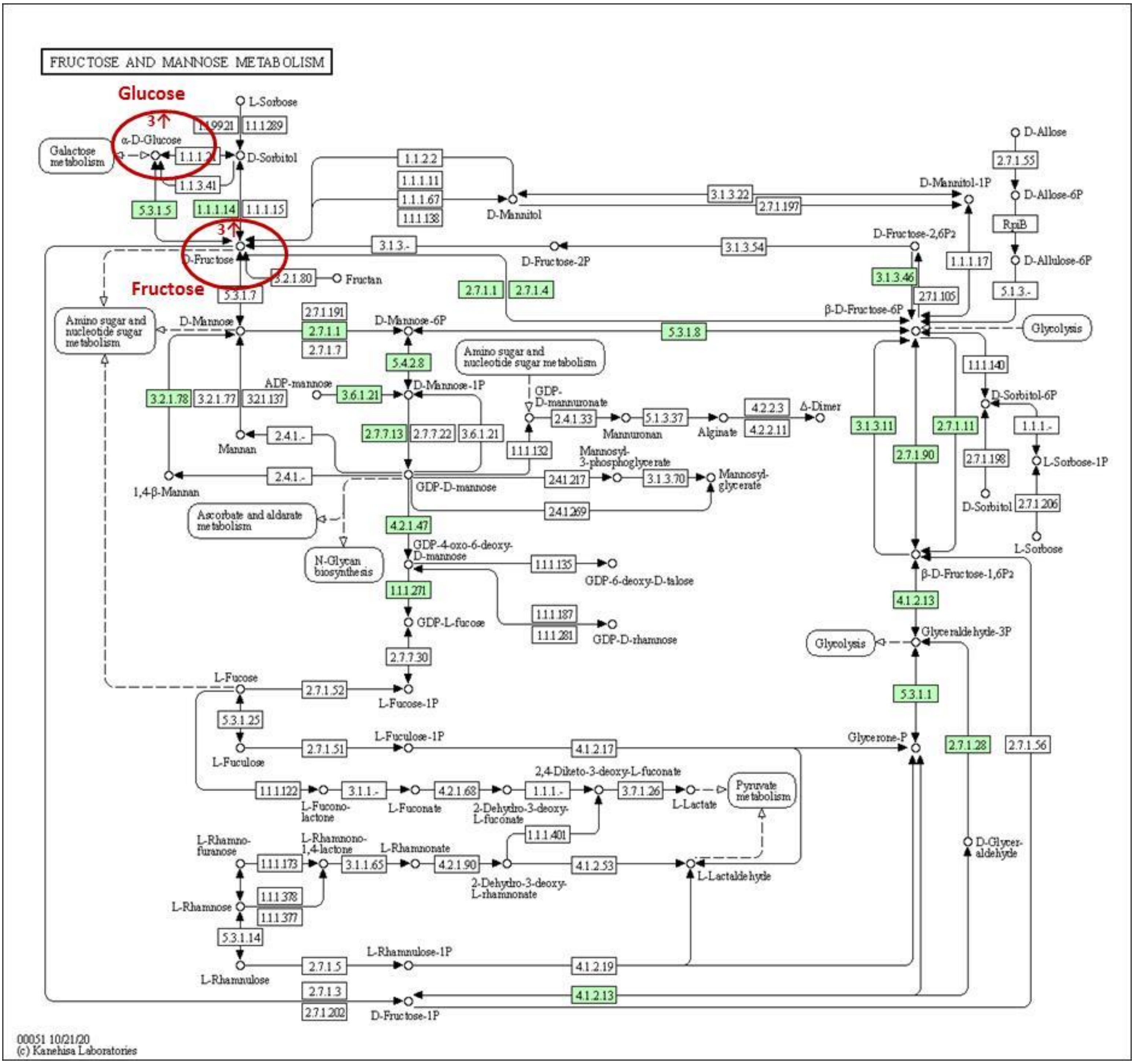

Figure S3.3-6. KEGG scheme 6. Fructose and mannose metabolism.

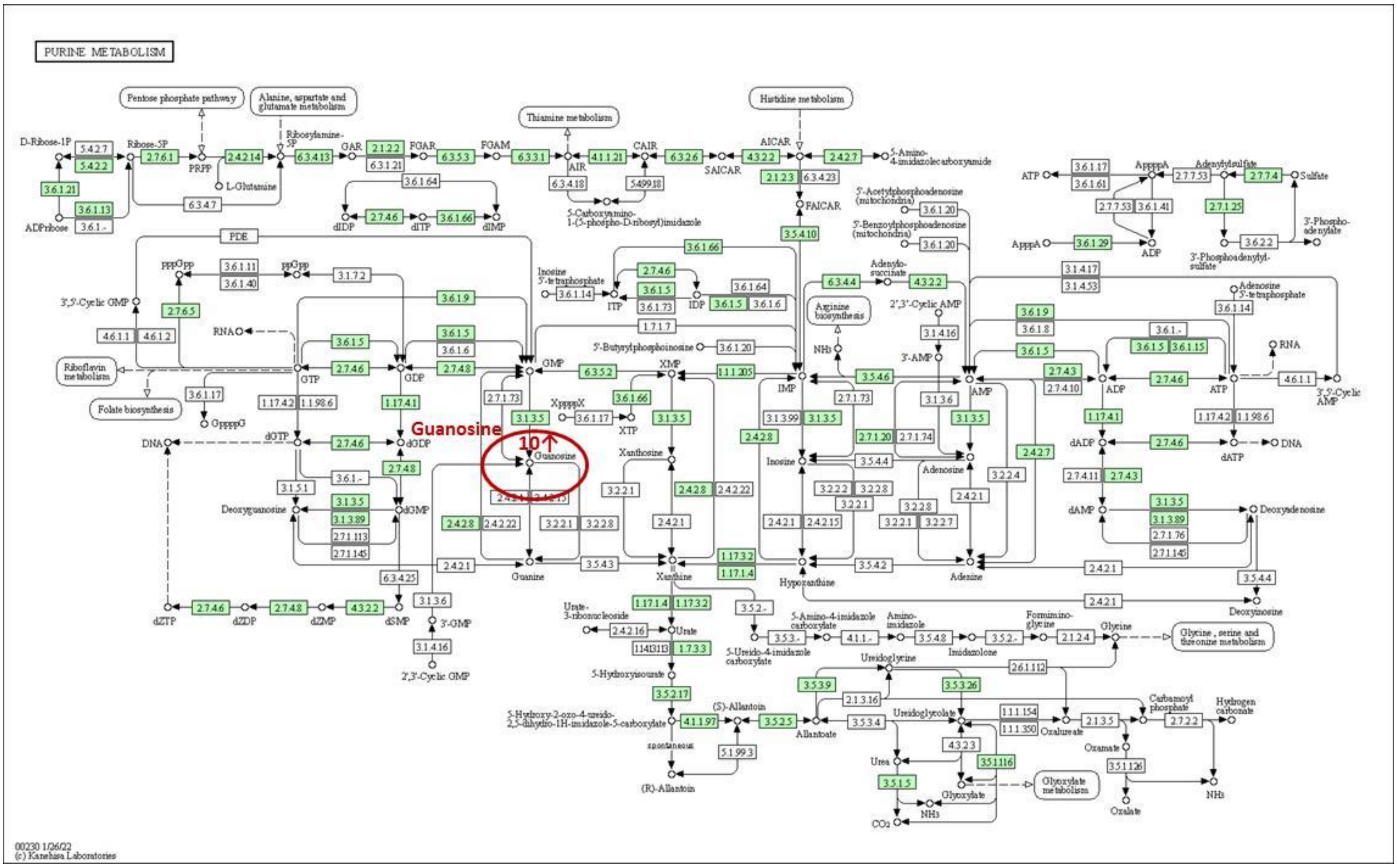

Figure S3.3-7. KEGG scheme 7. Purine metabolism.

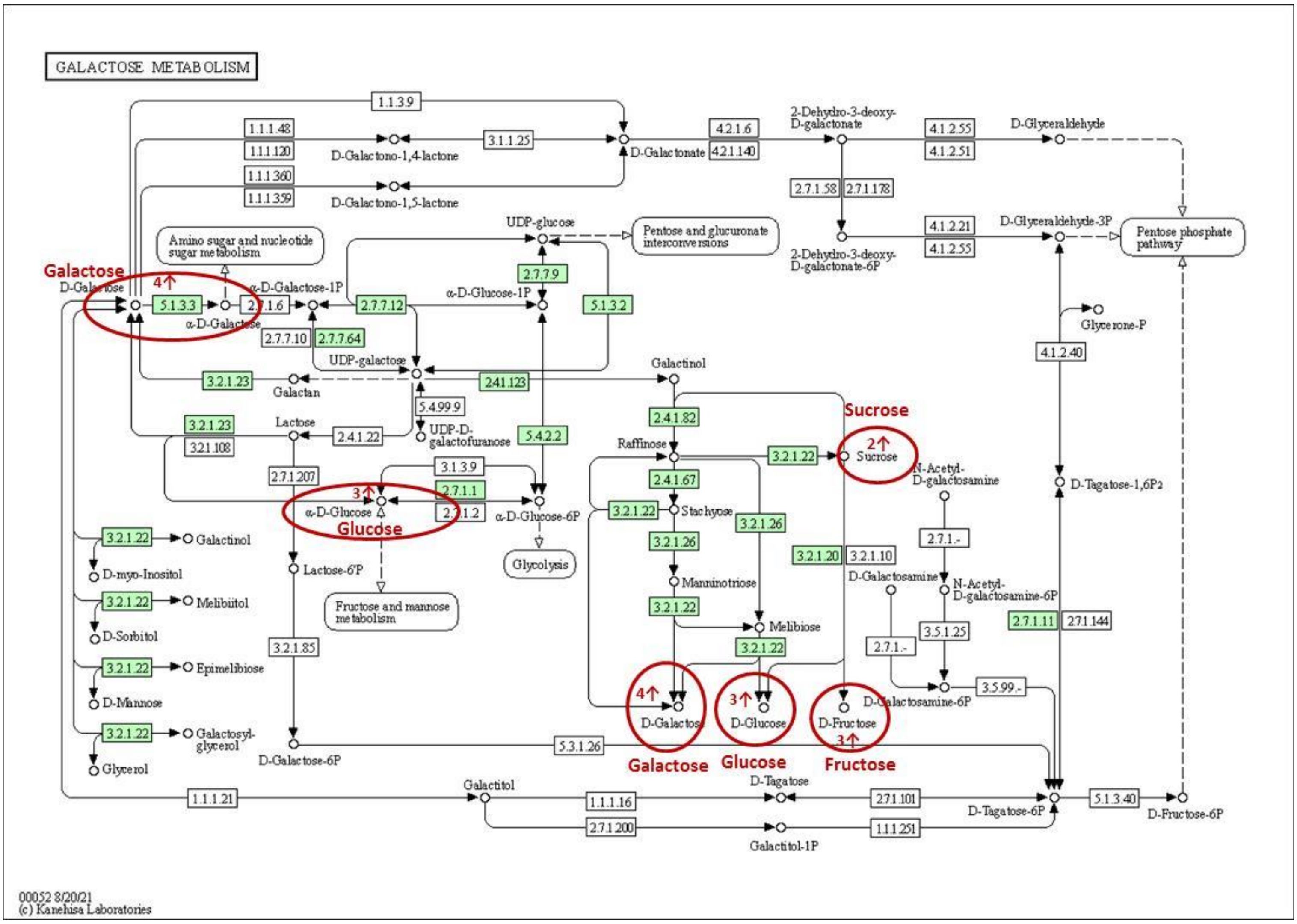

Figure S3.3-8. KEGG scheme 8. Galactose metabolism.

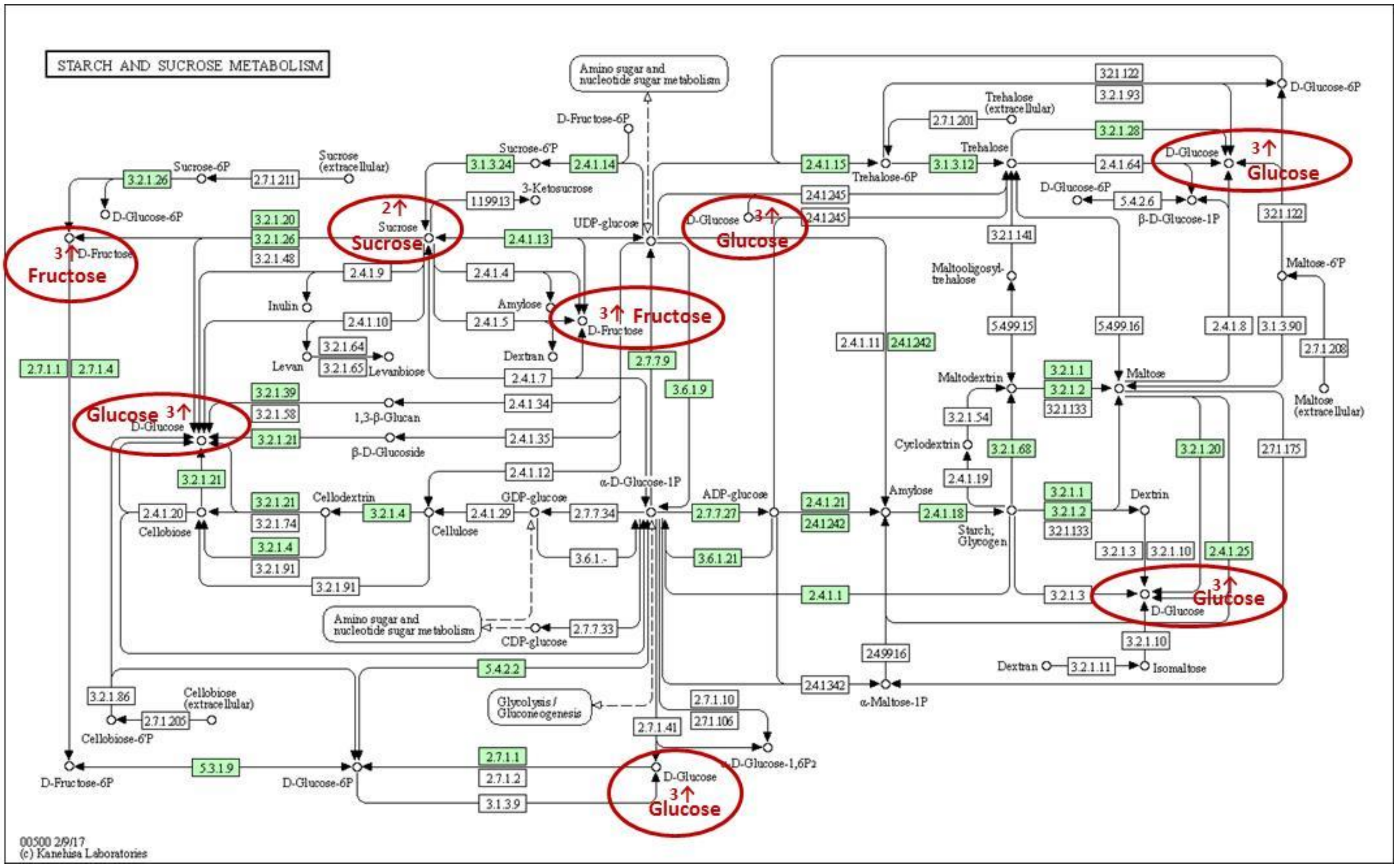

Figure S3.3-9. KEGG scheme 9. Starch and sucrose metabolism.

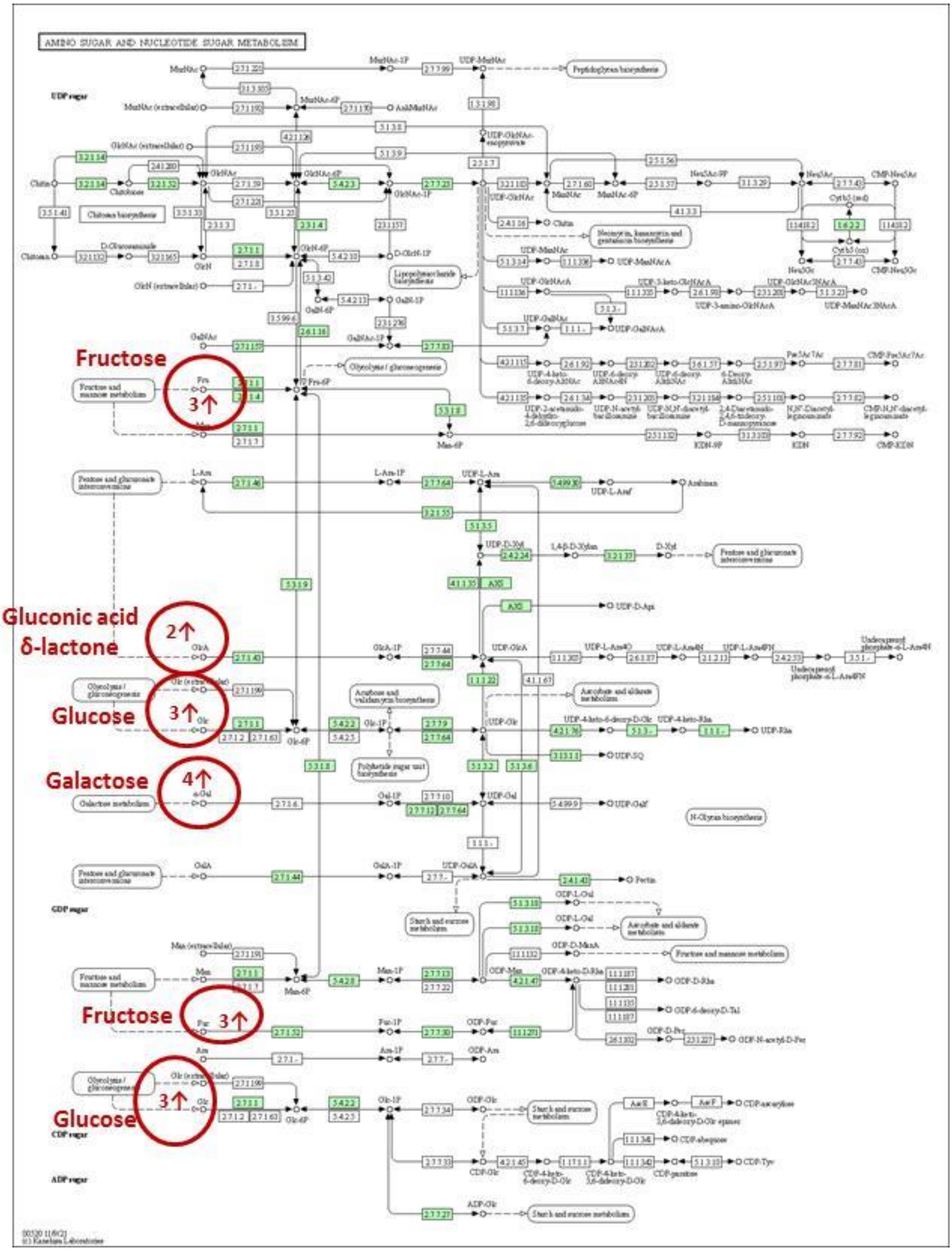

Figure S3.3-10. KEGG scheme 10. Amino sugar and nucleotide sugar metabolism.
