# Supplementary material for: Metabolic Responses of Amaranthus caudatus Roots and Leaves to Zinc Stress
Source: Plants (Basel). 2025 Jul 9;14(14):2119. doi: 10.3390/plants14142119 (PMC12300844; doi:10.3390/plants14142119)
Supplement: Supplementary file 1 [file plants-14-02119-s001.zip › plants-3701072_Osmolovskaya_et_al_Supplementary_S1(Protocol_Figures_Tables)_revised.pdf]

Metabolic responses of *Amaranthus caudatus* roots and leaves to zinc stress

Natalia Osmolovskaya [1,†,*], Tatiana Bilova [1,2,†], Anastasia Gurina [1], Anastasia Orlova [2], Viet D. Vu [1,3], Stanislav Sukhikh [4], Tatiana Zhilkina [5], Nadezhda Frolova [2,*], Elena Tarakhovskaya [1,6], Anastasia Kamionskaya [5] and Andrej Frolov [2]

Supplementary Information 1

[1] Department of Plant Physiology and Biochemistry, St. Petersburg State University, 199034 St. Petersburg, Russia;

[2] Laboratory of Analytical Biochemistry and Biotechnology, K.A. Timiryazev Institute of Plant Physiology of the Russian Academy of Science, 127276 Moscow, Russia;

[3] Coast Branch - Vietnam Russian Tropical Center, Khanh Hoa, Nha Trang City, Vietnam;

[4] Laboratory of Microbiology and Biotechnology, Immanuel Kant Baltic Federal University, 236041, Kaliningrad, Russia;

[5] Federal Research Centre Fundamentals of Biotechnology of the Russian Academy of Science, 119071 Moscow, Russia;

[6] Vavilov Institute of General Genetics, St. Petersburg Branch, Russian Academy of Sciences, 199034 St. Petersburg, Russia.

[†]These authors contributed equally to the manuscript

*Corresponding authors:

Dr. Nadezhda Frolova

Laboratory of Analytical Biochemistry and Biotechnology, K.A. Timiryazev Institute of Plant Physiology of the Russian Academy of Science, 127276 Moscow, Russia

Tel. +7 (499) 678-54-00

Email: frolovanadja@yandex.ru

Dr. Natalia Osmolovskaya

St. Petersburg State University

Department of Plant Physiology and Biochemistry, Universitetskaya nab. 7/9, 199034, St Petersburg, Russia

Tel. +7 (812) 3289695

Email: natalia_osm@mail.ru

Directory

Protocols

Protocol S1-1. Annotation algoritm applied to elucidate structure of di- and oligosaccharides based on the EI mass spectra and RI……………………………………………………………………………4

Protocol S1-2. Composition of nutrient solution……………………………………………………6

Protocol S1-3. Plant sample preparation (extraction and derivatization) for gas chromatography-mass spectrometry analysis………………………………………………………………………..……7

Figures

Figure S1(1). Shoots and roots of seven week-old *A.caudatus* plants grown in the **first independent experiment**………………………………………………………………………………………..8

Figure S1(2). Shoots and roots of seven week-old *A.caudatus* plants grown in the **second independent experiment**…………………………………………………………………..…………..…10

Figure S1(3). Impact of exogenous Zn^{2+} on physiological parametrs (stomatal conductivity, chlorophyll content and activity of photosystem II) of *A.caudatus* plants grown in the first and second independent experiments…………………………………………………………………………….…......12

Figure S1(4). Leaf related water content of *A. caudatus* young and mature leaves of control and Zn-treated plants of the 1st and 2nd plant experiments. ………..……………………………………………………..13

Figure S1(5). The growth in fresh and dry weight of *A. caudatus* root, young and mature leaves in response to Zn^{2+}-exposure for 1 week……………….………………………………………………….....14

Figure S1(6). EI-MS spectra of di- and trisaccharides from in-house library……………………..……..15

Figures S1(7). GC-MS information supporting annotation of RI2600 Disaccharide and its preliminary annotation as aldohexosylfructose…………………………………………………………………..…...16

Figures S1(8). GC-MS information supporting annotation of RI2504 Disaccharide and its preliminary annotation as aldohexosylfructose……………………………….………………………..…...18

Figure S1(9). Metabolic response of *A. caudatus* mature leaves to Zn^{2+}-treatment…………..……………..20

Figure S1(10). Pathway Analysis of the Zn-regulated metabolites of *A. caudatus* mature leaves annotated by untargeted GC-EI-Q-MS…………………………………………...…………………….……21

Figure S1(11). Gluconic acid (6TMS) experimental t_R, RI and EI-MS spectra and its EI-MS and RI similarity with that of NIST and GMD libraries performed by NIST Search program ……………………………….22

Figure S1(12).Calibration curve of individual standards ……………………………………………..........24

Tables

Table S1(1). Metabolites analized by untargeted gas chromatography-electron ionization-quadrupole mass spectrometry (GC-EI-Q-MS) approach in dry methanolic extracts obtained from young and mature leaves and roots of *A. caudatus* Zn-treated and control plants …..………………………………………….……....25

Table S1(2). Zn-regulated metabolites in young leaves of *A. caudatus* ..……………………………....58

Table S1(3). Zn-regulated metabolites in roots of *A. caudatus* …..…………………………….......…61

Table S1(4). Zn-regulated metabolites in mature leaves of *Amaranthus caudatus* ..…...…………….………65

Table S1(5). Structures of common fragments in EI spectra of carbohydrates...…………………………..67

Table S1(6). Zn-responsive metabolic pathways in *A. caudatus* young leaves…….…………………………68

Table S1(7). Zn-responsive metabolic pathways in *A. caudatus* roots…………….……………….………70

Table S1(8). Zn-responsive metabolic pathways in *A. caudatus* mature leaves……………………………72

Table S1(9). Changes in content of metabolites detected by GCMS targeted analysis in mature leaves of *A. caudatus* in response to Zn^{2+}-treatment………………………………………………………… …74

Table S1(10). Gas chromatographic separation conditions and electron ionization-quadrupole-mass spectrometry (EI-Q-MS) settings for analysis of *A. caudatus* primary polar thermally stabile metabolites……………………………………………………………………………………..76

Protocols

Protocol S1-1. Annotation algoritm applied to elucidate structure of di- and oligosaccharides based on the EI mass spectra and RI

The generalized protocol for assessment of the oligosaccharide structures assumes the following steps:

1) Localization of the t_R neighbors in the chromatograms identified by co-elution with authentic standards;
2) Inspection of the EI mass spectra for the presence of the signals characteristic for specific sugar moieties;
3) Inspection of the EI mass spectra for the presence of the signals characteristic for specific types of the glycosydic linkage.

Additionally to the metabolite assigned as RI2864 di- or oligosaccharide (the corresponding annotation algoritm was described in the manuscript text in the Results section), here we provide two other examples to demonstrate annotation algoritm applied to elucidate structure of di- and oligosaccharides from EI-MS and RI.

Example 1

RI2600 disaccharide (Table S1(2)), which showed a 9.1-fold increase in abundance in Zn-treated *A. caudatus* young leaves in comparison to the controls. This metabolite was annotated as a disaccharide based on the following information (Figure S1(7)):

1) it is eluted in the t_R window between elution of two disaccharides - sucrose (t_R 38.95, RI2541, in-house library) and trehalose (t_R 41.59, RI2675.5, in house library).

2) its EI spectrum showed diagnostic fragment signals at *m/z* 451, 361 (which is the neutral loss of a TMSOH moiety from *m/z* 451) and *m/z* 271 (this signal originated from the loss of a TMSOH group from the fragment ion with *m/z* 361) which served as the indicators for the presence of an aldopyranose ring moiety, and at *m/z* 451 and 437 which indicated the presence of a ketohexose (fructose) moiety in the pyranose or furanose forms (Kamerling et al., 1972).

3) the presence of a relatively intense signal at *m/z* 437 also might indicate the presence of 1→1 or 1→2 glycosidic linkages within the molecule. Additionally, the ratio of signals at *m/z* 217 and 204 was 0.7. It is known that pyranose forms the fragments at *m/z* 217 with a lower relative intensity than at *m/z* 204 (Kamerling et al., 1972), and Kamerling et al reported the ratio 217/204 of 0.8 for β-D-glucopyranosyl-(1→1)-D-fructose. Thus, RI2600 disaccharide might be tentatively assigned as aldohexosylfructose.

Example 2

RI2504 disaccharide (Table S1(3)), which showed the most pronounced down-regulation (a 4.7-fold decrease in relative intensity) in roots of Zn-treated plants in comparison to the controls. Figure S1(8) represents the total current chromatogram (TIC) with the RI2504 disaccharide peak together with the corresponding EI mass spectrum, XIC for the characteristic *m/z* 361 and integrated areas for the peak at t_R 38.24, which were

used in statistical analysis (resulted in the 4.7-fold significant difference between Zn-treated and control variants). The metabolite was annotated to the class of disaccharides by the following reasons:

1) the metabolite eluted in close proximity to the t_R of sucrose (t_R 38.95, Table S1(1));

2) the EI mass spectrum of this metabolite contained the fragments at *m/z* 451, 437, 361, 271, which are characteristic for disaccharides, and signals which are generally typical for carbohydrates – *m/z* 319, 217, 191. The signals at *m/z* 451, 361 and 271 indicate the presence of the aldohexosylpyranose moiety as a part of the disaccharide molecule, while relatively intense signal at *m/z* 437 indicates the presence of a fructofuranose unit;

3) ratio of the signals at *m/z* 217/204 appeared to be >>1, since intensity of the peak at *m/z* 217 usually is higher for furanose form, relatively high intensity of the signal at *m/z* 217 suggested the presence of fructofuranose. Therefore, RI2504 disaccharide can be tentatively assigned as **aldohexosylfructos**e with unknown type of glycosidic linkage.

Protocol S1-2. Composition of nutrient solution

The composition of full nutrient solution (in mmol/L) was as follows: $Ca(NO_3)_2 \times 4H_2O$ – 3.81; KNO_3 – 6.44; $MgSO_4 \times 7H_2O$ – 0.81; KH_2PO_4 – 1.83; NH_4NO_3 – 0.87; Fe-EDTA – 0.09; H_3BO_3 – 0.047; $MnSO_4 \times 5H_2O$ – 0.007; $ZnSO_4 \times 7H_2O$ – 0.0007; $CuSO_4 \cdot 5H_2O$ – 0.0008; $(NH_4)_2MoO_4$ – 0.0005. Nutrient solutions in the culturing vessels were continuously aerated and replaced every seven days. The pH values were daily monitored and maintained at 5.8±0.1. If necessary, the pH values were re-adjusted with 0.01 N H_2SO_4 or NaOH.

Protocol S1-3. Plant sample preparation (extraction of low-weight thermally stable metabolites and their derivatization) and their gas chromatography-mass spectrometry analysis.

The in-house established protocol for profiling of low-weight thermally stable polar primary metabolites was in described in Leonova et al., 2020 and included (i) extraction of plant material with aq. methanol, (ii) subsequent derivatization of the extracted primary metabolites and (iii) their analysis by gas chromatography-electron ionization-quadrupole-mass spectrometry (GC-EI-Q-MS). This protocol was performed with a few following modifications.
In detail, approximately 10 and 20 mg of ground dry leaf and root material, respectively, were extracted with 1 mL methanol. After vortexing (3000 g, 30 s) and centrifugation (12000 g, 4 °C, 10 min) of the suspensions, the resulted supernatants were collected. The plant material residues were additionally supplemented with 0.1 mL of deionized water. After a following vortex and centrifugation cycle, the obtained supernatants were combined with the first portions. The total extract volume was 1090 µL. Aliquots (30 µL) of the resulted aq. methanolic extracts were freeze-dried under reduced pressure with Labconco CentriVap centrifugal concentrator (Labconco, Kansas City, USA). The volumes of extracts, used for derivatization and GC-MS analysis were optimized in a series of preliminary experiments in which the injection amounts were optimized to avoid loss of chromatographic resolution and excessive carry-over (was verified by exploring corresponding inter-sample blanks). The residues were sequentially derivatized with methoxyamine hydrochloride in pyridine, and *N*-methyl-*N*-(trimethylsilyl) trifluoroacetamide (MSTFA) according to the established procedure [Leonova et al., 2020]. The samples (1µL) were injected with CTC GC PAL Liquid Injector (Shimadzu Deutschland GmbH, Duisburg, Germany) into GC2010 gas chromatograph coupled online to a quadrupole mass selective detector Shimadzu GCMS QP201 operating under the instrumental settings summarized in Supplementary Information 1, Table S1-10.

Besides the experimental samples, the sequence for the GC-MS analysis also included: (i) quality controls (QCs, i.e. aliquots of the pool prepared by mixing all individual extracts), (ii) C_8–C_{20}-alkanes dissolved in hexane for calculation of metabolite retention indices, (iii) mixes of 29 authentic standards representing individual points of external calibration (additional information is given below). To avoid the misleading readouts and interference with the targeted biological effect, we did not supplement internal standard to the extraction solvent. Instead, assessment of the method performance relied on QCs, i.e. the approach which proved to be applicable in our previous work [Leonova et al., 2020].

Figures

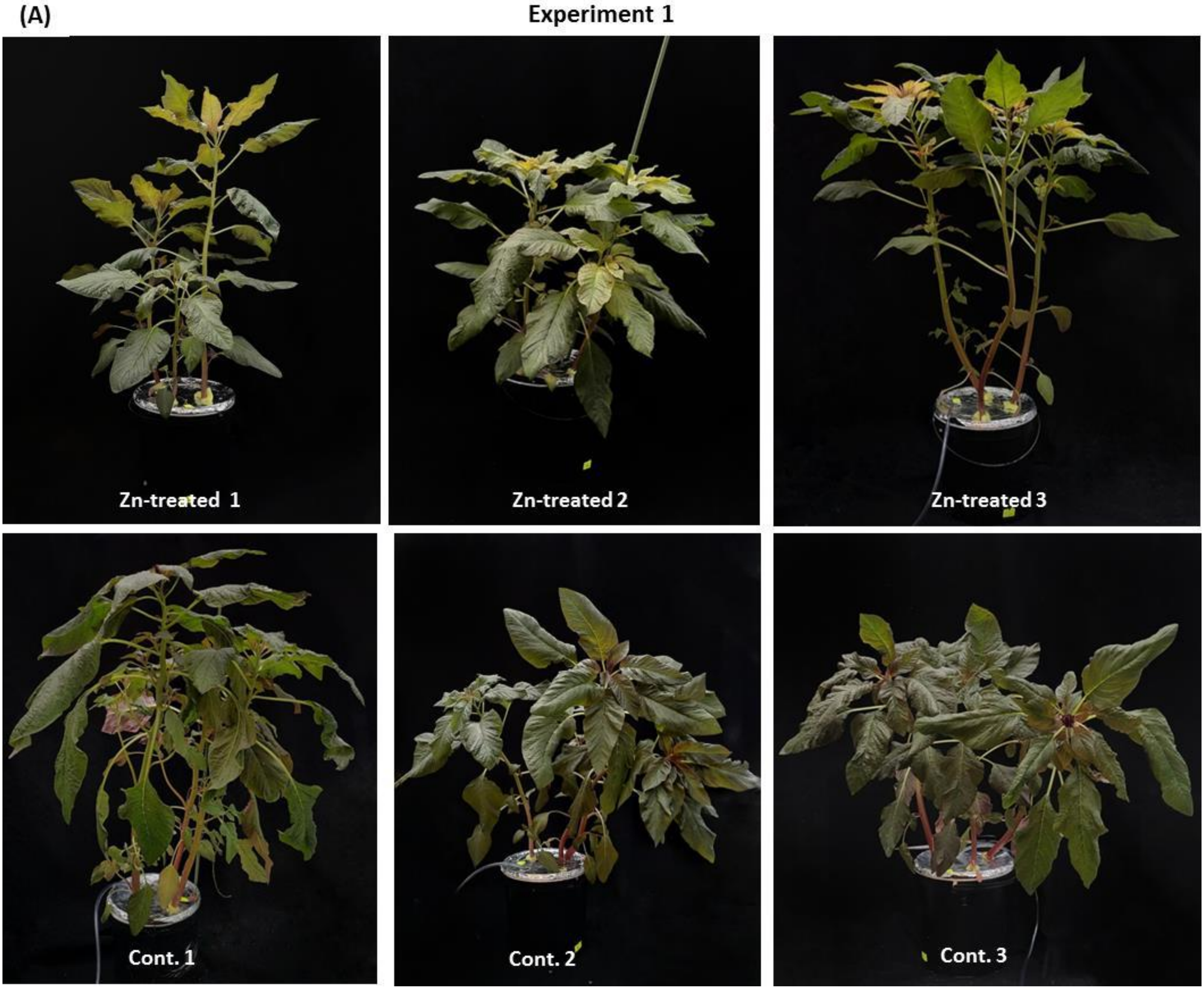

Figure S1(1). Shoots (**A**) of seven week-old *A.caudatus* plants grown in the **first independent experiment** in hydroponic nutrient solution in the presence (Zn^{2+}-treated group, $n = 9$) and absence (Control group, $n = 9$) of 300 μmol/L $ZnSO_4$ for one week. Each photo presents three plants grown in a vessel. There were three vessels in control and Zn^{2+}-treated groups.

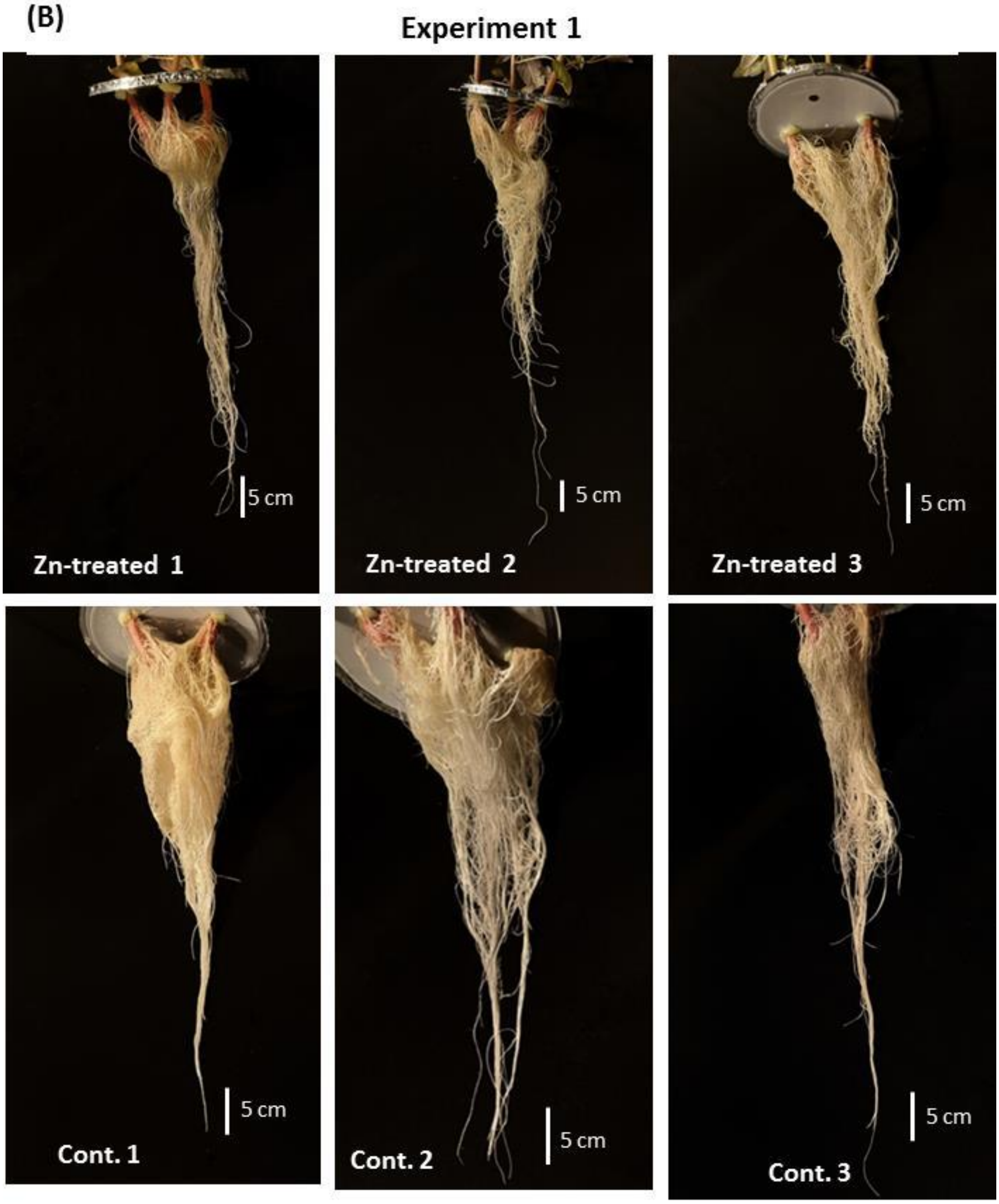

Figure S1(1). Root systems (**B**) of seven week-old *A.caudatus* plants grown in the **first independent experiment** in hydroponic nutrient solution in the presence (Zn^{2+}-treated group, *n* = 9) and absence (Control group, *n* = 9) of 300 µmol/L $ZnSO_4$ for one week. Each photo presents three plants grown in a vessel. There were three vessels in control and Zn^{2+}-treated groups.

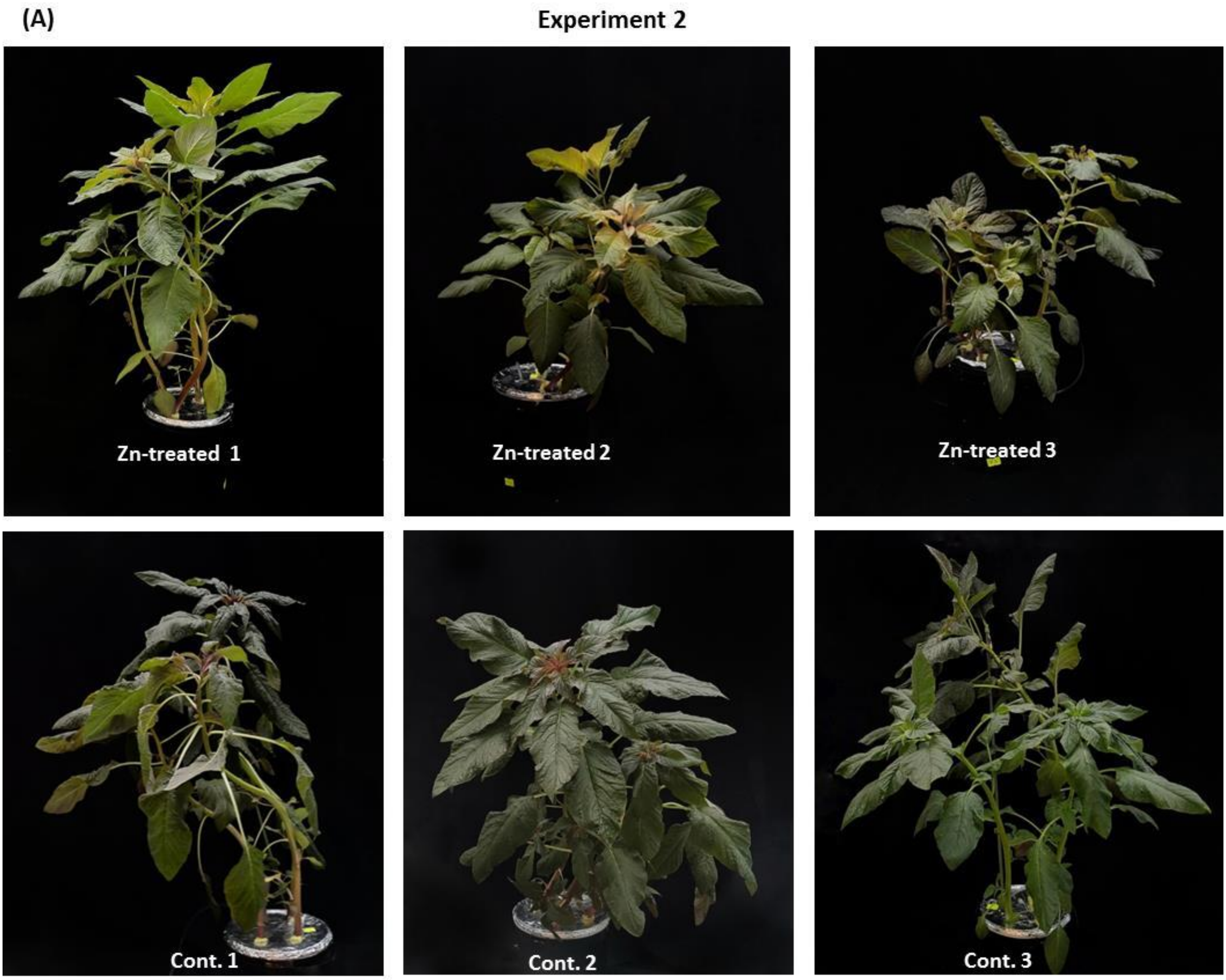

Figure S1(2). Shoots (**A**) of seven week-old *A.caudatus* plants grown in the **second independent experiment** in hydroponic nutrient solution in the presence (Zn^{2+}-treated group, *n* = 9) and absence (Control group, *n* = 9) of 300 μmol/L $ZnSO_4$ for one week. Each photo presents three plants grown in a vessel. There were three vessels in control and Zn^{2+}-treated groups.

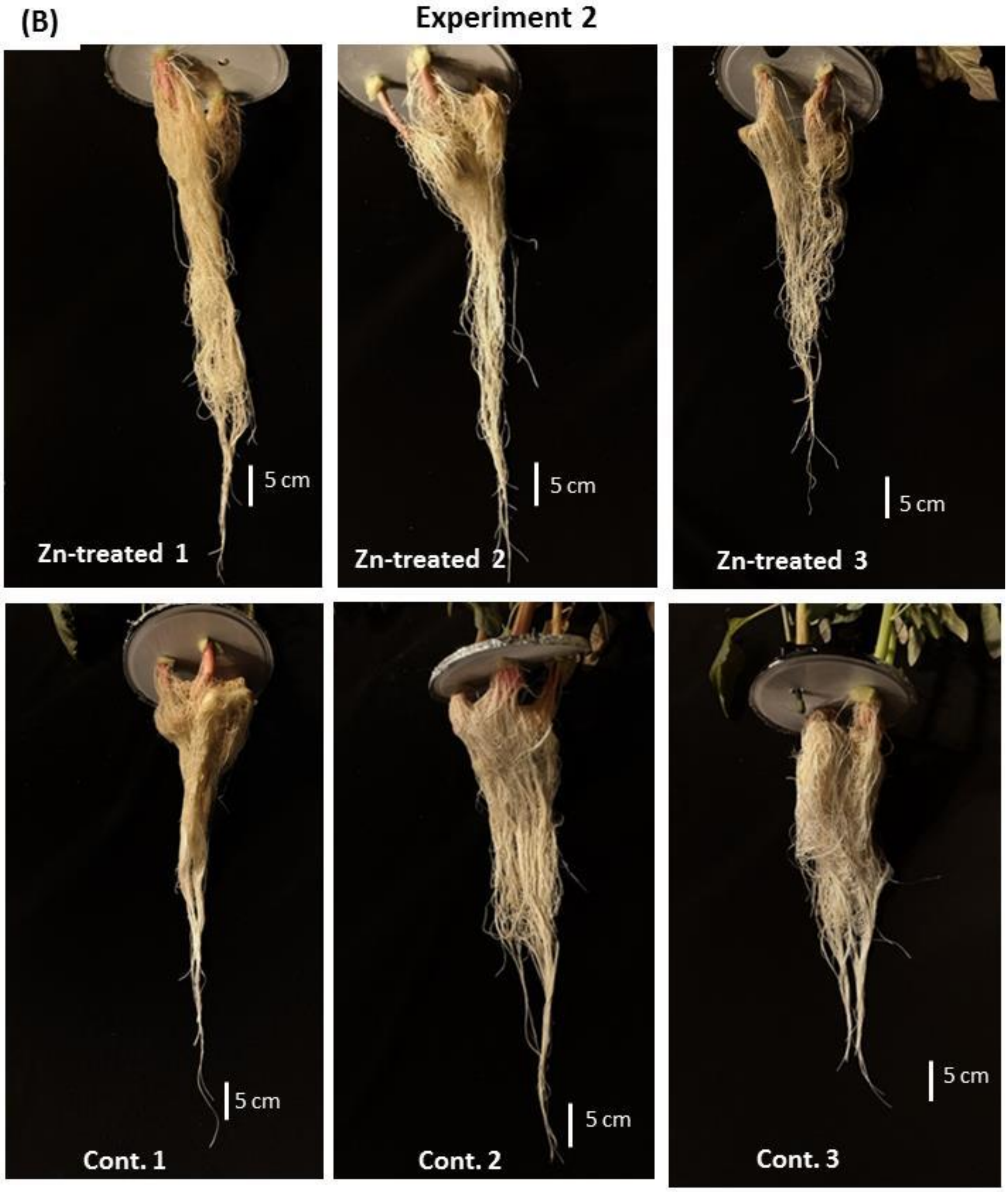

Figure S1(2). Root systems (**B**) of seven week-old *A.caudatus* plants grown in the **second independent experiment** in hydroponic nutrient solution in the presence (Zn^{2+}-treated group, $n = 9$) and absence (Control group, $n = 9$) of 300 µmol/L $ZnSO_4$ for one week. Each photo presents three plants grown in a vessel. There were three vessels in control and Zn^{2+}-treated groups.

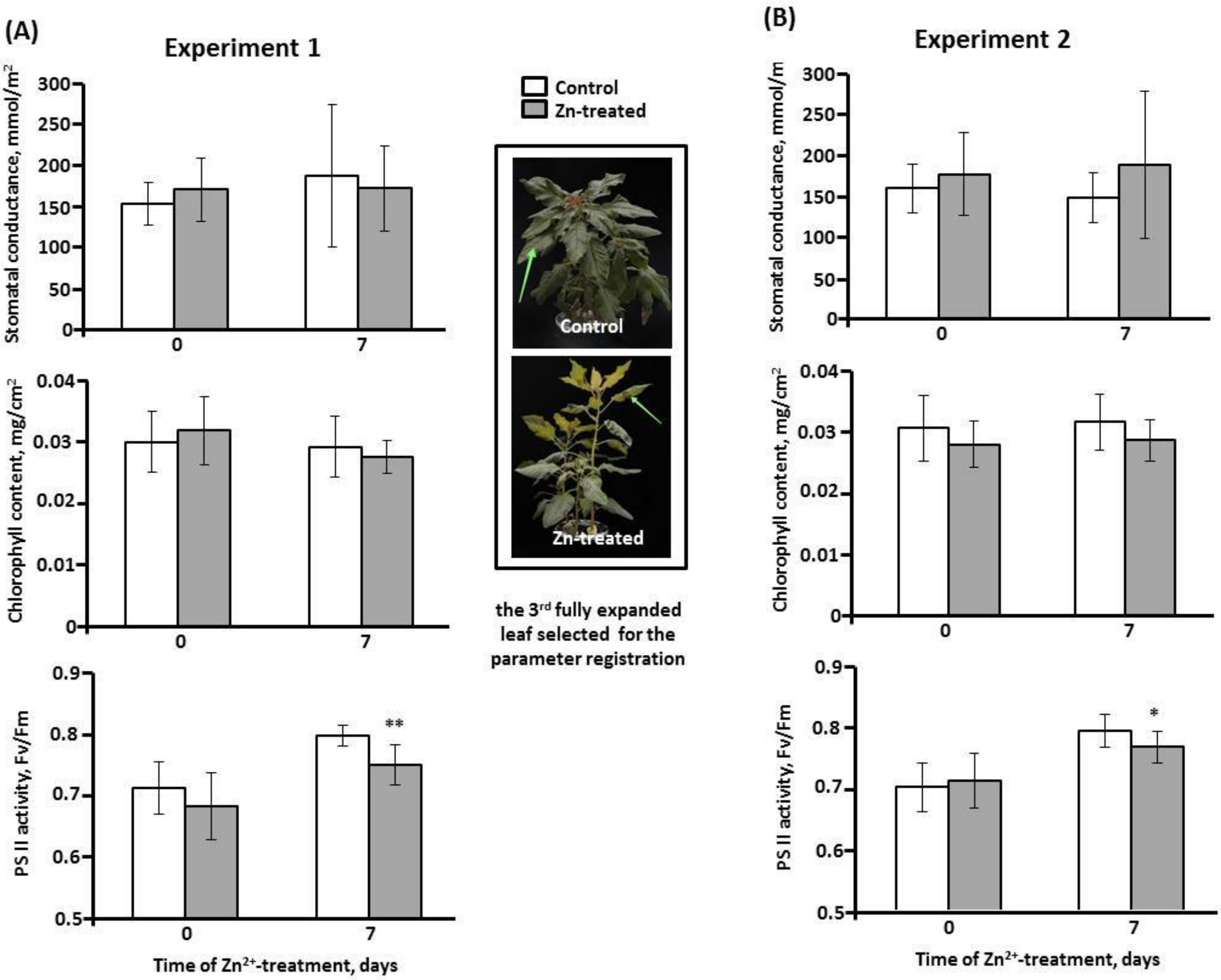

Figure S1(3). Impact of exogenous Zn^{2+} (300 µmol/L) on stomatal conductivity, chlorophyll content and photosystem II (PS II) activity of the third mature leaf (the leaf is indicated by green arrows at photos in the insert; numeration of the mature leaves were done just beneath the plant top with young (i.e. not fully expanded) leaves) from six week-old (before Zn^{2+}-exposure, 0 day) and seven week-old *A.caudatus* plants (n = 9) (after Zn^{2+}-exposure, 7 day) grown in the first (A) and second (B) independent identical experiments in hydroponic nutrient solution. Grey columns indicate the Zn-treated group, namely plants which physiological parameters were measured prior (0 day) and after the Zn-stress exposure (7 day). The Zn-stress exposure of the plants was performed by supplementation of 300 µmol/L $ZnSO_4$ in hydroponic nutrient solution for one week. White columns indicate the control group of plants which were grown during the whole experimental period (from 0 to 7 days) in hydroponic nutrient solution without the addition of 300 µmol/L $ZnSO_4$. Fv/Fm – variable fluorescence (Fv)/maximal fluorescence (Fm). Asteriks * and ** indicate significant difference between Zn-treated and control groups per time point at p(t-test)<0.05 and <0.01, respectively. The experimental data obtained for a leaf of each plant are presented in Supplementary Information 2, Tables S2(1–3).

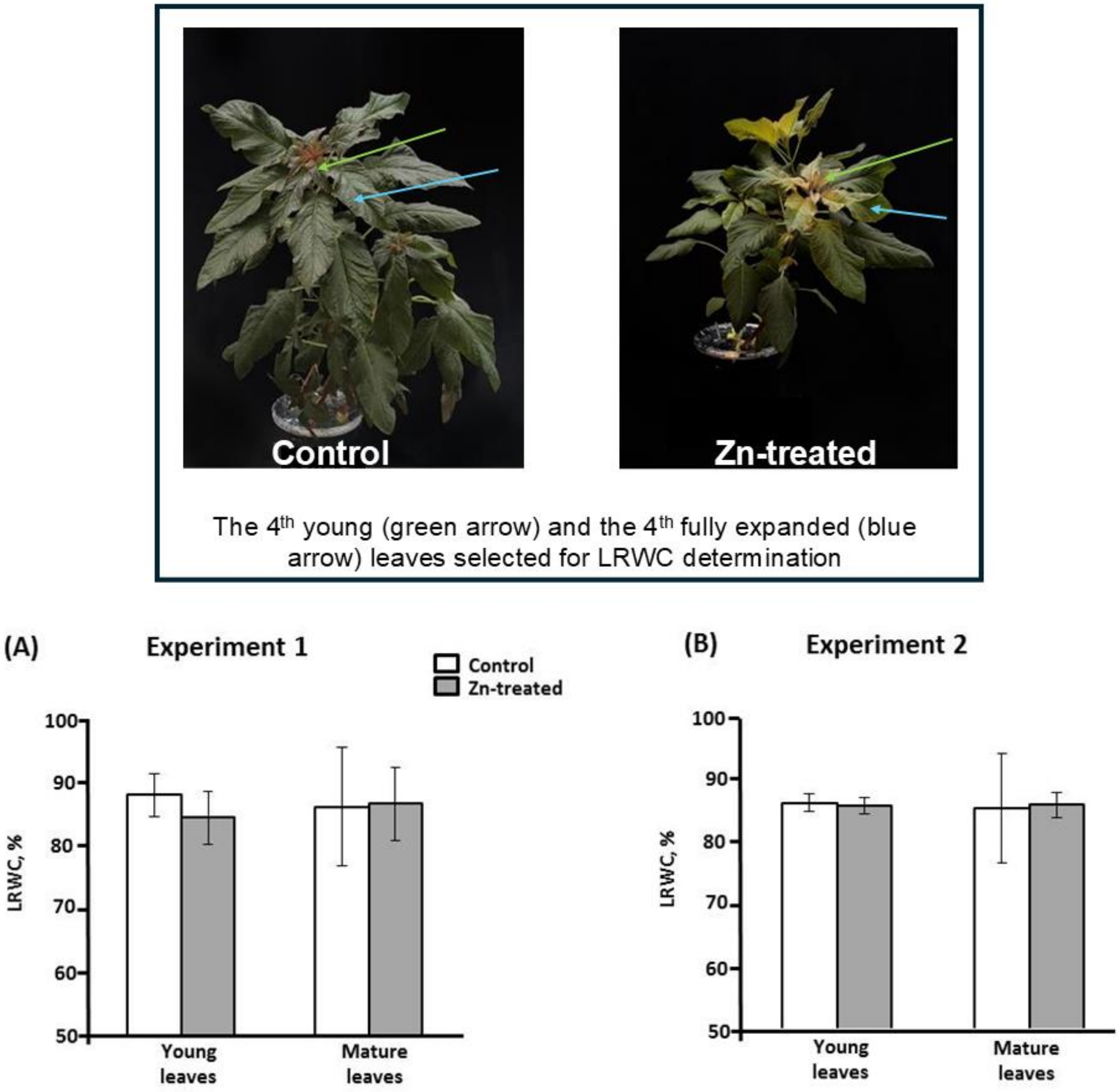

Figure S1(4). Leaf related water content (LRWC) of *A. caudatus* young and mature leaves of control and Zn-treated plants of the 1st and 2nd plant experiments. In each experiment the 4th young and the 4th mature leaves of every plant (as indicated by green arrows at photos in the insert; numeration of the mature leaves were done just beneath the plant top with young (i.e. not fully expanded) leaves) were collected from seven week-old plants (*n* = 9) grown in hydroponic nutrient solution in presence (Zn^{2+}-treated group) and absence (control group) of 300 μmol/L $ZnSO_4$ for one week. The experimental data obtained for each plant are presented in Supplementary Information 2, Table S2(4).

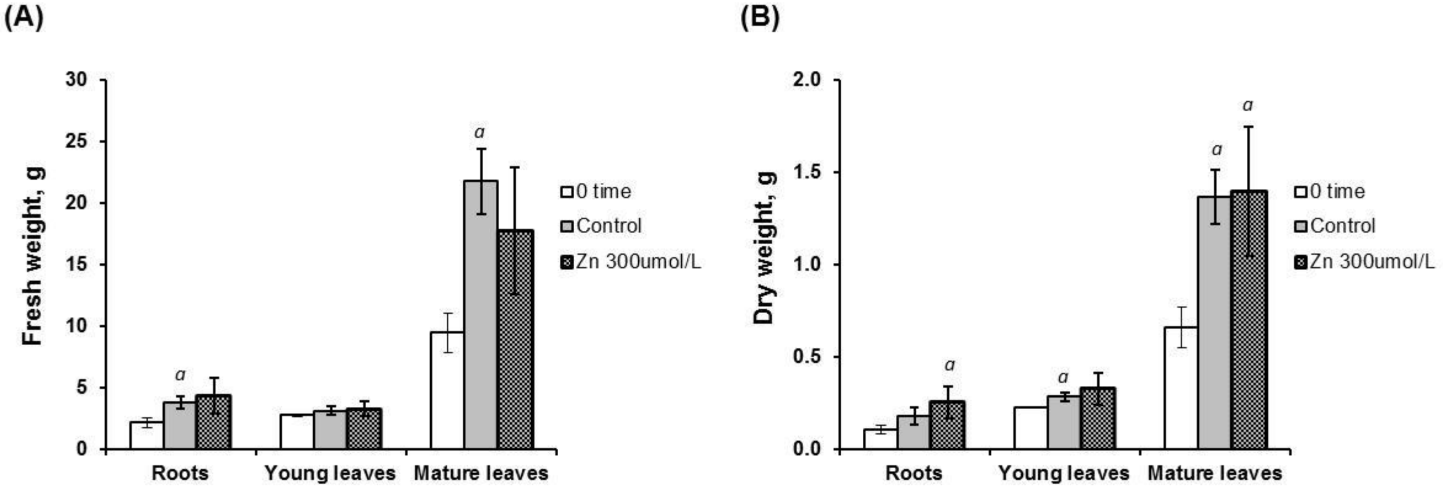

Figure S1(5). Fresh (A) and dry (B) weights of *A. caudatus* root, young and mature leaves assessed before and after the treatment with Zn^{2+} for 1 week in parallel to the corresponding untreated controls. The organs were collected from six-week old (0 time) and seven week-old plants (n = 3, where each of the three biological replicates presented a pool of the organs from three individual plants, Supplementary Information 2, Tables S2(5, 6) grown for one week in hydroponic nutrient solution in presence (Zn^{2+}-group) and absence (control group) of 300 µmol/L $ZnSO_4$. [a]denotes significant (t-test, p<0.05) difference as compared with six-week-old plants (0 time).

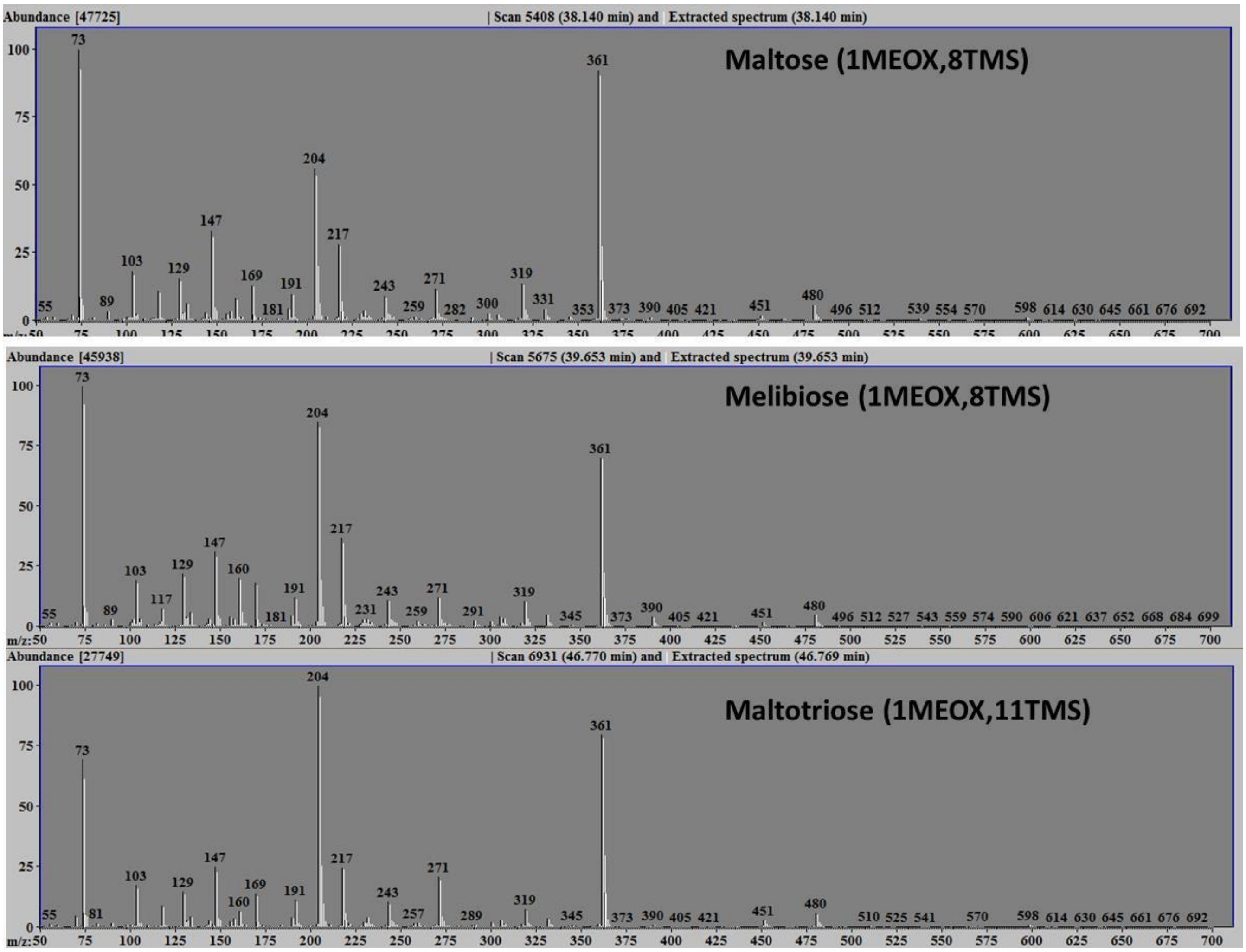

Figure S1(6). EI-MS spectra of di- and trisaccharides from in-house library.

The electron ionization (EI) mass spectra of di- and trisaccharides were recorded during the GC-MS analysis (performed with Shimadzu GCMS QP2010 system equipped with a quadrupole mass selective detector operated under the instrumental settings summarized in Table S1(10)) of three standard solutions: maltose (50 µmol/L), melibiose (50 µmol/L) and maltotriose (50 µmol/L). The di- and trisaccharide standard solutions were dried and subjected to two-step derivatization as described in Method section. One µL of derivatized sugar solution (50 µmol/L) was injected into the GC-MS instrument. The resulted chromatogrammes were processed and the di- and trisaccharides EI-MS spectra were asquired with AMDIS software (www.amdis.net/).

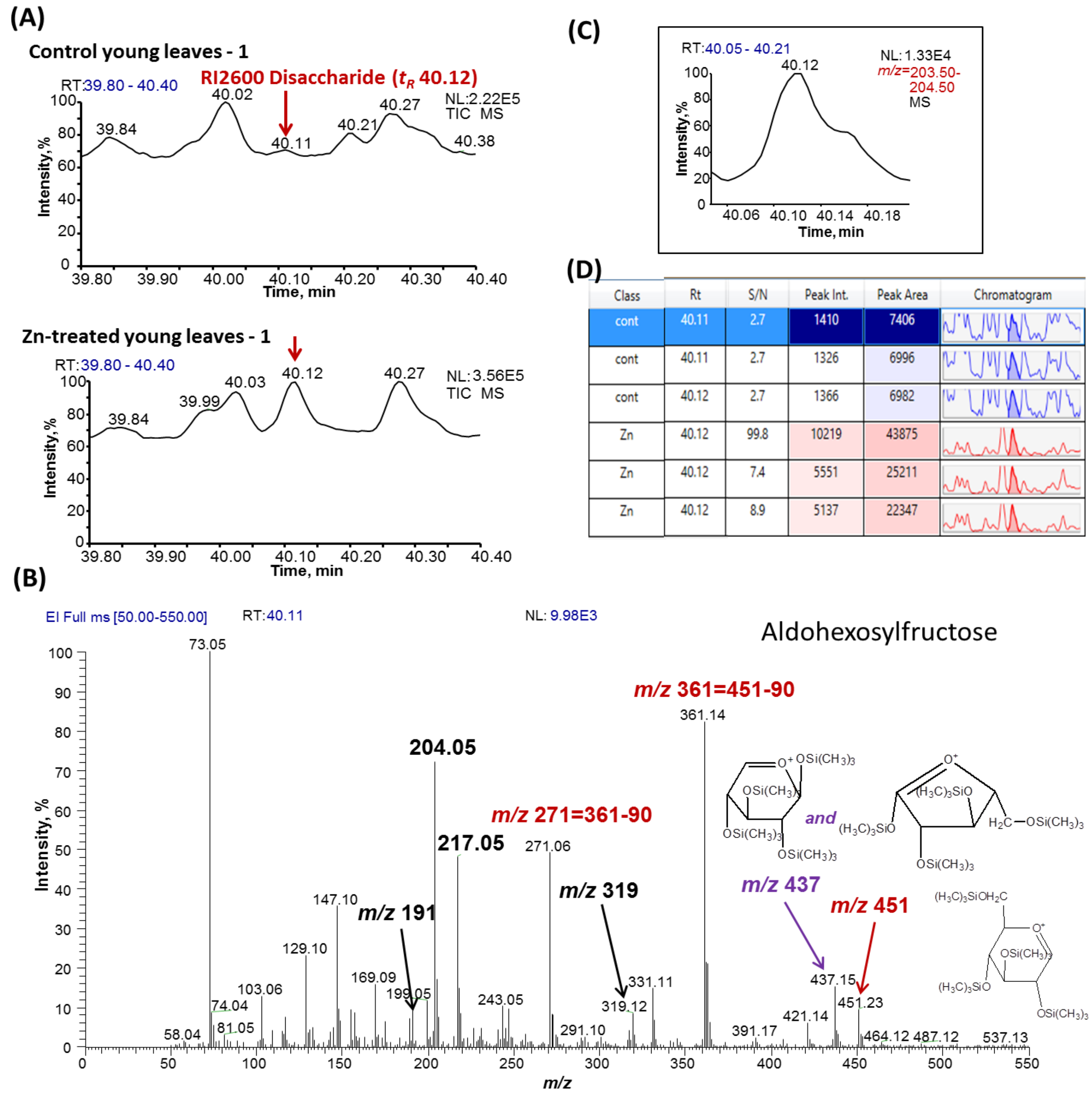

Figure S1(7). GC-MS information supporting annotation of the metabolite, which demonstrated a 9-fold abundance increase in Zn-treated *A. caudatus* young leaves in comparison to the controls and eluted at 40.12 min (retention index RI 2600). Based on this, the metabolite was annotated as disaccharide and tentatively identified as aldohexosylfructose. A – The total ion current chromatogram (TIC) of control and Zn-treated *A. caudatus* young leaf extracts presented for t_R window 39.8–40.4 min in which a peak of the RI2600 disaccharide was eluted; B – EI mass spectra of the metabolite; fragment signals marked with red arrows refer to the moieties of ring structure of aldohexose (at *m/z* 451, 361and 271) and marked with violet arrows refer to the moieties of aldo- or ketohexose (mainly fructose, at *m/z* 437), respectively, according to Kamerling et al., 1971 and 1972. The characteristic loss of 90 u corresponds to the cleavage of $(CH_3)_3SiOH$). The *m/z* values and arrows marked with black bold font presented fragments diagnostic for carbohydrates and their structures

showed in Table S1(5). C – extracted ion chromatogram (XIC) for *m/z* 204±0.5 and t_R 40.12 of the RI2600 disaccharide; D – a screenshot from the MSDial program window represented the RI2600 disaccharide XIC peak area integration values for three control and three Zn-treated samples.

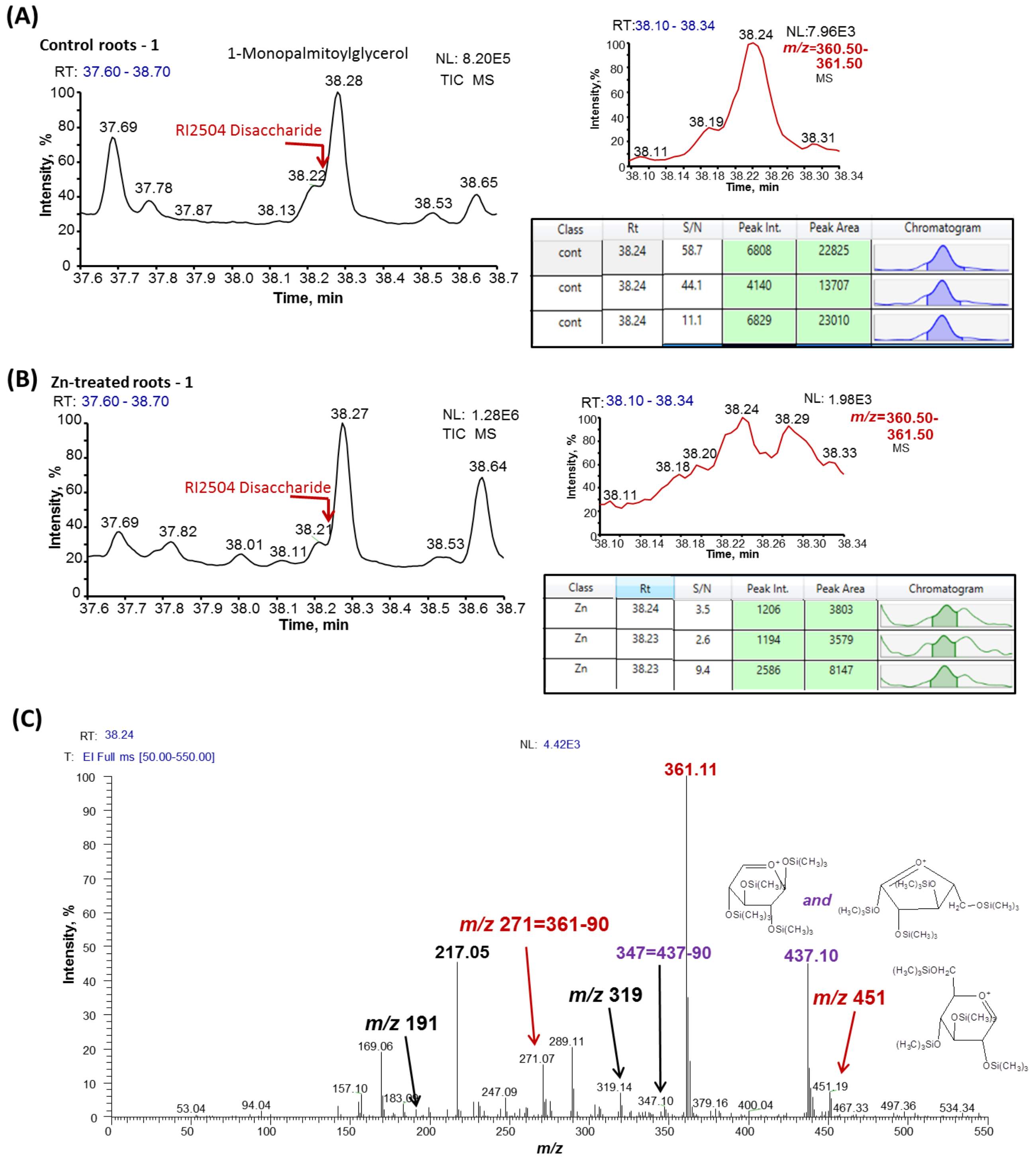

Figure S1(8). GC-MS information supporting annotation of the metabolite, which demonstrated a 4-fold abundance decrease in Zn-treated *A. caudatus* roots as compared with control and eluted at t_R 38.24 (RI2504). Based on this, the metabolite was annotated as disaccharide and tentatively identified as aldohexosylfructose. A and B – The total ion chromatogram of control and Zn-treated *A. caudatus* root extracts, respectively, recorded for t_R window 37.6–38.7 min in which a peak of the RI2504 disaccharide located; the inserts present the extracted ion chromatograms (XICs) for *m/z* 361±0.5 and t_R 38.24 of the RI2504 disaccharide and integration (with MSDial software) of the XIC peak areas in three control and three Zn-treated samples; C – EI mass spectra of the metabolite; fragments marked with red arrows refer to the moieties of ring chain structure of aldohexose

(at *m/z* 451, 361 and 271) and marked with violet arrows refer to the moieties of ring chain structure of fructose (at *m/z* 437) according to Kamerling et al., 1971 and 1972 works. The characteristic loss of 90 u corresponds to the cleavage of $(CH_3)_3SiOH$ moiety). The *m/z* values and arrows marked with black bold font presented fragments diagnostic for carbohydrates and their structures showed in Table S1(5).

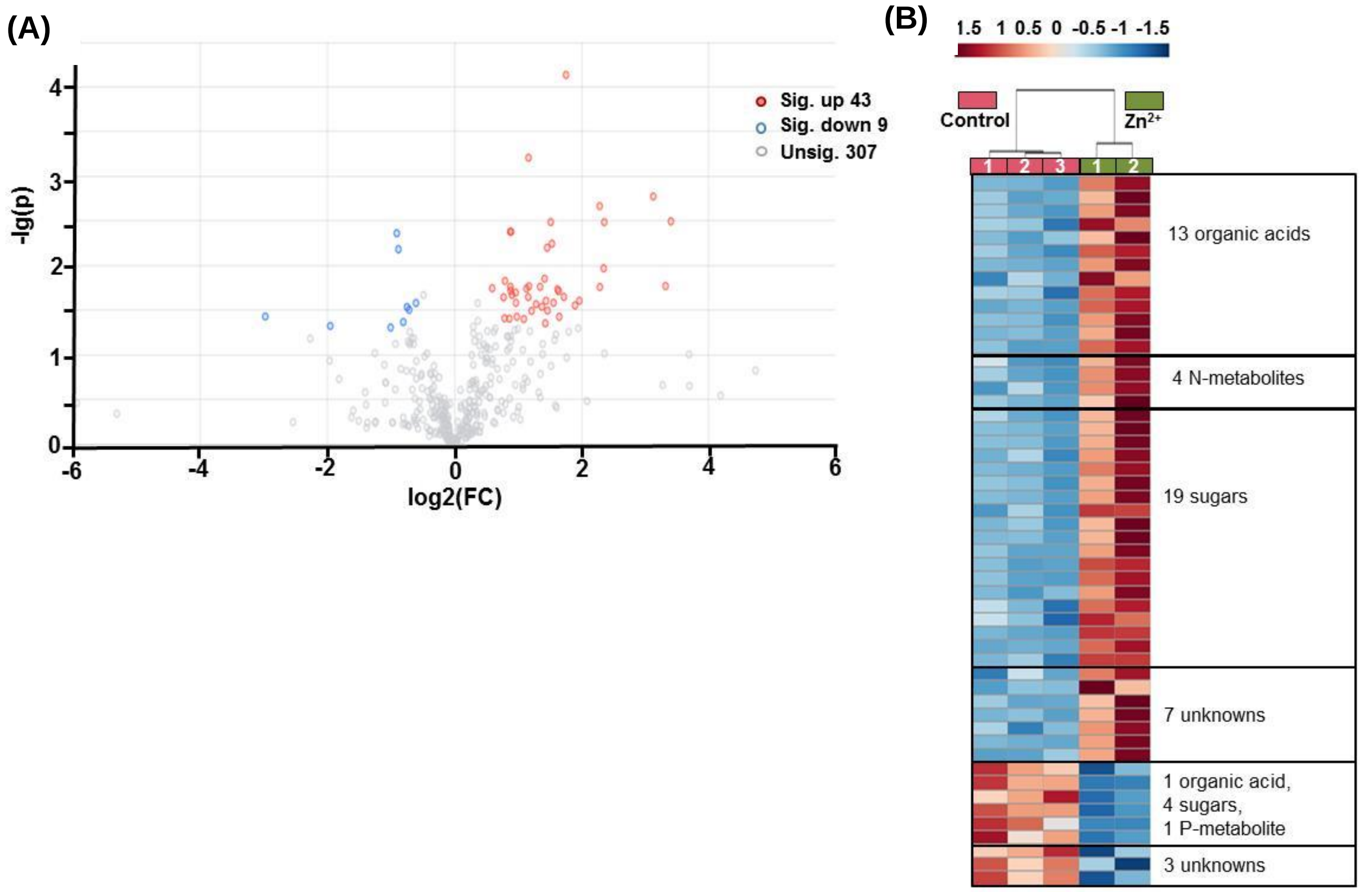

Figure S1(9). Metabolic response of *Amaranthus caudatus* **mature leaves** to a seven day-long treatment with 300 µmol/L $ZnSO_4$ (*n* = 3) in comparison to untreated controls (*n* = 3). The patterns of primary metabolites were characterized in leaf aq. methanolic extracts by untargeted GC-EI-Q-MS approach after appropriate sequential derivatization with methoxyamine hydrochloride in pyridine, and *N*-methyl-*N*-(trimethylsilyl) trifluoroacetamide (MSTFA). The Zn^{2+} solution was supplemented to the nutrient medium. A – volcano plot representing the numbers of the features differentially (≥1.5-fold, $p \leq 0.05$) abundant in *A. caudatus* mature leaves in comparison to the mature leaves of control plants. Color filled circles indicate the features, which were significantly higher- (Sig. up, red) and lower- (Sig. down, blue) abundant in Zn-treated mature leaves in comparison to the controls, grey dots correspond to the features which did not show any significant regulation in mature leaves in response to Zn^{2+}-treatment. B – hierarchical clustering with heat map representation of the the metabolites, significantly ($p \leq 0.05$) increasing and decreasing their abundance (43 and 9, respectively, grouped by classes) upon treatment with Zn^{2+}. The list of the Zn^{2+}-regulated metabolites is presented at Supplementary Information 1, Table S1(4). The volcano plot and the heatmap were constructed in MetaboAnalyst 5.0 (https://www.metaboanalyst.ca).

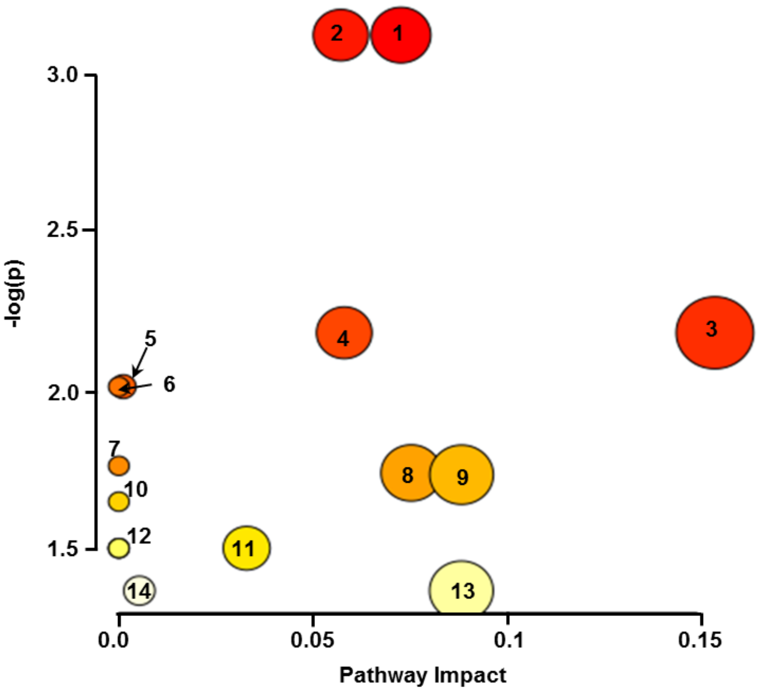

#	Pathway Name
1	Citrate cycle (TCA cycle)
2	Glyoxylate and dicarboxylate metabolism
3	Pyruvate metabolism
4	Carbon fixation
5	Glycolysis / Gluconeogenesis
6	Fructose and mannose metabolism
7	Purine metabolism
8	Galactose metabolism
9	Starch and sucrose metabolism
10	Amino sugar and nucleotide sugar metabolism
11	Sulfur metabolism
12	Ala, Asp and Glu metabolism
	Propanoate metabolism
	Butanoate metabolism
13	Glycerophospholipid metabolism
14	Glycerolipid metabolism

Figure S1(10). Pathway analysis of the Zn^{2+}-regulated (≥1.5-fold, t-test $p \leq 0.05$) metabolites of *A. caudatus* mature leaves annotated by untargeted GC-EI-Q-MS. Metabolite annotation relied on co-elution with authentic standards, as well as by a specral similarity search against NIST and GMD spectral libraries (Supplementary information 1, Table S1(1)). The pathway analysis relied on *Arabidopsis thaliana* pathway library (deposited in the KEGG on-line platform since 03.2020) and presents the results from combined pathway enrichment analysis (global test) and pathway topology analysis (relative-betweenness centrality) to highlight the most confident pathways related to the Zn^{2+}-induced stress response. The both indicators - the higher cycle position along *Y*-axis and more intense red color of the circles, indicate higher significance of the observed differences, whereas the other two indicators - the circle size and position along the *X*-axis indicate the impact of the annotated metabolites in the corresponding pathways. The pathways (marked numerically) listed in the corresponding tables on the right panels are ranked according to the results of pathway enrichment analysis. The symbol * marks the pathways with their pathway impact value ≥0.1 which are considered as main contributors to metabolic response to Zn-exposure. More details for the Pathway Analysis are provided in Supplementary information 1, Table S1(8). To address the regulated metabolic pathways separately for the Zn^{2+}-dependently up- ad down-regulated (≥1.5-fold, t-test $p \leq 0.05$) metabolites (i.e. those increasing and decreasing their abundance upon Zn^{2+} treatment), please refer to the Supplementary information 3, Part 3. The pathway analysis was done in MetaboAnalyst 4.0 (https://www.metaboanalyst.ca) and details on the analysis are provided in Supplementary Information 4.

(A)

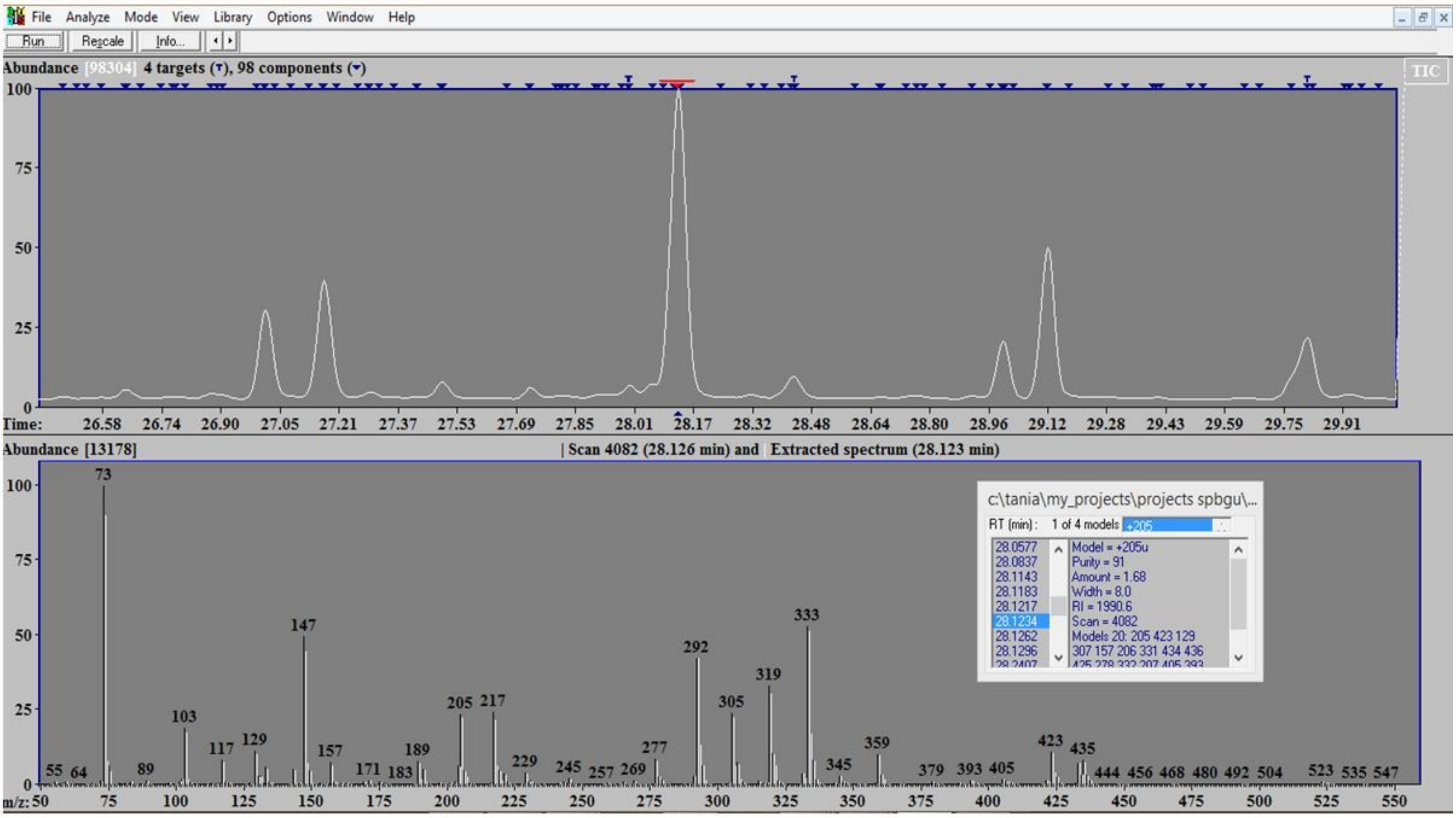
File Analyze Mode View Library Options Window Help
Run Rescale Info...
Abundance [98304] 4 targets (▼), 98 components (▼)
TIC
Time: 26.58 26.74 26.90 27.05 27.21 27.37 27.53 27.69 27.85 28.01 28.17 28.32 28.48 28.64 28.80 28.96 29.12 29.28 29.43 29.59 29.75 29.91
Abundance [13178]
| Scan 4082 (28.126 min) and Extracted spectrum (28.123 min)
c:\tania\my_projects\projects spbgu\...
RT (min): 1 of 4 models +205
Model = +205u
Purity = 91
Amount = 1.68
Width = 8.0
RI = 1990.6
Scan = 4082
Models 20: 205 423 129
307 157 206 331 434 436
m/z: 50 75 100 125 150 175 200 225 250 275 300 325 350 375 400 425 450 475 500 525 550

(B)

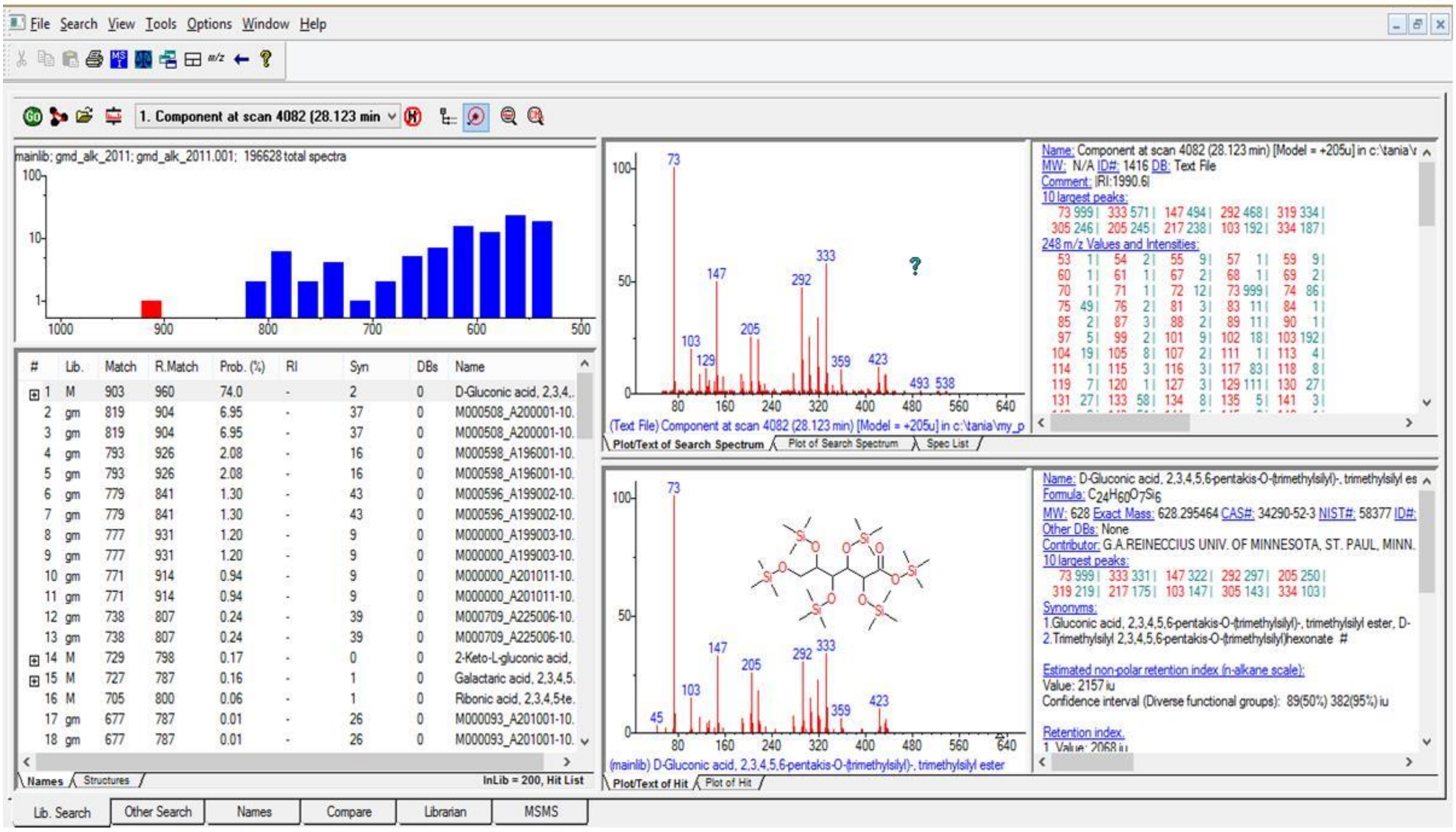
File Search View Tools Options Window Help
1. Component at scan 4082 (28.123 min
mainlib; gmd_alk_2011; gmd_alk_2011.001; 196628 total spectra
Lib. Match R.Match Prob. (%) RI Syn DBs Name
1 M 903 960 74.0 - 2 0 D-Gluconic acid, 2,3,4,..
14 M 729 798 0.17 - 0 0 2-Keto-L-gluconic acid,
15 M 727 787 0.16 - 1 0 Galactaric acid, 2,3,4,5.
16 M 705 800 0.06 - 1 0 Ribonic acid, 2,3,4,5-te.
Names Structures
InLib = 200, Hit List
(Text File) Component at scan 4082 (28.123 min) [Model = +205u] in c:\tania\my_p
Plot/Text of Search Spectrum Plot of Search Spectrum Spec List
Name: Component at scan 4082 (28.123 min) [Model = +205u] in c:\tania\
MW: N/A ID#: 1416 DB: Text File
Comment: |RI:1990.6|
10 largest peaks:
73 999 | 333 571 | 147 494 | 292 468 | 319 334 |
305 246 | 205 245 | 217 238 | 103 192 | 334 187 |
248 m/z Values and Intensities:
(mainlib) D-Gluconic acid, 2,3,4,5,6-pentakis-O-(trimethylsilyl)-, trimethylsilyl ester
Plot/Text of Hit Plot of Hit
Name: D-Gluconic acid, 2,3,4,5,6-pentakis-O-(trimethylsilyl)-, trimethylsilyl es
Formula: C24H60O7Si6
MW: 628 Exact Mass: 628.295464 CAS#: 34290-52-3 NIST#: 58377 ID#:
Other DBs: None
Contributor: G.A.REINECCIUS UNIV. OF MINNESOTA, ST. PAUL, MINN.
10 largest peaks:
73 999 | 333 331 | 147 322 | 292 297 | 205 250 |
319 219 | 217 175 | 103 147 | 305 143 | 334 103 |
Synonyms:
1.Gluconic acid, 2,3,4,5,6-pentakis-O-(trimethylsilyl)-, trimethylsilyl ester, D-
2.Trimethylsilyl 2,3,4,5,6-pentakis-O-(trimethylsilyl)hexonate #
Estimated non-polar retention index (n-alkane scale):
Value: 2157 iu
Confidence interval (Diverse functional groups): 89(50%) 382(95%) iu
Retention index.
Lib. Search Other Search Names Compare Librarian MSMS

(C)

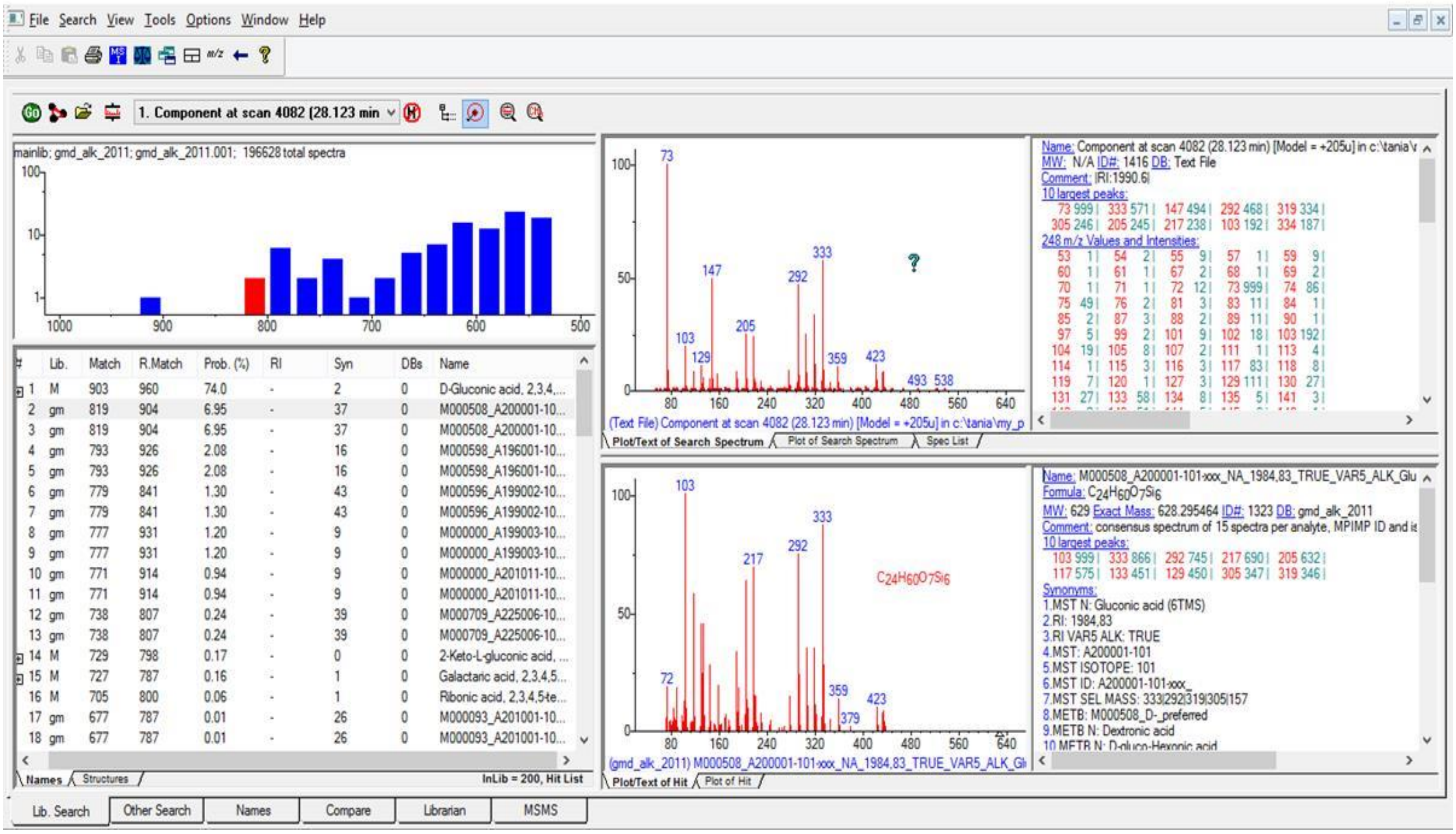

Figure S1(11). Comparison of the experimental t_R, RI and EI-MS spectra of gluconic acid-6TMS (**A**) with its EI-MS and RI similarity data obtained from NIST (M, mainlib of NIST, **B**) and GMD (gm, **C**) libraries performed by the NIST Search program. “?” denotes experimental EI-MS.

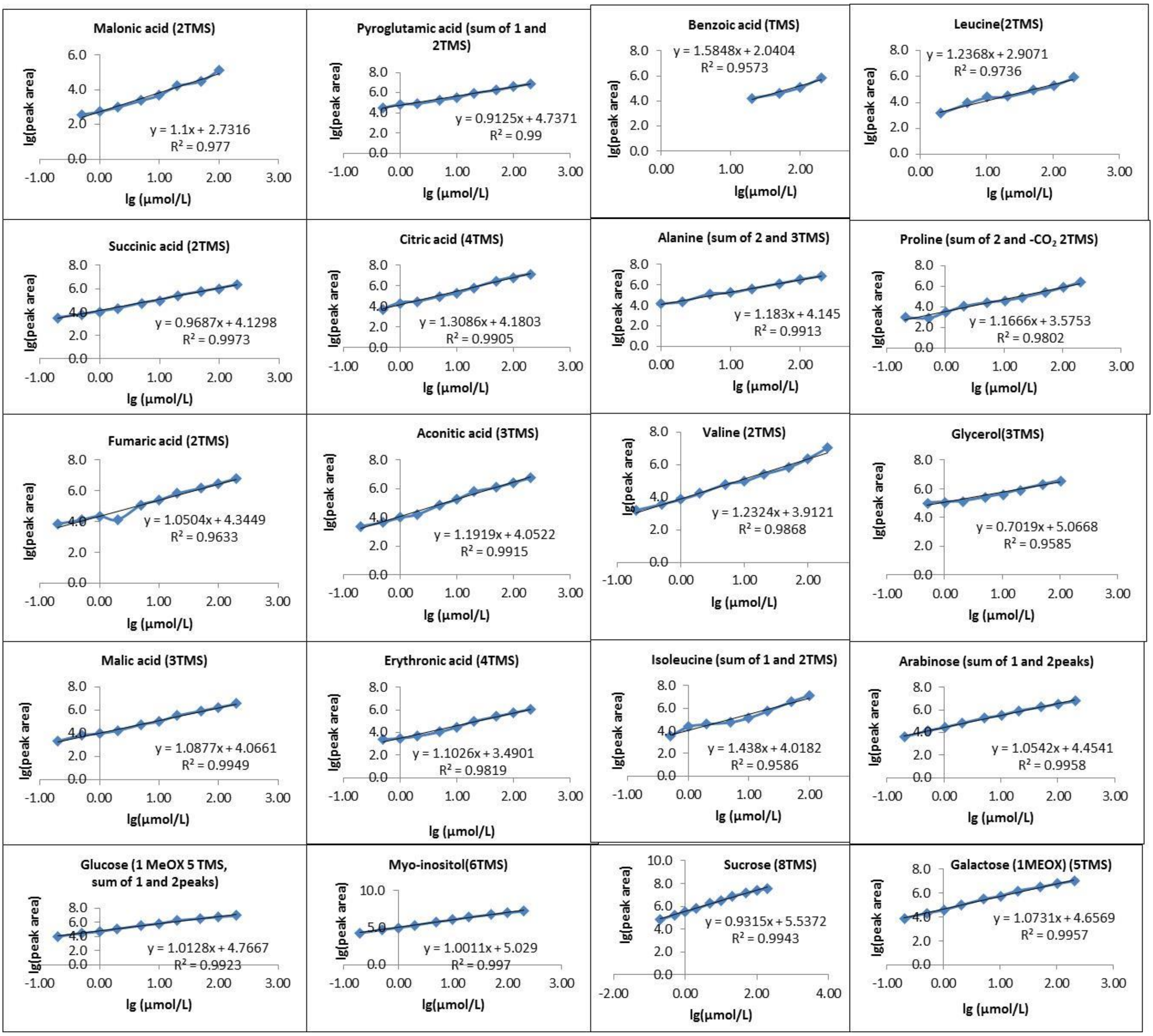

Figure S1(12). Calibration curves of several individual standards.

The undividual standards were used for targeted analysis of *A. caudatus* root and leave metabolites. The standards were prepared as a total mix serially diluted in the range from 0.2 μmol/L to 0.2 mmol/L, then the standard solutions were freeze-dried, sequentially derivatized with methoxyamine hydrochloride in pyridine, and *N*-methyl-*N*-(trimethylsilyl) trifluoroacetamide (MSTFA) as it was done for other experimental samples (Methods section) and were included in the same sequence batch with experimental samples for further GMS analysis. Calibration relied on external standardization, accomplished with the mixture of authentic standards as follows: the analyte peak areas extracted from XIC chromatogrammes were log-tranformed and the resulting individual calibration curves were plotted as the function of logarithmic scale for the applied concentration (0.2, 0.5, 1, 2, 5, 10, 20, 50, 100 and 200 μmol/L).

Tables

Table S1(1). Metabolites analyzed by untargeted gas chromatography-electron ionization-quadrupole mass spectrometry (GC-EI-Q-MS)-based approach in dry methanolic extracts obtained from young and mature leaves and roots of *A. caudatus* Zn^{2+}-treated and control plants.

#	Metabolites/features[a]	Deriva-tives[b]	t_R, min[c]	RI_a (RI_{ST})[d]	ΔRI [e]	*m/z* [f]	EI-MS[g]	Library[h], match factor
1	RI995 Unknown	-	5.21	995.4		258	151(99.9), 147(48.2), 73 (25.4), 66 (22.4), 258(18.4),131(17.8), 204(17.7), 110(16.3), 52(14.9), 77(12.8), 94(9.5), 135(7.0), 138(7.1), 137(5.9), 162(5.9), 88 (5.1), 166(5.0)	-
2	Carbodiimide*	2TMS	5.23	996.0	42.9	186	171 (99.9), 172 (17.8), 78 (11.3), 173 (8.1), 64 (6.1), 186 (7.3)	NIST, 848
3	RI1001 Unknown	-	5.30	1000.6		126	126 (99.9), 127(52.6), 221(43.4), 69(31.3), 140(25.0), 139(22.5), 134(20.6), 79(19.7), 155(15.2), 263(15.1), 264(7.7), 223(7.0)	-
4	RI1003 Unknown	-	5.35	1002.7		117	117(99.9), 73(94.8), 133(69.7), 147(60.7), 126(48.0), 75(24.9), 127(22.2), 69(20.0), 140(17.0), 58(14.7), 139(11.6), 148(10.2), 59(9.3), 178 (8.6),74(8.0), 207(7.7), 149(7.2)	-
5	Boric acid*	3TMS	5.44	1007.2	2.8	221	221 (99.9), 73 (82.8), 263 (26.9), 222 (23.9), 223 (12.5), 264 (7.3), 205 (7.1), 74 (7.0), 262 (6.7), 175 (6.2)	NIST, 920
6	Glycine_1	1TMS	5.63	1016.1	97.3	132	132 (99.9), 75 (92.4), 88 (36.3), 73 (29.3), 133 (9.7), 59 (8.6), 76 (6.7), 74 (5.2), 61(6.2)	NIST, 705
7	Glycine_2	1TMS	5.66	1017.8	99	132	132 (99.9), 73(61.7), 75(57.7),133(9.5), 59(7.5), 74(6.3), 61(6.2), 60(5.4)	NIST, 691
8	RI1023 Unknown	-	5.76	1022.7		184	77(99.9), 184(71.0), 134(70.6), 207(55.3), 214(41.1), 281(23.6), 140(18.1), 229(14.4), 103(13.8), 107(13.7), 143(12,9), 110(10.5), 59(10.2), 96(7.5), 91(6.4), 55(6.4), 60(6.1), 88(6.0), 185(5.6), 215(5.2)	-
9	1,2-Propylene glycol*	2TMS	5.77	1022.5	8.7	117	117 (99.9), 73 (62.0), 147 (55.9), 66 (11.2), 118 (10.40, 148 (8.6), 75 (6.1), 81 (5.8), 74 (5.6)	NIST, 950

10	RI1024 Unknown		5.79	1024.5		158	56(99.9), 73(53.9), 158(47.0), 114(10.3), 81(10.0), 155(9.5), 100(6.8), 128(6.3), 159(6.3), 207(6.3)	-
11	N,N-Dimethylglycine*	1TMS	5.92	1031.0	10	58	58 (99.9), 160 (4.9)	NIST, 879
12	Methyl-lactate*	1TMS	6.03	1035.4	15.4	89	73 (99.9), 89(55.2), 161(23.3), 59(20.4), 74(8.5), 71(8.0), 147(7.1), 57(5.5)	NIST, 655
13	Hydroxycarbamic acid*	2TMS	6.11	1039.1	20.1	206	73 (99.9), 147 (61.1), 103 (55.5), 206 (29.6), 132 (21.2), 75 (12.7), 148 (9.9), 133 (9.4), 131 (9.3), 74 (8.9), 59 (7.2), 60 (7.1), 104 (5.2), 149 (5.2), 207 (5.4), 221 (6.5),	NIST, 684
14	2-Hydroxypyridine*	1TMS	6.44	1055.0	83	152	152 (99.9), 166 (12.6), 153 (10.6), 167 (9.4), 78 (7.5), 122 (6.3)	NIST, 781
15	Lactic acid*	2TMS	6.79	1076	8	117	73 (99.9), 147 (72.5), 59 (32.4), 117 (25.3), 75 (22.6), 133 (14.1), 148 (12.3), 74 (11.4), 191 (10.9), 58 (10.8), 72 (5.0), 118 (6.1), 119 (9.5), 120 (5.9), 131 (6.6), 134 (8.5), 135 (5.9), 149 (5.5), 209 (5.7)	in-house, NIST, 835
16	RI1082 Unknown	-	6.95	1082.2		240	240 (99.9), 75(98.4), 207(83.9), 73(64.7), 96(58.3), 190(56.4), 68(24.6), 148(22.2), 116(18.7), 71(18.4), 88(15.6), 92(14.0), 132(12.4), 55(12.0), 87(12.0), 171(11.5), 194(10.9), 84(10.6), 179(9.8), 82(8.2), 54(8.8), 121(7.2), 140(7.1),158(6.3), 161(5.8), 160(5.6), 138(5.6),	-
17	RI1090 Unknown	-	7.14	1090		190	66(9.0), 73(45.3), 75 (23.3), 147(100), 148(14.7), 149(8.2), 190(19.8), 221(7.8), 265(11.7)	-
18	Glycolic acid*	2TMS	7.35	1098.9	17.9	177	147 (99.9), 73 (75.5), 177 (17.1), 66 (16.6), 148 (15.9), 205 (11.3), 149 (9.7), 133 (9.5), 161 (6.4), 75 (5.9), 74 (5.3)	in-house, NIST, 924
19	Ethanolamine	2TMS	7.45	1103	83	102	102 (99.9), 73 (47.1), 147 (12.4), 103 (10.3), 104 (6.7), 74 (5.9)	NIST, 851
20	**Alanine**	2TMS	7.69	1112.9 (**1113)**	0.47	116	116 (99.9), 73 (59.3), 147 (42.5), 187 (34.8), 103 (19.2), 117 (13.7), 57 (11.4), 71 (9.5), 70 (6.3), 85 (5.6), 188 (5.6)	ST, in-house, NIST, 925
21	1-Octanol*	1TMS	7.71	1113.7	68.9	187	75 (99.9), 103 (86.4), 187 (79.4), 57 (43.5), 71 (38.8), 85 (22.3), 70 (16.9), 69 (16.7), 84 (12.6), 56 (12.3), 91 (5.0), 83 (6.7), 98 (5.2), 104 (8.3), 105 (10.1), 111 (6.1), 112 (12.2), 188 (11.3)	NIST, 763
22	RI1127 Unknown	-	8.03	1126.7		132	57(8.5), 119 (5.4), 132 (100), 133(6.4), 281(7.1)	-
23	RI1131 Unknown	-	8.14	1131.2		140	52 (5.5), 58(17.8), 59 (10.1), 60 (5.9),	-

							69 (9.7), 73 (86.6), 74 (9.4), 75 (31.7), 77(11.0), 78(6.5), 90(59), 102(6.1), 132(12.8), 134(16.9), 140(100), 141(5.6) , 147(34.2), 184(12.5)	
24	RI1134 Unknown	-	8.20	1133.7		140	60 (5.4), 69(5.8), 73(11.7), 75(7.6), 77(6.6), 90(5.2), 132(12.9), 134(13.6), 140 (100), 184(12.9), 258(5.0)	-
25	RI1136 Unknown	-	8.26	1136		156	84(5.2), 86(15.7), 113(7.7), 129(48.0), 147(63.9), 148(6.2), 156(100), 157(13.2), 158(5.0), 220(51.7)	-
26	RI1140 Unknown (? Alanine-derived)	-	8.34	1139.4		116	116(99.9),117(8.8), 118(3.9), 355(1.5)	-
27	RI1141 Unknown	-	8.35	1141		220	73(99.9), 147(53.6), 220(43.1), 103(11.1), 116(10.9), 74(8.9), 148(8.4), 133(6.9), 75(6.7), 59(5.7)	-
28	Glyoxylic acid*	1MEOX, 1TMS	8.42	1142.6	7.6	218	218 (99.9), 147 (65.2), 233 (33.3), 190 (24.3), 219 (16.3)	NIST, 773
29	**Oxalic acid***	2TMS	8.49	1145.5 (1147)	0.1	190	147 (99.9), 73 (92.3),148 (15.8), 149 (9.3), 66 (7.8), 74 (7.5), 72 (6.5), 75 (6.4), 190 (5.6), 133 (5.0)	ST, in-house, NIST, 943
30	Hydroxycarbamic acid	2TMS	8.65	1152.2	133.2	132	132 (99.9), 73 (30.8),133 (24.0), 147(23.8), 89 (12.8), 75 (11.3), 59 (10.5), 134 (8.6), 206 (5.0)	NIST, 754
31	RI1156 Unknown	-	8.76	1156.6		219		-
32	N-Formylpiperidine*	-	8.88	1161.7	10.7	84	113 (99.9), 98 (31.2), 56 (29.8), 84 (27.8), 207 (20.6), 112 (20.3),52 (9.8), 76 (9.6), 85 (8.4), 70 (7.8), 72 (7.1), 127 (7.1), 54 (6.4), 99 (5.6), 114 (5.7)	NIST, 891
33	RI1167 Unknown	-	9.02	1167.1		152		-
34	RI1178 Unknown	-	9.28	1177.7		119		-
35	Phosphoric acid monomethyl ester*	2TMS	9.47	1185.5	15.5	241	241 (99.9), 163 (21.5), 242 (17.3), 133(14.3), 187 (13.1), 211 (10.2), 243 (9.1), 151 (8.5), 256 (8.2), 135 (6.8), 89 (5.6), 195 (5.0)	NIST, 835
36	**Isoleucine**	1TMS	9.54	1189.1 (1189)	10.5	86	86(99.9), 73(56.0), 147(30.3), 75(22.4), 89(18.1), 133(18.1), 70(11.9), 74(10.7), 217(9.2), 146(8.8), 148(6.3), 134(5.5)	ST, in-house, NIST, 575

37	RI1196 Unknown	-	9.72	1195.8		119		-
38	RI1205 Unknown	-	9.96	1205.2		146		-
39	RI1211 Unknown	-	10.11	1211.2		102		-
40	**Malonic acid***	2TMS	10.11	1211.3 (1212)	6.5	233	147 (99.9), 75 (26.8), 102 (24.1), 148 (15.5), 104 (11.1), 131 (11.1), 149 (9.7), 66 (9.1), 233 (6.4), 103 (6.0), 119 (5.3), 132 (5.0),	ST, in-house, NIST, 804
41	RI1212 Unknown	-	10.13	1212		184		-
42	**Valine**	2TMS	10.26	1217.2 (1218)	12.17	144	144 (99.9), 73 (32.4), 218 (18.5), 145 (11.4), 131 (8.6), 57 (8.5), 100 (7.3), 133 (5.7)	ST, in-house, NIST, 887
43	RI1223 Unknown	-	10.42	1223.2		191		-
44	Ethanolamine	3TMS	10.65	1232.1	25.9	174	174 (99.9), 147 (89.1), 73 (79.7), 72 (50.2), 86 (30.7), 175 (18.1), 59 (17.8), 148 (12.9), 100 (11.3), 176 (8.7), 102 (5.6), 130 (6.9), 131 (6.2), 133 (8.6), 146 (7.2), 149 (7.6), 189 (5.7),	in-house, NIST, 830
45	RI1242 Unknown	-	10.90	1242.1		116		-
46	**Urea***	2TMS	11.07	1248.9 (1247)	0.1	189	147 (99.9), 189 (62.7), 171 (20.6), 148 (17.1), 190 (11.4), 146 (10.1), 66 (9.5), 149 (7.9), 87 (7.6), 173 (7.5), 87 (7.6), 79 (5.5), 99 (5.9), 191 (5.3)	ST, in-house, NIST, 878
47	RI1250 Unknown	-	11.11	1250.1		133		-
48	**Benzoic acid***	1TMS	11.19	1253.3 (1254)	0.3	179	179 (99.9), 135 (69.8), 105 (62.9), 73 (35.1), 207 (30.9), 58 (18.3), 140 (18.1), 180 (16.1), 69 (12.9), 136 (12.4), 76 (5.3), 89 (5.6), 96 (5.3), 78 (5.0), 184 (5.3)	ST, in-house, NIST, 817
49	RI1255 Unknown	-	11.23	1254.7		176		-
50	RI1257 Unknown	-	11.28	1257.4		176		-
51	RI1260 Unknown	-	11.36	1259.8		228		-
52	RI1265 Amine	-	11.50	1265.3		174		-
53	RI1269 Octanoic acid*	1TMS	11.60	1269.1		117		NIST, 764

54	**Leucine**	2TMS	11.67	1272 (1272)	0	158	158 (99.9), 73 (28.1), 218 (15.2), 159 (14.0), 71 (12.6), 57 (10.0), 142 (9.0), 86 (6.2)	ST, in-house, NIST, 807
55	Phosphate*	3TMS	11.72	1273.7	0.7	299	299 (99.9), 73 (41.9), 300 (24.5), 314 (17.9), 301 (13.2), 283 (5.7), 133 (5.0)	NIST, 937
56	**Glycerol***	3TMS	11.79	1276.4 (1276)	15.6	205	147 (99.9), 73 (83.9), 205 (53.5), 117 (36.5), 103 (35.8), 218 (18.9), 148 (15.5), 133 (12.8), 206 (10.1),149 (10.0), 74 (6.4), 75 (5.9), 129 (9.4), 131 (5.4), 191 (5.2), 203 (5.7), 204 (7.5)	ST, in-house, NIST, 928
57	RI1282 Unknown	-	11.93	1281.7		140		-
58	Hydroxycarbamic acid*	3TMS	12.13	1289.9		132	132 (99.9),73 (87.7), 75 (37.7), 147 (298.8), 231 (22.7), 206 (22.0), 133 (17.4),74 (11.6), 145 (11.3), 55 (10.6), 117 (10.0), 110 (7.2), 89 (5.7), 119 (6.4), 129 (6.4), 131 (8.9), 134 (9.0), 143 (5.6), 176 (5.2), 221 (5.6), 269 (6.9)	NIST, 672
59	**Isoleucine**	2TMS	12.24	1294 (1292)	4.8	158	158 (99.9), 73 (51.2), 71 (19.6), 218 (16.6), 159 (15.8), 57 (15.3), 142 (11.7), 269 (9.4), 100 (6.2), 160 (6.2), 86 (5.7), 148 (5.4)	ST, in-house, NIST, 793
60	Nicotinic acid*	1TMS	12.33	1296.9	0	180	180 (99.9), 136 (78.3), 106 (44.4), 78 (26.0), 181 (16.6), 137 (14.2), 51 (9.3), 299 (7.9)	in-house, NIST, 870
61	**Proline**	2TMS	12.36	1299 (1299)	2.1	142	73(99.9), 142(78.5), 144(48.3), 231(44.6), 71 (34.7), 269(28.0), 216(22.2), 232 (21.3), 159(19.0)	ST, in-house, NIST,
62	Glycine*	3TMS	12.51	1304.3 (1306)	5.7	174	174 (99.9), 73 (47.9), 248 (21.7), 86 (21.4), 175 21.3), 132 (20.4), 206 (13.9), 133 (13.8), 100 (11.2), 341 (11.2), 59(7.4), 176 (8.7), 276 (7.8), 429 (8.0)	ST, in-house, NIST, 793
63	RI1306 Unknown	-	12.54	1305.7		140		-
64	RI1312 Unknown	-	12.71	1312.1		280		-
65	RI1314 Unknown	-	12.76	1314.3		105		-
66	**Succinic acid***	2TMS	12.82	1316.6 (1317)	0.6	247	147 (99.9), 73 (33.1), 148 (1.6), 247 (11.3), 75 (9.2), 149 (8.1)	ST, in-house, NIST, 934
67	RI1319 Unknown	-	12.90	1319.6		283		-
68	RI1324 Unknown	-	13.00	1323.7		220		-

69	Glyceric acid*	3TMS	13.18	1330.4	5.3	292	147 (99.9), 189 (67.9), 292 (62.8), 103 (34.9), 102 (30.1), 205 (19.9), 133 (19.7), 293 (17.5), 148 (16.6), 117 (15.2), 149 (9.9), 191 (6.9), 294 (8.9), 307 (10.9)	NIST, 940
70	RI1331 unknown	-	13.22	1331.9		184		-
71	Uracil*	2TMS	13.35	1337.1	4.1	241	241 (99.9),99 (73.1), 147 (56.0), 255 (55.9), 132 (46.9), 256 (44.7), 113 (25.4), 126 (22.9), 242 (19.6), 257 (15.1), 97 (11.2), 131 (5.7), 243 (8.9)	NIST, 646
72	Itaconic acid*	2TMS	13.50	1342.9	16.1	183	147 (99.9), 183 (95.9),	NIST, 746
73	Methylmaleic acid*	2TMS	13.65	1348.9		259	58 (18.2), 84 (14.3), 148 (13.8), 259 (10.5), 215 (9.6), 149 (9.5), 75 (8.9), 184 (7.7), 165 (6.6)	NIST, 729
74	**Fumaric acid***	2TMS	13.74	1352.4 (1352)	1.6	245	245 (99.9), 147 (45.9), 73 (33.3), 246 (18.4), 143 (15.8), 75 (12.9), 247 (8.7), 149 (6.4), 133 (6.3), 148 (6.3)	ST, in-house, NIST, 916
75	**Alanine**	3TMS	13.86	1357 (1357)	4.0	188	188(99.9), 100(52.9), 73(31.5), 147(27.6), 262(21.0), 189(20.2), 132(16.4), 114 (11.8), 133(10.5), 190(8.8)	ST, in-house, NIST, 601
76	RI1359 Unknown (? *p*-Aminobenzoic acid)	-	13.90	1358.7		281		-
77	RI1361 Unknown	-	13.95	1360.7		278		-
78	RI1363 Unknown	-	14.00	1362.5		158		-
79	1-Nitroso-3,5-dinitro-hexahydro-1,3,5-triazine*	-	14.09	1365.9	-	132	132 (99.9), 57 (11.3), 154 (6.9), 133 (6.1), 85 (5.8)	NIST, 825
80	RI1367 Unknown	-	14.12	1367.3		133		-
81	RI1370 Unknown	-	14.19	1370		117		-
82	3,4-Dihydroxybutyric acid γ-lactone*	-	14.23	1371.4		247	147(99.9), 73 (88.3), 247(229), 101(21.3), 148 (14.7), 131(126), 102(12.4), 116(11.8), 134(10.0), 129(9.8), 149(8.6), 132(8.3)	NIST, 839
83	RI1377 Unknown	-	14.38	1377.3		229		-
84	RI1382 Unknown	-	14.51	1382.3		140		-
85	RI1388 Unknown	-	14.65	1387.7		116		-

86	Thymine*	2TMS	14.89	1397.2	20.2	255	255 (99.9), 113 (36.1), 270 (30.6), 147 (28.0), 256 (22.3), 73 (18.7), 120 (10.8), 140 (9.7), 257 (8.4), 271 (7.9), 59 (5.0)	NIST, 900
87	RI1400 unknown	-	14.96	1399.8		239		-
88	RI1401 Carboxylic acid	-	14.98	1400.9		184		-
89	Glutaric acid*	2TMS	15.11	1405.8	1.8	160	147 (99.9), 261 (26.0), 55 (25.7), 158 (22.6), 97 (16.0), 148 (11.8), 160 (10.0), 149 (9.3), 129 (8.9), 143 (8.3), 116 (7.6), 67 (5.3), 145 (5.8), 203 (5.4), 233 (8.0), 262 (7.9)	in-house, NIST, 646
90	3-Deoxytetronic acid (2,4-Dihydroxybutanoic acid)*	3TMS	15.25	1411.6	14.4	103	103 (99.9), 73 (65.6), 147 (52.0), 219 (33.2), 129 (24.7), 229 (19.8), 104 (12.6), 86 (12.2), 145 (11.6), 220 (9.7), 55 (8.3), 68 (5.8), 105 (8.1), 131 (6.4), 158 (6.9)	NIST, 785
91	RI1415 Unknown	-	15.35	1415.8		160		-
92	2-Deoxytetronic acid (3,4-Dihydroxybutanoic acid)*	3TMS	15.76	1432.1	6.9	233	73 (99.9), 147 (38.4), 189 (21.9), 233 (21.8), 231 (14.4), 133 (9.9), 129 (9.5), 132 (9.5), 246 (9.4), 117 (9.1), 74 (8.5), 101 (6.4), 148 (6.8), 157 (7.0), 191 (6.9), 203 (6.7), 202 (5.9), 321 (5.4), 190 (5.0)	NIST, 911
93	RI1434 Unknown	-	15.81	1434		257		-
94	RI1436 Unknown	-	15.85	1435.7		243		-
95	RI1440 Unknown	-	15.95	1439.8		98		-
96	RI1448 Unknown	-	16.14	1447.5		132	132(99.9), 174(64.3), 56(38.8), 73(34.3), 75(29.0), 280(27.4), 156(23.2), 281(22.5), 172(20.3), 133(19.9), 155(18.8), 129(16.0), 130(15.8), 128(10.2)	-
97	RI1451 Unknown	-	16.24	1451.2		295		-
98	RI1453 Unknown	-	16.29	1453.5		98		-
99	Decanoic acid*	1TMS	16.39	1457.5	3.5	117	117 (99.9), 73 (83.0), 75 (77.3), 229 (75.0), 129 (33.1), 132 (29.3), 131 (28.6), 230 (13.6), 76 (12.5), 145 (11.9), 81 (9.7), 55 (7.8), 95 (6.7), 201 (6.6), 118 (6.1), 231 (5.0)	NIST, 894
100	RI1461 Unknown	-	16.47	1460.6		185		-
101	RI1461 Unknown	-	16.49	1461.3		255		-

102	Citramalic acid*	3TMS	16.66	1468.2	5.2	247	73 (99.9), 147 (81.8), 247 (71.9), 75 (48.5), 117 (20.7), 115 (17.8), 185 (17.3), 133 (15.6), 74 (12.0), 149 (11.7), 81 (6.8), 101 (5.7), 160 (7.5), 163 (9.2), 175 (5.5), 188 (8.9), 203 (10.8), 321 (9.0), 285 (5.5)	GDM, NIST, 615
103	RI1469 Unknown	-	16.68	1469.2		359		-
104	RI1473 Unknown	-	16.78	1473		140		-
105	Arabino-Hexos-2-ulose*	4TMS	16.88	1477.2	636.8	234	73 (99.9), 147 (61.1), 234 (40.6), 117 (29.4), 205 (26.5), 103 (23.3), 130 (16.8), 84 (14.2), 133 (12.5), 89 (12.2), 59 (9.3), 72 (7.0), 74 (5.6), 102(10.0), 104 (6.9), 131 (8.6), 132 (10.2), 140 (5.0), 148 (8.3), 149 (9.2), 162 (7.2), 187 (6.2), 217 (11.0), 218 (9.0), 235 (11.9), 245 (6.7)	NIST, 755
106	**Malic acid***	3TMS	17.12	1486.9 (1487)	96.9	233	73 (99.9), 147 (70.2), 233 (23.8), 245 (12.3), 133 (11.6), 189 (10.7), 148 (10.4), 74 (8.8), 55 (8.2), 75 (8.0), 101 (6.8), 117 (6.3), 149 (6.8), 175 (5.9), 191 (6.7), 265 (5.2), 307 (6.8), 335 (5.3)	ST, in-house, NIST, 907
107	Pyroglutamic acid	1TMS	17.36	1496.4	11.6	84	84 (99.9), 73 (25.2), 75 (22.6), 158 (11.7), 157 (11.3), 186 (6.7), 85 (5.8)	in-house, NIST, 893
108	RI1499 Unknown	-	17.41	1498.6		160		-
109	RI1500 Unknown	-	17.46	1500.6		85		-
110	RI1502 Unknown	-	17.49	1501.8		280		-
111	N-acetyl-serine*	2TMS	17.52	1503.1	6.1	116	116 (99.9), 103 (84.4), 186 (71.7), 261 (64.6), 132 (62.6), 174 (56.0), 144 (48.6), 191 (38.2), 100 (34.4), 171 (31.5), 68 (9.3), 101 (21.4), 115 (7.1), 131 (14.1), 145 (13.8), 146 (9.4), 126 (12.7), 158 (25.1), 184 (7.9), 187 (8.2), 190 (6.1), 192 (6.0), 218 (8.3), 244 (5.0), 248 (6.5), 262 (15.1), 263 (8.2), 276 (12.4)	GDM
112	Salicylic acid*	2TMS	17.55	1504.2	0.8	267	267 (99.9), 73 (75.2), 268 (21.8), 209 (12.9), 149 (12.7), 269 (8.7), 135 (8.4), 75 (8.0), 91 (7.6), 193 (6.8), 74 (6.7), 249 (6.0), 133 (5.1),	NIST, 859
113	Adipic acid*	2TMS	17.57	1504.9	91.8	111	111 (99.9), 141 (62.1), 93 (41.5), 55 (41.0), 147 (32.7), 75 (28.0), 185 (26.5), 117 (24.0), 69 (21.7), 143 (21.5), 54 (5.3), 109 (5.1), 112 (7.8), 120 (6.6), 122 (5.8), 138 (5.4), 142 (7.5), 155 (10.3), 159 (13.9), 172 (19.8), 204 (5.4), 210 (12.8), 217 (6.3), 218 (5.9), 275 (17.4)	NIST, 767

114	**Pyroglutamic acid**	2TMS	17.83	1516 (1516)	4	156	156 (99.9), 73 (50.8), 147 (17.7), 157 (13.5), 258 (10.0), 230 (8.1)	ST, in-house, NIST, 954
115	RI1519 Unknown	-	17.91	1519.3		241		-
116	RI1523 Unknown	-	18.01	1523.6		132		-
117	RI1526 Unknown	-	18.07	1525.9		174		-
118	RI1528 Unknown	-	18.13	1528.7		217		-
119	RI1530 Unknown	-	18.15	1529.5		199		-
120	5-Methylcytosine*	2TMS	18.26	1534	1	254	71 (99.9), 57 (81.0), 85 (69.5), 254 (40.9), 73 (32.1), 99 (27.1), 69 (25.1), 70 (18.4), 113 (17.0), 55 (16.2), 56 (11.0), 79 (11.9), 83 (10.9), 84 (13.1), 97 (6.1), 98 (5.9), 103 (5.1), 111 (14.1), 112 (9.3), 125 (7.3), 126 (5.9), 127 (13.3), 130 (5.3), 180 (6.7), 239 (12.5), 255 (10.7), 258 (5.1), 369 (8.6), 281 (5.3), 342 (7.7)	GDM
121	Uracil derivative	-	18.32	1536.7		447		-
122	RI1538 Unknown	-	18.37	1538.7		132		-
123	**Erythronic acid***	4TMS	18.42	1540.9 (1540)	26.1	292	292 (99.9), 205 (35.4), 117 (34.8), 220 (33.1),102 (23.6), 293 (21.0), 130 (20.3), 221 (12.3), 132 (9.1), 58 (9.0), 66 (6.8), 119 (6.2), 131 (7.8), 143 (8.3), 177 (5.6), 207 (5.0), 291 (7.7), 294 (7.5), 319 (5.3)	ST, in-house, NIST, 915
124	RI1543 Unknown	-	18.47	1542.9		188		-
125	RI1549 Unknown	-	18.62	1549.1		219		-
126	RI1554 Unknown	-	18.74	1554		188		-
127	RI1555 Unknown	-	18.77	1555.3		140		-
128	Threonic acid*	4TMS	18.84	1558.5	35.3	292	73 (99.9), 147 (77.9), 292 (58.0), 220 (24.9), 217 (20.1), 117 (20.0), 205 (19.3), 293 (17.2), 130 (16.0), 103 (15.4), 74 (8.7), 102 (15.0), 133 (7.8), 149 (7.4), 221 (8.6), 245 (5.8), 291 (5.8), 319 (6.6)	NIST, 927
129	RI1559 Unknown	-	18.87	1559.6		241		-

130	RI1567 Unknown cinnamaldehyde	-	19.06	1567.4		132		-
131	RI1572 Unknown	-	19.17	1572.3		117		-
132	RI1573 Unknown	-	19.18	1572.5		110		-
133	α-Hydroxyglutaric acid*	3TMS	19.21	1573.8	8.2	247	147 (99.9), 129 (88.5), 247 (42.7), 79 (37.5), 52 (35.3), 148 (30.3), 116 (27.1), 74 (25.0), 203 (21.3), 159 (19.3), 85 (17.7), 86 (13.2), 109 (10.0), 113 (12.4), 115 (13.2), 130 (15.3), 157 (19.0), 169 (14.2), 172 (13.5), 191 (14.9), 213 (6.4), 221 (10.5), 248 (8.3), 250 (5.0), 255 (5.0), 267 (8.9), 271 (7.3), 272 (8.1), 294 (6.1), 309 (8.0), 417 (5.1)	in-house, NIST, 626
134	**Proline [+CO_2]**	2TMS	19.30	1577.6 (1578)	5.2	142	73 (99.9), 75 (89.1), 147 (80.6), 79 (67.5), 58 (61.2), 52 (37.7), 207 (34.7), 69 (21.5), 132 (17.6), 51 (17.2), 50 (11.5), 53 (6.1), 55 (5.9), 56 (6.4), 57 (6.2), 59 (15.9), 66 (6.4), 70 (5.5), 72 (5.2), 76 (8.9), 77 (16.0), 78 (15.9), 80 (5.7), 84 (5.5), 89 (5.4), 127 (12.7), 133 (12.3), 134 (7.6), 142 (12.2), 143 (6.2), 148 (13.5), 149 (8.7), 191 (5.5), 209 (5.7)208 (8.4)	ST, in-house, GDM
135	RI1583 Unknown	-	19.42	1582.7		167		-
136	RI1592 Unknown	-	19.64	1591.9		262		-
137	RI1596 Unknown	-	19.73	1595.9		342		-
138	RI1597 Unknown	-	19.75	1596.6		353		-
139	3-Hydroxy-3-methylglutaric acid*	3TMS	19.81	1598.9	11.1	247	73 (99.9), 147 (68.7), 247 (39.2), 199 (27.4), 231 (23.3), 115 (21.8), 109 (21.7), 273 (20.9), 75 (20.0), 363 (14.9), 55 (12.2), 74 (8.2), 83 (5.7), 117 (13.6), 133 (7.0), 148 (10.1), 149 (10.5), 163 (5.7), 203 (8.7), 204 (5.3), 274 (5.7)	NIST, 899
140	Pimelic acid*	2TMS	19.88	1601.9	8.9	155	73 (99.9), 75 (97.8), 155 (93.9), 125 (65.1), 113 (44.8), 55 (38.5), 149 (31.6), 97 (31.4), 289 (28.8), 173 (27.1),59 (14.1), 61 (8.6), 72 (9.4), 74 (21.7), 76 (14.0), 77 (18.1), 81 (16.3), 84 (16.0), 85 (10.7), 99 (7.9), 112 (7.0), 117 (13.5), 124 (5.9), 131 (14.7), 145 (10.5), 151 (5.7), 156 (11.8), 185 (5.7), 261 (5.7),270 (9.4), 290 (7.5), 298 (5.5)	GDM
141	RI1602 Unknown	-	19.88	1601.9		248		-
142	RI1613 Unknown	-	20.12	1612.7		144		-

143	Arabinonic acid-1,4-lactone*	3TMS	20.34	1622.2	59.8	217	73 (99.9), 217 (55.4), 147 (50.6), 129 (35.4), 130 (21.7), 220 (20.7), 102 (16.4), 133 (13.5), 218 (12.4), 103 (12.1), 55 (5.5), 74 (8.2), 75 (11.1), 131 (7.8), 148 (8.0), 189 (5.4), 219 (5.2), 231 (10.7), 349 (5.9)	NIST, 887
144	*p*-Hydroxybenzoic acid*	2TMS	20.37	1623.8	2.7	267	267 (99.9),223 (76.4), 193 (38.2), 132 (27.4), 268 (25.8), 282 (25.1), 224 (21.0), 160 (19.1), 96 (18.6), 126 (16.9), 68 (6.0), 89 (5.2), 91 (5.8), 103 (8.8), 116(6.7), 133 (8.3), 135 (5.9), 194 (6.3), 207 (12.2),225 (5.7), 269 (12.0)	NIST, 826
145	RI1626 Unknown	-	20.41	1625.5		202		-
146	RI1629 Unknown	-	20.49	1629.1		246		-
147	Xylonic acid-1,4-lactone*	3TMS	20.50	1629.5	35.5	117	73 (99.9), 117 (69.8), 217 (22.4), 246 (18.4), 130(17.9), 204 (14.8), 189 (14.3), 207 (14.3), 58 (13.5), 102 (12.0), 59 (7.7), 118 (7.7), 119 (6.6), 133 (5.9), 142 (6.1), 140 (5.0), 143 (5.3), 144 (11.1), 191 (5.7), 216 (7.9), 232 (6.3), 259 (10.1), 260 (5.8)364 (5.0)	NIST, 656
148	RI1636 Unknown	-	20.66	1636.4		132		-
149	RI1643 Unknown	-	20.82	1643.3		226		-
150	RI1646 Unknown	-	20.88	1645.9		355		-
151	Ribonic acid-14-lactone*	3TMS	20.88	1645.9	16.1	117	73 (99.9), 147 (32.1), 117 (24.5), 75 (19.9), 69 (15.6), 133 (14.2), 57 (13.2), 245 (12.7), 58 (12.2), 71 (10.0), 74 (6.0), 78 (9.3), 85 (6.7), 102 (8.6), 132 (8.0), 148 (10.1), 215 (8.9), 217 (11.8), 246 (10.2), 281 (9.9)	NIST, 678
152	RI1646 Unknown	-	20.88	1646		245		-
153	RI1650 Unknown (indol-derivate)	-	20.97	1649.9		202		-
154	Dodecanoic acid*	1TMS	20.99	1651	0	257	117 (99.9), 257 (87.0), 73 (81.8), 75 (64.2), 132 (40.8), 129 (36.4), 131 (21.7), 145 (17.8), 258 (16.7), 55 (15.4), 57 (6.9), 69 (6.7), 76 (5.0), 81 (5.7), 83 (5.6), 95 (8.2), 118 (8.9), 133 (6.9),	in-house, NIST, 916
155	RI1656 Carbohydrate	-	21.10	1656		217		-
156	RI1657 Unknown (indol-derivate)	-	21.13	1656.9		202		-
157	RI1666 Unknown	-	21.34	1666.3		140		-

158	RI1673 Carbohydrate	-	21.49	1673.1		217		-
159	RI1678 Unknown	-	21.62	1678.6		141		-
160	RI1688 Unknown	-	21.84	1688.4		132		-
161	Xylitol*	5TMS	21.93	1692.5	11.2	307	73 (99.9), 217 (64.3), 307 (59.0), 319 (53.7), 147 (50.7), 103 (48.5), 129 (32.5), 308 (17.1), 157 (15.6), 320 (14.9), 117 (8.6), 133 (7.7), 148 (8.0), 149 (5.0), 189 (6.4), 205 (13.5), 219 (6.2), 229 (6.6), 243 (6.0), 277 (7.3), 306 (10.9), 309 (8.5), 321 (8.0)	NIST, 864
162	RI1696 Carbohydrate	-	22.02	1696.2		217		-
163	RI1703 Unknown	-	22.18	1703.4		157		-
164	RI1707 Unknown	-	22.27	1707.7		132		-
165	C5-Sugar alcochol	-	22.45	1716.1		217		-
166	Ribitol*	5TMS	22.55	1720.7	6.4	217	217 (99.9), 73 (79.5), 147 (70.8), 103 (52.0), 155 (38.2), 319 (36.5), 205 (35.6), 307 (30.8), 212 (28.7), 57 (11.1), 71 (16.5), 85 (13.1), 89 (6.9), 91 (6.6), 104 (6.6), 115 (6.0), 117 (20.5), 129 (26.0), 141 (11.7), 152 (6.0), 153 (10.4), 154 (6.9), 157 (9.8), 169 (5.3), 189 (19.5), 190 (5.2), 198 (6.1), 204 (16.8), 206 (7.6), 218 (26.7), 219 (7.4), 229 (6.7), 277 (5.9), 308 (5.0), 320 (12.6), 321 (6.0), 332 (10.4)	NIST, 850
167	Glycerol-2-phosphate*	4TMS	22.57	1721.2	22.8	299	243 (99.9), 299 (97.0), 211 (36.2), 140 (28.0), 300 (26.2), 315 (22.3), 389 (22.2), 244 (18.1), 301 (15.8), 445 (12.3), 136 (11.1), 267 (10.5), 285 (10.0), 373 (11.2)	in-house, NIST, 715
168	RI1725 Unknown	-	22.66	1725.4		208		-
169	RI1726 Unknown	-	22.68	1726.4		357		-
170	RI1730 Lyxonic acid-1,4-lactone or Arabinonic acid γ-lactone*	3TMS	22.75	1729.7	119.3	217	73 (99.9), 147 (37.7), 217 (20.0), 117 (19.7), 246 (15.5), 204 (11.3), 102 (11.0), 130 (10.0), 75 (9.5), 231 (9.5), 69 (7.3), 74 (8.9), 103 (7.6), 133 (7.3), 148 (5.8), 149 (7.1), 259 (6.5), 364 (6.6)	NIST, 906
171	RI1731 Unknown	-	22.79	1731.4		215		-
172	RI1733 Unknown	-	22.82	1733		186		-
173	RI1735 Carbohydrate	-	22.87	1735.5		217		-
174	RI1743 Unknown	-	23.05	1743.4		140		-

175	RI1744 Unknown	-	23.06	1744.1		215		-
176	RI1745 Unknown	-	23.08	1744.9		233		-
177	RI1747 Sugar-derived acid	-	23.13	1747.1		333		-
178	**Aconitic acid***	3TMS	23.13	1747.5 (1747)	3.5	229	147 (99.9), 73 (94.7), 229 (41.6), 211 (26.2), 375 (21.9), 285 (20.0), 148 (17.5), 149 (13.2), 75 (11.6), 215 (11.4), 67 (11.3), 74 (7.7), 133 (11.2), 230 (10.8), 376 (6.4)	ST, in-house, NIST, 896
179	**Arabinose 1**	4TMS	23.30	1755 (1755)	7.0	307	103(99.9), 217(89.5), 307(67.0), 73(59.6), 147(51.9), 218(23.1), 308(21.3), 256(16.0), 189(13.8), 320(13.2), 219(10.7), 129(9.8), 148(9.8), 291(8.9), 204(8.5), 309(5.4)	ST, in-house, NIST, 688
180	Glycerol-3-phosphate*	4TMS	23.38	1758.8	14.8	299	299 (99.9), 357 (92.6), 147 (44.0), 315 (30.8), 103 (27.1), 300 (25.6), 358 (25.2), 445 (21.9), 129 (19.7), 370 (17.3), 59 (6.4), 74 (10.2), 75 (14.6), 101 (12.7), 131 (9.2), 133 (12.8), 148 (6.9), 149 (5.4), 156 (12.6), 186 (5.8), 211 (11.8), 214 (5.8), 218 (9.8), 256 (9.2), 293 (6.0), 301 (13.9), 314 (5.9), 316 (8.1), 371 (5.6), 373 (7.8), 387 (11.5), 389 (5.9), 446 (8.1),	in-house, NIST, 927
181	**Arabinose 2**	4TMS	23.38	1758.9 (1760)	-	307	103(99.9), 217(89.5), 307(67.0), 73(59.6), 147(51.9), 218(23.1), 308(21.3), 256(16.0), 189(13.8), 320(13.2), 219(10.7), 129(9.8), 148(9.8), 291(8.9), 204(8.5), 309(5.4)	ST, in-house
182	RI1764 C5-Carbohydrate	-	23.50	1764.5		217		-
183	RI1767 Unknown	-	23.56	1767		332		-
184	Arabinonic acid*	5TMS	23.59	1768.4	43.6	292	292 (99.9), 103 (56.1), 129 (52.3), 293 (27.4), 205 (22.9), 333 (22.3), 155 (22.1), 307 (16.9), 305 (16.4), 207 (15.2), 66 (5.3), 69 (5.9), 104 (6.3), 132 (13.2), 134 (5.2), 143 (11.3), 191 (8.3), 192 (5.3), 223 (5.3), 244 (6.8), 246 (10.0), 277 (7.5), 306 (5.7), 308 (5.8), 331 (7.2),	GDM, NIST, 899
185	RI1776 C5-Sugar-derived acid	-	23.75	1776		292		-
186	RI1778 Unknown	-	23.77	1776.7		253		-
187	RI1781 Unknown	-	23.86	1780.9		290		-
188	RI1782 C5-Carbohydrate	-	23.89	1782.4		217		-

189	1,4-Benzenedicarboxylic acid*	2TMS	23.92	1783.7	4.3	295	295 (99.9), 296 (28.7), 221 (26.2), 251 (26.0), 429 (25.1), 103 (17.9), 204 (15.6), 79 (14.7), 71 (12.5), 57 (12.2), 85 (11.0), 109 (5.1), 135 (7.1), 169 (5.4), 192 (8.3), 252 (5.5), 297 (10.7), 310 (8.7), 341 (7.1), 342 (6.1), 355 (9.4), 430 (6.2), 431 (7.3)	NIST, 812
190	*p*-Coumaric acid (?)*	2TMS	23.93	1784.4	139.6	293	73 (99.9), 293 (82.5), 219 (70.3), 249 (56.1), 308 (39.9), 75 (31.1), 294 (27.4), 147 (26.0), 179 (25.1), 221 (18.4), 74 (13.0), 72 (9.6), 91 (6.5), 103 (11.5), 116 (5.9), 117 (16.7), 119 (15.6), 134 (6.1), 135 (7.6), 139 (9.5), 156 (5.6), 159 (5.1), 176 (5.5), 177 (6.5), 178 (17.2), 190 (5.0), 191 (11.3), 192 (6.1), 203 (9.8), 218 (5.4), 220 (11.4), 222 (6.9), 223 (12.8), 233 (12.6), 237 (7.7), 250 (13.5), 251 (12.0), 309 (8.5), 312 (11.2)	NIST, 843
191	RI1785 Unknown	-	23.94	1784.9		312		-
192	Azelaic acid*	2TMS	24.13	1793.7	4.7	317	73 (99.9), 317 (74.1), 149 (64.4), 75 (56.8), 55 (48.5), 129 (43.9), 117 (40.8), 201 (37.3), 152 (32.4), 147 (27.0), 67 (21.3), 69 (11.7), 72 (5.9), 74 (7.6), 76 (5.3), 79 (15.2), 81 (11.1), 83 (22.9), 85 (5.0), 93 (10.6), 97 (13.4), 99 (6.5), 107 (17.6), 109 (8.5), 111 (17.3), 123 (12.7), 124 (11.3), 125 (8.2), 130 (7.7), 131 (9.6), 135 (6.1), 148 (8.2), 150 (6.7), 151 (7.1), 153 (7.5), 171 (5.5), 183 (8.3), 185 (6.8), 204 (17.1), 214 (5.6), 217 (10.9), 225 (6.5), 273 (6.0), 318 (15.0), 319 (8.1)	NIST, 869
193	RI1797 Sugar-derived acid	-	24.21	1797.4		292		-
194	Fructofuranose	5TMS	24.24	1798.7	50.3	217	217 (99.9), 437 (51.9), 71 (38.4), 218 (25.0), 140 (23.7), 257 (23.6), 207 (15.5), 210 (14.7), 69 (14.4), 57 (12.0), 58 (9.4), 83 (5.9), 85 (10.3), 89 (9.0), 103 (7.6), 112 (6.7), 120 (5.0), 133 (5.7), 141 (9.7), 145 (11.2), 146 (5.1), 157 (6.6), 215 (5.5), 239 (9.7), 253 (8.8), 258 (6.3), 331 (6.1), 347 (5.8), 438 (8.9), 439 (9.9)	NIST, 658
195	RI1802 Unknown	-	24.31	1801.9		314		-
196	RI1807 Fructofuranose	5TMS	24.42	1807		217		NIST, 798
197	RI1808 C6-Carbohydrate	-	24.43	1807.9		437		-
198	Shikimic acid*	4TMS	24.45	1808.5	1.3	204	204 (99.9), 205 (13.6), 255 (8.0), 357 (6.7), 372 (6.5), 254 (5.6), 206 (5.3)	NIST, 905
199	RI1813 Unknown	-	24.54	1813		214		-
200	**Citric acid***	4TMS	24.56	1814.1 (1814)	1.6	273	73 (99.9), 147 (55.8), 273 (42.5), 148 (12.2), 347 (12.2), 274 (10.0), 207 (9.5), 131 (8.7), 211 (8.2), 363 (8.2), 67 (5.9), 117 (7.9), 129 (8.1), 211 (8.2), 221 (5.6), 363 (8.2)	ST, in-house, NIST, 762

201	Isocitric acid 1	4TMS	24.64	1818.1	20.9	273	147 (99.9), 73 (99.5), 273 (97.0), 245 (67.9), 319 (37.0), 375 (35.1), 274 (23.4), 347 (22.4), 83 (20.7), 129 (19.2), 55 (10.3), 67 (5.1), 74 (13.9), 84 (6.1), 128 (6.6), 143 (17.4), 156 (6.2), 171 (5.0), 191 (11.8), 203 (5.4), 201 (11.5), 217 (14.3), 219 (7.7), 221 (10.9), 237 (6.2), 246 (14.4), 247 (7.8), 275 (7.3), 283 (5.2), 285 (9.0), 305 (11.7), 320 (12.6), 321 (5.7), 349 (6.1), 348 (9.7), 349 (6.1), 363 (15.1), 377 (5.9), 390 (5.8), 465 (14.6)	NIST, 855
202	RI1822 Unknown	-	24.73	1822.3		203		-
203	Isocitric acid 2	4TMS	24.85	1827.9	11.1	273	147 (99.9), 73 (99.1), 75 (78.8), 273 (72.8), 245 (48.7), 375 (32.6), 83 (21.1), 148 (20.0), 246 (17.7), 140 (16.3), 52 (13.6), 68 (5.4), 70 (7.7), 76 (7.1), 84 (6.6), 100 (6.4), 101 (7.2), 116 (5.9), 129 (10.4), 133 (11.1), 139 (6.3), 143 (8.3), 145 (5.0), 169 (9.0), 185 (9.7), 191 (8.4), 211 (9.8), 212 (7.8), 222 (9.1), 223 (6.3), 234 (5.4), 247 (10.2), 257 (6.4), 261 (13.3), 263 (14.3), 275 (5.9), 283 (6.1), 284 (5.3), 285 (10.1), 286 (5.3), 287 (5.2), 299 (8.8), 306 (6.8), 321 (6.9), 349 (7.7), 374 (5.6), 376 (14.4), 377 (8.0), 466 (7.7)	NIST, 698
204	RI1830 Unknown	-	24.89	1829.9		382		-
205	RI1833 Unknown	-	24.96	1833.3		349		-
206	RI1835 Unknown	-	25.00	1835		369		-
207	RI1838 Unknown	-	25.05	1837.6		132		-
208	RI1839 Unknown	-	25.08	1839		261		-
209	RI1844 Unknown	-	25.19	1844.3		261		-
210	RI1846 Unknown	-	25.22	1845.7		188		-
211	Myristic acid (Tetradecanoic acid)*	1TMS	25.22	1846.1	4.0	285	117 (99.9), 285 (86.3), 73 (72.9), 75 (57.8), 129 (40.3), 131 (22.7), 145 (20.6), 55 (15.7), 286 (15.6), 118 (10.2), 57 (6.4), 69 (7.5), 81 (5.4), 95 (6.4), 201 (5.6), 300 (5.1)	NIST, 852
212	Quinic acid*	5TMS	25.36	1852.4	9.8	345	345 (99.9), 73 (46.5), 255 (44.0), 346 30.8), 147 (19.7), 347 (14.0), 208 (10.8), 256 (10.2), 217 (8.6), 334 (8.6), 246 (7.3), 276 (5.2), 319 (6.0), 372 (5.1)	GDM
213	RI1855 Unknown	-	25.41	1854.9		233		-

214	Adenine*	2TMS	25.47	1858.2	5.8	264	264 (99.9), 221 (58.4), 279 (27.1), 295 (25.2), 265 (24.4), 207 (24.0), 281(23.3), 149 (19.1), 222 (12.5), 166 (11.2), 132 (5.0), 208 (7.0), 192 (6.6), 209 (6.5), 223 (7.8), 282 (6.6), 297 (5.0)	GDM, NIST, 783
215	RI1859 Unknown	-	25.48	1858.8		245		-
216	RI1860 Unknown	-	25.52	1860.2		204		-
217	RI1862 Unknown	-	25.55	1862		233		-
218	RI1865 Unknown	-	25.62	1865.4		174		-
219	RI1870 Sugar-derived acid	-	25.73	1870.7		292		-
220	RI1871 Unknown	-	25.74	1871.1		143		-
221	RI1872 Unknown	-	25.76	1871.8		355		-
222	Gluconic acid δ-lactone*	4TMS	25.89	1878.5	3.8	319	73 (99.9),129 (50.6), 147 (37.0), 319 (32.9), 220 (21.5), 157 (12.8), 229 (12.7), 103 (10.3), 320 (10.2), 130 (9.5), 74 (8.4), 75 (6.7), 117 (5.5), 133 (8.6), 143 (5.6), 189 (9.2), 204 (6.9), 321 (5.0),	NIST, 916
223	RI1880 Unknown	-	25.93	1880		186		-
224	RI1881 Unknown	-	25.96	1881.4		217		-
225	RI1886 Unknown	-	26.06	1886.4		204		-
226	RI1889 Sugar-derived acid	-	26.15	1888.9		217		-
227	RI1891 Carbohydrate	-	26.15	1891		204		-
228	RI1893 Unknown	-	26.20	1893.2		233		-
229	RI1896 C5- or C6-Carbohydrate	-	26.26	1896.2		204		-
230	RI1898 Unknown	-	26.30	1897.8		233		-

231	Glucaric acid-1,4-lactone*	4TMS	26.37	1901.5	42.5	217	73 (99.9), 217 (73.7), 147 (36.2), 174 (22.7), 205 (20.6), 189 (15.9), 292 (14.6), 218 (13.1), 291 (12.8), 465 (12.3), 56 (7.7), 67 (6.8), 50 (5.2), 102 (6.0), 116 (7.1), 130 (6.2), 135 (5.9), 143 (11.0), 148 (8.8), 175 (9.7), 177 (11.3), 190 (6.3), 191 (10.7), 204 (6.8), 208 (6.2), 219 (10.1), 220 (8.3), 221 (9.2), 244 (5.7), 261 (6.8), 285 (5.8), 293 (5.5), 306 (5.4), 319 (9.0), 331 (7.2), 333 (7.1), 334 (6.6), 335 (5.6)	GDM, NIST, 770
232	RI1907 Sugar-derived acid	-	26.48	1907.1		217		-
233	RI1915 C6-Carbohydrate	-	26.64	1915.4		275		-
234	RI1918 Unknown	-	26.70	1917.9		132		-
235	RI1921 Unknown	-	26.75	1921		300		-
236	RI1922 C5- or C6-Carbohydrate	-	26.78	1922.2		319		-
237	RI1929 Sugar alcochol	-	26.91	1928.8		319		-
238	RI1930 C5- or C6-Carbohydrate	-	26.94	1930.1		204		-
239	*trans*-2-hydroxy-Cinnamic acid (*o*-Coumaric acid)*	2TMS	27.00	1933.7	8.7	293	293 (99.9),74 (75.5), 249 (66.1), 219 (64.4), 308 (59.3), 294 (26.0), 179 (18.8), 309 (18.3), 250 (15.5), 220 (15.2), 74 (8.1), 115 (7.3), 135 (.6), 139 (10.7), 175 (5.8), 191 (5.6), 192 (6.8), 203 (7.8), 221 (6.4), 223 (7.1), 233 (9.1), 251 (6.1), 310 (5.8)	NIST, 903
240	Fructose 1	1MEOX, 5TMS	27.03	1934.8	81.8	217	217 (99.9), 73 (82.8), 103 (71.4), 307 (59.5), 147 (48.4), 422 (16.2), 129 (11.2), 277 (11.2), 117 (10.0), 205 (10.0), 74 (7.4), 148 (9.1), 157 (5.2), 189 (5.8), 191 (6.7), 204 (5.6), 231 (5.7), 263 (6.2), 321 (6.4), 423 (6.8)	in-house, NIST, 825
241	RI1941 Unknown	-	27.14	1940.7		140		-
242	Fructose 2	1MEOX, 5TMS	27.19	1942.9	79.9	217	217 (99.9), 73 (88.9), 103 (78.3), 307 (59.9), 439 (34.1), 218 (26.2), 422 (20.4), 308 (19.7), 148 (13.7), 133 (12.6), 56 (6.0), 74 (7.9), 79 (8.3), 86 (8.3), 113 (5.7), 146 (5.7), 157 (5.9), 174 (7.4), 201 (5.3), 219 (7.0), 309 (9.7), 423 (6.6), 441 (5.4), 454 (7.8)	in-house, NIST, 782
243	*n*-Valeric acid (*n*-Pentanoic acid)*	1TMS	27.21	1944.4	2.4	299	117 (99.9), 299 (81.8), 75 (63.4), 132 (58.0), 129 (33.7), 184 (29.1), 145 (27.4), 85 (25.3), 57 (21.5), 69 (21.3), 55 (11.1), 67 (6.8), 71 (14.6), 81 (6.6), 83 (12.9), 95 (8.8), 99	NIST, 756

							(10.0), 109 (6.0), 116 (7.6), 118 (9.9), 125 (6.8), 127 (9.0), 131 (18.2), 155 (5.0), 166 (5.3), 169 (6.1), 185 (6.0), 285 (6.7), 300 (20.1), 301 (8.8)	
244	RI1930 C5- or C6-Carbohydrate	-	27.27	1947.4		204		-
245	RI1947 Unknown	-	27.28	1947.1		230		-
246	RI1949 Carbohydrate	-	27.31	1949.3		361		-
247	RI1952 Carbohydrate	-	27.37	1952.4		319		-
248	RI1957 Sugar-derived acid	6TMS	27.46	1956.7	52.3	333	147 (99.9), 292 (70.4), 333 (64.5), 319 (47.5), 85 (38.6), 84 (29.0), 148 (25.7), 217 (25.6), 205 (25.5), 129 (21.7), 99 (17.2), 143 (17.1), 159 (12.1), 168 (10.0), 191 (17.2), 305 (14.9), 321 (9.8), 332 (9.4), 335 (12.7), 369 (9.8), 429 (7.6)	GDM
249	RI1959 Unknown	-	27.51	1959		174		-
250	Lysine	4TMS	27.61	1964.6	23.6	174	200 (99.9), 174 (88.8), 317 (35.2), 302 (33.1), 72 (29.9), 201 (25.7), 319 (20.6), 173 (15.3), 195 (12.3), 202 (11.7), 230 (11.5), 272 (10.6), 303 (11.5), 318 (10.9), 332 (7.4), 398 (10.4)	GDM, NIST,
251	RI1970 Sugar-derived acid	-	27.73	1969.7		217		-
252	RI1974 Sugar-derived acid	-	27.73	1970.6		217		-
253	*cys*-Caffeic acid*	3TMS	27.76	1972.3	0	219	219 (99.9), 396 (67.5), 73 (46.7), 397 (22.6), 381 (17.6), 112 (17.4), 220 (17.4), 398 (10.5), 169 (10.3), 249 (9.3), 307 (8.7), 382 (6.5), 74 (5.5)	NIST, 892
254	RI1975 Carbohydrate	-	27.82	1975.3		204		-
255	**Galactose***	1MEOX, 5TMS	27.91	1979.6 (1979)	69.6	319	73 (99.9), 319 (97.2), 147 (54.3), 205 (46.6), 217 (30.5), 320 (30.5), 129 (25.8), 103 (24.4), 218 (23.1), 157 (21.9), 52 (6.4), 74 (13.6), 78 (5.0), 117 (10.0), 131 (6.4), 133 (6.5), 148 (9.6), 149 (6.6), 189 (6.0), 191 (8.1), 206 (10.2), 207 (9.8), 219 (8.7), 229 (8.2), 277 (8.1), 281 (6.0), 307 (6.1)	ST, in-house, NIST, 889
256	RI1984 Sugar derived acid	-	27.99	1983.9		333		-

257	Mannose*	1MEOX, 5TMS	28.00	1984.3	84.3	319	73 (99.9), 319 (85.3), 147 (74.4), 205 (44.5), 320 (26.4), 157 (24.9), 129 (23.9), 103 (23.7), 218 (18.5), 217 (13.1), 100 (6.6), 117 (11.9), 133 (9.2), 148 (9.8), 159 (5.0), 191 (5.9), 201 (5.0), 206 (7.6), 229 (7.0), 259 (5.3), 291 (8.6), 292 (5.1), 321 (13.0), 374 (10.6)	in-house, NIST, 752
258	RI1988 Sugar-derived acid	-	28.08	1988.4		292		-
259	RI1989 Amine	-	28.08	1988.5		174		-
260	RI1991 Sugar-derived acid (Gluconic acid)*	-	28.13	1990.7		333	73(99.9), 333(49.6), 292(47.0), 147(37.5), 305(20.4), 103(18.9), 217(15.0), 334(14.6), 293(10.3), 359(8.5), 74(7.9), 277(7.8), 189(6.2), 306(5.8), 129(5.3), 143(5.2)	NIST, 795
261	**Glucose 1**	1MEOX, 5TMS	28.13	1990.5 (1990)	110.5	319	319 (99.9), 205 (36.8), 320 (32.2), 157 (17.2), 218 (14.4), 129 (13.6), 321 (13.5), 206 (8.7), 229 (8.3), 219 (6.8)	ST, in-house
262	RI1995 Unknown	-	28.26	1997.5		293		-
263	RI1999 Phenolic compound	-	28.29	1999.1		219		-
264	RI2001 Unknown	-	28.32	2000.8		105		-
265	RI2006 Sugar-derived acid	-	28.42	2005.7		333		-
266	**Glucose 2**	1MEOX, 5TMS	28.45	2007.2 (2007)	108.2	319	73 (99.9), 221 (86.1), 147 (66.7), 295 (49.5), 319 (38.1), 281 (28.9), 207 (26.6), 222 (21.0), 369 (20.4), 205 (18.9), 74 (8.9), 103 (10.0), 129 (9.6), 133 (5.0), 148 (9.7), 149 (5.1), 157 (7.5), 208 (5.0), 217 (9.2), 218 (6.6), 282 (8.6), 297 (8.9), 320 (10.3), 321 (5.3), 341 (5.3)	ST, in-house, NIST, 665
267	Palmitelaidic acid (*trans*-9-hexadecenoic acid)*	1TMS	28.62	2015.8	0.5	117	132 (99.9), 117 (68.2), 73 (41.9), 96 (18.8), 129 (17.5), 311 (17.4), 171 (15.7), 116 (14.9), 95 (14.3), 55 (14.0), 84 (12.5), 61 (5.3), 86 (5.8), 97 (7.9), 102 (9.1), 111 (6.9), 118 (7.3), 145 (6.8), 149 (9,4), 152 (5.3), 161 (5.5), 172 (6.5), 174 (13.8), 183 (5.5), 199 (6.9), 200 (5.9), 216 (5.3), 246 (9.7), 262 (6.2), 269 (5.7), 290 (9.4), 305 (9.2)	NIST, 655
268	RI2019 Carbohydrate	-	28.69	2019.2		204		-
269	RI2024 Carbohydrate	-	28.79	2024.8		204		-

270	RI2024 Unknown	-	28.80	2024.8		132		-
271	RI2027 Unknown	-	28.83	2026.5		294		-
272	RI2031 Amine	-	28.92	2031.1		174		-
273	RI2034 Sugar-derived acid	-	28.97	2033.8		333		-
274	RI2035 Carbohydrate	-	29.01	2035.3		361		-
275	RI2037 Amine	-	29.04	2037		174		-
276	Palmitic acid*	1TMS	29.12	2041.4	2.3	313	117 (99.9),313 (77.1), 73 (66.2), 75 (49.0), 132 (48.9), 129 (37.3), 145 (26.2), 314 (18.2), 131 (17.1), 55 (15.0), 57 (77.4), 69 (8.6), 74 (6.6), 81 (5.6), 83 (6.0), 95 (6.2), 118 (9.7), 133 (9.9), 201 (7.4), 315 (5.1)	NIST, 934
277	RI2053 Unknown	-	29.35	2052.9		267		-
278	RI2057 Unknown	-	29.43	2056.9		132		-
279	RI2066 Carbohydrate	-	29.61	2066.1		204		-
280	RI2071 Phenolic compound	-	29.71	2071.1		355		-
281	Oleanitrile*	-	29.77	2074.5		122		-
282	**Myo-inositol***	6TMS	29.82	2076.9 (2078)	3.2	305	305 (99.9), 73 (92.0), 217 (79.7),147 (53.0), 318 (52.6), 191 (42.8), 306 (26.7), 319 (22.4), 265 (21.4), 129 (18.6), 74 (7.8),103 (15.5), 133 (11.6), 148 (7.9), 192 (5.7), 204 (13.0), 218 (14.4), 219 (6.9), 221 (6.6), 291 (5.5), 307 (14.3), 367 (6.1), 432 (12.1), 433 (9.9)	ST, in-house, NIST, 935
283	Ferulic acid*	2TMS	29.92	2082.1	3.9	338	73 (99.9), 338 (94.1), 323 (69.5), 308 (54.2), 293 (42.3), 249 (35.7), 131 (29.6), 339 (25.3), 147 (17.3), 279 (16.4), 75 (16.2), 74 (9.4), 84 (5.3), 89 (8.1), 103 (5.8), 115 (5.6), 116 (7.6), 117 (7.5), 132 (7.9), 139 (5.5), 144 (6.1), 145 (6.1), 146 (7.5), 149 (5.9), 154 (5.5), 158 (8.9), 174 (16.3), 175 (8.9), 179 (7.3), 191 (6.7), 204 (9.2), 207 (8.7), 217 (9.9), 218 (9.5), 219 (16.1), 226 (6.0), 233 (10.5), 250 (10.8), 260 (7.3), 277(8.1), 294 (9.3), 309 (8.6), 324 (16.3), 325 (6.2), 327 (6.9), 340 (8.2)	in-house, NIST, 929

284	N-Acetyl-α-D-glucosamine*	4TMS	29.94	2082.8	54.2	173	173 (99.9), 73 (73.3), 131 (27,4), 217 (17.0), 147 (16.6), 174 (15.8), 55 (14.0), 136 (12.9), 75 (12.8), 259 (12.3), 57 (6.4), 69 (5.7), 81 (5.0), 83 (5.6), 84 (6.4), 103 (5.5), 116 (9.2), 133 (5.5), 140 (5.0), 143 (5.9), 156 (10.0), 158 (8.4),186 (6.4), 192 (5.1), 204 (9.5), 226 (5.3), 233 (7.3), 260 (7.0), 314 (6.9)	in-house
285	RI2087 Unknown	-	30.02	2087.1		204		-
286	RI2093 Unknown	-	30.15	2093.5		132		-
287	Adenine - derivate	-	30.20	2096.2		264		-
288	Benzenedicarboxylic acid derivative	-	30.24	2098.3		221		GDM
289	RI2105 Unknown	-	30.38	2105.4		210		-
290	RI2108 Unknown	-	30.44	2107.9		327		-
291	RI2114 Amine	-	30.54	2113.6		174		-
292	RI2116 Carbohydrate	-	30.58	2115.7		204		-
293	*trans*-Caffeic acid 1	3TMS	30.77	2124.9	18.1	219	219 (99.9), 396 (81.7), 73 (56.7), 397 (27.5), 381 (23.0), 220 (19.7), 398 (13.2), 249 (10.9), 109 (10.5), 191 (10.3), 126 (6.4), 137 (7.2), 253 (6.8), 267 (5.2), 293 (5.0), 301 (5.5), 355 (9.5), 370 (6.6), 382 (7.9), 383 (5.2)	NIST, 917
294	RI2129 Carbohydrate	-	30.86	2129.5		204		-
295	Heptadecanoic acid*	1TMS	30.95	2134.3	5.7	327	117 (99.9), 73 (90.9), 327 (85.2), 129 (43.0), 132 (42.6), 75 (28.2), 328 (23.2), 221 (23.0), 55(21.0), 145 (20.9), 57 (10.6), 69 (8.4), 74 (9.5), 81 (7.1), 83 (5.8), 84 (5.3), 95 (6.3), 96 (5.2), 103 (13.6), 104 (6.1), 131 (17.3), 118 (9.6), 134 (6.4), 143 (8.2), 187 (5.4), 201 (10.8), 217 (7.3), 283 (6.0), 295 (18.4), 296 (8.1), 299 (9.8), 311 (6.6), 313 (5.0), 329 (6.4), 342 (8.9)	NIST, 820
296	*trans*-Caffeic acid 2	3TMS	31.02	2137.6		219	219(99.9), 396 (69.6), 73(48.8), 397(29.7), 381(23.1), 204(18.1) , 220(15.7), 398(13.1), 382(10.8), 249(9.1), 189(6.2), 267(5.3)	NIST, 827
297	RI2146 Unknown	-	31.17	2145.6		369		-
298	RI2157 Unknown	-	31.41	2157.7		140		-

299	Phytol*	1TMS	31.45	2159.7	2.8	143	143 (99.9), 73(22.4), 75 (14.9), 123 (14.0), 144 (13.7), 81 (9.2), 124 (6.3), 69 (5.7), 95 (5.0)	NIST, 899
300	RI2161 Unknown	-	31.48	2161.4		140		-
301	RI2163 Unknown	-	31.52	2163.2		387		-
302	RI2170 Unknown	-	31.64	2169.5		249		-
303	RI2170 Unknown	-	31.66	2170.4		140		-
304	RI20172 Carbohydrate	-	31.70	2172.1		204		-
305	Glycerophosphoglycerol*	5TMS	31.87	2181.2	37.8	357	73 (99.9), 357 (87.9), 147 (66.7), 445 (60.4), 103 (57.3), 299 (52.4), 129 (50.7), 503 (41.1), 358 (40.4), 315 (28.6), 74 (10.0), 83 (11.5), 87 (8.9), 101 (23.1), 105 (13.2), 108 (7.2), 130 (9.1), 131 (20.9), 132 (19.9), 133 (22.7), 140 (9.8), 141 (10.3), 143 (6.5), 149 (25.5), 191 (18.8), 207 (21.0), 211 (19.1), 213 (13.3), 225 (7.4), 227 (9.7), 241 (5.4), 243 (9.0), 251 (6.2), 253 (7.4), 257 (13.0), 283 (5.8), 285 (15.8), 295 (13.1), 300 (25.1), 301 (5.7), 311 (6.1), 312 (15.1), 316 (6.1), 329 (8.6), 341 (10.7), 359 (18.2), 361 (8.0), 370 (8.1), 371 (9.6), 373 (16.6), 374 (5.3), 387 (6.0), 388 (7.6), 389 (12.1), 390 (6.4), 446 (14.7), 447 (5.6), 461 (12.6), 504 (13.7), 505 (8.7), 516 (19.4), 517 (9.1), 518 (5.0)	GDM, NIST, 803
306	RI2189 Unknown	-	32.03	2189.3		276		-
307	RI2191 Unknown Phosphate	-	32.07	2191.1		299		-
308	Linoleic acid*	1TMS	32.10	2192.7	9.3	337	73 (99.9), 75 (97.1), 81 (95.3), 67 (78.1), 337 (66.3), 95 (61.5), 55 (43.6), 96 (43.6), 82 (43.3), 262 (43.0), 54 (23.0), 57 (5.9), 61 (7.4), 66 (5.6), 68 (30.7), 69 (26.5), 74 (10.4), 77 (13.7), 78 (6.3), 79 (41.0), 80 (31.8), 83 (22.1), 84 (5.8), 85 (5.7), 91 (14.3), 93 (29.3), 94 (27.5), 97 (22.5), 105 (9.8), 107 (20.4), 108 (21.6), 109(33.8), 110 (25.5), 111 (10.0), 117 (34.4), 119 (9.8), 120 (5.7), 121 (31.7), 122 (16.7), 123 (19.3), 124 (15.4), 125 (6.3), 129 (40.5), 130 (5.0), 131 (18.9), 133 (10.8),135 (32.1), 136 (22.1), 137 (10.6),138 (9.4), 145 (11.0), 149 (20.6), 150 (28.6), 151 (8.4), 152 (6.2), 163 (9.1), 164 (15.4), 173 (7.7), 178 (22.1), 179 (5.1), 220 (11.8), 234 (5.8), 263 (8.0), 338 (17.4)	NIST, 946
309	RI2197 Amine	-	32.18	2196.8		174		-
310	α-Linolenic acid*	1TMS	32.22	2198.9	11.1	335	79 ((.9), 95 (76.0), 67 (67.8), 81 (60.5), 93 (59.5), 108 (52.5), 80 (43.2), 107 (38.7), 94 (37.9), 121 (34.5), 53 (5.9), 61 (7.9), 65 (5.3), 66 (6.7), 72 (5.4), 74 (14.0), 76 (10.0), 77	NIST, 911

							(20.1), 78 (14.6), 89 (5.1), 91 (33.9), 92 (8.2), 105 (21.5), 119 (22.3), 120 (8.1), 122 (23.1), 130 (10.5), 133 (22.7), 134 (10.7), 136 (16.9), 137 (10.3), 143 (8.6), 14 (6.8), 147 (13.0), 148 (6.6), 149 (23.8), 150 (10.5), 157 (5.7), 159 (8.7), 161 (6.6), 163 (9.9), 164 (5.7), 171 (7.5), 173 (10.3), 187 (5.0), 335 (18.9), 336 (5.1)	
311	*trans*-9-Octadecenoic acid*	1TMS	32.23	2199.2	5.2	339	117 (99.9), 129 (73.4), 339 (52.1), 96 (35.8), 84 (26.3), 98 (26.0), 69 (25.6), 97 (23.8), 93 (22.7), 132 (21.1), 54 (8.8), 56 (7.0), 57 (11.6), 70 (5.9), 82 (17.2), 99 (5.2), 110 (15.8), 111 (12.9), 112 (5.3), 116 (7.6), 124 (8.8), 138 (6.4), 151 (5.0), 152 (8.5), 180 (8.4), 222 (13.2), 264 (14.3), 354 (6.1)	GDM, NIST, 741
312	RI2203 Unknown	-	32.30	2203		140		-
313	Oleic acid*	1TMS	32.35	2205.5	10.5	339	117 (99.9), 339 (74.8), 129 (37.7), 97 (35.9), 73 (35.1), 145 (31.3), 81 (27.7), 96 (25.8), 55 (21.4), 74 (19.2), 56 (9.5), 67 (16.4), 68 (7.9), 69 (15.7), 76 (8.9), 82 (16.5), 83 (16.0), 85 (13.6), 94 (8.0), 95 (10.3), 98 (15.8), 99 (10.0), 108 (10.1), 110 (14.8), 111 (16.5), 113 (8.6), 115 (5.3), 118 (11.4), 121 (7.4), 142 (6.0), 143 (9.6), 144 (5.2), 146 (6.0), 151 (5.8), 152 (8.1), 166 (9.7), 172 (5.2), 184 (5.8), 185 (17.7), 199 (17.1), 203 (5.1), 217 (6.3), 222 (10.5), 241 (5.2), 257 (6.5), 258 (5.6), 264 (10.0), 286 (5.5), 295 (5.8), 331 (5.9), 340 (12.1), 341 (5.9), 342 (5.7), 354 (13.2)	NIST, 622
314	RI2110 Unknown	-	32.45	2210.1		132		-
315	RI2214 Carbohydrate	-	32.52	2214		204		-
316	RI2220 Unknown	-	32.63	2219.8		131		-
317	Stearic acid*	1TMS	32.70	2223.3	13.1	341	117 (99.9), 341 (79.3), 73 (61.7), 132 (52.6), 75 (45.5), 129 (38.7), 145 (31.4), 342 (21.2), 131 (15.5), 55 (15.2), 57 (9.6), 69 (10.0), 74 (5.9), 81 (6.2), 83 (6.7), 95 (6.3), 97 (5.7), 118 (9.8), 133 (11.2), 201 (10.0), 313 (5.7), 343 (5.7), 356 (10.0)	NIST, 930
318	RI2227 Carbohydrate	-	32.77	2226.9		204		-
319	RI2228 Unknown	-	32.79	2227.9		158		-
320	RI2229 Unknown	-	32.81	2228.9		219		-
321	RI2235 Unknown	-	32.94	2235.4		140		-
322	RI2239 Amine	-	33.01	2239.1		174		-

323	RI2245 Oligosaccharide derived acid	-	33.13	2245.2		204		-
324	RI2247 Amine	-	33.17	2247.1		174		-
325	RI2256 Unknown	-	33.35	2256.2		326		-
326	RI2270 Carbohydrate	-	33.61	2269.6		204		-
327	RI2273 Unknown	-	33.70	2273.5		204		-
328	RI2273 Unknown	-	33.74	2276		159		-
329	2-O-Glycerol-α-d-galactopyranoside*	6TMS	33.88	2283	103	204	204 (99.9), 73 (47.2), 337 (21.7), 217 (20.8), 147 (18.5), 205 (18.5), 103 (16.0), 129 (14.9), 206 (8.4), 219 (7.9), 338 (6.4), 361 (5.2)	GDM, NIST, 951
330	RI2311 Disaccharide	-	34.44	2311.5		204		-
331	RI2317 Unknown	-	34.55	2317.3		140		-
332	RI2320 Disaccharide	-	34.61	2320.2		204		-
333	Oleic acid amide (9-Octadecenamide)*	-	34.67	2323.2		59		NIST, 836
334	RI2332 Unknown	-	34.85	2332.2		415		-
335	RI2336 Sugar-derived acid	-	34.91	2335.5		217		-
336	Dehydroabietic acid*	1TMS	35.07	2343.7	36.3	239	239 (99.9), 73 (38.6), 240 (19.8), 372 (11.7),209 (10.9), 357 (10.6), 209 (10.4), 173 (9.8), 117 (9.4), 147 (8.8), 103 (7.3), 141 (5.9), 143 (6.7), 171 (8.8), 217 (8.7), 255 (6.2)	NIST, 862
337	Diethylhexyl adipate (DEHA)*	-	35.16	2348.4		129		NIST, 878
338	RI2356 Disaccharide	-	35.32	2356.1		361		-
339	Myo-Inositol phosphate 1	7TMS	35.43	2361.7	36.3	318	73 (99.9), 318 (85.5), 315 (51.0), 299 (42.7), 147 (38.9), 387 (26.2), 316 (16.7), 217 (16.6), 138 (16.2), 191 (15.8), 74 (9.2), 75 (11.7), 103 (7.7), 129 (10.0), 133 (8.3), 149 (6.3), 207 (6.7), 300 (11.0), 388 (6.7), 390 (6.5)	NIST, 902

340	Oleic acid amide*	1TMS	35.52	2366.7	60.3	338	131 (99.9), 144 (73.5), 116 (37.7), 338 (33.6), 128 (33.4), 198 (17.4), 353 (13.1), 115 (12.5), 184 (12.1), 158 (11.1), 50 (5.6), 52 (5.2), 61 (8.5), 69 (8.5), 84 (6.9), 100 (9.2), 145 (10.1), 221 (7.0), 254 (7.2), 339 (7.0), 430 (6.2)	NIST, 872
341	RI2368 Unknown	-	35.55	2368.1		157		-
342	1,8-dihydroxyanthraquinone*	2TMS	35.61	2370.8	181.2	369	183 (99.9), 369 (75.7), 93 (43.9), 133 (25.6), 370 (21.5), 73 (20.2), 69 (16.7), 129 (16.7), 107 (15.8), 171 (13.2), 61 (6.8), 71 (5.3), 78 (8.1), 105 (11.2), 119 (10.3), 112 (5.4), 121 (5.4), 127 (6.0), 221 (8.7), 223 (5.0), 259 (5.7), 263 (6.5), 267 (6.5), 367 (5.8)	NIST, 904
343	RI2374 Disaccharide	-	35.66	2373.6		204		-
344	RI2377 Amine	-	35.74	2377.5		174		-
345	RI2387 Unknown	-	35.91	2386.5		295		-
346	Uridine	3TMS	35.97	2389.4	24.9	217	217 (99.9), 73 (96.3), 259 (31.3), 103 (26.5), 218 (24.5), 147 (22.8), 169 (22.5), 243 (14.9), 75 (11.7), 219 (11.1), 74 (8.3), 129 (10.5), 148 (6.7), 191 (8.4), 213 (5.1), 231 (5.1), 245 (9.8), 257 (5.2), 315 (5.9)	NIST, 895
347	RI2391 Disaccharide	-	36.00	2391.1		204		-
348	Arachidic acid*	1TMS	36.03	2392.2	3.2	369	369 (99.9), 117 (66.8), 75 (60.4), 129 (57.2), 370 (37.6), 131 (37.0), 132 (31.4), 55 (25.6), 145 (23.3), 371 (23.0), 56 (9.3), 57 (15.7), 61 (6.8), 69 (7.9), 70 (8.3), 76 (9.3), 89 (5.6), 93 (5.5), 95 (9.4), 112 (5.5), 116 (10.0), 118 (8.1), 119 (5.9), 121 (6.1), 130 (9.6), 146 (6.0), 201 (6.6), 205 (6.0), 206 (5.0), 208 (9.8), 209 (7.7), 319 (5.0), 327 (7.9), 384 (5.5), 415 (5.4)	NIST, 710
349	*myo*-Inositol phosphate 2	7TMS	36.27	2404.8	74.2	318	318 (99.9), 87 (81.6), 299 (74.7), 315 (64.2), 387 (62.3), 243 (48.4), 388 (45.0), 71 (39.1), 319 (33.1),320 (32.5), 193 (23.6), 209 (30.7), 210 (23.3), 233 (21.6), 316 (22.0), 317 (28.4), 343 (16.8), 345 (25.2), 346 (18.8), 347 (14.6), 470 (15.4), 471 (13.6), 494 (13.3), 496 (13.5), 523 (7.1)	NIST, 602
350	RI2430 Unknown	-	36.77	2430		427		-
351	RI3433 Unknown	-	36.83	3433.1		136		-
352	RI2440 Disaccharide	-	36.97	2440.3		204		-
353	RI2443 Disaccharide	-	37.03	2443.2		361		-

354	RI2453 Disaccharide	-	37.23	2453.3		217		-
355	RI2472 Disaccharide	-	37.59	2471.6		219		-
356	RI2477 Disaccharide	-	37.69	2476.8		361		-
357	RI2481 Disaccharide	-	37.78	2481.5		361		-
358	RI2483 Unknown	-	37.82	2483.2		377		-
359	RI2504 Disaccharide	-	38.24	2504.5		361		-
360	1-Monopalmitoylglycerol*	1TMS	38.29	2507	74	371	371 (99.9), 372 (30.2), 147 (26.4), 73 (24.0), 239 (21.8), 129 (17.6), 203 (17.6), 57 (15.5), 71 (11.6), 95 (9.5), 55 (8.5), 69 (62), 75 (7.0), 83 (9.2), 85 (8.5), 95 (9.5), 97 (5.8), 101 (5.9), 109 (7.0), 117 (6.8), 123 (5.2), 131 (6.2), 133 (5.2), 145 (7.6), 205 (8.3), 459 (8.5)	NIST, 931
361	2-Hydroxysebacic acid*	2TMS	38.64	2525.2	466.2	317	317 (99.9), 73 (39.1), 318 (27.5), 109 (17.0), 129 (12.1), 155 (11.1), 319 (10.6), 147 (9.3), 75 (7.5), 391 (6.0)	NIST, 800
362	RI2529 Disaccharide	-	38.70	2529		361		-
363	RI2528 Unknown	-	38.70	2528.1		236		-
364	RI2532 Unknown	-	38.77	2531.8		173		-
365	Adenosine*	4TMS	38.94	2540.1	113.9	230	236 (99.9), 245 (71.5), 231 (33.6), 208 (24.9), 540 (23.3), 237 (20.3), 232 (18.1), 280 (17.8), 541 (9.6), 334 (8.3), 542 (6.0)	NIST, 641
366	Sucrose*	8TMS	38.95	2540.8 (2541)	69.9	361	361 (99.9), 73 (37.6), 362 (31.0), 217 (25.6), 437 (21.3), 363 (15.3), 271 (11.6), 147 (11.4), 169 (10.8), 103 (10.4), 129 (8.5), 218 (5.8), 438 (8.2),	ST, in-house, NIST, 954
367	Behenic acid (Docosanoic acid)*	1TMS	39.07	2547	37	397	117 (99.9), 73 (83.1), 397 (56.1), 217 (52.4), 132 (52.0), 129 (50.2), 145 (39.3), 171 (27.1), 147 (23.8), 55 (12.0), 58 (22.1), 59 (6.6), 69 (15.6), 71 (10.9), 75 (19.1), 82 (5.5), 95 (9.0), 97 (13.4), 98 (5.1), 111 (9.6), 116 (7.4), 118 (13.8), 123 (5.6), 128 (5.9), 131 (17.4), 133 (17.5), 159 (8.3), 170 (5.3), 172 (5.4), 177 (5.1), 183 (5.5), 191 (8.5), 193 (7.4), 201 (8.3), 202 (5.6), 205 (8.1), 219 (6.3), 231 (7.5), 233 (6.3), 239 (5.5), 143 (10.4), 245 (7.4), 272 (5.0), 273 (7.0), 282 (7.8), 291 (6.0), 295 (5.2), 306 (5.0), 320 (6.8), 353 (5.7), 360 (6.7), 398 (12.5), 412 (13.6), 436 (6.5), 518 (5.0)	NIST, 750

368	RI2561 Disaccharide	-	39.36	2561.3		217		-
369	Delta(9)-tetrahydrocannabinolic acid*	2TMS	39.42	2564.8	87.2	502	502 (99.9), 73 (52.1), 503 (38.7), 487 (27.3), 504 (17.6), 488 (11.1), 267 (6.6), 251 (5.7), 74 (5.6), 505 (5.5), 489 (5.4)	NIST, 707
370	RI2568 Disaccharide	-	39.50	2568.4		204		-
371	RI2579 Disaccharide	-	39.70	2579		361		-
372	RI2592 Disaccharide	-	39.97	2592.5		204		-
373	RI2600 Disaccharide	-	40.12	2599.8		204		-
374	RI2615 Disaccharide	-	40.42	2615		361		-
375	RI2619 Disaccharide	-	40.52	2619.4		361		-
376	RI2621 Unknown	-	40.53	2621		411		-
377	RI2628 Disaccharide	-	40.67	2628.2		204		-
378	2-Oleoylglycerol*	2TMS	40.70	2629.7	114.3	129	129 (99.9), 73 (80.0), 147 (41.8), 103 (37.0), 67 (27.4), 81 (25.0), 79 (21.5), 395 (20.9), 95 (19.1), 131 (19.0), 55 (16.0), 69 (11.0), 75 (10.4), 80 (9.8), 82 (6.5), 91 (6.1), 93 (11.1), 94 (5.8), 105 (6.7), 107 (7.6), 109 (10.6), 121 (9.6), 123 (8.9), 130 (14.3), 133 (13.4), 137 (5.3), 148 (5.9), 149 (10.8), 205 (5.4), 203 (16.7), 262 (10.5), 305 (11.1), 483 (6.8)	NIST, 755
379	1-Monooleoylglycerol*	2TMS	40.76	2632.9	151.1	129	129 (99.9), 397 (66.5), 73 (58.0), 147 (53.3), 103 (39.1), 55 (31.7), 203 (26.3), 69 (25.4), 201 (24.9), 83 (23.1), 57 (12.4), 67 (13.2), 71 (6.5), 75 (12.8), 81 (14.3), 95 (14.8), 96 (5.6), 97(15.3), 98 (7.7), 101 (11.7), 109 (9.9), 111 (9.4), 116 (11.3), 117 (12.2), 121 (7.7), 130 (17.6), 131 (14.5), 132 (5.5), 133 (10.8), 135 (9.4), 145 (7.2), 146 (5.8), 148 (9.5), 149 (11.5), 205 (11.5), 215 (5.8), 219 (5.8), 246 (5.8), 247 (5.9), 257 (21.3), 265 (14.2), 307 (14.4), 314 (5.8), 339 (5.4), 367 (5.0), 398(18.9), 399 (5.7), 410 (8.7), 485 (12.0), 486 (5.5)	NIST, 81.9
380	RI2635 Unknown	-	40.81	2635.1		204		-
381	RI2636 Lysolipid	-	40.82	2635.8		393		-

382	Guanosine*	5TMS	40.96	2642.8	159.2	324	73 (99.9), 324 (85.9), 245 (46.7), 280 (37.7), 368 (30.9), 230 (28.3), 325 (23.1), 103 (20.6), 147 (20.3), 296 (15.9), 74 (8.4), 75 (6.8), 115 (5.2), 169 (5.5), 217 (12.4), 231 (6.7), 243 (11.6), 246 (9.3), 259 (7.3), 281 (8.6), 295 (10.9), 326 (8.9), 369 (9.3)	GDM, NIST, 887
383	1-Monostearateglycerol*	2TMS	41.12	2651.2	114.8	399	399 (99.9), 400 (35.5), 73 (26.3), 203 (21.2), 55 (18.1), 75 (18.1), 71 (17.7), 85 (16.6), 145 (16.1), 267 (16.1), 51 (7.9), 52 (6.7), 57 (11.5), 59 (9.4), 69 (5.2), 72 (5.8), 83 (9.8), 95 (7.8), 97 (9.9), 101 (6.3), 103 (7.3), 111 (8.1), 116 (9.7), 118 (5.2), 131 (10.5), 146 (9.2), 149 (7.3), 189 (5.5), 204 (5.9), 205 (10.7), 245 (5.0), 295 (6.8), 341 (8.1), 401 (10.3), 487 (12.0)	NIST, 759
384	RI2656 Disaccharide	-	41.21	2655.8		204		-
385	RI2657 Disaccharide	-	41.23	2656.6		361		-
386	RI2663 Disaccharide	-	41.36	2663.2		204		-
387	RI2668 Disaccharide	-	41.46	2668.4		204		-
388	Trehalose*	8TMS	41.59	2675.5	140.5	361	361 (99.9), 204 (83.8), 79 (78.4), 129 (64.0), 217 (62.7), 205 (57.5), 97 (37.2), 82 (33.2), 317 (32.3), 218 (30.7), 268 (19.6), 270 (17.7), 274 (24.1), 315 (20.5), 328 (19.5), 358 (16.7), 395 (18.8), 501 (12.8)	in-house, NIST, 531
389	RI2680 Di- or oligosaccharide	-	41.69	2680.1		204		-
390	Lignoceric acid (Tetracosanoic acid)*	1TMS	41.94	2692.7	89.3	425	117 (99.9), 425 (83.9), 132 (59.4), 73 (57.8), 145 (44.3), 129 (41.8), 75 (40.5), 426 (28.7), 440 (24.8), 55 (18.5), 57 (18.3), 69 (15.1), 71 (8.9), 74 (6.2), 83 (10.4), 85 (5.5), 97 (10.1), 98 (7.0), 111 (6.9), 118 (9.9), 130 (5.5), 133 (17.9), 146 (6.2), 201 (15.4), 381 (6.5), 397 (7.6), 427 (6.7), 441 (9.6)	NIST, 828
391	RI2694 Di- or oligosaccharide	-	41.96	2694.7		204		-
392	RI2703 Disaccharide	-	42.14	2702.7		361		-
393	RI2733 Di- or oligosaccharide	-	42.72	2732.6		204	204(99.9), 73(39.8), 205(19.4), 217(15.5), 129(13.0), 147(10.3), 259(9.4), 175(9.3), 206(8.5), 103(6.7)	-
394	RI2745 Unknown	-	42.98	2745.5		219		-

395	RI2750 Di- or oligosaccharide	-	43.06	2749.7		204	204(99.9), 73(32,5), 205(18.1), 217(12.9), 259(11.7), 189(11.0), 129(10.3), 206(8.4), 147(8.2), 103(5.8)	-
396	RI2763 Di- or oligosaccharide	-	43.33	2763.2		204		-
397	RI2777 Di- or oligosaccharide	-	43.60	2777.2		204		-
398	RI2783 Di- or oligosaccharide	-	43.71	2782.5		204		-
399	RI2798 Di- or oligosaccharide	-	44.01	2797.8		204		-
400	RI2813 Di- or oligosaccharide	-	44.32	2813.4		204		-
401	RI2824 Di- or oligosaccharide	-	44.52	2823.8		204		-
402	Stigmastane*	-	44.54	2825	156	394	55(37.0), 67(29.0), 69(26.0), 79(33.9), 81(72.9), 83(38.8), 91(29.9), 92(6.0), 93(21.2), 94(14.1), 95(39.0), 105(70.6), 106(10.2), 107(30.2), 109(24.6), 111(5.3), 118(5.0), 119(22.1), 120(5.3), 121(14.8), 123(13.8), 131(20.6), 133(21.5), 135(16.8), 137(8.8), 143(10.0), 159(17.0), 161(12.1), 173(7.8), 199(7.5), 213(16.4), 228(18.5), 229(8.9), 239(10.6), 253(99.9), 254(28.5), 255(37.6), 256(9.2), 282(16.2), 351(10.2), 379 (20.3), 394(34.2), 395(11.4)	NIST, 647
403	Phosphoconjugated compound	-	44.63	2829.4		299	117(99.9), 453(75.4),132(52.9), 145(47.1), 133(31.3), 299(29.7), 468(29.2), 454(28.1), 57 (26.8), 74(17.6), 357(13.0), 257(12.9), 109(10.8), 111(13.9). 349(7.1), 211(5.9)	-
404	Hexacosanoic acid*	1TMS	44.64	2829.7	151.3	453	117 (99.9), 73 (93.7), 132 (61.8), 129 (61.6), 453 (61.5), 75 (52.1), 145 (46.4), 55 (32.3), 57 (31.4), 468 (26.9), 56 (6.9), 58 (16.8), 69 (19.1), 70 (6.0), 71 (8.5), 74 (16.0), 76 (6.7), 83 (17.4), 95 (11.8), 97 (8.7), 98 (8.0), 99 (7.6), 109 (6.7), 111 (14.0), 112 (6.2), 116 (5.9), 118 (10.0), 130 (6.3), 131 (13.8), 133 (19.7), 135 (8.2), 143 (9.3), 146 (10.0), 148 (15.4), 152 (5.4), 154 (7.0), 155 (7.6), 159 (8.6), 169 (7.3), 185 (10.6), 187 (9.4), 201 (24.6), 211 (5.0), 215 (6.3), 218 (7.0), 235 (5.7), 243 (7.9), 269 (5.7), 271 (5.3), 299(12.7), 301 (5.4), 313 (5.2), 315 (5.4), 341 (7.0), 342 (5.6), 356 (8.2), 358 (6.3), 371 (6.7), 415 (6.4), 425 (6.9), 455 (5.4), 468 (26.9), 469 (10.7)	NIST, 616

405	RI2837 Oligosaccharide	-	44.78	2836.9	204	204 (99.9), 207 (57.1), 147 (56.7), 217 (41.6), 435 (38.0), 208 (37.2), 191 (30.4), 133 (26.8), 319 (17.6), 509 (14.7), 99 (13.4), 146 (11.1), 220 (7.8), 291 (9.9), 408 (8.7), 436 (11.1), 510 (9.8)	-
406	RI2847 Unknown	-	44.97	2846.7	200	204 (99.9), 58 (60.8), 115 (47.2), 57 (46.9), 259 (39.5), 169 (37.5), 333 (29.6), 211 (29.1), 101 (26.2), 60 (22.9), 54 (17.6), 90 (17.1), 112 (21.9), 153 (16.3), 164 (11.5), 172 (10.2), 189 (20.6), 197 (19.5), 198 (10.8), 200 (12.8), 202 (19.1), 206 (20.8), 223 (16.3), 229 (16.1), 232 (14.0), 234 (12.4), 236 (9.7), 252 (16.0), 262 (12.9), 278 (10.1), 286 (9.9), 305 (17.1), 310 (10.4), 345 (11.7), 354 (9.6), 367 (7.9), 373 (15.0), 393 (16.9), 396 (16.8), 406 (10.9), 417 (10.2), 419 (11.5), 431 (14.8), 436 (16.4), 468 (10.7), 506 (10.8), 537 (10.0)	-
407	RI2864 Di- or oligosaccharide	-	45.31	2863.8	204	207 (99.9), 73 (91.1), 204 (88.9), 208 (30.3), 219 (27.8), 249 (15.1), 206 (14.0), 143 (12.9), 169 (12.7), 415 (12.4), 166 (7.9), 190 (8.1), 286 (9.0), 319 (11.3), 351 (9.1), 364 (6.9), 416 (9.9), 477 (6.2)	-
408	RI2872 Oligosaccharide	-	45.48	2872.3	204	204 (99.9), 217 (77.2), 361 (38.5), 305 (35.2), 124 (34.9), 167 (33.2), 116 (28.9), 369 (27.3), 308 (23.9), 377 (22.3), 367 (14.2), 407 (14.5), 476 (16.7)	-
409	RI2887 Oligosaccharide	-	45.78	2887.6	204	204(99.9), 73 (38.2), 217(27.6), 129 (22.4), 205(20.7), 103(18.3), 361(15.1), 147(13.4), 191(11.6), 337(11.0), 169(6.4), 191(11.6), 243(8.3), 271(6.1), 337(11.0), 361(15.1), 362(5.2)	-
410	RI2891 Unknown	-	45.84	2891	502	169 (99.9), 502 (91.6), 203 (71.1), 320 (43.1), 254 (37.7), 316 (32.9)	-
411	RI2898 Unknown	-	45.98	2898	467	281 (99.9), 467 (55.3), 259 (42.9), 91 (40.7), 243 (40.7), 268 (29.0), 194 (27.5), 239 (18.4), 459 (12.5)	-
412	RI2898 Di- or oligosaccharide	-	45.99	2898.3	361	73 (99.9), 259 (93.4), 361 (74.1), 147 (67.2), 103 (73.5), 129 (36.5), 355 (36.5), 221 (35.3), 254 (30.8), 281 (30.6), 66 (7.2), 87 (5.6), 96 (12.2), 104 (11.1), 112 (5.2), 121 (6.8), 142 (5.6), 151 (6.3), 157 (9.1), 167 (6.2), 170 (7.2), 185 (5.0), 191 (21.9), 192 (9.4), 218 (8.0), 229 (7.1), 230 (5.5), 232 (5.6), 239 (7.9), 243 (8.7), 255 (6.9), 260 (26.5), 261 (14.4), 268 (5.4), 272 (6.5), 273 (7.9), 284 (5.2), 307 (6.0), 327 (8.6), 356 (9.0), 357 (13.9), 362 (17.5), 363 (13.7), 429 (23.5), 430 (6.9), 431 (7.1), 437 (20.3), 438 (8.4), 450 (6.9), 468 (5.0), 504 (9.1), 505 (5.8)	-
413	RI2932 Di- or oligosaccharide	-	46.65	2931.8	361	103 (99.9), 361 (63.7), 147 (47.7), 284 (39.7), 362 (29.5), 191 (29.0), 169 (24.7), 260 (24.3), 260 (24.3), 437 (23.8), 261 (18.0), 74 (13.2), 76 (13.6), 104 (10.9), 105 (12.1), 121 (17.0), 125 (7.6), 164 (7.6), 170 (8.5), 173 (7.6), 179 (6.5), 183 (9.2), 189 (12.3), 193	-

							(9.7), 194 (5.6), 209 (15.1), 211 (16.3), 213 (5.0), 217 (17.5), 229 (7.7), 231 (9.7), 232 (7.5), 255 (8.7), 257 (11.1), 261 (18.0), 269 (6.7), 296 (6.0), 297 (7.7), 329 (5.5), 342 (7.2), 347 (8.5), 378 (5.8), 451 (5.2)	
414	RI2939 Unknown	-	46.78	2938.7		91		-
415	RI2962 Unknown	-	47.24	2961.9		481	117 (99.9), 131 (88.1), 57 (71.3), 109 (67.8), 132 (66.7), 133 (64.0), 281 (58.1), 481 (57.3), 217 (56.3), 189 (48.3), 61 (27.0), 74 (34.4), 85 (32.6), 106 (20.6), 108 (14.6), 112 (22.0), 116 (28.6), 118 (22.6), 124 (12.5), 126 (23.0), 136 (23.3), 142 (15.2), 148 (38.8), 150 (33.9), 151 (31.5), 154 (16.5), 156 (12.3), 157 (32.5), 162 (14.6), 167 (13.8), 175 (16.7), 179 (25.0), 201 (27.8), 211 (17.1), 213 (13.9), 215 (14.7), 228 (11.6), 231 (19.9), 232 (16.5), 247 (12.1), 255 (19.3), 260 (20.3), 261 (20.5), 264 (9.4), 266 (20.1), 269 (16.4), 301 (15.2), 309 (21.7), 311 (.9), 336 (9.1),364 (12.4), 366 (8.1), 376 (6.1), 377 (7.6), 398 (7.7), 401 (21.8), 416 (12.9), 419 (8.1), 427 (6.7), 429 (21.6), 432 (10.0), 439 (9.1), 443 (13.5), 445 (6.0), 453 (10.5), 455 (6.0), 453 (10.5), 455 (6.0), 460 (6.9), 472 (6.5), 475 (13.8), 478 (7.7), 482 (22.7), 486 (7.6), 488 (8.3), 497 (12.5), 547 (9.2), 549 (12.5)	-
416	Ergosta-7-en-3-beta-ol*	1TMS	48.28	3014.6	235.6	255	75 (99.9), 81 (96.1), 83 (95.4), 147 (73.8), 55 (71.9), 255 (71.8), 69 (64.4), 107 (62.3), 105 (59.9), 145 (50.6), 50 (6.7), 51 (8.9), 52 (9.0), 53 (7.8), 57 (31.7), 59 (12.0), 61 (7.4), 67 (33.5), 68 (6.0), 70 (9.1), 71 (17.5), 73 (35.6), 79 (20.9), 82 (13.5), 85 (16.6), 91 (41.1), 92 (9.9), 93 (47.5), 94 (12.4), 95 (35.7), 97 (17.2), 103 (5.5), 104 (6.2), 106 (7.3), 108 (7.9), 110 (6.3), 116 (5.9), 117 (11.0), 118 (6.3), 123(12.9), 127 (5.9), 131(42.4), 132 (7.1), 133 (47.9), 134 (17.7), 136 (39.3), 136 (7.1), 137 (22.1), 141 (6.5), 143 (8.6), 144 (8.1), 146 (18.1), 149 (16.5), 157 (18.6), 160 (10.3), 162 (5.3), 169 (13.4), 170 (5.4), 171 (15.2), 173 (12.1), 174 (14.8), 178 (12.7), 183 (8.5), 188 (5.1), 193 (10.9), 196 (6.2), 199(12.0), 201 (18.2), 205(5.2), 211 (12.4), 212 (6.0), 213 (29.8), 214 (8.2), 215 (14.8), 223 (5.4), 225 (7.4), 227 (12.1), 228 (21.2), 229 (20.3), 230 (8.0), 239 (8.8), 240 (5.5), 241 (13.5), 243 (8.4), 253 (27.3), 256 (22.2), 257 (6.8), 267 (10.8), 269 (6.0), 271 (5.4), 295 (8.2), 301 (5.5), 309 (9.6), 318 (19.1), 329 (9.8), 330 (7.1), 341 (7.0), 344 (32.0), 345 (27.0), 346 (9.3), 351 (21.0), 355 (7.6), 358 (7.6), 372 (12.7), 379 (11.8), 469 (18.3), 484 (34.9)	NIST, 605
417	Stigmasterol*	1TMS	48.65	3033.5	228.5	343	343 (99.9), 255 (71.3), 81 (61.7), 83 (60.7), 75 (47.8), 55 (44.3), 107 (41.6), 105 (36.3), 344 (35.1), 253 (34.7), 57 (14.0), 67 (18.1), 69 (33.7), 71 (7.1), 73 (29.8), 79 (18.5), 82 (6.0), 85 (6.8), 91 (25.0), 93 (24.7), 94 (9.2), 95 (32.5), 97 (12.3), 106 (5.6), 108 97.3), 109 (24.1), 117 (11.7), 119 (25.6), 120 (7.4), 121 (21.6), 122 (5.0), 123 (12.5), 129 (14.6), 131 (21.6), 132 (6.6), 133 (30.9), 134 (10.3), 135 (21.3), 137 (11.2), 143 (11.6),	NIST, 799

							144 (7.4), 145 (30.9), 146 (10.7), 147 (31.7), 149 (10.4), 157 (12.3), 159 (27.3), 160 (6.5), 161 (17.1), 163 (5.1), 164 (13.5), 169 (6.4), 171 (9.4), 173 (14.6), 175 (8.1), 178 (7.2), 185 (9.3), 187 (11.1), 199 (9.8), 201 (10.2), 211 (7.4), 213 (23.8), 214 (5.3), 215 (9.6),227 (10.0), 228 (12.6), 229 (25.7), 239 (6.3), 241 (8.6), 253 (34.7), 254 (9.5), 256 (14.2), 281 (5.8), 282 (5.0), 318 (14.2), 329 (8.8), 344 (35.1), 345 (26.0), 346 (5.9), 351 (13.9), 357 (5.3), 371 (7.6), 357 (5.3), 371 (7.6), 372 (14.0), 379 (12.6), 394 (8.9), 441 (7.1), 469 (20.5), 470 (7.8), 484 (33.6), 485 (13.2)	
418	RI3089 Oligosaccharide	-	49.60	3089.1		361	361(99.9), 73(46.7), 217(31.3), 204(29.3), 437(26.1), 362(26.0), 169(17.2), 363(17.2), 129(14.1), 438(13.5), 451(12.2), 452(9.1), 218(8.6), 364(5.7)	-
419	β-Sitosterol*	1TMS	49.71	3087.5	196.8	486	75 (99.9), 255 (67.4), 213 (61.7), 95 (52.6), 145 (42.9), 107 (42.7), 133 (40.6), 119 (38.7), 91 (34.8), 105 (34.6), 51 (6.6), 55 (16.1), 56 (10.1), 57 (9.7), 58 (12.0), 61 (11.9), 63 (5.0), 66 (8.7), 67 (15.3), 69 (19.8), 71 (8.0), 72 (7.0), 79 (20.7), 81 (33.7), 82 (6.4), 84 (10.7), 85 (9.1), 92 (11.7), 99 (7.3), 106 (11.9), 108 (14.0), 109 (15.0), 112 (6.3), 116 (6.0), 120 (14.9), 121 (21.8), 123 (8.0), 124 (8.7), 132 (11.0), 134 (16.5), 135 (28.4), 136 (9.3), 137 (20.7), 139 (5.8), 141 (7.2), 143 (12.4), 146 (18.4), 148 (33.3), 158 (5.9), 162 (5.2), 173 (16.8), 174 (5.4), 175 (10.7), 178 (5.5), 179 (8.1), 181 (7.0), 183 (5.5), 187 (7.3), 196 (6.6), 199 (7.9), 201 (15.1), 203 (9.2), 207 (23.3), 214 (11.4), 215 (5.2), 251 (6.8), 253 (8.5), 256 (17.4), 268 (5.5), 281 (10.7), 282 (13.1), 283 (10.9), 288 (8.5), 290 (8.3), 291 (5.0), 295 (12.6), 296 (8.5), 290 (8.3), 291 (5.0), 296 (12.6), 296 (8.1), 303 (9.6), 304 (7.7), 318 (5.0), 319 (5.2), 325 (5.8), 343 (10.4), 35 (20.6), 354 (5.8), 355 (21.5), 356 (14.5), 357 (7.8), 382 (12.0), 396 (15.3), 429 (25.4), 430 (12.5), 431 (9.8), 471 (25.0), 488 (18.5), 489 (5.4), 503 (8.2)	NIST, 536

[a] – Annotation of metabolite features relied on retention indices (RI), retention times (t_R) and in-house spectral library of authentic standards as well as on RI and spectral information of NIST 08 (National Institute of Standards and Technology) and GDM (Golm Metabolome Database). Unidentified metabolites are designated as "Unknown" with associated retention index (RI). The names of metabolites/features annotated only to a certain chemical class (for which the exact the exact structure could not be assigned) begin with the RI, followed by the name of the annotated chemical class. The annotation to specific chemical classes was confirmed by the presence of characteristic signals in the EI spectra (for example, saccharide-phosphate - *m/z* 299, 315, 387; disaccharide - *m/z* 361, 204, 319; saccharide deriv. acid - *m/z* 292, 333, 103). The metabolites/features are listed in the order of increasing retention times (t_R). Arabic numbers in the names of the features annotated to the same structures indicate the order of their elution. Bold font highlights metabolites which were identified by co-elution with

corresponding standards; *marks metabolites which are represented by only one feature; [b]numbers of the TMS and MEOX groups in the structures of annotated derivatives. [c] Retention time, min. [d] RI, Kovach retention index calculated from the retention time of alkanes, (RI of authentic standards). [e] RI difference between the NIST library RI entries and observed RI values. [f] The *m/z* value refers to the most compound-characteristic fragment signal selected for quantification by peak areas in characteristic extracted ion chromatograms. fragment information extracted from the EI (electron ionization) of individual analytes and presented as *m/z* (% relative intensity). [h] Libraries of metabolite EI mass spectra and RI used for the feature identification: in house – in-house RI and spectral library of authentic standards (ST marks metabolites which were identified by coelution with corresponding authentic standards), GDM – Golm Metabolome Database (http://gmd.mpimp-golm.mpg.de/) and NIST 08 – National Institute of Standards and Technology. Number presents NIST search match factor.

Table S1(2). Individual metabolites identified as Zn^{2+}-dependently regulated in young leaves of *A. caudatus*

#	Metabolite feature[a]	Derivatization[b]	RI[c]	*m/z*[d]	FC[e]	*p*[f]
	Metabolites increasing their abundance in response to Zn^{2+} *treatment*[g]					
1	Glyoxylic acid	1MEOX, 1TMS	1142.6	218	22	0.015
2	**3-Hydroxy-3-methylglutaric acid**	3TMS	1598.9	247	3.6	0.002
3	**Gluconic acid δ-lactone**	4TMS	1878.5	319	2.8	0.008
4	Ribonic acid-14-lactone	3TMS	1645.9	117	6.2	0.018
5	Lyxonic acid-1,4-lactone	3TMS	1729.7	217	1.8	0.017
6	Arabinonic acid-1,4-lactone	3TMS	1622.2	217	2	0.005
7	**Gluconic acid**	6TMS	1990.7	333	10	0.003
8	**RI1889 Sugar-derived acid**	-	1888.9	217	5.4	**≤0.001 / 0.019**
9	RI1797 Sugar-derived acid	-	1797.4	292	2.9	0.011
10	**RI1974 Sugar-derived acid**	-	1970.6	217	2.8	0.023
11	RI1984 Sugar_derived acid	-	1983.9	333	2.6	0.046
12	**Shikimic acid**	4TMS	1808.5	204	2.5	0.041
13	**Salicylic acid**	2TMS	1504.2	267	23	**≤0.001/ 0.025**
14	Octanoic acid	1TMS	1269.1	117	>100	≤0.001
15	Stearic acid	1TMS	2223.3	341	3	0.001
16	Arachidic acid	1TMS	2392.2	369	2.8	**≤0.001 / 0.005**
17	Myristic acid	1TMS	1846.1	285	2.6	0.02
18	Oleic acid	1TMS	2205.5	339	2	0.034
19	Heptadecanoic acid	1TMS	2134.3	327	2.1	0.002
20	**Behenic acid (Docosanoic acid)**	1TMS	2547	397	2	0.009
21	1-Monostearateglycerol	2TMS	2651.2	399	2.2	0.036
22	**Ethanolamine**	3TMS	1232.1	174	4.2	0.038
23	**N-acetyl-serine**	2TMS	1503.1	116	12	0.002
24	**Pyroglutamic acid**	1TMS	1496.4	84	5.5	0.006
25	**5-Methylcytosine**	2TMS	1534	254	7.4	0.023
26	**RI1764 C5-Carbohydrate**	-	1764.5	217	2.1	0.007
27	**Fructofuranose 1**	5TMS	1807	217	4.3	0.013
28	**Fructose 2**	1MEOX, 5TMS	1942.9	217	4.3	0.021
29	Fructofuranose 2	5TMS	1798.7	217	3.9	0.011
30	**Fructose 1**	1MEOX, 5TMS	1934.8	217	3.8	0.024
31	**Glucose 1**	1MEOX, 5TMS	1990.5	319	3.7	0.019
32	**Glucose 2**	1MEOX, 5TMS	2007.2	319	2.6	0.033
33	**Mannose**	1MEOX, 5TMS	1984.3	319	3.5	0.028
34	**Galactose**	1MEOX, 5TMS	1979.6	319	2.1	0.042
35	**Myo-inositol**	6TMS	2076.9	305	1.9	0.044
36	RI1896 C5-or C6-Carbohydrate	-	1896.2	204	1.5	0.025
37	**RI1673 Carbohydrate**	-	1673.1	217	1.5	0.044
38	RI1975 Carbohydrate	-	1975.3	204	2.3	0.005
39	RI1915 C6-Carbohydrate	-	1915.4	275	7.5	0.002
40	**RI2035 Carbohydrate**	-	2035.3	361	3.4	0.031
41	**RI1891 Carbohydrate**	-	1891	204	2.6	0.028

42	**RI1808 C6 Carbohydrate**	-	1807.9	437	4.5	0.026
43	**RI1949 Carbohydrate**	-	1949.3	361	5.5	0.02
44	Sucrose	8TMS	2540.8	361	6.6	0.043
45	**RI2600 Disaccharide**	-	2599.8	204	9.1	0.022
46	**RI2615 Disaccharide**	-	2615	361	7.3	0.003
47	RI2579 Disaccharide	-	2579	361	4.3	0.003
48	RI2481 Disaccharide	-	2481.5	361	2.8	0.041
49	RI2694 Di- or oligosaccharide	-	2694.7	204	2.7	0.018
50	RI2932 Di- or oligosaccharide	-	2931.8	361	3.1	0.005
51	**RI3089 Oligosaccharide**	-	3089.1	361	6.7	0.016
52	RI2887 Oligosaccharide	-	2887.6	204	2.3	**≤0.001 / 0.018**
53	RI2777 Di- or oligosaccharide	-	2777.2	204	1.6	0.033
54	Phosphoric acid monomethyl ester	2TMS	1185.5	241	1.7	0.036
55	**RI2191 Unknown phosphate**	-	2191.1	299	1.7	0.005
56	**RI1573 Unknown**	-	1572.5	110	36	≤0.001
57	**RI2483 Unknown**	-	2483.2	377	12.5	0.012
58	**RI2256 Unknown**	-	2256.2	326	6.1	0.018
59	RI2027 Unknown	-	2026.5	294	3.8	0.019
60	**RI2939 Unknown**	-	2938.7	91	4.7	0.009
61	**RI1859 Unknown**	-	1858.8	245	3.5	0.009
62	**RI1813 Unknown**	-	1813	214	3.4	0.009
63	RI2157 Unknown	-	2157.7	140	3.4	0.026
64	RI1886 Unknown	-	1886.4	204	3.2	0.003
65	RI2621 Unknown	-	2621	411	3.1	0.005
66	RI1643 Unknown	-	1643.3	226	3.1	0.005
67	**RI2001 Unknown**	-	2000.8	105	2.8	0.006
68	RI2430 Unknown	-	2430	427	2.7	0.005
69	RI2108 Unknown	-	2107.9	327	2.6	0.004
70	**RI1629 Unknown**	-	1629.1	246	2.6	0.034
71	**RI2368 Unknown**	-	2368.1	157	2.5	0.028
72	RI1461 Unknown	-	1461.3	255	2.4	0.009
73	RI1448 Unknown	-	1447.5	132	2.4	0.032
74	**RI1024 Unknown**	-	1024.5	158	2.4	0.018
75	**RI1881 Unknown**	-	1881.4	217	2.3	0.008
76	RI2146 Unknown	-	2145.6	369	2.3	0.003
77	RI2273 Unknown	-	2276	159	2.3	0.034
78	RI2891 Unknown	-	2891	502	2.2	0.008
79	**RI1596 Unknown**	-	1595.9	342	1.9	0.002
80	RI1731 Unknown	-	1731.4	215	1.7	0.03
81	RI2387 Unknown	-	2386.5	295	1.6	0.014
82	RI1921 Unknown	-	1921	300	1.6	0.029
83	**RI1436 Unknown**	-	1435.7	243	1.5	0.004
	Metabolites decreasing their abundance in response to Zn^{2+} treatment[h]					
1	**Succinic acid**	2TMS	1316.6	247	2.0	0.012
2	Linoleic acid	1TMS	2192.7	337	1.7	0.003
3	2-Oleoylglycerol	2TMS	2629.7	129	1.7	0.012
4	RI2657 Disaccharide	-	2656.6	361	1.7	0.049
5	**RI2477 Disaccharide**	-	2476.8	361	2.5	0.002

6	**Glycerol-3-phosphate**	4TMS	1758.8	299	1.7	0.017
7	RI1554 Unknown	-	1554	188	2.0	0.032
8	RI1543 Unknown	-	1542.9	188	2.0	0.035
9	**RI1528 Unknown**	-	1528.7	217	2.0	0.011
10	**RI1785 Unknown**	-	1784.9	312	3.3	0.032

Trimethylsilyl (TMS) and methyloxime (MEOX) derivatives of polar metabolites (i.e. individual features) from dry methanolic extracts of *A. caudatus* young leaves were annotated by untargeted GC-EI-Q-MS. The *A. caudatus* plants were grown in hydroponic nutrient solution with or without (controls) supplementation of 300 µmol/L Zn^{2+} under controlled conditions (24/18 °C day/night, relative humidity of 70–75%) and 16:8 light-darkness regimen. Young leaves were harvested from seven-week-old plants (n = 3). [a] Annotation of metabolite features relied on retention index (RI), retention time (t_R), on co-elution with authentic standards and spectral similarity in a search against the in-house spectral library as well as on RI and spectral information of NIST 08, National Institute of Standards and Technology) and GMD (Golm Metabolome Database) (Supplementary information 1, Table S1(1)). Metabolites are arranged by the following chemical classes: organic acids, fatty acids, lysolipids, N-metabolites, monosaccharides, polyols, disaccharides, organic phosphates.The numbers in the names of the features representing the same metabolite indicate the order of their elution. The names of the metabolites annotated to chemical classes without assignment of exact chemical structure were built by the combination of the metabolite RI and the name of the chemical class. The annotation to specific chemical class was confirmed by corresponding class-specific characteristic signals (for example, *m/z* 299, 315 and 387 for saccharide-phosphates, *m/z* 361, 204 and 319 for disaccharides, *m/z* 292, 333 and 103 for sugar acids). Unidentified metabolites are designated as "Unknown" specified with their retention indices (RI). Bold fond marks metabolites which were also Zn-regulated in roots. [b] The patterns of trimethylsilyl (abbreviated as TMS) and methyloxime (MEOX) substitutions. [c]RI, Kovach retention index calculated from the retention times of alkane elution standards. [d] The MS signal (*m/z* value) refering to the most compound-characteristic fragment ion selected for quantification by integration of peak areas in characteristic extracted ion chromatograms. [e]FC, fold change (at least 1.5-fold) in metabolite relative abundances in Zn-treated young leaves compared with controls. [f]t-test p-value ≤ 0.05. Bold font and symbol "/" marks p-values calculated without and with false discovery rate (FDR) correction by Benjamini-Hochberg method and did not exceed the p-value threshold 0.05 after application of the FDR correction. [g]metabolites ≥1.5-fold increasing their abundance in young leaves upon treatment with Zn^{2+} in comparison to the controls and [h] metabolites ≥1.5-fold decreasing their abundance in young leaves upon treatment with Zn^{2+} in comparison to the controlscontrols.

Table S1(3). Individual metabolites identified as Zn^{2+}-dependently regulated in the roots of *A. caudatus*.

#	Metabolite feature[a]	Derivatization[b]	RI[c]	*m/z*[d]	FC[e]	*p*[f]
	Metabolites increasing their abundance in response to Zn^{2+} treatment[g]					
1	Malonic acid	2TMS	1211.3	233	2.1	0.0019
2	Glyceric acid	3TMS	1330.4	292	2.5	**≤0.001 / 0.004**
3	Citric acid	4TMS	1814.1	273	2.7	**0.0035 / 0.01**
4	Citramalic acid	3TMS	1468.2	247	1.8	**0.002 / 0.01**
5	3,4-Dihydroxybutyric acid γ-lactone	-	1371.4	247	3.7	**≤0.001 / 0.007**
6	Adipic acid	2TMS	1504.9	111	1.9	0.023
7	Threonic acid	4TMS	1558.5	292	3.8	**0.001 / 0.007**
8	Erythronic acid	4TMS	1540.9	292	2.0	**≤0.001 / 0.003**
9	α-Hydroxyglutaric acid	3TMS	1573.8	247	2.4	**0.003 / 0.014**
10	**3-Hydroxy-3-methylglutaric acid**	3TMS	1598.9	247	2.4	**≤0.001 / 0.007**
11	**Gluconic acid**	6TMS	1990.7	333	59	**≤0.001 / 0.003**
12	**Gluconic acid δ-lactone**	4TMS	1878.5	319	25	**≤0.001 / 0.007**
13	RI1630 Xylonic acid-1,4-lactone	3TMS	1629.5	117	2.8	**≤0.001 / 0.005**
14	RI1776 C5-Sugar-derived acid	-	1776.0	292	3.3	**≤0.001 / 0.002**
15	RI1988 Sugar-derived acid	-	1988.4	292	69	**≤0.001 / 0.004**
16	RI1957 Sugar-derived acid	-	1956.7	333	48	**≤0.001 / 0.003**
17	**RI1974 Sugar-derived acid**	-	1970.6	217	30	**0.001 / 0.008**
18	**RI1889 Sugar-derived acid**	-	1888.9	217	30	**≤0.001 / 0.007**
19	RI1970 Sugar-derived acid	-	1969.7	217	22	**≤0.001 / 0.006**
20	RI2006 Sugar-derived acid	-	2005.7	333	4.3	**0.002 / 0.009**
21	RI2034 Sugar-derived acid	-	2033.8	333	2.3	**0.009 / 0.03**
22	**Shikimic acid**	4TMS	1808.5	204	2.7	**≤0.001 / 0.005**
23	**Salicylic acid**	2TMS	1504.2	267	27	**≤0.001 / ≤0.001**
24	2-Hydroxysebacic acid	2TMS	2525.2	317	5.2	**≤0.001 / 0.005**
25	**Behenic acid**	1TMS	2547.0	397	1.7	**0.006 / 0.02**
26	1-Monooleoylglycerol	2TMS	2632.9	129	1.7	**0.006 / 0.02**
27	Oleic acid amide	1TMS	2366.7	338	9.6	0.013
28	RI2239 Amine	-	2239.1	174	4.4	0.049
29	Proline [+CO_2]	2TMS	1577.6	142	6.8	≤0.001
30	Alanine	3TMS	1357.0	188	2.9	0.04
31	Leucine	2TMS	1271.7	158	2.0	0.042
32	N,N-Dimethylglycine	1TMS	1040.4	58	1.9	**0.004 / 0.02**
33	**N-acetyl-serine**	2TMS	1503.1	116	1.5	**0.012 / 0.035**
34	**5-Methylcytosine**	2TMS	1534.0	254	3.7	**≤0.001 / ≤0.001**
35	Uridine	3TMS	2389.4	217	2.3	**0.005 / 0.02**
36	Adenine - derivate	2TMS	2096.2	264	1.8	0.0018
37	Arabinose 1	1MEOX, 4TMS	1755.0	307	2.3	≤0.001
38	Arabinose 2	1MEOX, 4TMS	1758.9	307	2.2	**0.005 / 0.02**
39	Arabino-Hexos-2-ulose (2-Ketoglucose)	4TMS	1477.2	234	2.3	**0.02 / 0.044**
40	**Fructofuranose**	5TMS	1807.0	217	2.8	**≤0.001 / 0.005**
41	**Fructose 1**	1MEOX, 5TMS	1934.8	217	2.8	**≤0.001 / 0.004**
42	**Fructose 2**	1MEOX, 5TMS	1942.9	217	2.7	**≤0.001 / 0.006**

43	**Glucose 1**	1MEOX, 5TMS	1990.5	319	4.0	**≤0.001 / 0.002**
44	**Glucose 2**	1MEOX, 5TMS	2007.2	319	2.0	**≤0.001 / 0.006**
45	**Mannose**	1MEOX, 5TMS	1984.3	319	3.0	**≤0.001 / ≤0.001**
46	**Galactose**	1MEOX, 5TMS	1979.6	319	9.0	**≤0.001 / ≤0.001**
47	**Myo-inositol**	6TMS	1979.6	319	2.0	**0.001 / 0.008**
48	Ribitol	5TMS	1720.7	217	2.8	**≤0.001 / 0.003**
49	RI1929 Sugar alcohol	-	1928.8	319	2.1	**0.001 / 0.007**
50	RI1930 C5-or C6-Carbohydrate	-	1930.1	204	2.6	**0.005 / 0.02**
51	**RI1808 C6 Carbohydrate**	-	1807.9	437	2.9	**≤0.001 / 0.004**
52	**RI1949 Carbohydrate**	-	1949.3	361	3.1	**0.002 / 0.009**
53	**RI1891 Carbohydrate**	-	1891.0	204	4.4	**0.005 / 0.02**
54	**RI2035 Carbohydrate**	-	2035.3	361	1.8	**0.003 / 0.01**
55	2-O-Glycerol-α-d-galactopyranoside	6TMS	2283.0	204	2.2	**0.001 / 0.008**
56	RI2703 Disaccharide	-	2702.7	361	3.7	**0.002 / 0.009**
57	Trehalose	8TMS	2675.5	361	2.7	**≤0.001 / ≤0.001**
58	RI2561 Disaccharide	-	2561.3	217	3.4	**0.001 / 0.008**
59	**RI2615 Disaccharide**	-	2615.0	361	1.9	**0.007 / 0.02**
60	RI2592 Disaccharide	-	2592.5	204	1.7	0.031
61	**RI2600 Disaccharide**	-	2599.8	204	1.7	**0.006 / 0.02**
62	RI2798 Di- or oligosaccharide	-	2797.8	204	3.4	0.039
63	RI2864 Di- or oligosaccharide	-	2863.8	204	6.8	**≤0.001 / 0.007**
64	RI2750 Di- or oligosaccharide	-	2749.7	204	1.8	0.028
65	RI1141 Unknown	-	1141.0	220	5	0.005
66	RI1559 Unknown	-	1559.6	241	>100	≤0.001
67	**RI2483 Unknown**	-	2483.2	377	41	**0.0015 / 0.008**
68	**RI1881 Unknown**	-	1881.4	217	3.6	**0.002 / 0.01**
69	**RI2256 Unknown**	-	2256.2	326	3.6	0.0015
70	**RI1629 Unknown**	-	1629.1	246	3.6	**≤0.001 / ≤0.001**
71	**RI2001 Unknown**	-	2000.8	105	3.4	**0.003 / 0.01**
72	RI1250 Unknown	-	1250.1	133	3.1	**0.01 / 0.04**
73	RI1833 Unknown	-	1833.3	349	2.9	**0.009 / 0.03**
74	RI1555 Unknown	-	1555.3	140	2.8	**0.007 / 0.02**
75	RI2532 Unknown	-	2531.8	173	2.8	**0.01 / 0.03**
76	**RI1573 Unknown**	-	1572.5	110	2.6	**≤0.001 / 0.002**
77	**RI2939 Unknown**	-	2938.7	91	2.3	**0.0055 / 0.02**
78	**RI1859 Unknown**	-	1858.8	245	2.3	**≤0.001 / 0.001**
79	**RI1024 Unknown**	-	1024.5	158	2.1	**0.002 / 0.009**
80	RI1995 Unknown	-	1997.5	293	2.1	**0.001 / 0.007**
81	**RI1785 Unknown**	-	1784.9	312	2.1	**0.006 / 0.02**
82	RI1400 unknown	-	1399.8	239	2	**≤0.001 / ≤0.001**
83	RI1613 Unknown	-	1612.7	144	1.9	**0.001 / 0.008**
84	**RI2368 Unknown**	-	2368.1	157	1.9	0.033
85	RI1839 Unknown	-	1839.0	261	1.8	0.02
86	RI1538 Unknown	-	1538.7	132	1.7	≤0.001
87	RI1242 Unknown	-	1242.1	116	1.7	0.023
88	**RI1813 Unknown**	-	1813.0	214	1.6	**≤0.001 / 0.007**

89	RI1862 Unknown	-	1862.0	233	1.5	**0.013 / 0.035**
	Metabolites decreasing their abundance in response to Zn^{2+} treatment[h]					
1	Oxalic acid	2TMS	1145.5	190	1.60	**0.004 / 0.015**
2	**Succinic acid**	2TMS	1316.6	247	1.54	**≤0.001 / 0.006**
3	Methylmaleic acid	2TMS	1348.9	259	2.32	**0.02 / 0.05**
4	RI1401 Carboxylic acid	-	1400.9	184	3.41	0.028
5	RI1870 Sugar-derived acid	-	1870.7	292	2.01	**0.001 / 0.008**
6	*trans*-Caffeic acid	3TMS	2137.6	219	3.99	**0.0075 / 0.02**
7	Itaconic acid	2TMS	1342.9	183	2.36	**0.012 / 0.035**
8	**Ethanolamine**	3TMS	1232.1	174	2.79	**0.007 / 0.023**
9	RI1265 Amine	-	1265.3	174	1.94	0.003
10	**Pyroglutamic acid**	1TMS	1496.4	84	2.17	0.024
11	Oleic acid amide (9-Octadecenamide)	-	2323.2	59	1.55	**0.02 / 0.048**
12	RI1140 Unknown (Alanine-derivate)	-	1139.4	116	1.69	**0.006 / 0.022**
13	RI2066 Carbohydrate	-	2066.1	204	2.99	0.031
14	RI1952 Carbohydrate	-	1952.4	319	1.57	**0.013 / 0.035**
15	**RI1673 Carbohydrate**	-	1673.1	217	1.77	**0.012 / 0.035**
16	**RI1764 C5-Carbohydrate**	-	1764.5	217	2.09	**≤0.001 / ≤0.001**
17	RI2356 Disaccharide	-	2356.1	361	1.85	**≤0.001 / ≤0.001**
18	**RI2477 Disaccharide**	-	2476.8	361	1.58	**0.007 / 0.023**
19	RI2529 Disaccharide	-	2529.0	361	2.47	**0.003 / 0.013**
20	RI2504 Disaccharide	-	2504.5	361	4.67	**0.011 / 0.034**
21	RI2680 Di- or oligosaccharide	-	2680.1	204	1.70	0.039
22	**RI3089 Oligosaccharide**	-	3089.1	361	1.88	**0.007 / 0.023**
23	RI2783 Di- or oligosaccharide	-	2782.5	204	3.12	0.03
24	**Glycerol-3-phosphate**	4TMS	1758.8	299	2.16	**≤0.001 / 0.002**
25	Glycerophosphoglycerol	5TMS	2181.2	357	5.08	**≤0.001 / ≤0.001**
26	*myo*-Inositol phosphate	7TMS	2361.7	318	1.79	**0.001 / 0.008**
27	**RI2191 Unknown hosphate**	-	2191.1	299	1.65	**≤0.001 / 0.002**
28	Phosphoconjugated compound	-	2829.4	299	2.41	**0.0015 / 0.008**
29	Phytol	1TMS	2159.7	143	2.34	**0.008 / 0.03**
30	β-Sitosterol	1TMS	3087.5	486	2.97	**0.003 / 0.012**
31	RI1733 Unknown	-	1733.0	186	1.53	**0.003 / 0.01**
32	**RI1436 Unknown**	-	1435.7	243	1.57	0.0017
33	RI1592 Unknown	-	1591.9	262	1.63	**≤0.001/ 0.005**
34	RI1583 Unknown	-	1582.7	167	1.63	**0.006 / 0.022**
35	RI2110 Unknown	-	2210.1	132	1.68	0.036
36	RI1767 Unknown	-	1767.0	332	1.68	**0.001 / 0.008**
37	RI1469 Unknown	-	1469.2	359	2.01	**0.0055 / 0.02**
38	RI1572 Unknown	-	1572.3	117	2.02	**0.0015 / 0.008**
39	RI1453 Unknown	-	1453.5	98	2.06	≤0.001
40	**RI1528 Unknown**	-	1528.7	217	2.08	**0.01 / 0.03**
41	RI1678 Unknown	-	1678.6	141	2.11	0.025
42	RI1502 Unknown	-	1501.8	280	2.21	**0.015 / 0.041**
43	**RI1596 Unknown**	-	1595.9	342	2.37	**≤0.001 / ≤0.001**
44	RI1367 Unknown	-	1367.3	133	2.65	**≤0.001 / 0.005**

45	RI1461 Unknown	-	1461.3	255	2.71	**0.005 / 0.02**
46	RI2105 Unknown	-	2105.4	210	3.31	0.019
47	RI1778 Unknown	-	1776.7	253	>100	≤0.001
48	RI1744 Unknown	-	1744.1	215	>100	≤0.001

Trimethylsilyl (TMS) and methyloxime (MEOX) derivatives of polar metabolites (i.e. individual features) from dry methanolic extracts of *A. caudatus* roots were annotated by untargeted GC-EI-Q-MS. The *A. caudatus* plants were grown in hydroponic nutrient solution with or without (controls) supplementation of 300 µmol/L Zn^{2+} under controlled conditions (24/18 °C day/night, relative humidity of 70–75%) and 16:8 h light-darkness regimen. Roots were harvested from seven-week-old plants (n = 3, each replicate was the pool of three samples). [a] Annotation of metabolite features relied on retention index (RI), retention time (t_R), on co-elution with authentic standards and spectral similarity search against the in-house spectral library or against NIST 08 (National Institute of Standards and Technology) and GMD (Golm Metabolome Database) libraries (Supplementary information 1, Table S1(1)). Metabolites are arranged by the following chemical classes: organic acids, fatty acids, lysolipids, N-metabolites, monosaccharides, polyols, disaccharides, organic phosphates. Numbers in the names of the features representing the same metabolite indicate the order of their elution. The names of the metabolites annotated to certain chemical classes without assignment of exact chemical structure were built by the combination of the metabolite RI and the name of the chemical class. The annotation to specific chemical class was confirmed by corresponding class-specific characteristic signals (for example, *m/z* 299, 315 and 387 for saccharide-phosphates, *m/z* 361, 204 and 319 for disaccharides, *m/z* 292, 333 and 103 for sugar acids). Unidentified metabolites were designated as "unknowns" and are specified with their retention indices (RI). Bold fond marks metabolites which were also Zn-regulated in young leaves. [b] The patterns of trimethylsilyl (TMS) and methyloxime (MEOX) substitutions. [c]RI, Kovach retention index calculated from the retention times of alkane elution standards. [d] The MS signal (*m/z* value) refering to the most compound-characteristic fragment ion selected for quantification by integration of peak areas in characteristic extracted ion chromatograms. [e]FC, fold change (at least 1.5-fold) in metabolite relative abundances in the roots of Zn-treated plants in comparison to the controls. [f] T-test: p-value ≤ 0.05. Bold font and symbol "/" marks p-values calculated without and with false discovery rate (FDR) correction by Benjamini-Hochberg method and did not exceed the p-value threshold 0.05 after application of the FDR correction, [g] metabolites ≥1.5-fold increasing their abundance in roots upon the treatment with Zn^{2+} in comparison to the controls and [h] metabolites ≥1.5-fold decreasing their abundance in roots upon the treatment with Zn^{2+} in comparison to the controls.

Table S1(4). Individual metabolites identified as Zn^{2+}-dependently regulated in mature leaves of *A. caudates.*

#	Metabolite feature[a]	Derivative[b]	RI[c]	*m/z*[d]	FC[e]	*p*[f]
	Metabolites increasing their abundance in response to Zn^{2+} treatment[g]					
1	Malic acid	3TMS	1486.9	233	2.7	0.006
2	Erythronic acid	4TMS	1540.9	292	2.0	0.037
3	2-Deoxytetronic acid (3,4-Dihydroxybutanoic acid)	3TMS	1432.1	233	1.5	0.018
4	Gluconic acid δ-lactone	4TMS	1878.5	319	1.9	0.021
5	RI1747 Sugar-derived acid	-	1747.1	333	1.7	0.039
6	RI1811 Sugar-derived acid	-	1811.2	292	1.9	0.004
7	RI1984 Sugar-derived acid	-	1983.9	333	2.7	0.014
8	RI1889 Sugar-derived acid	-	1888.9	217	2.0	0.026
9	RI2006 Sugar-derived acid	-	2005.7	333	2.2	0.017
10	Salicylic acid	2TMS	1504.2	267	8.7	0.002
11	*p*-Coumaric acid	2TMS	1784.4	293	2.5	0.017
12	*trans*-2-hydroxycinnamic acid (*o*-Coumaric acid)	2TMS	1933.7	293	2.7	0.025
13	2-Hydroxysebacic acid	2TMS	2525.2	317	2.9	0.003
14	Carbodiimide	2TMS	1012	186	1.6	0.051
15	N,N-Dimethylglycine	1TMS	1040.4	58	1.7	0.015
16	Guanosine	5TMS	2642.8	324	10.0	0.017
17	5-Methylcytosine	2TMS	1534	254	3.8	0.050
18	Fructofuranose	5TMS	1798.7	217	3.3	0.022
19	Fructose 1	1MEOX,5TMS	1934.8	217	2.8	0.032
20	Fructose 2	1MEOX,5TMS	1942.9	217	2.9	0.026
21	Galactose	1MEOX,5TMS	1979.6	319	3.9	0.025
22	Glucose 1	1MEOX,5TMS	1990.5	319	2.9	0.006
23	Glucose 2	1MEOX,5TMS	2007.2	319	2.6	0.029
24	RI1808 C6-Monosaccharide	-	1807.9	437	3.1	0.018
25	RI1915 C6-Monosaccharide	-	1915.4	275	10.6	0.003
26	RI1949 Monosaccharide	-	1949.3	361	3.1	0.037
27	RI2035 Monosaccharide	-	2035.3	361	2.3	0.032
28	Sucrose	8TMS	2540.8	361	2.2	0.018
29	RI2344 Disaccharide	-	2344.3	204	2.2	0.001
30	RI2494 Disaccharide	-	2493.7	361	5.1	0.003
31	RI2599 Disaccharide	-	2599.1	361	1.8	0.017
32	RI2600 Disaccharide	-	2599.8	204	1.8	0.019
33	RI2676 Di- or oligosaccharide	-	2676.3	204	2.4	0.027
34	RI2887 Oligosaccharide	-	2887.6	204	3.4	**≤0.001 / 0.03**
35	RI2932 Oligosaccharide	-	2931.8	361	4.9	0.002
36	2-O-Glycerol-α-D-galactopyranoside	6TMS	2283	204	1.8	0.004
37	RI1082 Unknown	-	1082.2	240	1.9	0.021
38	RI1367 Unknown	-	1367.3	133	1.8	0.039
39	RI1612 Unknown	-	1611.5	215	2.7	0.044
40	RI1859 Unknown	-	1858.8	245	3.7	0.028
41	RI1881 Unknown	-	1881.4	217	1.7	0.023
42	RI2256 Unknown	-	2256.2	326	4.9	0.017
43	RI2801 Unknown	-	2800.9	338	2.2	0.023
	Metabolites decreasing their abundance in response to Zn^{2+} treatment[h]					
1	Succinic acid	2TMS	1316.6	247	1.64	0.031
2	RI2680 Di- or oligosaccharide	-	2680.1	204	1.85	0.007
3	RI2755 Di- or oligosaccharide	-	2755.3	204	1.68	0.029
4	RI2783 Di- or oligosaccharide	-	2782.5	204	1.88	0.004
5	RI3091 Oligosaccharide	-	3091.5	259	7.89	0.037
6	Glycerol-3-phosphate	4TMS	1758.8	299	1.75	0.043
7	RI1319 Unknown	-	1319.6	283	2.01	0.049
8	RI1136 Unknown	-	1136	156	3.88	0.047
9	RI1835 Unknown	-	1835	369	1.52	0.026

Trimethylsilyl (TMS) and methyloxime (MEOX) derivatives of polar metabolites (i.e. individual features) were annotated in the dry methanolic extracts of *A. caudatus* **mature leaves** by untargeted GC-EI-Q-MS.

The *A. caudatus* plants were grown in hydroponic nutrient solution with or without (controls) supplementation of 300 µmol/L Zn^{2+} under controlled conditions (24/18 °C day/night, relative humidity of 70–75%) and 16:8 light-darkness regimen. The mature leaves were harvested from seven-week-old plants (*n* = 3, each replicate the pool of three samples). [a] Annotation of metabolite features relied on retention indices (RI), retention times (t_R), on co-elution with authentic standards and spectral similarity search against the in-house spectral library or NIST 08, National Institute of Standards and Technology) and GMD (Golm Metabolome Database) libraries (Supplementary information 1, Table S1(1)). Metabolites are arranged by the following chemical classes: organic acids, fatty acids, lysolipids, N-metabolites, monosaccharides, polyols, disaccharides, organic phosphates. The numbers in the names of the features representing the same metabolite indicate the order of their elution. The names of the metabolites annotated to chemical classes without assignment of exact chemical structure were built by the combination of the metabolite RI and the name of the chemical class. The annotation to specific chemical class was confirmed by corresponding class-specific characteristic signals (for example, *m/z* 299, 315 and 387 for saccharide-phosphates, *m/z* 361, 204 and 319 for disaccharides, *m/z* 292, 333 and 103 for sugar acids). Unidentified metabolites were designated as "unknowns" and are specified with their retention indices (RI). [b] the patterns of trimethylsilyl (abbreviated as TMS) and methyloxime (MEOX) substitutions. [c] RI, Kovach retention indices calculated from the retention times of alkane elution standards. [d] the MS signal (*m/z* value) refering to the most compound-characteristic fragment ion selected for quantification by integration of peak areas in the characteristic extracted ion chromatograms. [e] FC, fold change (at least 1.5-fold) in metabolite relative abundances in Zn-treated mature leaves compared to the controls. [f] t-test p-value≤0.05. Bold font and symbol “/” marks p-values calculated without and with false discovery rate (FDR) correction by Benjamini-Hochberg method and did not exceed the p-value threshold 0.05 after application of the FDR correction. [g]metabolites ≥1.5-fold increasing their abundance in mature leaves upon treatment with Zn^{2+} in comparison to the controls and [h] metabolites ≥1.5-fold decreasing their abundance in mature leaves upon treatment with Zn^{2+} in comparison to the controls.

Table S1(5). Structures of common fragments in EI spectra of carbohydrates (Harvey and Vougor, 2019).*

m/z	**Structure**
191	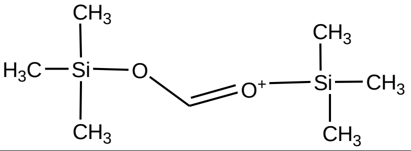
204	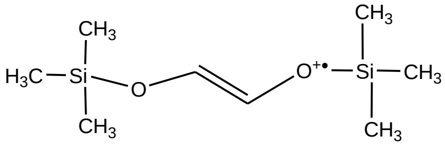
205	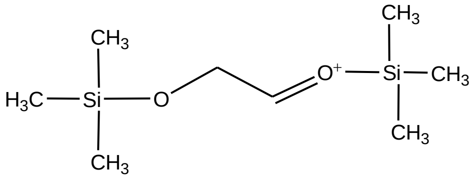
217	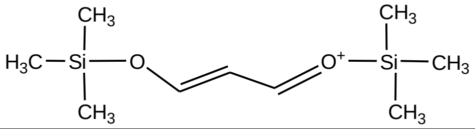
319	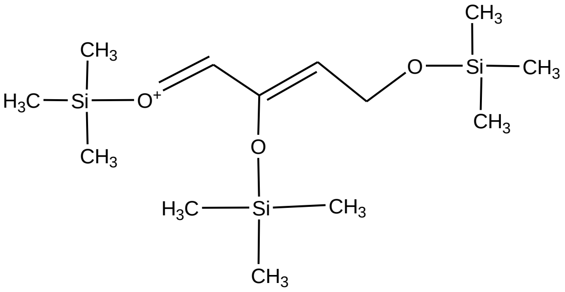

* EI spectra of carbohydrates presented in Supplementary Information 1, Figures S1(7,8).

Table S1(6). Annotation of the Zn-responsive metabolic pathways in *A. caudatus* **young leaves** [a].

#	Metabolic pathway[b]	Total Cmpd[c]	Hits[d]	Raw *p*[e]	-LOG(*p*)	Holm adjust[f]	FDR[g]	Impact[h]
1	Biosynthesis of unsaturated fatty acids	22	4 (octadecanoic acid; icosanoic acid; (9Z)-octadecenoic acid; linoleate)	3.22E-07	14.95	7.74E-06	7.74E-06	0
2	Fatty acid biosynthesis	56	4 (caprilic acid, myristic acid, stearic acid, oleic acids)	1.04E-04	9.17	2.39E-03	1.25E-03	0
3	Linoleic acid metabolism	4	1 (linoleic acid)	2.65E-03	5.93	5.83E-02	1.90E-02	1.0
4	Pentose phosphate pathway	19	1 (gluconic acid)	3.17E-03	5.76	6.65E-02	1.90E-02	0
5	Glutathione metabolism	26	1 (pyroglutamic acid)	5.95E-03	5.12	1.19E-01	2.51E-02	0.01
6	Citrate cycle (TCA cycle)	20	1 (succinate)	1.12E-02	4.49	2.13E-01	2.51E-02	0.04
7	Sulfur metabolism	15	1 (succinate)	1.12E-02	4.49	2.13E-01	2.51E-02	0.03
8	Alanine, aspartate and glutamate metabolism	22	1 (succinate)	1.12E-02	4.49	2.13E-01	2.51E-02	0
	Propanoate metabolism	20	1 (succinate)	1.12E-02	4.49	2.13E-01	2.51E-02	0
	Butanoate metabolism	17	1 (succinate)	1.12E-02	4.49	2.13E-01	2.51E-02	0
9	Cutin, suberine and wax biosynthesis	18	2 ((9Z)-octadecenoic acid, docosanoic acid)	1.22E-02	4.41	2.13E-01	2.51E-02	0.12
10	Glyoxylate and dicarboxylate metabolism	29	2 (glyoxylate, succinate)	1.26E-02	4.38	2.13E-01	2.51E-02	0.3
11	Purine metabolism	63	1 (glyoxylate)	1.54E-02	4.18	2.13E-01	2.63E-02	0
	Glycine, serine and threonine metabolism	33	1 (glyoxylate)	1.54E-02	4.18	2.13E-01	2.63E-02	0
12	Glycerolipid metabolism	21	1 (glycerol 3-phosphate)	1.69E-02	4.08	2.13E-01	2.70E-02	0.01
13	Amino sugar and nucleotide sugar metabolism	50	2 (fructose, mannose)	2.27E-02	3.79	2.13E-01	3.41E-02	0
14	Glycerophospholipid metabolism	37	2 (glycerol 3-phosphate, ethanolamine)	2.42E-02	3.72	2.13E-01	3.41E-02	0.1
15	Fructose and mannose metabolism	20	3 (fructose, mannose, glucose)	2.81E-02	3.57	2.13E-01	3.55E-02	0.04

16	Galactose metabolism	27	5 (glucose, fructose, galactose,mannose, myo-inositol)	2.81E-02	3.57	2.13E-01	3.55E-02	0.05
17	Starch and sucrose metabolism	22	3 (sucrose, glucose, fructose)	2.99E-02	3.51	2.13E-01	3.59E-02	0.4
18	Phenylalanine, tyrosine and tryptophan biosynthesis	22	1 (shikimate)	4.13E-02	3.19	2.13E-01	4.43E-02	0.08
19	Inositol phosphate metabolism	28	1 (*myo*-inosytol)	4.43E-02	3.12	2.13E-01	4.43E-02	0.1
20	Phosphatidylinositol signaling system	26	1 (*myo*-inosytol)	4.43E-02	3.12	2.13E-01	4.43E-02	0.03
21	Ascorbate and aldarate metabolism	18	1 (*myo*-inosytol)	4.43E-02	3.12	2.13E-01	4.43E-02	0

[a] The table presents the pathway analysis results calculated in Metaboanalist 5.0 (https://www.metaboanalyst.ca/). [b] The annotation of metabolic pathways for identified metabolites in *A. caudatus* young leaves (Table 1) relied on Kyoto Encyclopedia of Genes and Genomes (KEGG) pathway library of the model plant *Arabidopsis thaliana* L. The annotated pathways are ranked according to the results of pathway enrichment analysis and are available in Supplementary Information 3, Part 1. Grey backgroung marks the pathways with calculated pathway impact value ≥0.1 which might be considered as potential contributors to Zn-induced metabolic response; [c] Total Cmpd is the total number of compounds in the pathway; [d] Hits is the actually matched number metabolites from the uploaded identified metabolite data (Table 1); [e]Raw *p* is the original *p*-value calculated from the enrichment analysis (details on the analysis provided in Supplementary Information 4); [f] The Holm *p* is the *p*-value adjusted by Holm-Bonferroni method; [g] FDR *p* is the *p*-value adjusted according to the false discovery rate; [h]Impact is the pathway impact value calculated from pathway topology analysis (details on the analysis provided in Supplementary Information 4). Vizualization of the pathway analysis results are presented in Figure 6a.

Table S1(7). Annotation of Zn-responsive metabolic pathways in *A. caudatus* **roots** [a].

#	Metabolic pathway[b]	Total Cmpd[c]	Hits[d]	Raw *p*[e]	-LOG (*p*)	Holm adjust[f]	FDR[g]	Impact[h]
1	Glycerolipid metabolism	21	2 (glycerate, glycerol 3-phosphate)	1.68E-06	13.30	5.04E-05	2.89E-05	0.01
2	Fructose and mannose metabolism	20	2 (fructose, mannose)	2.30E-06	12.98	6.68E-05	2.89E-05	0.04
3	Glyoxylate and dicarboxylate metabolism	29	4 (oxalate, citrate, succinate,glycerate)	3.79E-06	12.48	1.06E-04	2.89E-05	0.05
4	Starch and sucrose metabolism	22	3 (trehalose, glucose, fructose)	3.86E-06	12.47	1.06E-04	2.89E-05	0.3
5	Inositol phosphate metabolism	28	2 (*myo*-inositol, inositol 1-phosphate)	1.96E-05	10.84	5.10E-04	9.81E-05	0.1
6	Phosphatidylinositol signaling system	26	2 (*myo*-inositol, inositol 1-phosphate)	1.96E-05	10.84	5.10E-04	9.81E-05	0.03
7	Citrate cycle (TCA cycle)	20	2 (succinate, citrate)	6.04E-05	9.71	1.45E-03	2.59E-04	0.2
8	Galactose metabolism	27	5 (glucose, fructose, galactose,mannose, *myo*-inositol)	1.05E-04	9.16	2.42E-03	3.95E-04	0.04
9	Amino sugar and nucleotide sugar metabolism	50	3 (fructose, arabinose, glucose, *myo*-inositol,)	1.62E-04	8.73	3.57E-03	5.41E-04	0.01
10	Pentose phosphate pathway	19	1(gluconic acid)	2.13E-04	8.46	4.46E-03	6.38E-04	0
11	Glycine, serine and threonine metabolism	33	1 (glycerate)	3.00E-04	8.11	6.01E-03	8.19E-04	0.02
12	Glycerophospholipid metabolism	37	2(Ethanolamine, glycerol 3-phosphate)	4.83E-04	7.64	9.17E-03	1.19E-03	0.1
13	Phenylalanine, tyrosine and tryptophan biosynthesis	22	1(shikimate)	5.16E-04	7.57	9.28E-03	1.19E-03	0.08
14	Arginine and proline metabolism	34	1(proline)	6.24E-04	7.38	1.06E-02	1.26E-03	0.07
15	Sulfur metabolism	15	1(succinate)	7.15E-04	7.24	1.14E-02	1.26E-03	0.03
16	Propanoate metabolism	20	1(succinate)	7.15E-04	7.24	1.14E-02	1.26E-03	0
	Butanoate metabolism	17	1(succinate)	7.15E-04	7.24	1.14E-02	1.26E-03	0
17	Aminoacyl-tRNA biosynthesis	46	3 (leucine, alanine, proline)	7.68E-04	7.17	1.14E-02	1.28E-03	0
18	Alanine, aspartate and glutamate metabolism	22	2 (alanine, succinate)	8.60E-04	7.06	1.14E-02	1.36E-03	0
19	Ascorbate and aldarate metabolism	18	1 (*myo*-inositol)	1.32E-03	6.63	1.45E-02	1.98E-03	0

20	Steroid biosynthesis	45	1 (beta-sitosterol)	2.69E-03	5.92	2.69E-02	3.84E-03	0.007
21	Pyrimidine metabolism	38	1 (uridine)	4.67E-03	5.37	4.20E-02	6.37E-03	0.03
22	Cutin, suberine and wax biosynthesis	18	1 (docosanoic acid)	5.85E-03	5.14	4.68E-02	7.63E-03	0
23	Valine, leucine and isoleucine biosynthesis	22	2 (2-methylmaleate, leucine)	1.32E-02	4.32	9.27E-02	1.65E-02	0.02
24	C5-Branched dibasic acid metabolism	6	1 (2-methylmaleate)	1.80E-02	4.02	1.08E-01	2.16E-02	0.5
25	Glutathione metabolism	26	1 (pyroglutamic acid)	2.38E-02	3.74	1.19E-01	2.75E-02	0.01
26	Selenocompound metabolism	13	1 (alanine)	4.03E-02	3.21	1.61E-01	4.23E-02	0
	Carbon fixation in photosynthetic organisms	21	1 (alanine)	4.03E-02	3.21	1.61E-01	4.23E-02	0
27	Valine, leucine and isoleucine degradation	37	1 (leucine)	4.23E-02	3.16	1.61E-01	4.23E-02	0

[a] The table presents the Pathway analysis results calculated in Metaboanalist 4.0 (https://www.metaboanalyst.ca/). [b] The annotation of metabolic pathways for identified metabolites in *A. caudatus* roots (Table 2) relied on Kyoto Encyclopedia of Genes and Genomes (KEGG) pathway library of the model plant *Arabidopsis thaliana* L. The annotated pathways are ranked according to results of pathway enrichment analysis and are available in Supplementary Information 3, Part 2. The grey backgroung marks the pathways with calculated pathway impact value ≥0.1 which might be considered as potential contributors to Zn-induced metabolic responses; [c] Total Cmpd is the total number of compounds in the pathway; [d] Hits is the actually matched number metabolites from the uploaded identified metabolite data (Table 2). [e] Raw *p* is the original *p*-value calculated from the enrichment analysis (details on the analysis provided in Supplementary Information 4); [f] The Holm *p* is the *p*-value adjusted by Holm-Bonferroni method; [g] FDR *p* is the *p*-value adjusted with consideration of the false discovery rate; [h] Impact is the pathway impact value calculated from pathway topology analysis (details on the analysis provided in Supplementary Information 4). Vizualization of the pathway analysis results are presented in Figure 6b.

Table S1(8). Annotation of the Zn-responsive metabolic pathways in *Amaranthus caudatus* **mature leaves** [a].

#	Metabolic pathway[b]	Total Cmpd[c]	Hits[d]	Raw *p*[e]	-LOG(*p*)	Holm adjust[f]	FDR[g]	Impact [h]
1	Citrate cycle (TCA cycle)	20	2 (malate, succinate)	6.9E-04	3.16	1.11E-02	5.55E-03	0.07
2	Glyoxylate and dicarboxylate metabolism	29	2 (malate, succinate)	6.9E-04	3.16	1.11E-02	5.55E-03	0.06
3	Pyruvate metabolism	22	1 (malate)	6.3E-03	2.20	8.84E-02	2.51E-02	0.2
4	Carbon fixation in photosynthetic organisms	21	1 (malate)	6.3E-03	2.20	8.84E-02	2.51E-02	0.06
5	Glycolysis / Gluconeogenesis	26	1 (glucose)	9.4E-03	2.03	1.13E-01	2.51E-02	0.001
6	Fructose and mannose metabolism	20	2 (glucose, fructose)	9.4E-03	2.03	1.13E-01	2.51E-02	0
7	Purine metabolism	63	1 (guanosine)	1.7E-02	1.77	1.69E-01	3.22E-02	0
8	Galactose metabolism	27	4 (galactose, glucose, sucrose, fructose)	1.8E-02	1.75	1.69E-01	3.22E-02	0.08
9	Starch and sucrose metabolism	22	3 (sucrose, glucose, fructose)	1.8E-02	1.74	1.69E-01	3.22E-02	0.09
10	Amino sugar and nucleotide sugar metabolism	50	4 (fructose, glucose, galactose, gluconolactone)	2.2E-02	1.66	1.69E-01	3.54E-02	0
11	Sulfur metabolism	15	1 (succinate)	3.1E-02	1.51	1.87E-01	3.57E-02	0.03
12	Alanine, aspartate and glutamate metabolism	22	1 (succinate)	3.1E-02	1.51	1.87E-01	3.57E-02	0
	Propanoate metabolism	20	1 (succinate)	3.1E-02	1.51	1.87E-01	3.57E-02	0
	Butanoate metabolism	17	1 (succinate)	3.1E-02	1.51	1.87E-01	3.57E-02	0
13	Glycerophospholipid metabolism	37	1 (glycerol 3-phosphate)	4.3E-02	1.37	1.87E-01	4.28E-02	0.09
14	Glycerolipid metabolism	21	1 (glycerol 3-phosphate)	4.3E-02	1.37	1.87E-01	4.28E-02	0.01

[a] The table presents the pathway analysis results calculated in Metaboanalist 4.0 (https://www.metaboanalyst.ca/). [b] The annotation of metabolic pathways for identified metabolites in *A. caudatus* mature leaves (Supplementary Information 1, Table S1(4)) relied on Kyoto Encyclopedia of Genes and Genomes (KEGG) pathway library of the model plant *Arabidopsis thaliana* L. The annotated pathways are ranked according to the results of pathway enrichment analysis and are available in Supplementary Information 3, Part 3. The grey backgroung marks the pathways with calculated pathway impact value ≥0.05 which might be considered as potential contributors to Zn-induced metabolic response; [c]Total Cmpd is the total number of compounds in the pathway; [d] Hits is the actually matched number metabolites from the uploaded identified metabolite data (Table S1(4)); [e]Raw *p* is the original *p*-value calculated from the enrichment analysis (details on the analysis provided in Supplementary Information 4); [f] The Holm *p* is the

p-value adjusted by Holm-Bonferroni method; [g] FDR *p* is the *p*-value adjusted with consideration of the false discovery rate; [h] Impact is the pathway impact value calculated from pathway topology analysis (details on the analysis provided in Supplementary Information 4). Vizualization of the Pathway analysis results are presented in Figure S1(10).

Table S1(9). Changes in the contents of metabolites detected by GC-EI-Q-MS-based targeted analysis in **mature leaves** of *A. caudatus* in response to Zn^{2+} treatment.

Metabolite[a]	Average contents, µmol/g DW[b]				ML (Zn-treated v. cont.)	
	ML cont		ML Zn			
	µmol/g DW	± StD	µmol/g DW	± StD	FC[c]	[d]*p*
Metabolites increasing their abundance in mature leaves upon treatment with Zn^{2+}						
Malic acid (3TMS)	2.70	0.27	6.83	1.04	**↑2.5**	**0.01**
Erythronic acid (4TMS)	2.90	0.27	5.37	1.22	**↑1.8**	**0.04**
Galactose (1MEOX, 5TMS)	0.04	0.02	0.15	0.04	**↑3.6**	**0.02**
Glucose (1MEOX, 5TMS)	0.27	0.04	0.74	0.14	**↑2.8**	**0.01**
Sucrose (8TMS)	4.24	0.44	9.83	2.21	**↑2.3**	**0.02**
Metabolites decreasing their abundance in mature leaves upon treatment with Zn^{2+}						
Succinic acid (2TMS)	4.82	0.51	2.89	0.63	**↓1.7**	**0.03**
Metabolites demonstrating no significant changes in their contents						
Malonic acid (2TMS)	0.85	0.18	0.89	0.17	↑≤1.3	0.78
Fumaric acid (2TMS)	0.58	0.06	0.65	0.17	↑≤1.3	0.53
Citric acid (4TMS)	0.86	0.95	0.40	0.22	↓2.2	0.56
Aconitic acid (3TMS)	2.88	0.82	2.32	0.43	↓≤1.3	0.45
Oxalic acid (2TMS)	23.1	2.95	14.6	18.5	↓1.6	0.46
Benzoic acid (TMS)	11.7	8.81	8.8	0.54	↓1.3	0.14
Isoleucine (2TMS)	0.13	0.11	0.08	0.04	↓1.6	0.61
Valine (2TMS)	0.52	0.19	0.17	0.01	↓3.0	0.09
Alanine (2&3TMS)	4.38	1.31	2.99	0.93	↓1.5	0.29
Pyroglutamic acid (1&2TMS)	4.11	1.96	7.3	4.78	↑1.8	0.35
Glycerol (3TMS)	0.90	0.29	1.53	0.13	↑1.7	0.07
Arabinose (4TMS)	0.18	0.04	0.32	0.07	↑1.7	0.06
myo-Inositol (6TMS)	0.15	0.02	0.25	0.10	↑1.6	0.19

The analysis relied on the targeted GC-EI-Q-MS assay; [a] list of trimethylsilyl (TMS) and methyloxime (MEOX) derivatives of the metabolites demonstrating a significant increase in tissue contents (up-regulation), significant decrease in tissue contents (down-regulation) or demonstrated no significant changes in mature leaves of *A. caudatus* in response to Zn^{2+} treatment in comparison to the controls. [b]The average contents of metabolites in µmol/g DW (dry weight) and their standard deviations (StD) found in mature leaves of the control plants (ML cont) and in the mature leaves of the plants exposed to

Zn^{2+} (ML Zn) are presented. [c]FC, fold change (not less than 1.3-fold) in the metabolite contents in Zn-treated samples compared with controls. Arrows indicate increased (↑) or decreased (↓) contents of the indicated metabolite in the Zn-treated sample compared with controls. The metabolites were defined as those Zn-dependently increasing of decreasing their abundance, when their tissue contents in mature leaves of Zn^{2+}-treated plants, increased or decreased in comparison to the untreated controls more than 1.3-fold, respectively. Statistically significant (t-test, $p \leq 0.05$) differences are marked in bold font. FC value 1.0 indicates no difference between compared Zn-treated and untreated samples

Table S1(10). Gas chromatographic (GC) separation conditions and electron ionization-quadrupole-mass spectrometry (EI-Q-MS) settings for analysis of *Amaranthus caudatus* primary polar thermally stabile metabolites.

**Parameters**	**Setting**
	**GC settings**
Separation column	[illegible] capillary column (30 m × 0.25 mm ID, 0.25 µm film thickness, Thermo Fisher [illegible]tific, Bremen, Germany)
Carrier gas / carrier gas flow rate	Helium / 1 mL/min
Injector operation mode	Splitless mode (90 s splitless time)
Injector temperature	250°C
Temperature program	1 min at 40°C<br>ramp 15°C/min to 70°C<br>1 min at 70°C<br>ramp 6°C/min to 320°C<br>12 min at 320°C
**Parameters**	**MS settings**
Ionization mode	Electron ionization (EI)
Electron energy	70 eV
Operation mode	scanning at 0.34 sec $scan^{-1}$
m/z range	50–700

The analysis was accomplished with Shimadzu GC2010 gas chromatograph coupled online to a quadrupole mass selective detector Shimadzu GCMS QP2010 with CTC GC PAL Liquid Injector (Shimadzu Scientific Instruments, Australia).

Literature

Leonova, T., Ihling, C., Saoud, M., Frolova, N., Rennert, R., Wessjohann, L.A., Frolov, A., 2022. Does filter-aided sample preparation provide sufficient method linearity for quantitative plant shotgun proteomics? Front. Plant Sci. 13, 874761. https://doi.org/10.3389/fpls.2022.874761

Boldizsár, I., Füzfai, Z., & Molnár-Perl, I. (2011). Characteristic fragmentation patterns of trimethylsilyl and trimethylsilyl-oxime derivatives of plant disaccharides as obtained by gas chromatography coupled to ion-trap mass spectrometry. *Journal of chromatography. A*, *1218*(43), 7864–7868. https://doi.org/10.1016/j.chroma.2011.08.060

Füzfai, Z., Boldizsár, I., & Molnár-Perl, I. (2008). Characteristic fragmentation patterns of the trimethylsilyl and trimethylsilyl-oxime derivatives of various saccharides as obtained by gas chromatography coupled to ion-trap mass spectrometry. *Journal of chromatography. A*, *1177*(1), 183–189. https://doi.org/10.1016/j.chroma.2007.11.023

Harvey, D. J., and Vouros, P. (2020). Mass spectrometric fragmentation of trimethylsilyl and related alkylsilyl derivatives. *Mass Spectrom Rev* 39, 105–211. doi: 10.1002/mas.21590.

Kamerling J.P. and Vliegenthart J.F.G. (1972). Mass Spectrometry of Pertrimethylsilyl Oligosaccharides containing fructose units. Tetrahedron, 28, 4375-4387.
